# Supplementary material for: Harnessing indole scaffolds to identify small-molecule IRE1α inhibitors modulating XBP1 mRNA splicing
Source: Nat Commun. 2025 Sep 26;16:8531. doi: 10.1038/s41467-025-64291-4 (PMC12475274; doi:10.1038/s41467-025-64291-4)
Supplement: Supplementary file 1 — Supplementary Information [file 41467_2025_64291_MOESM1_ESM.pdf]

Supporting Information

Harnessing Indole Scaffold to Identify Small-molecule IRE1 Inhibitors Modulating *XBP1* mRNA Splicing

Yang Liu,[1,2,3,#] Amrutha K. Avathan Veettil,[1,2,3,#] Raphael Gasper,[4] Mao Jiang,[1,2,3] Leon Wagner,[1,2,3] Oguz Hastürk,[1,2,3] and Peng Wu[1,2,3,*]

[1]Chemical Genomics Centre, Max Planck Institute of Molecular Physiology, Dortmund 44227, Germany

[2]Department of Chemical Biology, Max Planck Institute of Molecular Physiology, Dortmund 44227, Germany

[3]Faculty of Chemistry and Chemical Biology, TU Dortmund University, Dortmund 44227, Germany

[4]Crystallography and Biophysics Unit, Max Planck Institute of Molecular Physiology, Dortmund 44227, Germany

[#]Equally contributing authors

[*]Correspondence, P. Wu, email: peng.wu@mpi-dortmund.mpg.de

CONTENTS

SUPPLEMENTARY DATA FIGURES ……………………………………………………….……... S3

SUPPLEMENTARY TABLES……………………………………………………………….… S13

GENERAL CHEMISTRY INFORMATION………………………………………………….….. S19

SYNTHETIC PROCEDURES AND COMPOUND CHARACTERIZATION………………………...S20

NMR SPECTRA ………….……………………………………………………………….…..…S48

LC TRACES …….……………………………………………………………...............................S103

REFERENCES ……………… ………………………………………...……………………....… S121

SUPPLEMENTARY DATA FIGURES

a

```
 550        560        570        580        590        600        610
GSSPSLEQDD GDEETSVVIV GKISFCPKDV LGHGAEGTIV YRGMFDNRDV AVKRILPECF SFADREVQLL

 620        630        640        650        660        670        680
RESDEHPNVI RYFCTEKDRQ FQYIAIELCA ATLQEYVEQK DFAHLGLEPI TLLQQTTSGL AHLHSLNIVH

 690        700        710        720        730        740        750
RDLKPHNILI SMPNAHGKIK AMISDFGLCK KLAVGRHSFS RRSGVPGTEG WIAPEMLSED CKENPTYTVD

 760        770        780        790        800        810        820
IFSAGCVFYY VISEGSHPFG KSLQRQANIL LGACSLDCLH PEKHEDVIAR ELIEKMIAMD PQKRPSAKHV

 820        830        840        850        860        870        880
PQKRPSAKHV LKHPFFWSLE KQLQFFQDVS DRIEKESLDG PIVKQLERGG RAVVKMDWRE NITVPLQTDL

 890        900        910        920        930        940        950
RKFRTYKGGS VRDLLRAMRN KKHHYRELPA EVRETLGSLP DDFVCYFTSR FPHLLAHTYR AMELCSHERL

 960        970
FQPYYFHEPP EPQPPVTPDA L
```

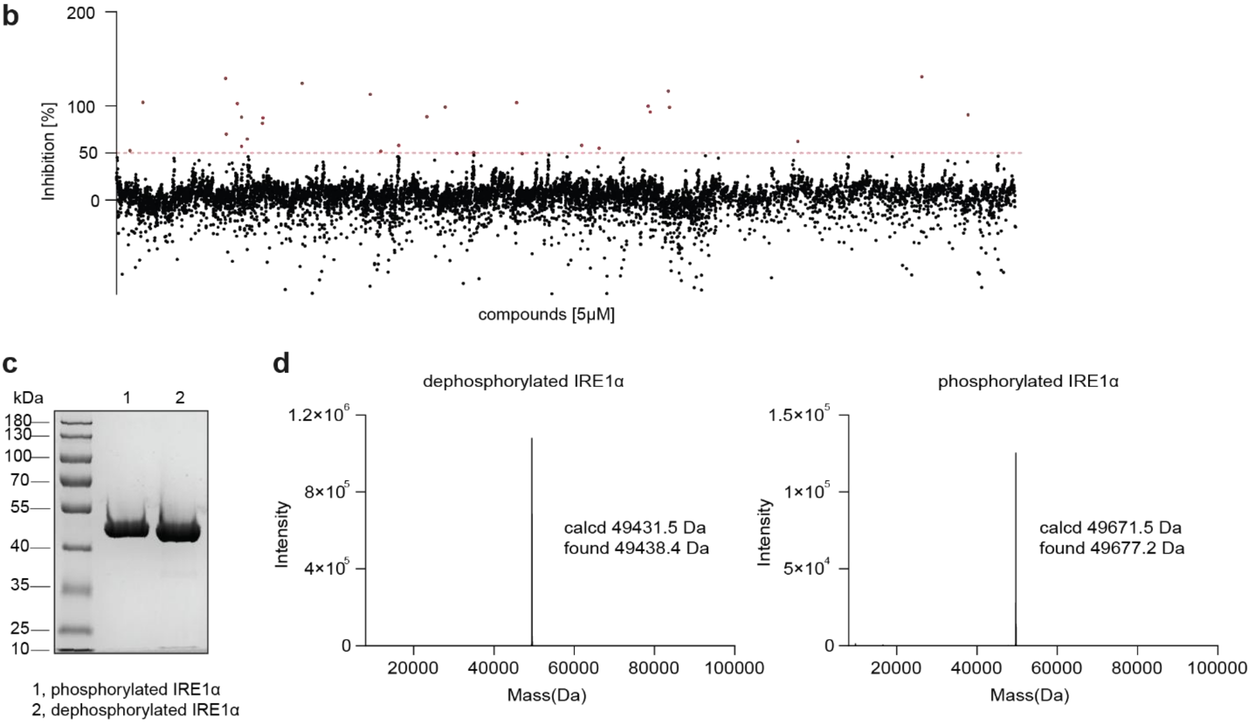

Supplementary Fig. 1. Identification of the IRE1α inhibitors. (a) The sequence of the truncated IRE1α (547-977). The kinase domain F571-F832 (according to UniProt) is shown in brown and the Kinase-extension nuclease (KEN) domain L835-H963 is shown in purple. Phosphorylation sites are shown in red. (b) In-house compound library screening, compounds with inhibition of >50% were identified as primary hits (red dots). (c) Coomassie blue SDS PAGE gel of the purified proteins. Representative result of n = 3 (d) Deconvoluted mass spectrums of dephosphorylated IRE1α and phosphorylated IRE1α. Dephosphorylated IRE1α calculated mass: 49431.5 Da, found 49438.4 Da. Phosphorylated IRE1α calculated mass: 49671.5 Da, found 49677.2 Da.

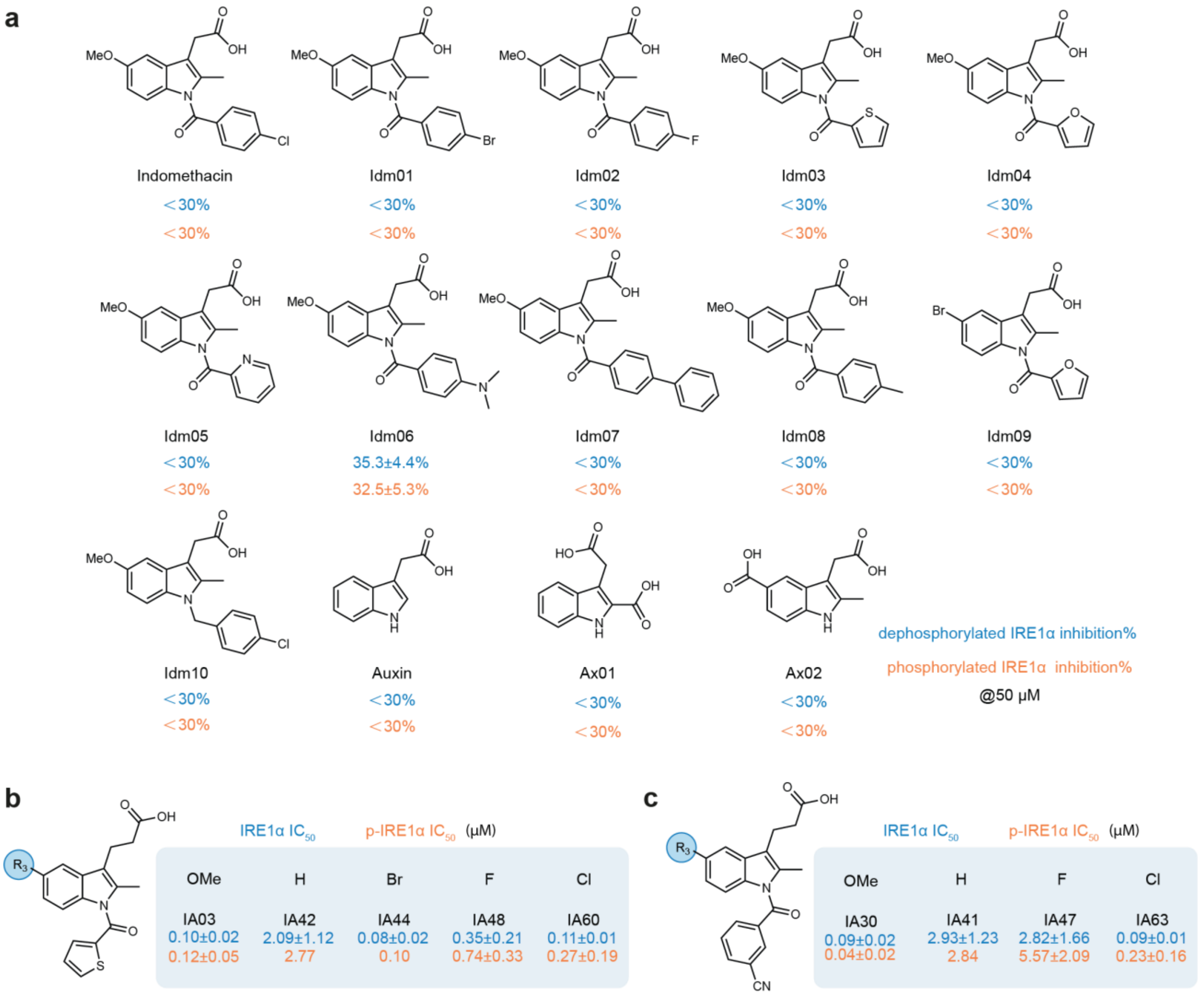

Supplementary Fig. 2. Structures of indomethacin, auxin, their analogues, and their IRE1α inhibitory activities. (a) Structures of indomethacin, auxin, and their analogues obtained from an in-house library and their activities (inhibition%) at 50μM based on the FRET assay. Data are presented as mean±SEM, n=3. (b) Analogues based on the thiophene-2carbonyl scaffold of IA03. All data are presented as mean±SEM n=3, except IA42 and IA44 p-IRE1α measurement, n=2. (c) Analogues based on the 3-cyanobenzoyl scaffold of IA30. All data are presented as mean±SEM n=3, except IA41 p-IRE1α measurement, n=2.

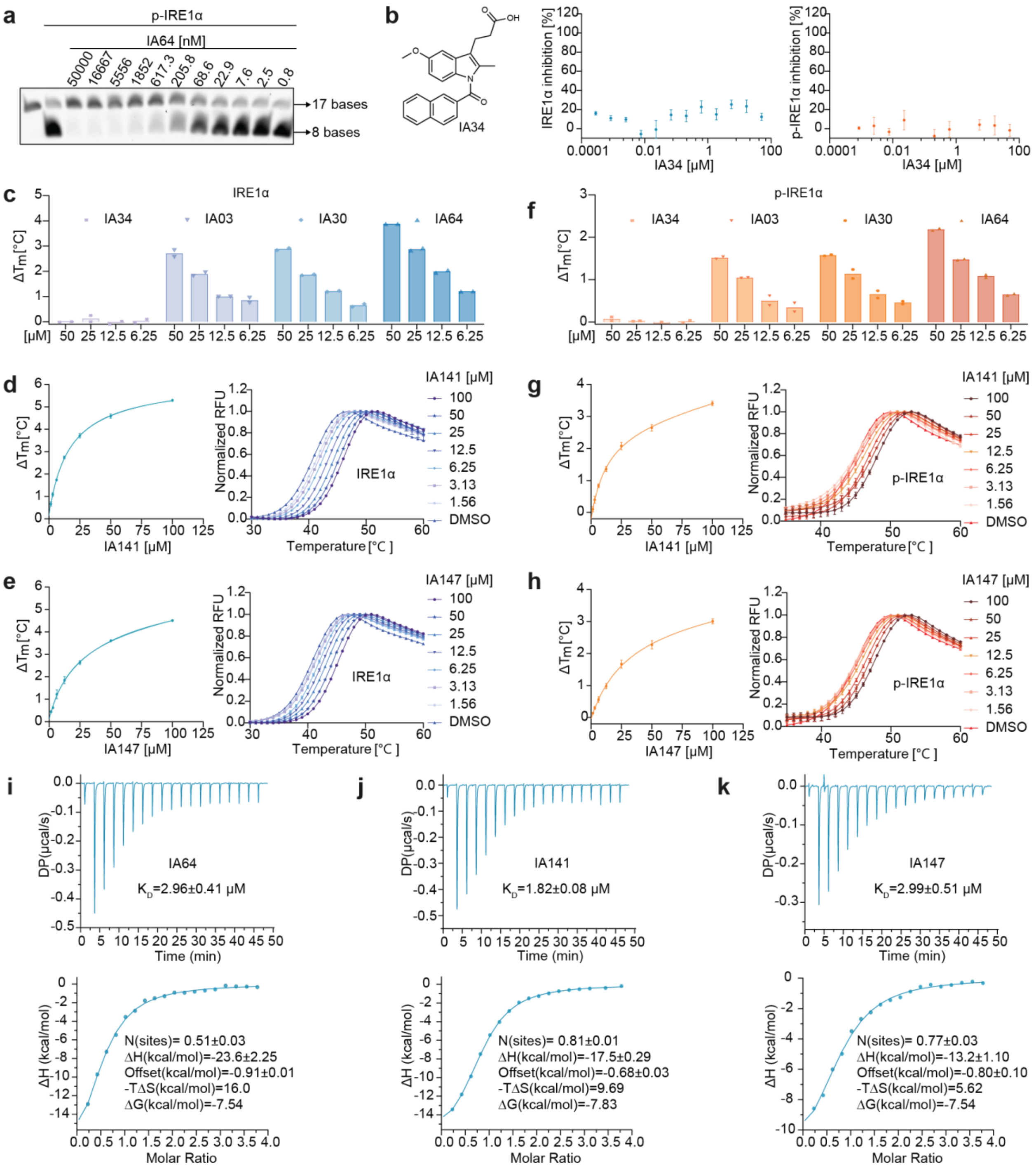

Supplementary Fig. 3. Evaluation of the inhibition and binding activity of the IRE1α inhibitors. (a) IA64 concentration-dependently inhibited p-IRE1α activity in the gel-based cleavage assay. Representative result of n = 3. (b) The negative control compound IA34 and its FRET activity against IRE1α and p-IRE1α. Data are presented as mean±SEM, n=3. (c) IRE1α protein ΔT_m values under the treatment of IA34, IA03, IA30, and IA64. Data are presented as mean±SD, n=2. (d) IRE1α protein ΔT_m values (left panel) and melting curve (right panel) under the treatment of different IA141 concentrations. Data are presented as mean ±

SEM, n=4. (e) IRE1α protein ΔT_m values (left panel) and melting curve (right panel) under the treatment of different IA147 concentrations. Data are presented as mean±SEM, n=4. (f) P-IRE1α ΔT_m values under the treatment of IA34, IA03, IA30 and IA64. Data are presented as mean±SD, n=2. (g) p-IRE1α ΔT_m values (left panel) and melting curve (right panel) under different concentrations of IA141. Data are presented as mean±SEM, n=4. (h) p-IRE1α ΔT_m values (left panel) and melting curve (right panel) under different concentrations of IA147. Data are presented as mean±SEM, n=4. (i) (j) (k) Binding affinity of IA64, IA141 and IA147 to IRE1α in ITC assay.

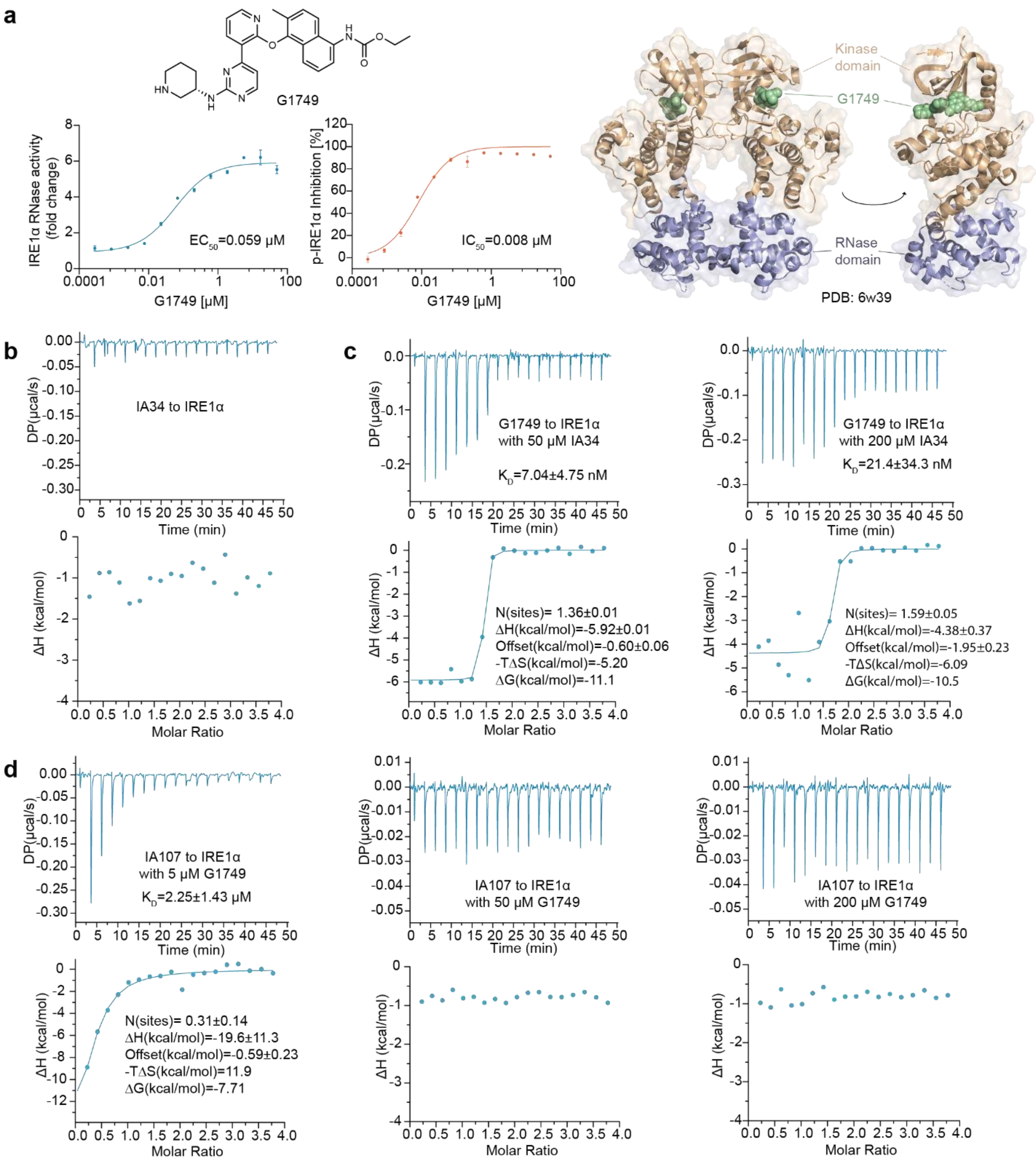

Supplementary Fig. 4. Evaluation of the inhibition and binding activity of IRE1α inhibitors. (a) The reported inhibitor G1749 binds to the kinase pocket of IRE1α, activating IRE1α activity while inhibiting p-IRE1α. Data are presented as mean±SEM, n=3. (b) ITC result of the negative control compound IA34. (c) ITC result of the G1749 titrated into IRE1α. IRE1α was pre-incubated with 50 μM and 200 μM IA34, respectively. (d) ITC result of IA107 titrated into IRE1α. IRE1α was pre-incubated with 5 μM, 50 μM, and 200 μM G1749, respectively.

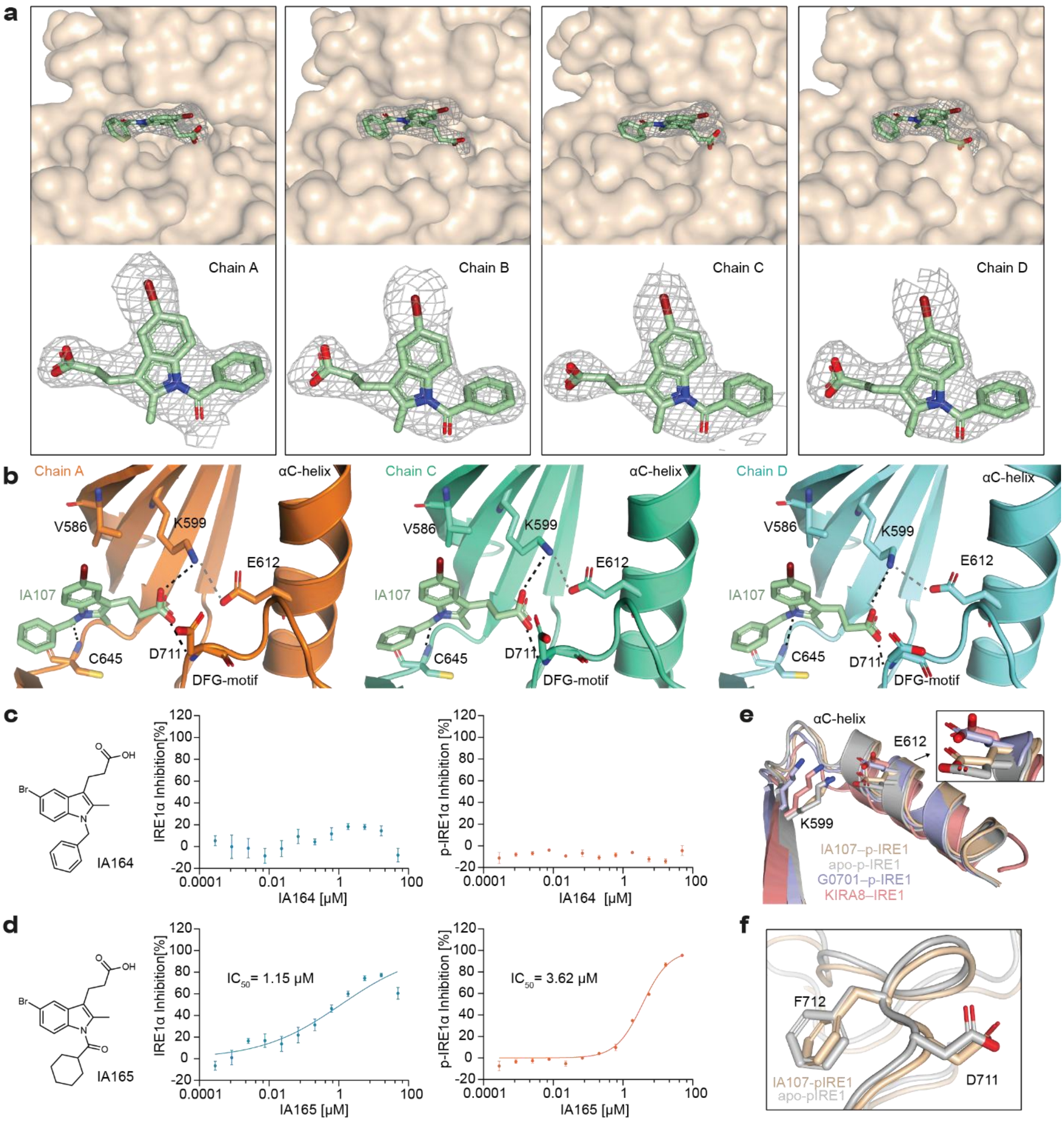

Supplementary Fig. 5. IA107-bound p-IRE1α complex structure. (a) 2Fo-Fc electron density maps around IA107 co-crystalized with p-IRE1α. Shown in σ 1.0. (b) Ligand interactions between chains A, C, and D. (c) Compound IA164 without the amid group did not show activity against IREα and p-IRE1 in the FRET assay. Data are presented as mean±SEM, n=3. (d) Compound IA165 with a cyclohexane group at the N-position decreased the inhibitory activity in the FRET assay. Data are presented as mean±SEM, n=3. (e) Superimposition of the αC-helix from the IA107-bound p-IRE1α (wheat, PDB 9gow), apo-pIRE1α (grey, PDB 6w3c), G0701-pIRE1α (purple, PDB 6w3e) and KIRA8-IRE1α (pink, 6urc). (f) Details of the DFG-motif conformation of the IA107–p-IRE1α complex (wheat) align with that of the apo-p-IRE1α (grey, PDB 6w3c).

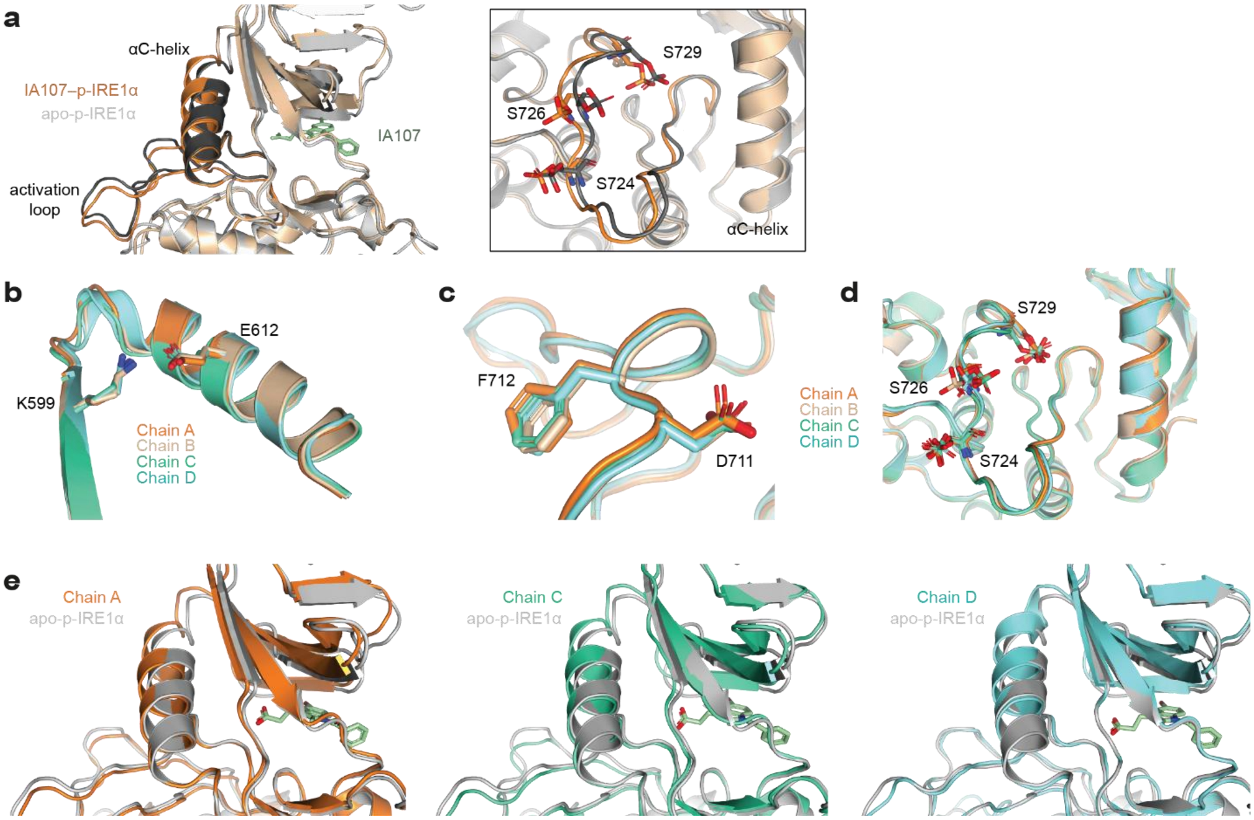

Supplementary Fig. 6. Interaction and conformation of other chains in the asymmetric unit of IA107–p-IRE1α. (a) Superimposition details of kinase domain αC-helix and the activation loop from the IA107–p-IRE1α (wheat, PDB 9gow) and apo-p-IRE1α (grey, PDB 6w3c). (b) Superimposition view of the αC-helix and K599-E612 salt bridge of all chains. (c) Superimposition view of the DFG motif of all chains. (d) Superimposition view of the activation loop and phosphorylation sites of all chains. (e) Superimposition view of the αC-helix from chains A, C, and D of the IA107–p-IRE1α with the apo-pIRE1α (grey, PDB 6w3c). Chain A is shown in orange, chain B in wheat, chain C in greencyan, and chain D in aquamarine.

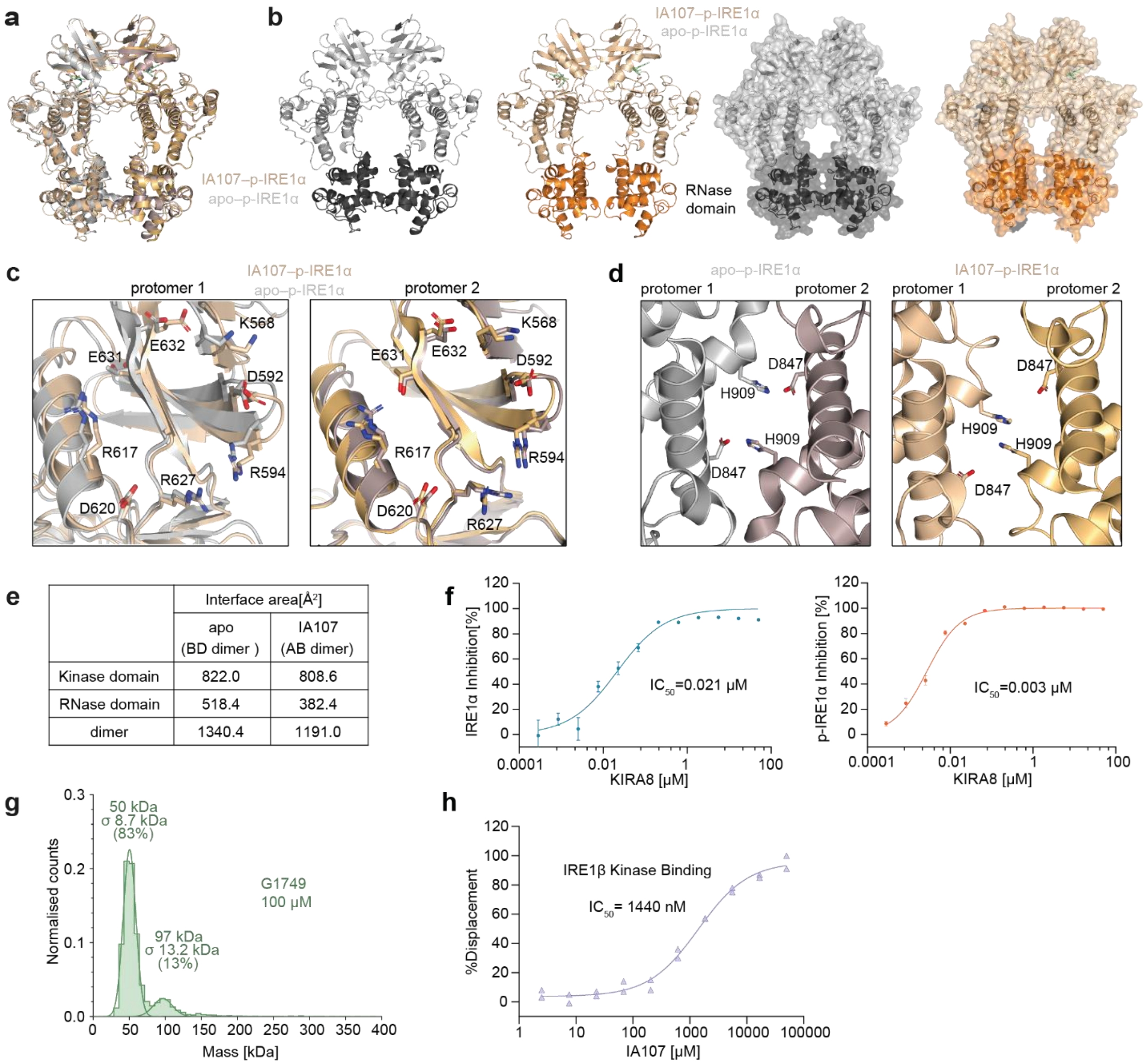

	Interface area[Å²]	
	apo (BD dimer)	IA107 (AB dimer)
Kinase domain	822.0	808.6
RNase domain	518.4	382.4
dimer	1340.4	1191.0

Supplementary Fig. 7. IA107-bound p-IRE1α complex structure, Part II. (a). Superimposition of the back-to-back dimers from the IA107–pIRE1α, chain A and chain B (wheat, PDB 9gow), and apo-p-IRE1α (grey, PDB 6w3c). (b) Cartoon and surface views of the back-to-back dimers of the apo-form p-IRE1α (grey, 6w3c) and the AB-dimer of IA107–p-IRE1α complex (wheat, 9gow). The RNase domains are highlighted in dark grey (apo-p-IRE1α) and orange (IA107–p-IRE1). (c) Superimposition views of the conformation of key residues in the kinase domain interface of the two protomers. (d) The residue H909 in the RNase domain of IA107–p-IRE1α is shifted in comparison with that of the apo-p-IRE1. The hydrogen bonds between D847 and H909 at the two protomers interface were broken. (e) Quantitative interface areas of apo-p-IRE1α (6w3c) and 107–p-IRE1α (9gow) dimer, data were calculated using the PDBePISA web server. (f) KIRA8 activity tested in-house. Data are presented as mean±SEM, n=3. (g) The reported p-IRE1α inhibitor G1749 inhibited the dimerization of p-IRE1. (h) IA107 bound to the IRE1β kinase domain with an IC_{50} of 1440 nM tested in the LanthaScreen™ Eu kinase binding assay (Thermo Fisher SelectScreen). n=2.

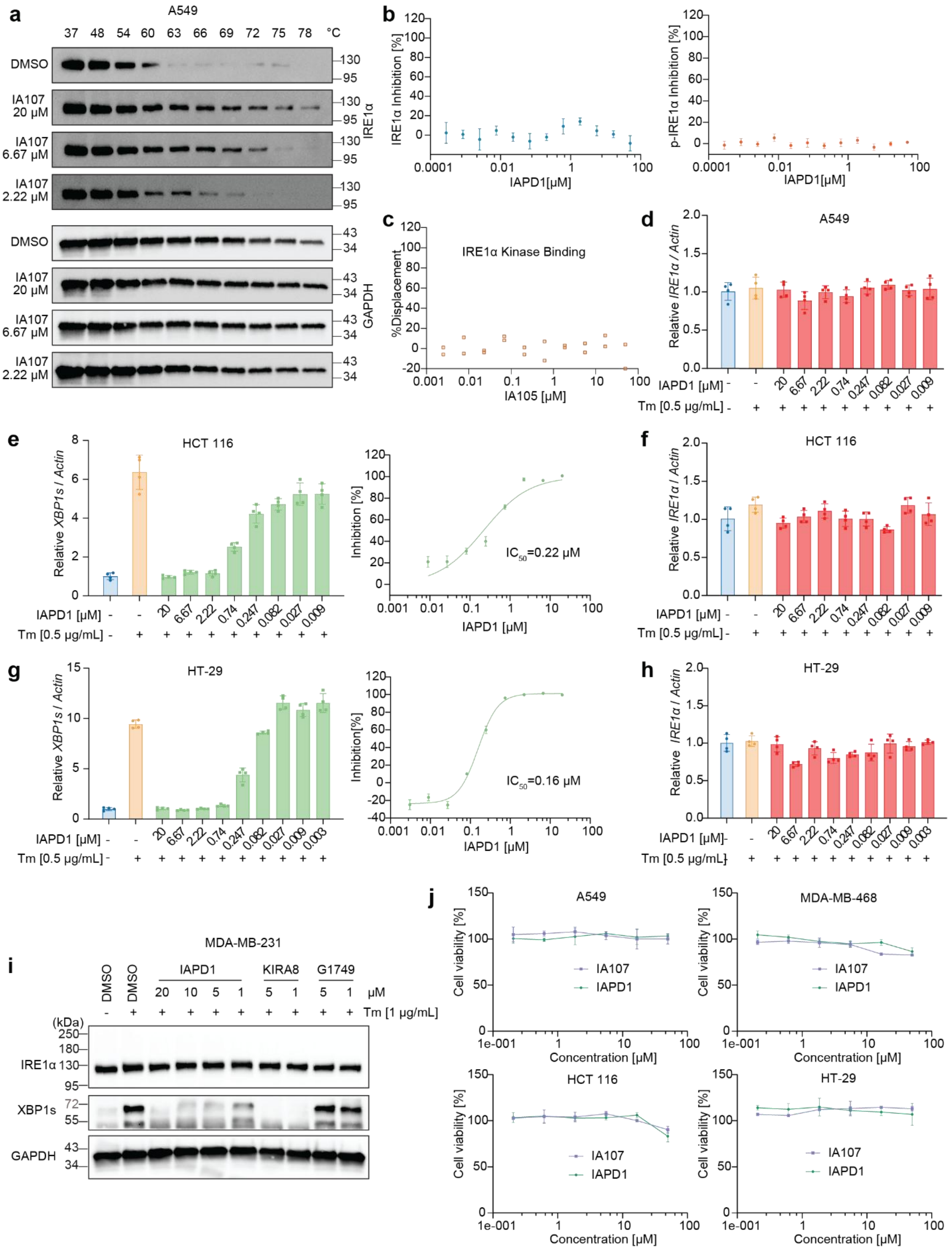

a
A549
37 48 54 60 63 66 69 72 75 78 °C
DMSO
IA107 20 µM
IA107 6.67 µM
IA107 2.22 µM
IRE1α
GAPDH
b
IRE1α Inhibition [%]
p-IRE1α Inhibition [%]
IAPD1[µM]
c
IRE1α Kinase Binding
%Displacement
IA105 [µM]
d
A549
Relative IRE1α / Actin
IAPD1 [µM]
Tm [0.5 µg/mL]
e
HCT 116
Relative XBP1s / Actin
Inhibition [%]
IC50=0.22 µM
IAPD1 [µM]
f
HCT 116
Relative IRE1α / Actin
g
HT-29
Relative XBP1s / Actin
Inhibition[%]
IC50=0.16 µM
h
HT-29
Relative IRE1α / Actin
i
MDA-MB-231
DMSO DMSO IAPD1 KIRA8 G1749
20 10 5 1 5 1 5 1 µM
Tm [1 µg/mL]
(kDa)
IRE1α
XBP1s
GAPDH
j
A549
MDA-MB-468
HCT 116
HT-29
Cell viability [%]
Concentration [µM]
IA107
IAPD1

Supplementary Fig. 8. Cellular evaluation of IA107 and the prodrug IAPD1. (a) IA107 concentration-dependently stabilized full-length IRE1α protein in A549 cell lysate. Representative result of n = 2 (b) Prodrug IADP1 did not inhibit the RNase activity of IRE1α and p-IRE1α in the FRET assay. Data are presented as mean±SEM, n=3. (c) Prodrug IADP1 did not show any detectable binding signal to the IRE1α kinase domain tested in the LanthaScreen™ Eu kinase binding assay (Thermo Fisher SelectScreen). n=2. (d) Treatment of IAPD1 and tunicamycin (Tm) has minimal impacts on the *IRE1α* transcription level in A549 cells. Data are presented as mean±SEM, n=4. (e) IAPD1 treatment inhibited the ER stress-induced *XBP1* mRNA splicing level in HCT 116 cells. Data are presented as mean±SEM, n=4. (f) IAPD1 treatment did not influence the *IRE1α* transcription level in HCT 116 cells. Data are presented as mean±SEM, n=4. (g) IAPD1 inhibited the ER stress-induced *XBP1* mRNA splicing level in HT 29 cells. Data are presented as mean±SEM, n=4. (h) IAPD1 did not influence the *IRE1α* transcription level in HT 29 cells. Data are presented as mean±SEM, n=4 (i) IAPD1 inhibited the XPB1s protein level in MDA-MB-231 cells by western blot assay. Representative result of n = 2. (j) IAPD1 and the carboxylic acid compound IA107 did not show cytotoxicity towards A549, MDA-MB-468, HCT 116, and HT-29 cell lines. Data are presented as mean±SEM, n=3.

SUPPLEMENTARY TABLES

Table S1, X-ray crystallography data collection and refinement statistics (molecular replacement)

	**p-IRE1 α /IA107**
**PDB ID**	9GOW
**Data collection**	
Wavelength (Å)	0.9677
Resolution (Å)	45.06-3.0 (3.107-3.0)
Space group	P1
Cell dimension	
a, *b*, *c* (Å)	67.18, 77.5, 141.06
α, *β*, *γ* (°)	74.607, 78.089, 65.634
R_{merge}	0.2049 (2.029)
R_{meas}	0.2388 (2.355)
I/σ(I)	6.14 (0.79)
$CC_{1/2}$	0.989 (0.301)
Completeness (%)	91.89 (92.1)
Redundancy	3.8 (3.9)
Wilson *B* (Å^2)	78.24
**Refinement**	
Resolution (Å)	3.0
Number of reflections	45705
R_{work} / R_{free}	0.2312 / 0.2806
Number of atoms	
Protein	12263
Ligand	156
B factors (Å^2)	85.58
Protein (Å^2)	85.58
Ligand (Å^2)	85.59
R.m.s. d	
Bond lengths (Å)	0.003
Bond angles (°)	0.57
Ramachandran (favored/allowed/outlier) (%)	94/6/0
Copies/ a.s.u.	4

The dataset was collected from a single crystal. Statistics for the highest-resolution shell are shown in parentheses. r.m.s.d., root mean square deviation; a.s.u., asymmetric unit.

Table S2, Kinase profiling data of IA107 at 5 μM, related to Fig. 6i.

Kinase	Technology	ATP(μM)	%Inhibition average	Dup Difference
ABL1	ZLYTE	Km app	3	1
ACVR1B (ALK4)	ZLYTE	Km app	-5	6
AKT1 (PKB alpha)	ZLYTE	Km app	0	0
AMPK A1/B1/G1	ZLYTE	Km app	9	2
AURKA (Aurora A)	ZLYTE	Km app	5	1
BTK	ZLYTE	Km app	8	2
CAMK1D (CaMKI delta)	ZLYTE	Km app	8	0
CDK1/cyclin B	ZLYTE	Km app	4	1
CHEK1 (CHK1)	ZLYTE	Km app	10	12
CLK2	ZLYTE	Km app	7	1
CSNK1G2 (CK1 gamma 2)	ZLYTE	Km app	6	1
CSNK2A1 (CK2 alpha 1)	ZLYTE	Km app	6	2
DAPK3 (ZIPK)	ZLYTE	Km app	1	2
DNA-PK	ZLYTE	Km app	-2	0
DYRK1A	ZLYTE	Km app	4	1
DYRK3	ZLYTE	Km app	2	0
EEF2K	ZLYTE	Km app	5	2
EGFR (ErbB1)	ZLYTE	Km app	5	7
EPHA2	ZLYTE	Km app	4	2
EPHB4	ZLYTE	Km app	3	3
ERBB2 (HER2)	ZLYTE	Km app	-3	1
FGFR1	ZLYTE	Km app	4	0
FLT3	ZLYTE	Km app	6	2
FRAP1 (mTOR)	ZLYTE	Km app	5	0
GSK3B (GSK3 beta)	ZLYTE	Km app	21	1
HIPK4	ZLYTE	Km app	6	5
IGF1R	ZLYTE	Km app	3	3
IKBKB (IKK beta)	ZLYTE	Km app	4	1
IKBKE (IKK epsilon)	ZLYTE	Km app	2	3

INSR	ZLYTE	Km app	5	0
JAK3	ZLYTE	Km app	0	3
KDR (VEGFR2)	ZLYTE	Km app	1	1
KIT	ZLYTE	Km app	0	0
LCK	ZLYTE	Km app	8	3
MAP2K1 (MEK1)	ZLYTE	100	3	1
MAP2K6 (MKK6)	ZLYTE	100	2	4
MAPK1 (ERK2)	ZLYTE	Km app	2	1
MAPK14 (p38 alpha)	ZLYTE	100	6	1
MAPK8 (JNK1)	ZLYTE	100	-8	1
MAPKAPK2	ZLYTE	Km app	5	1
MARK2	ZLYTE	Km app	8	3
MET (cMet)	ZLYTE	Km app	3	2
NEK1	ZLYTE	Km app	17	7
NTRK2 (TRKB)	ZLYTE	Km app	5	1
PAK4	ZLYTE	Km app	-3	4
PDGFRB (PDGFR beta)	ZLYTE	Km app	0	2
PHKG2	ZLYTE	Km app	26	11
PIM1	ZLYTE	Km app	39	1
PLK1	ZLYTE	Km app	3	0
PRKACA (PKA)	ZLYTE	Km app	9	5
PRKCB1 (PKC beta I)	ZLYTE	Km app	7	1
PRKD1 (PKC mu)	ZLYTE	Km app	0	2
PTK2 (FAK)	ZLYTE	Km app	4	0
RET	ZLYTE	Km app	9	3
ROCK1	ZLYTE	Km app	1	2
RPS6KA3 (RSK2)	ZLYTE	Km app	1	0
RPS6KB1 (p70S6K)	ZLYTE	Km app	3	1
SGKL (SGK3)	ZLYTE	Km app	1	1
SRC	ZLYTE	Km app	4	4
STK4 (MST1)	ZLYTE	Km app	0	5

SYK	ZLYTE	Km app	3	1
TAOK2 (TAO1)	ZLYTE	Km app	-4	9
TEK (Tie2)	ZLYTE	Km app	6	7
GSG2 (Haspin)	Adapta	Km app	4	11
ACVR1 (ALK2)	LanthaScreen Binding		2	3
BMPR1B (ALK6)	LanthaScreen Binding		12	1
BMPR2	LanthaScreen Binding		-4	4
BRSK2	LanthaScreen Binding		1	3
CAMKK2 (CaMKK beta)	LanthaScreen Binding		4	8
CASK	LanthaScreen Binding		-14	23
CDC7/DBF4	LanthaScreen Binding		8	9
CDK8/cyclin C	LanthaScreen Binding		35	11
DDR2	LanthaScreen Binding		-1	2
ERN1	LanthaScreen Binding		91	7
ERN2	LanthaScreen Binding		70	6
ICK	LanthaScreen Binding		-2	4
LIMK1	LanthaScreen Binding		-16	8
MAP3K11 (MLK3)	LanthaScreen Binding		-3	0
MAP3K14 (NIK)	LanthaScreen Binding		0	0
MAP4K1 (HPK1)	LanthaScreen Binding		-2	10
MAPK15 (ERK7)	LanthaScreen Binding		17	5
MYO3B (MYO3 beta)	LanthaScreen Binding		-2	2
RIPK2	LanthaScreen Binding		0	2
STK32B (YANK2)	LanthaScreen Binding		2	13
STK33	LanthaScreen Binding		-4	6
STK38 (NDR)	LanthaScreen Binding		6	3
TGFBR1 (ALK5)	LanthaScreen Binding		54	6
TLK1	LanthaScreen Binding		-12	0
TNIK	LanthaScreen Binding		8	4
TTK	LanthaScreen Binding		6	1
ULK2	LanthaScreen Binding		-3	7

Table S3, Sequence of used primers and oligonucleotides

Primers/oligos	Sequence (5’-3’)	Experiment	Source
Dual-labeled XBP1 hairpin	5′FAM-CAUGUCCGCAGCGCAUG-3′BHQ1	FRET, gel-based cleavage assay	IDT
IRE1α_G547_Fwd	CTGTATTTTCAGGGCGGATCCGGCAGCAGCCCCTCCC	subclone	IDT
IRE1α_L977_Rev	TCCTCTAGTACTTCTCGACAAGCTTTTAGAGGGCGTCTGGAGTCACTG	subclone	IDT
hActin Fwd	GCGAGAAGATGACCCAGATC	RT-qPCR	IDT
hActin Rev	CCAGTGGTACGGCCAGAGG	RT-qPCR	IDT
hXBP1s-Fwd	GAGTCCGCAGCAGGTG	RT-qPCR	IDT
hXBP1s-Rev	CAATACCGCCAGAATCCA	RT-qPCR	IDT
hIRE1α-Fwd	GTGAGCGACAGAATAGAAAAGG	RT-qPCR	IDT
hIRE1α-Rev	GGAAGCGAGATGTGAAGTAGC	RT-qPCR	IDT

Table S4, Small-molecule screening information

Category	Parameter	Description
Assay	Type of assay	Fluorescence resonance energy transfer (FRET), *in vitro*
	Target	IRE1α (ERN1)
	Primary measurement	Real-time fluorescence intensity, excitation 485, emission 535
	Assay protocol and reagents	Protocol and reagents are posted in the Method section
Library	Library size	11393 compounds at 10 mM in DMSO
	Library composition	Small molecules and pseudo-natural products
	Quality control	LC-MS and Tube Auditor™ inspection of the sample collection
	Source	Compound Management and Screening Center Dortmund
Screen	Format	384-well plate
	Concentration tested	5 μM, 0.05% DMSO
	Plate controls	Control: DMSO control; Blank control: only substrate
	Dispensing system	ECHO dispenser (Beckmann), Multidrop dispenser (Thermo)
	Detection instrument	TECAN spark
	Normalization	inhibition% = 100 × (average slope of DMSO control - slope of the sample)/ (average slope of DMSO control − average slope of blank control)
Post-HTS	Hit criteria	inhibition% ⩾ 50%
	Hit rate	48/11393, 0.42%
	Retesting of initial activities	Hits retested in dose-response mode using the screening assay
	Compound purity and structure	Resynthesized the hit compound and validated
	Structure confirmation	NMR, HRMS
	Additional assay(s)	Gel-based cleavage assay; DSF assay; ITC

GENERAL CHEMISTRY INFORMATION

The commercially available solvents and reagents were purchased from Sigma-Aldrich, TCI Chemical, Fisher Scientific, or BLD Pharm and were used without further purification. Reactions were monitored by thin layer chromatography (TLC) and an LC-MS Agilent 1260 II Infinity system equipped with a mass detector (column: InfinityLab Poroshell 120 EC-C18, 2.1 × 150, 2.7 μm). Appropriate gradient systems were applied by mixing water + 0.1% trifluoroacetic acid (TFA) and acetonitrile (+ 0.1% TFA). Analytical thin-layer chromatography was carried out using Merck silica gel aluminum plates with F-254 indicator, visualized under UV light (at 254 nm), iodine stain, or dipping in potassium permanganate stain (1.5 g of $KMnO_4$, 10 g of K_2CO_3, 1.25 mL of 10% aqueous NaOH solution, and 200 mL of water). The final products were purified by column chromatography over silica gel (Merck 60 particle size 0.040−0.063 mm) and reverse phase chromatography using acetonitrile and water. All solvents for chromatography were laboratory grade. The ^{1}H and ^{13}C NMR spectra were recorded on a Bruker DRX400 (400 MHz), DRX500 (500 MHz), DRX600 (600 MHz), and DRX700 (700 MHz) spectrometers in $CDCl_3$, DMSO-d_6 and CD_3OD. Data are reported in the following order: chemical shift in ppm; multiplicities are indicated s (singlet), d (doublet), t (triplet), q (quartet), hep (heptet), dd (doublet of doublet), dt (doublet of triplets), td (triplet of doublets), tt (triplet of triplets) and m (multiplet). Coupling constants (*J*) are given in Hertz (Hz). High-resolution mass spectra were recorded on an LTQ Orbitrap mass spectrometer coupled to an Accela HPLC System (HPLC column: Hypersyl GOLD, 50 mm × 1 mm, 1.9 μm) and on a compact QTOF (Bruker Daltonics GmbH & Co. KG, Bremen, Germany) coupled to an Agilent 1260 Infinity II system (Agilent Technologies, Waldbronn, Germany) consisting of a G7129A autosampler, a G7116A column oven, a G7117C photodiode array detector and a G7111B quaternary pump system by ESI or APCI ionization method. Compounds cms01-11, IA02, 04, 72, and indomethacin were obtained from COMAS (Compound Management und Screening Center at Max Planck Institute of Molecular Physiology, Dortmund).[1] All other compounds were synthesized based on the described procedures in the following pages. Chemical yields refer to the isolated pure substances.

SYNTHETIC PROCEDURES AND COMPOUND CHARACTERIZATION

Synthetic route A to obtain compounds IA03, 34, 36 64, 66, 76, 77, 81, 84, 89, 92, 93, 96:

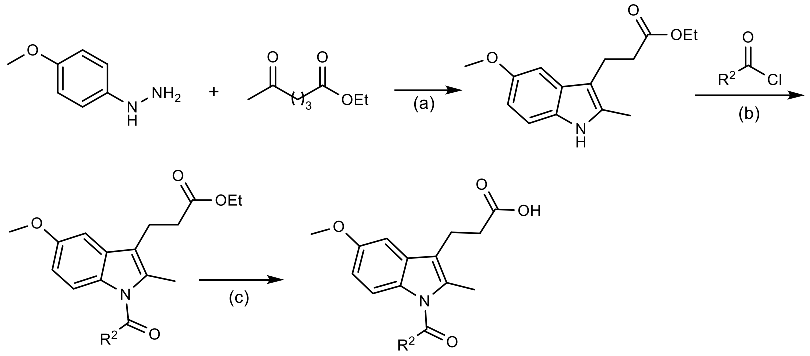

Supplementary Fig. 9. Synthetic route A. *(*a) i. Glacial acetic acid, sodium acetate, reflux, 3h; ii. Ethanol, 4M HCl in 1,4-dioxane, reflux, 15 h, (b) NaH, DMF, Ar, 0 °C – rt, 12 h, (c) Trimethyltin hydroxide, DCE, 80 °C

i. Ethyl 3-(5-methoxy-2-methyl-1*H*-indol-3-yl)propanoate (IA50)

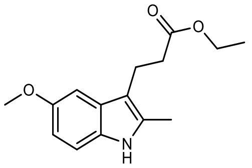

To a stirred mixture of 4-methoxyphenyl hydrazine hydrochloride (1.5 g, 8.59 mmol) and ethyl 4-acetylbutyrate (1.35 g, 8.59 mmol), sodium acetate (704.64 mg, 8.59 mmol) in glacial acetic acid (12 ml) was added. The resulting reaction mixture was heated to reflux for 3 h, and evaporated under vacuum to give a crude residue, which was dissolved in ethanol (7 ml) and treated with 4M HCl in 1,4-dioxane (5 ml) and refluxed for another 15 h. The mixture was then concentrated *in vacuo* to give the residue that was taken up in ethyl acetate and washed sequentially with water, aqueous potassium carbonate solution, and brine. The combined organic layers were dried over anhydrous magnesium sulphate, concentrated and purified by flash chromatography using ethyl acetate/petroleum ether (11% v/v) to yield ethyl 3-(5-methoxy-2-methyl-1*H*-indol-3-yl)propanoate (1.35 g, 60% yield) as a pale brown oil. ^{1}H NMR (400 MHz, $CDCl_3$) δ = 7.67 (s, 1H), 7.14 (d, 1H, *J* = 8.8 Hz), 6.96-6.95 (m, 1H), 6.78-6.75 (m, 1H), 4.15-4.10 (m, 2H), 3.86 (s, 3H), 3.00 (t, 2H, *J* = 7.6 Hz), 2.60 (t, 2H, *J* = 7.6 Hz), 2.37 (s, 3H), 1.25-1.21 (m, 3H). Synthesized by using modified literature procedure.[2]

ii. General procedure for *N*-acylated 3-(5-methoxy-2-methyl-1*H*-indol-3-yl) propanoic acid

Ethyl 3-(5-methoxy-2-methyl-1*H*-indol-3-yl)propanoate (1 equiv, 130 mg, 0.50 mmol) was added to a round-bottom flask with 2 mL *N, N*-dimethylformamide (DMF). The reaction mixture was maintained under an argon atmosphere at -20˚C. Then sodium hydride (NaH) (60%, 1.5 equiv, 0.75 mmol, 30 mg) was added carefully to the reaction mixture, which was allowed to stir for 15 min, followed by the addition of acid chloride (2 equiv). The resulting mixture was

allowed to gradually warm to room temperature and stirred overnight. Once the reaction is completed as monitored by TLC or LCMS, the mixture was carefully quenched using methanol and extracted using ethyl acetate. The organic layers were combined, dried over anhydrous magnesium sulphate, and evaporated *in vacuo* to obtain the *N*-acylated ethyl 3-(5-methoxy-2-methyl-1*H*-indol-3-yl)propanoate that was dissolved in 1,2-dichloroethane (DCE), to which was added trimethyltinhydroxide (2-4 equiv). The resulting reaction mixture was refluxed until complete conversion as monitored by TLC or LC-MS. The mixture was evaporated *in vacuo* and the residue was taken up into ethyl acetate and the organic layer was washed with 5% HCl 3 times and again with 5 ml brine and dried over anhydrous magnesium sulphate, concentrated and purified by flash chromatography using appropriate solvent gradients as mentioned under each final compound.

Synthetic route B to obtain 104, 107, 110, 137, 141, 147, 148:

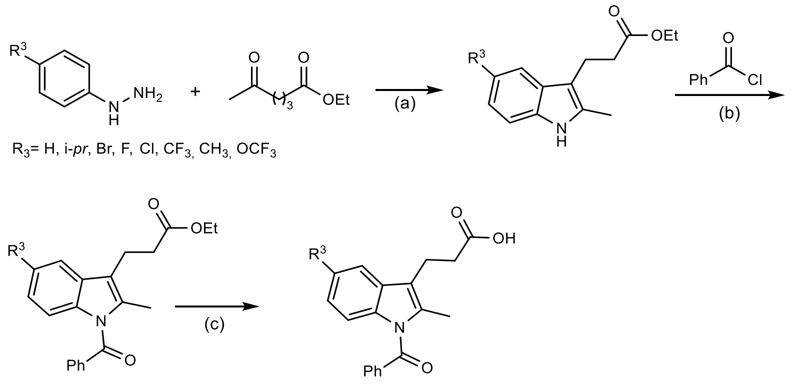

Supplementary Fig. 10. Synthetic route B. (a) H_2SO_4, Ethanol, reflux, 16-24 h; (b) NaH, DMF, Ar, 0 °C – rt, 12 h, (c) Trimethyltin hydroxide, DCE, 80 °C

To a mixture of 4 substituted phenylhydrazine hydrochloride of choice (1 equiv) and ethyl 4-acetylbutyrate (1 equiv) in ethanol (1.5 mL/mmol), conc. H_2SO_4 (aq) (120 µL/mmol) was added and stirred at reflux for 16-24 h. The reaction mixture was allowed to cool to room temperature, water (20 mL) was added and then aqueous layer was extracted with ethyl acetate (3 × 10 mL). The combined organic phases were washed with 10% HCl (10 mL) and then with saturated sodium bicarbonate (10 mL), dried over anhydrous magnesium sulphate and concentrated under reduced pressure. Purified by flash chromatography using ethyl acetate: petroleum ether (5-10% v/v) to yield the corresponding indole.

Subsequently, the intermediate was dissolved in *N*, *N*-dimethylformamide (DMF). The reaction mixture was maintained under an argon atmosphere at -20˚C. Then sodium hydride (60%, 1.5 equiv) was added carefully to the reaction mixture, which was allowed to stir for 15 min, followed by the addition of acid chloride (2 equiv). The resulting mixture was allowed to warm to room temperature and stirred overnight. Once the reaction is completed as monitored by TLC or LCMS, the mixture was carefully quenched using methanol and extracted using ethyl acetate. The organic layers were combined, dried over anhydrous magnesium sulphate, and evaporated *in vacuo* to obtain the corresponding indole ester that was dissolved in 1,2-dichloroethane (DCE), to which was added trimethyltinhydroxide (2-4 equiv). The resulting reaction mixture was refluxed until complete conversion as monitored by TLC or LC-MS.

The final reaction mixture was evaporated *in vacuo* and the residue was taken up into ethyl acetate and washed organic layer with 5% HCl for 3 times and again with 5 ml brine and dried over anhydrous magnesium sulphate, concentrated and purified using flash chromatography using appropriate solvent gradients as mentioned under each final compound.

Synthetic route C to obtain IA 138, 140, 142, 143, 145, 146, 149, 152, 153, 158, 159, 162, 163:

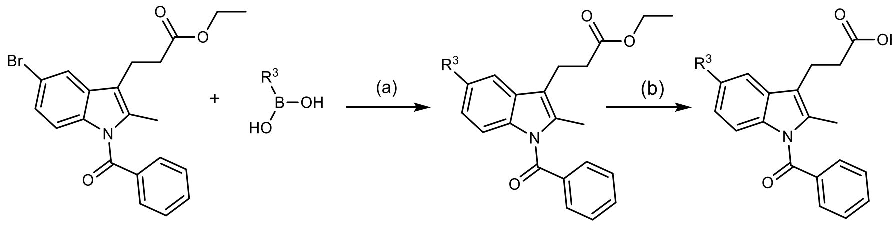

Supplementary Fig. 11. Synthetic route C. (a)Pd(dppf)Cl_2 (5.0 mol%), K_3PO_4, 1,4-dioxane and water (4:1), Ar, 80°C, 14 h (b) Trimethyltin hydroxide, DCE, 80 °C

i. Ethyl 3-(5-bromo-2-methyl-1*H*-indol-3-yl) propanoate (IA99)

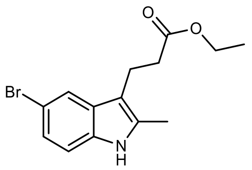

To a stirred mixture of 4-bromophenylhydrazine hydrochloride (1 equiv, 1000 mg, 4.47 mmol) and ethyl 4-acetylbutyrate (707. 814 mg, 1 equiv, 4.47 mmol, 715.69 µL) in ethanol (7 mL), conc. H_2SO_4 (aq) (0.675 mL) was added and stirred at reflux for 16 h. The reaction mixture was allowed to cool to room temperature, water (20 mL) was added, and then aqueous layer was extracted with EA (3 × 10 mL). The combined organic phases were washed with 10% HCl (10 mL) and then with saturated sodium bicarbonate (10 mL), dried over anhydrous magnesium sulphate and concentrated under reduced pressure. Purification by flash column chromatography on silica gel using ethyl acetate/petroleum ether (8% v/v) to get ethyl 3-(5-bromo-2-methyl-1*H*-indol-3-yl)propanoate as a pale brown oil (723.30 mg, 52 % yield).; ^{1}H NMR (600 MHz, $CDCl_3$) δ= 7.81 (s, 1H), 7.60 (d, 1H, *J* = 1.8 Hz), 7.18 (dd, 1H, *J*= 8.40, *J*= *J*= 8.4, 1.8 Hz), 7.11 (d, 1H, *J*= 8.4), 4.11 (q, 2H, *J* = 7.2 Hz), 2.98 (t, 2H, *J* = 7.7 Hz), 2.59 (t, 2H, *J* = 7.8 Hz), 2.37 (s, 3H) 1.23 (t, 3H, *J* = 7.2 Hz). ^{13}C NMR (151 MHz, $CDCl_3$) δ= 173.41, 133.98, 132.93, 130.28, 123.83, 120.60, 112.60, 111.74, 110.24, 60.56, 35.24, 19.70, 14.33, 11.77. Prepared using modified literature procedure.[3]

ii. Ethyl 3-(1-benzoyl-5-bromo-2-methyl-1*H*-indol-3-yl) propanoate (IA105/IAPD1)

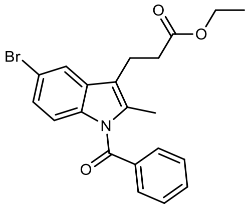

Ethyl 3-(5-bromo-2-methyl-1*H*-indol-3-yl)propanoate (1 equiv, 853.00 mg, 2.75 mmol) was added into a round-bottom flask with 5 mL *N, N*-dimethylformamide (DMF). The reaction mixture was maintained under an argon atmosphere at -20˚C. Then sodium hydride (NaH) (60%, 1.5 equiv, 4.12 mmol, 164.98 mg) was carefully added to the reaction mixture and stirred for 15 minutes, followed by the addition of benzoyl chloride (2 equiv). The reaction mixture was allowed to gradually warm to room temperature and stirred overnight. Once the reaction is completed as monitored by TLC or LCMS, the mixture was carefully quenched using methanol and extracted using ethyl acetate. The organic layers were combined, dried over anhydrous magnesium sulphate, and evaporated *in vacuo* to obtain the crude product which was purified by flash column using ethyl acetate/ petroleum ether (6% v/v) to get ethyl 3-(1-benzoyl-5-bromo-2-methyl-1*H*-indol-3-yl)propanoate (865.86 mg, 76% yield). A bright yellow oil; ^{1}H NMR (700 MHz, $CDCl_3$) δ = 7.69-7.67 (m, 2H), 7.65-7.63 (m, 1H), 7.59 (d, 1H, *J* = 1.4 Hz), 7.51-7.46 (m, 2H), 7.12 (dd, 1H, *J* = 9.1, 2.1 Hz), 6.87 (d, 1H, *J* = 8.8 Hz), 4.14 (q, 2H, *J* = 7.0 Hz), 3.00-2.98 (m, 2H), 2.61 (t, 2H, *J* = 7.7 Hz), 2.34 (s, 3H), 1.25 (t, 3H, *J* = 7.0 Hz). ^{13}C NMR (176 MHz, $CDCl_3$) δ 172.86, 169.60, 135.39, 135.37, 135.34, 133.28, 131.49, 129.89, 128.99, 125.80, 120.85, 117.14, 115.91, 115.73, 60.77, 34.42, 19.57, 14.35, 13.21. HRMS (ESI): m/z calculated for $C_{21}H_{21}BrNO_3$ $[M+H]^+$:414.0700, found: 414.0697.

iii. <u>General procedure for 5- substituted 3-(1-benzoyl-2-methyl-1*H*-indol-3-yl) propanoic acid</u>

A Schlenk tube was charged with IA105 (1 equiv), corresponding boronic acid (1.5 equiv), [1,1'-Bis(diphenylphosphino)ferrocene]dichloropalladium(II) ($Pd(dppf)Cl_2$) (5.0 mol%) and potassium phosphate tribasic (K_3PO_4) (2.0 equiv). A degassed mixture of 1,4-dioxane: water (4:1) was added to the Schlenk tube and heated to 80°C under an argon atmosphere. The reaction progress was monitored using both LC-MS and TLC. Upon completion, the reaction mixture was diluted with ethyl acetate and filtered through a celite plug. The celite plug was washed with ethyl acetate and filtrates were collected. The combined organic filtrate was then washed sequentially with water, followed by brine, and the organic layer was separated. The organic phase was dried over anhydrous magnesium sulfate, filtered, and concentrated *in vacuo*. The crude product was re-dissolved in a minimal amount of ethyl acetate and passed through a short silica plug for further purification. After evaporating the solvent, the intermediate product was used directly in the subsequent step without further purification.

Intermediate obtained after the general procedure (1 equiv) was dissolved in 1,2-dichloroethane (DCE), to which was added trimethyltinhydroxide (2-4 equiv). The resulting reaction mixture was refluxed until complete conversion as monitored by TLC or LC-MS. The resulting mixture was evaporated *in vacuo* and the residue was taken up into ethyl acetate and washed organic layer with 5% HCl for 3 times and again with 5 ml brine and dried over magnesium sulphate, concentrated, and purified using by flash chromatography using appropriate solvent gradients as mentioned under each final compound.

3-(1-(4-Chlorobenzoyl)-5-methoxy-2-methyl-1*H*-indol-3-yl)propanoic acid (IA01)

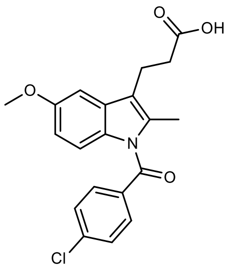

The aldehyde resin (1.0 equiv, 500 mg, 0.55 mmol) was dried in high vacuum overnight and suspended in 5 mL of dichloroethane (DCE). To this suspension, (4-methoxyphenyl) hydrazine hydrochloride (4.0 equiv, 384.19 mg, 2.22 mmol) and triethylamine (5 equiv, 162.55 mg, 2.75 mmol, 242.61 µL) were added under an argon atmosphere. The mixture was stirred at 45 °C overnight. After cooling to room temperature, the resin was filtered and washed three times each with 5 mL of *N*, *N*-dimethylformamide (DMF), 90/10(v/v) DMF/H_2O, DMF, dichloromethane, ethyl-acetate, and methanol.

The hydrazine resin (500 mg, 0.47 mmol) was dried in a high vacuum overnight and suspended in 5 mL of pyridine. To the mixture 4-chlorobenzoyl chloride ((12 equiv, 987.06 mg, 5.64 mmol, 723.12 µL) was added under argon. The mixture was shaken at 80°C overnight. After cooling the resin was filtered and washed three times with each 5 mL of *N*, *N*-dimethylformamide (DMF), 90/10(v/v) DMF/H_2O, DMF, dichloromethane, ethyl-acetate, and methanol.

The acylated hydrazine resin (150 mg, 0.13 mmol) was suspended in 6 mL of DCE/TFA (1/1). The 5-oxohexanoic acid (20 equiv, 327.20 mg, 1.26 mmol) was added, and the mixture was heated for 2 h at 70°C. After cooling the resin was filtered and washed three times with each 5 mL of dichloromethane, ethyl-acetate, and methanol. The filtrate was evaporated to dryness, and the crude product was purified by preparative HPLC using water and acetonitrile as the solvent system to obtain the title compound as a white solid (65 mg, 67% yield). ^{1}H NMR (DMSO-d_6, 500 MHz) δ= 12.17 (bs, 1H), 7.67-7.62 (m, 4H), 7.07 (d, 1H, *J* = 2.5 Hz), 6.94 (d, 1H, *J* = 9.0 Hz), 6.70 (dd, 1H, *J* = 9.0, 2.5 Hz), 3.78 (s, 3H), 2.89 (t, 2H, *J* = 7.5 Hz), 2.48 (m, 2H), 2.20 (s, 3H). The analytical data were consistent with those reported in the literature.[1]

3-(5-Methoxy-2-methyl-1-(thiophene-2-carbonyl)-1*H*-indol-3-yl)propanoic acid (IA03):

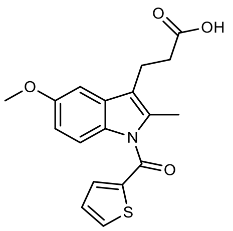

Synthesized by following synthetic route A with 0.29 mmol of ethyl 3-(5-methoxy-2-methyl-1-(thiophene-2-carbonyl)-1*H*-indol-3-yl)propanoate and 0.59 mmol of trimethyltinhydroxide. Purified by flash column using methanol/dichloromethane (5% v/v) to yield the pure product as a white powder (72 mg, 71.5 % yield); ^{1}H NMR ($CDCl_3$, 600 MHz) δ= 7.72 (1H, dd, *J* = 4.9, 0.9 Hz), 7.53 (1H, dd, *J* = 3.7, 0.9 Hz), 7.14-7.12 (m, 2H), 6.93 (1H, d, *J*

= 2.5 Hz), 6.70 (1H, dd, *J* = 9.0, 2.5 Hz), 3.85 (3H, s), 3.03 (2H, t, *J* = 7.7 Hz), 2.70 (2H, t, *J* = 7.7 Hz), 2.41 (3H, s). Data were consistent with those reported in the literature.[1]

3-(1-(4-(Fluorosulfonyl)benzoyl)-5-methoxy-2-methyl-1*H*-indol-3-yl)propanoic acid (IA06):

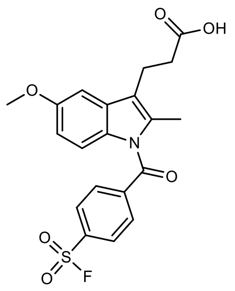

Synthesized by a procedure analogous to IA01. A yellow powder (10.26 mg, 18% yield); ^{1}H NMR ($CDCl_3$, 500 MHz), δ= 8.06 (d, 2H, *J*= 8.5 Hz), 7.83 (d, 2H, *J*= 8.5 Hz), 6.86 (d, 1H, *J*= 2.5 Hz), 6.83 (d, 1H, *J*= 9 Hz), 6.62 (dd, 1H, *J*= 9 Hz, *J*= 2.5 Hz), 3.77 (s, 3H), 2.93 (t, 2H, *J*= 7.5 Hz), 2.62 (t, 2H, *J*= 7.5 Hz), 2.23 (s, 3H). ^{13}C NMR (126 MHz, $CDCl_3$) δ= 177.06, 167.02, 156.63, 142.65, 136.26 (d, *J*= 25.2), 134.45, 131.04, 130.88, 130.64 (d, 2C, *J*= 39.06 Hz), 129.1 (s, 2C), 119.06, 115.36, 111.75, 101.83, 55.92, 33.47, 19.39, 13.83. ^{19}F NMR (470 MHz, CDCl3) δ= -75.82. HRMS (ESI): m/z calculated for $C_{20}H_{18}FNO_6SNa$ [M+Na] $^{+}$: 442.0732 found: 442.0732.

3-(1-(Benzo[b]thiophene-2-carbonyl)-5-methoxy-2-methyl-1*H*-indol-3-yl)propanoic acid (IA10)

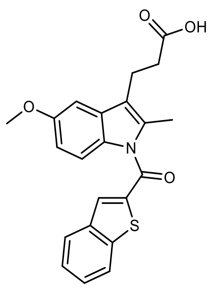

Synthesized by a procedure analogous to IA01. A yellow powder (4 mg, 8% yield); ^{1}H NMR ($CDCl_3$, 500 MHz), δ= 7.93 (d, 1H, *J*= 8.0 Hz), 7.83 (d, 1H, *J*= 8.0 Hz), 7.77 (s, 1H), 7.50 (m,1H), 7.43 (m,1H), 7.20 (d, 1H, *J*= 9.0 Hz), 6.95 (d, 1H, *J*= 2.5 Hz), 6.69 (dd, 1H, *J*= 9 Hz, *J*= 2.5 Hz), 3.85 (s, 3H), 3.05 (t, 2H, *J*= 7.5 Hz), 2.71 (t, 2H, *J*= 7.5 Hz), 2.45 (s, 3H). ^{13}C NMR (126 MHz, $CDCl_3$) δ 177.14, 163.60, 156.07, 142.58, 138.57, 138.15, 134.78, 131.73, 131.39, 130.50, 127.57, 126.02, 125.40, 123.04, 117.53, 114.95, 111.50, 101.14, 55.93, 33.71, 19.51, 12.98. HRMS (ESI): m/z calculated for $C_{22}H_{20}NO_4S$ [M+H]$^{+}$: 394.1108 found: 394.1105.

3-(1-(3-Cyanobenzoyl)-5-methoxy-2-methyl-1*H*-indol-3-yl)propanoic acid (IA30)

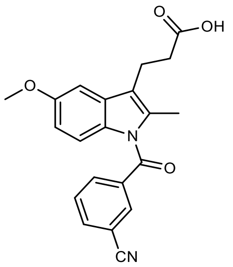

Synthesized by a procedure analogous to IA01. A yellow powder (11.9 mg, 12.1 % yield); ^{1}H NMR ($CDCl_3$, 500 MHz), δ= 7.97 (t, 1H, *J*= 1.5 Hz), 7.92-7.88 (m, 2H) 7.65-7.62 (m, 1H), 6.94 (d, 1H, *J*= 1.5 Hz), 6.85 (d, 1H, *J*= 9.0 Hz) 6.67 (dd, 1H, *J*= 9.0 Hz, J= 2.5 Hz), 3.85 (s, 3H), 3.01 (t, 2H, *J*= 7.5 Hz), 2.69 (t, 2H, *J*= 7.5 Hz), 2.33 (s, 3H). ^{13}C NMR (126 MHz, $CDCl_3$) δ 177.38, 167.18, 156.43, 137.27, 135.74, 134.58, 133.66, 133.15, 130.91, 129.91, 118.65, 117.72, 115.16, 113.49, 111.69, 101.64, 55.92, 33.59, 19.39, 13.61. HRMS (ESI): m/z calculated for $C_{21}H_{19}N_2O_4$ $[M+H]^+$: 363.1339 found: 363.1340.

3-(1-(9,10-Dioxo-9,10-dihydroanthracene-2-carbonyl)-5-methoxy-2-methyl-1*H*-indol-3-yl)propanoic acid (IA32)

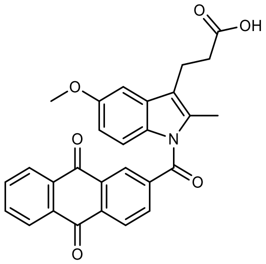

Synthesized by a procedure analogous to IA01. A yellow powder (7.0 mg, 8% yield); ^{1}H NMR ($CDCl_3$, 600 MHz) δ= 8.47-8.46 (m, 2H), 8.37-8.33 (m, 2H), 8.16-8.14 (m, 1H), 7.88-7.83 (m, 2H), 7.16 (d, 1H, *J*= 9.0 Hz), 6.95 (d, 1H, *J*= 2.4 Hz), 6.70 (dd, 1H, *J*= 9.0 Hz, *J*= 2.4 Hz), 3.85 (s, 3H), 3.03 (t, 2H, *J*= 7.5 Hz), 2.72 (t, 2H, *J*= 7.5 Hz), 2.26 (s, 3H). ^{13}C NMR (151 MHz, $CDCl_3$) δ= 182.67, 182.44, 174.83, 167.67, 156.54, 141.17, 135.76, 134.86, 134.75, 134.51, 134.08, 133.68, 133.57, 133.50, 131.20, 130.85, 128.53, 128.25, 127.79, 127.70, 119.04, 115.30, 111.84, 101.63, 55.93, 33.31, 19.76, 14.16. HRMS (ESI): m/z calculated for $C_{28}H_{22}NO_6$ $[M+H]^+$: 468.1442, found: 468.1444.

3-(1-(2-Naphthoyl)-5-methoxy-2-methyl-1*H*-indol-3-yl)propanoic acid (IA34)

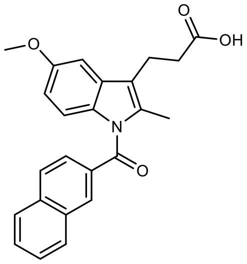

Synthesized by following synthetic route A with 0.15 mmol of ethyl 3-(1-(2-naphthoyl)-5-methoxy-2-methyl-1*H*-indol-3-yl)propanoate and 0.44 mmol of trimethyltin hyroxide. Purified by flash column using ethyl acetate/petroleum

ether (20% v/v) to yield pure product as a white powder (51.46 mg, 90% yield); ^{1}H NMR ($CDCl_3$, 500 MHz) δ= 8.24 (s, 1H), 7.95-7.90 (m, 3H), 7.75 (dd, 1H, *J*= 8.5 Hz, *J*= 1.7 Hz), 7.65-7.55 (m, 2H), 6.95 (d, 1H, *J* = 2.5 Hz), 6.91 (d, 1H, *J* = 9.0 Hz), 6.61 (dd, 1H, *J*= 9.0 Hz, J= 2.5 Hz), 3.84 (s, 3H), 3.05 (t, 2H, *J*= 7.7 Hz), 2.71 (t, 2H, *J*= 7.7 Hz), 2.38 (s, 3H). ^{13}C NMR ($CDCl_3$, 126 MHz) δ= 178.06, 169.69, 156.00, 135.43, 135.06, 133.07, 132.64, 131.41, 131.19, 130.57, 129.42, 128.78, 128.65, 128.08, 127.20, 125.67, 117.51, 115.33, 111.40, 101.15, 55.90, 33.90, 19.49, 13.41. HRMS (ESI): m/z calculated for $C_{24}H_{21}NO_4Na$ $[M+Na]^+$: 410.1363 found: 410.1356.

3-(5-Methoxy-2-methyl-1-(3-(trifluoromethyl)benzoyl)-1*H*-indol-3-yl)propanoic acid (IA36)

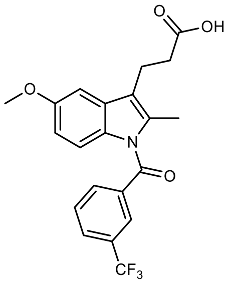

Synthesized by following synthetic route A with 0.05 mmol of ethyl 3-(5-methoxy-2-methyl-1-(3-(trifluoromethyl)benzoyl)-1*H*-indol-3-yl)propanoate and 0.20 mmol of trimethyltin hydroxide. Purified by flash column using ethyl acetate/petroleum ether (25% v/v) to yield the pure product as a white powder (18 mg, 87.4% yield); ^{1}H NMR ($CDCl_3$, 600 MHz), δ= 7.98 (s, 1H), 7.86 (pseudo t, 2H, *J* = 7.8 Hz), 7.63 (t, 1H, *J* = 7.8 Hz), 6.94 (d, 1H, *J* = 2.4 Hz), 6.91 (d, 1H, *J* = 9.0 Hz), 6.68 (dd, 1H, *J* = 9.0 Hz, *J* =3.0 Hz), 3.85 (s, 3H), 3.02 (t, 2H, *J*= 7.8 Hz), 2.69 (t, 2H, *J*= 7.8 Hz), 2.32 (s, 3H). ^{13}C NMR (151 MHz, $CDCl_3$) δ= 178.65, 168.02, 156.3, 136.74, 134.61, 132.93, 131.59 (q, *J* = 33.2 Hz), 131.06, 130.80, 129.51, 129.24 (q, *J* = 3.0 Hz), 126.63 (q, *J* = 3.7 Hz), 123.62 (q, *J* = 272.6 Hz), 118.34, 115.24, 111.63, 101.42, 55.89, 33.83, 19.39, 13.58. ^{19}F NMR (565 MHz, $CDCl_3$) δ= -62.79. HRMS (ESI): m/z calculated for $C_{21}H_{18}F_3NO_4Na$ $[M+Na]^+$: 428.1081, found: 428.1076.

3-(1-Benzoyl-5-methoxy-2-methyl-1*H*-indol-3-yl)propanoic acid (IA64)

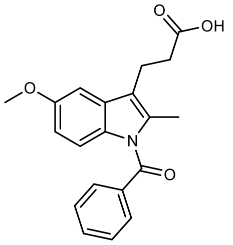

Synthesized by following synthetic route A with 0.33 mmol of ethyl 3-(1-benzoyl-5-methoxy-2-methyl-1*H*-indol-3-yl)propanoate and 0.99 mmol of trimethyltin hydroxide. Purified by flash column using methanol/dichloromethane (5% v/v) to yield the pure product as a white powder (109.68 mg, quant. yield); ^{1}H NMR ($CDCl_3$, 700 MHz), δ= 7.69-7.68 (m, 2H), 7.62-7.20 (m,1H), 7.47-7.49 (m, 2H), 6.93 (d, 1H, *J* = 2.8 Hz), 6.90 (d, 1H, *J* = 9.1 Hz), 6.65 (dd, 1H, *J*= 9.1 Hz, *J*= 2.8 Hz), 3.84 (s, 3H), 3.02 (t, 2H, *J*= 7.7 Hz), 2.68 (t, 2H, *J*= 7.7 Hz), 2.34 (s, 3H). ^{13}C NMR (176 MHz, $CDCl_3$) δ= 177.54, 169.68, 156.00, 135.94, 134.94, 132.83, 131.34, 130.57, 129.77, 128.87, 117.52, 115.34, 111.34,

101.11, 55.90, 33.77, 19.45, 13.39. HRMS (ESI): m/z calculated for $C_{20}H_{19}NO_4Na[M+Na]^+$: 360.1207, found: 360.1202.

3-(1-(4-Fluorobenzoyl)-5-methoxy-2-methyl-1*H*-indol-3-yl)propanoic acid (IA66)

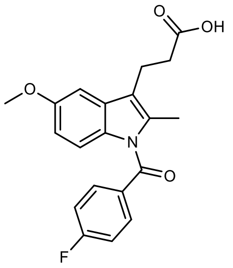

Synthesized by following synthetic route A with 0.25 mmol ethyl 3-(1-(4-fluorobenzoyl)-5-methoxy-2-methyl-1*H*-indol-3-yl)propanoate and 0.99 mmol trimethyltin hydroxide. Purified by flash column using ethyl acetate/petroleum ether (19 % v/v) to yield the pure product as a yellow powder (40 mg, 45.49 % yield); ^{1}H NMR ($CDCl_3$, 600 MHz) δ= 7.74-7.71 (m, 2H), 7.18-7.15 (m, 2H), 6.93 (d, 1H, *J* = 2.5 Hz), 6.87 (d, 1H, *J* = 9.0 Hz), 6.66 (dd, 1H, *J* = 9.0, 2.5 Hz), 3.84 (s, 3H), 3.02 (t, 2H, *J* = 7.7 Hz), 2.69 (t, 2H, *J* = 7.7 Hz), 2.36 (s, 3H). ^{13}C NMR (151 MHz, $CDCl_3$) δ= 178.02, 168.42, 165.61 (d, *J* = 254.8 Hz), 156.05, 134.89, 132.49 (d, *J* = 9.2 Hz), 131.97 (d, 2C, *J* = 3.2 Hz), 131.25, 130.57, 117.61, 116.14 (d, 2C, *J* = 22.1 Hz), 115.13, 111.41, 101.22, 55.90, 33.83, 19.43, 13.31. HRMS (ESI): m/z calculated for $C_{20}H_{19}FNO_4$ $[M+H]^+$: 356.1293, found: 356.1294.

3-(1-(4-(Dimethylamino)benzoyl)-5-methoxy-2-methyl-1*H*-indol-3-yl)propanoic acid (IA70)

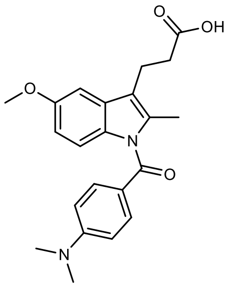

Synthesized by following the literature procedure.[1] A yellow crystalline powder (6 mg, 14% yield); ^{1}H NMR ($CDCl_3$, 700 MHz), δ= 7.63 (d, 2H, *J* = 8.9 Hz), 6.98 (d, 1H, *J* = 8.9 Hz), 6.93 (d, 1H, *J* = 2.4 Hz), 6.67-6.65 (m, 3H), 3.84 (s, 3H), 3.08 (s, 6H), 3.03 (t, 2H, *J* = 7.8 Hz), 2.69 (t, 2H, *J* = 7.8 Hz), 2.39 (s, 3H). ^{13}C NMR (176 MHz, $CDCl_3$) δ= 178.27, 169.29, 155.40, 153.73, 135.08, 132.85, 131.74, 129.92, 121.51, 115.83, 114.79, 111.07, 110.89, 100.63, 55.92, 40.20, 34.16, 19.57, 12.81. HRMS (ESI): m/z calculated for $C_{22}H_{25}N_2O_4$ $[M+H]^+$: 381.1809, found: 381.1805.

3-(1-([1,1'-Biphenyl]-4-carbonyl)-5-methoxy-2-methyl-1*H*-indol-3-yl)propanoic acid (IA75)

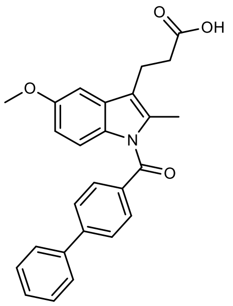

Synthesized by following the literature procedure.[1] A yellow powder (6 mg, 14% yield); ^{1}H NMR ($CDCl_3$, 700 MHz), δ= 7.78 (d, 2H, *J* = 8.4 Hz), 7.71 (d, 2H, *J* = 8.4), 7.66 (d, 2H, *J* = 7.7 Hz), 7.49 (t, 2H, *J* = 7.7 Hz), 7.42 (m, 1H), 6.98 (d, 1H, *J* = 9.1 Hz), 6.94 (d, 1H, *J* = 2.1 Hz), 6.67 (dd, 1H, *J* = 9.1, 2.1 Hz), 3.85 (s, 3H), 3.03 (t, 2H, *J* = 7.7 Hz), 2.70 (t, 2H, *J* = 7.7 Hz), 2.39 (s, 3H). ^{13}C NMR (176 MHz, $CDCl_3$) δ= 177.48, 169.40, 155.99, 145.69, 139.83, 134.98, 134.45, 131.35, 130.55, 130.52, 129.17, 128.50, 127.45, 127.43, 117.44, 115.31, 111.35, 101.13, 55.91, 33.79, 19.48, 13.39. The analytical data were consistent with those reported in the literature.[1]

3-(1-(Dimethylcarbamoyl)-5-methoxy-2-methyl-1*H*-indol-3-yl)propanoic acid (IA76)

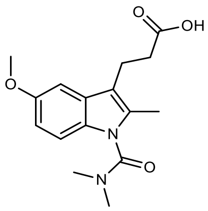

Synthesized by following synthetic route A with 0.15 mmol of ethyl 3-(1-(dimethylcarbamoyl)-5-methoxy-2-methyl-1*H*-indol-3-yl)propanoate and 0.60 mmol of trimethyltin hydroxide. Purified by flash chromatography using methanol/dichloromethane (50 % v/v) to yield the pure product as a colorless oil (40 mg, 87% yield); ^{1}H NMR ($CDCl_3$,500 MHz) δ= 7.09 (d, 1H, *J* = 9 Hz), 6.94 (d, 1H, *J* = 2.0 Hz), 6.82 (dd, 1H, *J* = 9, *J* = 2.5 Hz), 3.85 (s, 3H), 3.00 (m, 6H), 3.00-2.98 (2H, m), 2.64 (t, 2H, *J*= 8 Hz), 2.38 (s, 3H). ^{13}C NMR ($CDCl_3$, 126 MHz) δ= 178.88, 155.02, 154.77, 133.39, 129.95, 129.11, 113.54, 111.90, 111.52, 100.97, 55.98, 37.95, 37.89, 34.48, 19.53, 11.05. HRMS (ESI): m/z calculated for $C_{16}H_{21}N_2O_4$ $[M+H]^+$: 305.1496 found: 305.1498.

3-(5-Methoxy-2-methyl-1-(morpholine-4-carbonyl)-1*H*-indol-3-yl)propanoic acid (IA77)

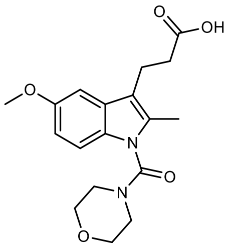

Synthesized by following synthetic route A with 0.36 mmol of ethyl 3-(5-methoxy-2-methyl-1-(morpholine-4-carbonyl)-1*H*-indol-3-yl)propanoate and 1.44 mmol of trimethyltin hydroxide. Purified by flash chromatography

using methanol/dichloromethane (4 % v/v) to yield the pure product as a white powder (56 mg, 45% yield); ^{1}H NMR ($CDCl_3$,600 MHz) δ= 7.19 (d, 1H, *J* = 9 Hz), 6.94 (d, 1H, *J* = 2.4 Hz), 6.83 (dd, 1H, *J* = 9, *J* =2.4 Hz), 3.85 (s, 3H), 3.77-3.68 (m, 4H), 3.51 (bs, 4H), 2.99 (t, 2H, *J*= 7.7 Hz), 2.64 (t, 2H, *J*= 7.7 Hz), 2.34 (s, 3H). ^{13}C NMR (151 MHz, $CDCl_3$) δ= 178.69, 155.23, 153.65, 133.43, 129.77, 129.22, 114.03, 111.70, 111.63, 101.13, 66.94, 55.95, 46.54, 34.37, 19.49, 11.20. HRMS (ESI): m/z calculated for $C_{18}H_{23}N_2O_5$ [M+H]$^+$: 347.1602 found: 347.1603.

3-(5-Methoxy-2-methyl-1-(tetrahydro-2*H*-pyran-4-carbonyl)-1*H*-indol-3-yl)propanoic acid (IA81)

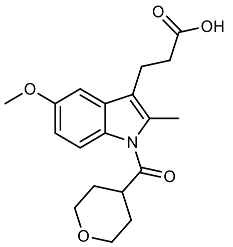

Synthesized by following synthetic route A with 0.30 mmol of ethyl 3-(5-methoxy-2-methyl-1-(tetrahydro-2H-pyran-4-carbonyl)-1*H*-indol-3-yl)propanoate and 0.87 mmol of trimethyltin hydroxide. Purified by flash chromatography using methanol/dichloromethane (2 % v/v) to yield pure product as a white powder (44 mg, 43% yield); ^{1}H NMR ($CDCl_3$, 500 MHz) δ= 7.66 (d, 1H, *J* = 9 Hz), 6.96 (d, 1H, *J* = 2.5 Hz), 6.86 (dd, 1H, *J* = 9, *J* =2.5 Hz), 4.07 (dt, 2H, *J* = 11.5, *J* = 3.5 Hz), 3.87 (s, 3H), 3.55 (td, 2H, *J* = 11.5, *J* = 2.0 Hz), 3.49-3.45 (m, 1H), 2.98 (t, 2H, *J*= 8.0 Hz), 2.63 (t, 2H, *J*= 7.5 Hz), 2.55 (s, 3H), 2.07-1.98 (m, 2H), 1.90-1.87 (m, 2H). ^{13}C NMR (126 MHz, $CDCl_3$) δ= 178.53, 175.35, 156.12, 134.34, 131.06, 130.09, 117.90, 115.16, 111.90, 101.52, 67.17, 67.10, 55.89, 42.65, 33.96, 29.35, 28.51, 19.38, 14.24. HRMS (ESI): m/z calculated for $C_{19}H_{23}NO_5Na$ [M+Na]$^+$: 368.1469 found: 368.1486.

3-(1-(Cyclopropanecarbonyl)-5-methoxy-2-methyl-1*H*-indol-3-yl)propanoic acid (IA84)

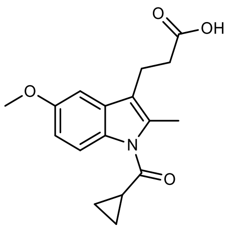

Synthesized by following synthetic route A with 0.28 mmol of ethyl 3-(1-(cyclopropanecarbonyl)-5-methoxy-2-methyl-1*H*-indol-3-yl)propanoate and 1.14 mmol of trimethyltin hydroxide. Purified by flash chromatography using ethyl acetate/petroleum ether (34 % v/v) to yield the pure product as a white powder (77.39 mg, 90% yield); ^{1}H NMR ($CDCl_3$, 700 MHz) δ= 7.85 (d, 1H, *J* = 9 Hz), 6.94 (d, 1H, *J* = 2.5 Hz), 6.84 (dd, 1H, *J* = 9.0, *J* = 2.5 Hz), 3.87 (s, 3H), 3.00 (t, 2H, *J*= 7.7 Hz), 2.65 (t, 2H, *J*= 7.7Hz), 2.56 (s, 3H), 2.35 (tt, 1H, *J* = 7.9, 4.6 Hz), 1.40 – 1.37 (m, 2H), 1.16-1.32 (m, 2H). ^{13}C NMR (176 MHz, $CDCl_3$) δ 178.26, 174.20, 155.99, 134.36, 130.80, 130.41, 116.87, 115.25, 111.60, 101.25, 55.92, 33.99, 19.37, 17.78, 13.73, 10.89. HRMS (ESI): m/z calculated for $C_{17}H_{19}NO_4$ [M+Na]$^+$: 324.1206 found: 324.1208.

3-(5-Methoxy-2-methyl-1-(piperidine-1-carbonyl)-1*H*-indol-3-yl)propanoic acid (IA89)

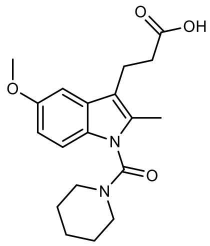

Synthesized by following synthetic route A with 0.26 mmol of ethyl 3-(5-methoxy-2-methyl-1-(piperidine-1-carbonyl)-1*H*-indol-3-yl)propanoate and 1.03 mmol of trimethyltin hydroxide. Purified by flash chromatography using ethyl acetate/petroleum ether (35 % v/v) to yield the pure product as a colorless gummy solid (26 mg, 29% yield); ^{1}H NMR ($CDCl_3$, 500 MHz) δ= 7.16 (d, 1H, J = 8.5 Hz), 6.94 (d, 1H, J = 2.0 Hz), 6.81 (dd, 1H, J = 8.5, J = 2.0 Hz), 3.853 (s, 3H), 3.49-3.38 (m, 4H), 3.00 (t, 2H, J= 7.7 Hz), 2.65 (t, 2H, J= 7.7Hz), 2.39 (s, 3H), 1.68-1.56 (s, 6H). ^{13}C NMR (126 MHz, $CDCl_3$) δ 178.74, 155.03, 153.47, 133.43, 130.11, 129.00, 113.35, 111.77, 111.44, 100.87, 55.99, 48.08, 47.26, 34.48, 26.31, 25.90, 24.47, 19.57, 11.10. HRMS (ESI): m/z calculated for $C_{19}H_{25}N_2O_4$ $[M+H]^+$: 345.1809 found: 345.1802.

3-(5-Mthoxy-2-methyl-1-(4,7,7-trimethyl-3-oxo-2-oxabicyclo[2.2.1]heptane-1-carbonyl)-1*H*-indol-3-yl)propanoic acid (IA92)

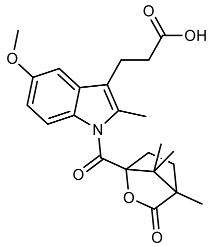

Synthesized by following synthetic route A with 0.18 mmol of ethyl 3-(5-methoxy-2-methyl-1-(4,7,7-trimethyl-3-oxo-2-oxabicyclo[2.2.1]heptane-1-carbonyl)-1*H*-indol-3-yl)propanoate and 0.72 mmol of trimethyltin hydroxide. Purified by flash chromatography using methanol/dichloromethane (7 % v/v) to yield the pure product as a brown powder (40 mg, 53% yield); ^{1}H NMR (700 MHz, $CDCl_3$) δ= δ 7.55 (d, 1H, J = 9.0 Hz), 6.89 (d, 1H, J = 2.5 Hz), 6.82 (dd, 1H, J = 9.0, 2.5 Hz), 3.85 (s, 3H), 2.98 (t, 2H, J = 7.7 Hz), 2.65 (m, 2H), 2.61-2.58 and 2.10-2.06 (2m, 2H), 2.43-2.39 and 1.86-1.82 (m, 2H), 2.41 (s, 3H), 1.29 (s, 3H), 1.12 (s, 3H), 0.86 (s, 3H). ^{13}C NMR (176 MHz, $CDCl_3$) δ = 178.09, 176.45, 172.08, 156.12, 134.12, 130.67, 130.44, 117.76, 114.67, 111.72, 101.25, 93.30, 77.34, 77.16, 76.98, 58.99, 55.95, 54.81, 33.49, 31.25, 29.85, 19.65, 17.84, 16.69, 13.70, 9.87. HRMS (ESI): m/z calculated for $C_{23}H_{27}NO_6Na$ $[M+Na]^+$: 436.1731 found: 436.1727.

3-(1-Isonicotinoyl-5-methoxy-2-methyl-1*H*-indol-3-yl)propanoic acid (IA93)

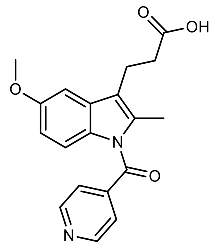

Synthesized by following synthetic route A with 0.26 mmol ethyl 3-(1-isonicotinoyl-5-methoxy-2-methyl-1*H*-indol-3-yl)propanoate and 1.06 mmol of trimethyltin hydroxide. Purified by flash chromatography using methanol/dichloromethane (2 % v/v) to yield the pure product as a yellow powder (30.45 mg, 34% yield); ^{1}H NMR ($CDCl_3$, 600 MHz) δ=8.81-8.80 (m, 2H), 7.54-7.53 (m, 2H), 7.02 (d, 1H, *J* = 9.0 Hz), 6.94 (d, 1H, *J* = 3 Hz), 6.69 (dd, 1H, *J* = 9.0, *J* = 3 Hz), 3.84 (s, 3H), 3.00 (t, 2H, *J* = 7.6 Hz), 2.67 (t, 2H, *J* = 7.6 Hz), 2.29 (s, 3H). ^{13}C NMR (151 MHz, $CDCl_3$) δ= 177.20, 167.34, 156.57, 150.50, 143.80, 134.29, 131.06, 130.80, 122.89, 119.11, 115.53, 111.69, 101.67, 55.90, 33.71, 19.47, 13.89. HRMS (ESI): m/z calculated for $C_{19}H_{19}N_2O_4$ $[M+H]^+$: 339.1340 found: 339.1354.

3-(5-Methoxy-1-(4-methoxybenzoyl)-2-methyl-1*H*-indol-3-yl)propanoic acid (IA96)

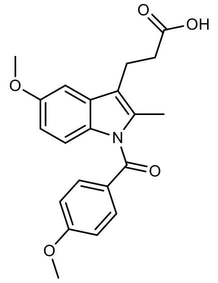

Synthesized by following synthetic route A with 0.34 mmol of ethyl 3-(5-methoxy-1-(4-methoxybenzoyl)-2-methyl-1*H*-indol-3-yl)propanoate and 0.67 mmol of trimethyltin hydroxide. Purified by column chromatography using ethyl acetate/petroleum ether (40% v/v) to yield the pure product as an off-white powder (119.85 mg, 97% yield); ^{1}H NMR ($CDCl_3$, 500 MHz) δ= 7.70-7.68 (m, 2H), 6.97-6.96(m, 2H), 6.93 (d, 1H, *J*= 2.5), 6.91 (d, 1H, *J*= 9.0), 6.65 (dd, 1H, *J* = 9.0, 2.5 Hz), 3.90 (s, 3H), 3.84 (s, 3H), 3.02 (t, 2H, *J* = 7.5 Hz), 2.69 (m, 2H), 2.37 (s, 3H). ^{13}C NMR (126 MHz, $CDCl_3$) δ 178.07, 169.07, 163.61, 155.77, 135.02, 132.44, 131.48, 130.31, 127.81, 116.87, 115.04, 114.12, 111.26, 100.95, 55.91, 55.69, 33.95, 19.48, 13.11. HRMS (ESI): m/z calculated for $C_{21}H_{22}NO_5$ $[M+H]^+$: 368.1493 found: 368.1493.

3-(1-Benzoyl-2-methyl-1*H*-indol-3-yl)propanoic acid (IA104)

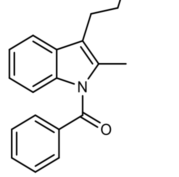

Synthesized by following synthetic route B with 0.30 mmol of ethyl 3-(1-benzoyl-2-methyl-1*H*-indol-3-yl)propanoate and 0.83 mmol of trimethyltinhydroxide. Purified by flash chromatography using ethyl acetate/petroleum ether (25% v/v) to yield the pure product as a white powder (90.72 mg, 99% yield); ^{1}H NMR ($CDCl_3$, 500 MHz) δ= 7.72-7.70 (m, 2H), 7.64-7.61 (m, 1H), 7.49 (m, 3H), 7.19-7.16 (m, 1H), 7.06-6.99 (m, 2H) 3.08-3.04 (m, 2H), 2.72-2.69(m, 2H), 2.37 (m, 3H). ^{13}C NMR (126 MHz, $CDCl_3$) δ= 178.13, 169.91, 136.67, 135.82, 134.14, 133.03, 129.92, 129.57, 128.91, 123.15, 122.61, 118.00, 117.44, 114.44, 33.99, 19.46, 13.19. HRMS (ESI): m/z calculated for $C_{19}H_{18}NO_3$ $[M+H]^+$: 308.1281 found:. 308.1281.

3-(1-Benzoyl-5-bromo-2-methyl-1*H*-indol-3-yl)propanoic acid (IA107)

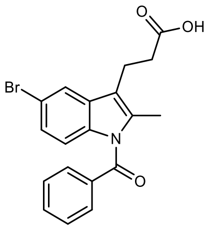

Synthesized by following synthetic route B with 0.20 mmol of ethyl 3-(1-benzoyl-5-bromo-2-methyl-1*H*-indol-3-yl)propanoate and 0.40 mmol of trimethyltin hydroxide. Purified by flash chromatography using ethyl acetate/petroleum ether (22% v/v) to yield the pure product as a white powder (76.61 mg, 99% yield); ^{1}H NMR ($CDCl_3$, 500 MHz) δ = 7.69-7.67 (m, 2H), 7.65-7.62 (m, 1H), 7.60 (d, 1H, *J* = 2.0 Hz), 7.51-7.48 (m, 2H), 7.13 (dd, 1H, *J* = 9, *J* = 2.0 Hz,), 6.87 (d, 1H, *J* = 9.0 Hz), 3.01 (t, 2H, *J* = 7.5 Hz), 2.68 (t, 2H, *J* = 7.5 Hz), 2.35 (m, 3H). ^{13}C NMR (126 MHz, $CDCl_3$) δ 177.98, 169.60, 135.55, 135.36, 135.33, 133.34, 131.34, 129.92, 129.01, 125.90, 120.75, 116.70, 115.97, 115.77, 33.91, 19.32, 13.23. HRMS (ESI): m/z calculated for $C_{19}H_{18}NO_3Br$ $[M+H]^+$: 386.03863 found:. 386.03879, HRMS (ESI): m/z calculated for $C_{19}H_{18}NO_3{}^{81}Br$ $[M+H]^+$: 388.0366 found: 388.0366.

3-(1-Benzoyl-5-isopropyl-2-methyl-1*H*-indol-3-yl)propanoic acid (IA110)

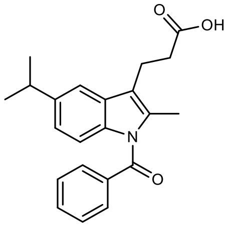

Synthesized by following synthetic route B with 0.13 mmol of ethyl 3-(1-benzoyl-5-isopropyl-2-methyl-1*H*-indol-3-yl)propanoate and 0.26 mmol of trimethyltin hydroxide. Purified by flash chromatography using ethyl acetate/petroleum ether (16% v/v) to yield the pure product as a white powder (40 mg, 87% yield); ^{1}H NMR ($CDCl_3$, 600 MHz) δ= 7.71-7.70 (m, 2H), 7.63-7.60 (m, 1H), 7.50-7.47 (m, 2H), 7.29 (m, 1H), 6.93-6.88 (m, 2H) 3.06 (t, 2H, *J* = 7.6 Hz), 2.97 (hep, 1H, *J* =6.9) 2.71 (t, 2H, *J* = 7.8 Hz), 2.36 (s, 3H), 1.28 (d, 6H, *J* = 6.6 Hz). ^{13}C NMR (151 MHz, $CDCl_3$) δ= 178.21, 169.84, 143.57, 136.01, 135.11, 134.26, 132.82, 129.81, 129.74, 128.86, 121.96, 117.62,

115.23, 114.32, 34.23, 34.01, 24.58, 19.42, 13.27. HRMS (ESI): m/z calculated for $C_{22}H_{24}NO_3$ $[M+H]^+$: 350.1751 found: 350.1751.

3-(1-Benzoyl-5-fluoro-2-methyl-1*H*-indol-3-yl)propanoic acid (IA137)

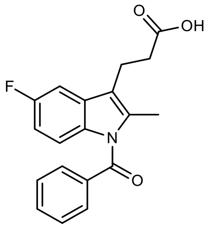

Synthesized by following synthetic route B with 0.09 mmol of ethyl 3-(1-benzoyl-5-fluoro-2-methyl-1*H*-indol-3-yl)propanoate and 0.28 mmol of trimethyltin hydroxide. Purified by flash chromatography using ethyl acetate/petroleum ether (17% v/v) to yield the pure product as a brown powder (21 mg, 67% yield); ^{1}H NMR ($CDCl_3$, 500 MHz) δ= 7.70-7.68 (m, 2H), 7.65-7.62 (m, 1H), 7.51-7.48 (m, 2H), 7.13 (dd, 1H, *J*=9.0, *J*= 2.5), 6.98 (q, 1H, *J*= 4.5), 6.77 (1H, td, *J* = 9.0, *J*= 2.5 Hz), 3.02-2.99 (m, 2H), 2.69-2.67 (m, 2H), 2.33 (s, 3H). ^{13}C NMR (126 MHz, $CDCl_3$,) δ= 177.75 (d, *J* = 71.7 Hz), 169.70 (s), 159.36 (d, *J* = 239.4 Hz), 135.72 (d, *J* = 35.7 Hz), 133.28 (d, *J*=44.1), 133.15 (s), 131.80 (s), 130.61 (d, *J* = 9.3 Hz), 129.85 (s), 128.97 (s), 117.34 (d, *J* = 3.8 Hz), 115.35 (d, *J* = 9.1 Hz), 110.80 (d, *J* = 25.2 Hz), 109.22 (d, *J* = 26.1 Hz), 103.70 (d, *J* = 23.9 Hz), 103.06 (d, *J* = 23.5 Hz), 34.16 (d, *J* = 103.4 Hz), 19.53 (d, *J* = 23.6 Hz), 12.64 (d, *J* = 190.9 Hz). ^{19}F NMR (470 MHz, $CDCl_3$) δ -120.73 (td, *J* = 8.9, 4.4 Hz), -124.94 (td, *J* = 9.5, 4.3 Hz). HRMS (ESI): m/z calculated for $C_{19}H_{16}NO_3FNa$ $[M+Na]^+$: 348.1007 found: 348.1004.

3-(1-Benzoyl-5-(furan-3-yl)-2-methyl-1*H*-indol-3-yl)propanoic acid (IA138)

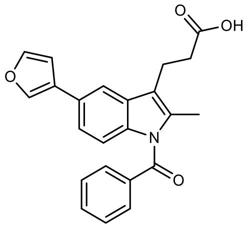

Synthesized by following synthetic route C with 0.08 mmol of ethyl 3-(1-benzoyl-5-(furan-3-yl)-2-methyl-1*H*-indol-3-yl)propanoate and 0.32 mmol of trimethyltin hydroxide. Purified by flash chromatography using ethyl acetate/petroleum ether (25% v/v) to yield the pure product as a yellow powder (16 mg, 54%); ^{1}H NMR ($CDCl_3$, 700 MHz) δ= 7.72-7.71 (m, 3H), 7.64-7.62 (m, 1H), 7.55 (d, 1H, *J*= 1.4 Hz), 7.50 (t, 2H, *J* = 7.7 Hz), 7.47 (t, 1H, *J* = 1.4 Hz), 7.17 (dd, 1H, *J* = 8.4, *J*= 1.4 Hz), 6.96 (d, 1H, *J* = 8.4 Hz), 6.72 (m, 1H), 3.07 (t, 2H, *J* = 7.7 Hz), 2.71 (t, 2H, *J* = 7.7 Hz), 2.37 (s, 3H). ^{13}C NMR (176 MHz, $CDCl_3$) δ 177.56, 169.77, 143.72, 138.30, 135.81, 135.73, 134.86, 133.08, 130.11, 129.91, 128.94, 127.16, 126.87, 121.48, 117.53, 115.13, 114.77, 109.30, 33.98, 19.42, 13.25. HRMS (ESI): m/z calculated for $C_{23}H_{20}NO_4$ $[M+H]^+$: 374.1387 found: 374.1386.

3-(1-Benzoyl-2-methyl-5-(thiophen-2-yl)-1*H*-indol-3-yl)propanoic acid (IA140)

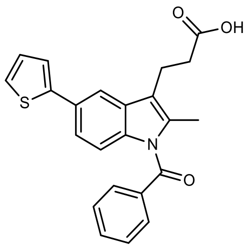

Synthesized by following synthetic route C with 0.07 mmol of ethyl 3-(1-benzoyl-2-methyl-5-(thiophen-2-yl)-1*H*-indol-3-yl)propanoate and 0.29 mmol of trimethyltin hydroxide. Purified by flash chromatography using ethyl acetate/petroleum ether (27% v/v) to yield the pure product as a white powder (20 mg, 73%); ^{1}H NMR ($CDCl_3$, 700 MHz) δ= 7.73-7.71 (m, 2H), 7.67 (d, 1H, *J* =2.1), 7.65-7.62 (m, 1H), 7.51-7.49 (m, 2H), 7.30 (dd, 1H, *J* = 8.6, *J* =1.8 Hz,), 7.28 (dd, 1H, *J* = 3.6, *J* = 1.1 Hz), 7.25 (dd, 1H, *J* = 5.1, *J* =1.1 Hz), 7.07 (dd, 1H, *J* = 5.1, *J* = 3.6 Hz), 6.96 (d, 1H, *J* = 8.6 Hz), 3.08 (t, 2H, *J* = 7.7 Hz), 2.72 (t, 2H, 7.7 Hz), 2.38 (s, 3H). ^{13}C NMR (176 MHz, $CDCl_3$) δ 178.25, 169.72, 145.07, 136.10, 135.63, 135.15, 133.14, 130.11, 129.93, 129.32, 128.96, 128.11, 124.46, 122.89, 121.73, 117.58, 115.34, 114.76, 34.07, 19.38, 13.26. HRMS (ESI): m/z calculated for $C_{23}H_{20}NO_3$ $[M+H]^+$: 390.1159 found: 390.1144.

3-(1-Benzoyl-5-chloro-2-methyl-1*H*-indol-3-yl)propanoic acid (IA141)

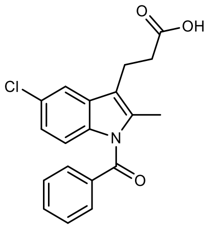

Synthesized by following synthetic route B with 0.22 mmol of ethyl 3-(1-benzoyl-5-chloro-2-methyl-1*H*-indol-3-yl)propanoate and 0.43 mmol of trimethyltin hydroxide. Purified by column chromatography using ethyl acetate/petroleum ether (15% v/v) to yield the pure product as colorless crystals (59 mg, 80% yield); ^{1}H NMR ($CDCl_3$, 500 MHz) δ= 7.69-7.66 (m, 2H), 7.65-7.62 (m, 1H), 7.51-7.48 (m, 2H), 7.44 (d, 1H, *J*= 2 Hz), 6.99 (dd, 1H, *J*= 8.8, *J*=2.0), 6.92 (d, 1H, *J*=8.5), 3.01 (t, 2H, *J* = 7.7 Hz), 2.68 (t, 2H, *J* = 7.7 Hz), 2.35 (s, 3H). ^{13}C NMR (126 MHz, $CDCl_3$) δ= 177.52, 169.62, 135.67, 135.38, 135.00, 133.30, 130.84, 129.91, 129.01, 128.34, 123.23, 117.71, 116.84, 115.38, 33.83, 19.35, 13.27. HRMS (ESI): m/z calculated for $C_{19}H_{17}NO_3Cl$ $[M+H]^+$: 342.08915 found: 342.08902; m/z calculated for $C_{19}H_{17}NO_3\,^{37}Cl$ $[M+H]^+$: 344.0862 found: 344.0860.

3-(1-Benzoyl-5-(3,5-dimethylisoxazol-4-yl)-2-methyl-1*H*-indol-3-yl)propanoic acid (IA142)

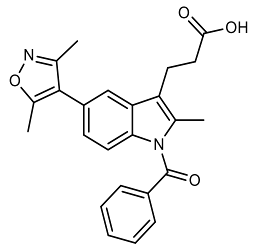

Synthesized by following synthetic route C with 0.04 mmol of ethyl 3-(1-benzoyl-5-(3,5-dimethylisoxazol-4-yl)-2-methyl-1*H*-indol-3-yl)propanoate and 0.14 mmol of trimethyltin hydroxide. Purified by preparative HPLC using acetonitrile and water as eluent to yield the pure product as a white powder (10 mg, 71% yield); ^{1}H NMR ($CDCl_3$, 500 MHz) δ=7.75-7.73 (m, 2H), 7.67-7.64 (m, 1H), 7.52 (t, 2H, *J* = 7.5 Hz), 7.33 (d, 1H, *J* = 1.0 Hz), 7.08 (d, 1H, *J* = 8.5 Hz), 6.92 (dd, 1H, *J* = 8.5, *J* =1.5 Hz), 3.06 (t, 2H, *J* = 7.5 Hz), 2.70 (t, 2H, *J* = 7.7 Hz), 2.41 (s, 3H), 2.38 (s, 3H), 2.28 (s, 3H). ^{13}C NMR (126 MHz, $CDCl_3$) δ= 176.85, 169.82, 165.35, 159.06, 135.98, 135.57, 135.19, 133.27, 130.02, 129.92, 129.03, 124.55, 124.29, 118.62, 117.33, 117.23, 114.69, 33.81, 19.35, 13.30, 11.69, 10.92. HRMS (ESI): m/z calculated for $C_{24}H_{23}N_2O_4$ $[M+H]^+$: 403.1652 found: 403.1650.

3-(1-Benzoyl-5-(2,4-dimethoxypyrimidin-5-yl)-2-methyl-1*H*-indol-3-yl)propanoic acid (IA143)

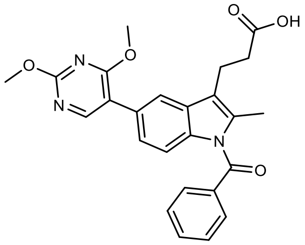

Synthesized by following synthetic route C with 0.10 mmol of ethyl 3-(1-benzoyl-5-(2,4-dimethoxypyrimidin-5-yl)-2-methyl-1*H*-indol-3-yl)propanoate and 0.31 mmol of trimethyltin hydroxide. Purified by flash column using methanol/dichloromethane (2 % v/v) to yield the pure product as a white powder (28,5 mg, 62% yield); ^{1}H NMR ($CDCl_3$, 700 MHz) δ= 8.30 (s, 1H), 7.73 (d, 2H, *J* = 7.7 Hz), 7.64 (t, 1H, *J* = 7.7 Hz), 7.57 (d, 1H, *J* = 1.4 Hz), 7.51 (t, 2H, *J* = 7.7 Hz), 7.16 (dd, 1H, *J* = 8.4, *J* =1.4 Hz), 7.02 (d, 1H, *J* = 8.4 Hz), 4.04 (s, 3H), 4.02 (s, 3H), 3.08 (t, 2H, *J* = 7.7 Hz), 2.70 (t, 2H, *J* = 7.7 Hz), 3.07 (s, 3H). ^{13}C NMR (176 MHz, $CDCl_3$) δ= 176.65, 169.78, 168.38, 164.39, 157.60, 136.13, 135.66, 134.93, 133.15, 129.93, 129.88, 128.98, 127.58, 124.08, 118.43, 117.68, 116.70, 114.31, 55.04, 54.35, 33.95, 19.49, 13.23. HRMS (ESI): m/z calculated for $C_{25}H_{24}N_3O_5$ $[M+H]^+$: 446.1711 found: 446.1709.

3-(1-Benzoyl-2-methyl-5-(4-(trifluoromethyl)phenyl)-1*H*-indol-3-yl)propanoic acid (IA145)

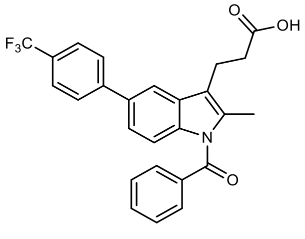

Synthesized by following synthetic route C with 0.10 mmol of ethyl 3-(1-benzoyl-2-methyl-5-(4-(trifluoromethyl)phenyl)-1*H*-indol-3-yl)propanoate and 0.42 mmol of trimethyltin hydroxide. Purified by preparative HPLC using acetonitrile and water as eluent to yield the pure product as a white powder (17 mg, 36% yield); ^{1}H NMR ($CDCl_3$, 500 MHz) δ= 7.84 (m, 1H), 7.78 (m, 1H), 7.74-7-73 (m 2H), 7.67-7.63 (m, 2H), 7.89-7.57 (m, 1H), 7.56-7.50 (m 3H), 7.28 (d, 1H, *J* = 1.5 Hz), 7.05 (d, 1H, *J* = 8.5 Hz), 3.11 (t, 2H, *J* = 7.7 Hz), 2.73 (t, 2H, *J* = 7.7 Hz), 2.40 (s, 3H). ^{13}C NMR (126 MHz, $CDCl_3$) δ= 176.93, 169.78, 142.54, 136.45, 135.59, 135.29, 134.58, 133.23, 131.38, 130.74, 130.24, 129.95, 129.32, 129.00, 123.93 (dd, *J* = 63.3, 3.8 Hz), 122.56, 117.61, 116.59, 114.85, 77.42, 77.16, 76.91, 33.87, 19.38, 13.27. ^{19}F NMR (470 MHz, $CDCl_3$) δ = -62.52. HRMS (ESI): m/z calculated for $C_{26}H_{21}NO_3F_3$ $[M+H]^+$: 452.1468 found: 452.1469.

3-(5-(3-Aminophenyl)-1-benzoyl-2-methyl-1*H*-indol-3-yl)propanoic acid (IA146)

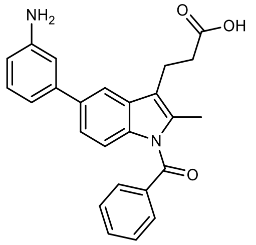

Synthesized by following synthetic route C with 0.05 mmol of ethyl 3-(5-(3-aminophenyl)-1-benzoyl-2-methyl-1*H*-indol-3-yl)propanoate and 0.14 mmol of trimethyltin hydroxide. Purified by preparative HPLC using acetonitrile and water as eluent to yield the pure product as a white powder (9 mg, 48% yield); ^{1}H NMR ($CDCl_3$, 700 MHz) δ= 8.10-8.09 (m, 1H), 7.69-7-67 (m, 2H), 7.64-7.62 (m, 2H), 7.60 (d, 1H, *J*= 1.4 Hz), 7.50-7.46 (m, 4H), 7.13 (m, 1H), 6.88 (d, *J* = 9.0 Hz, 1H), 3.02 (t, 2H, *J* = 7.7 Hz), 2.69 (t, 2H, *J* = 7.7 Hz), 2.34 (s, 3H). ^{13}C NMR (176 MHz, $CDCl_3$) δ= 178.61, 169.63, 135.52, 135.33, 133.32, 131.35, 130.32, 129.91, 129.00, 128.62, 125.89, 123.93, 120.75, 116.76, 115.97, 115.77, 113.92, 112.69, 111.80, 109.85, 34.06, 19.34, 13.24. HRMS (ESI): m/z calculated for $C_{25}H_{23}N_2O_3$ $[M+H]^+$: 399.1703 found: 399.1696.

3-(1-Benzoyl-2,5-dimethyl-1*H*-indol-3-yl)propanoic acid (IA147)

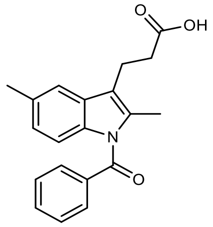

Synthesized by following synthetic route B with 0.24 mmol of ethyl 3-(1-benzoyl-2-methyl-5-(*p*-tolyl)-1*H*-indol-3-yl)propanoate and 0.73 mmol of trimethyltin hydroxide. Purified by flash column chromatography using ethyl acetate/petroleum ether (17 % v/v) to yield the pure product as a pale brown powder (77.39 mg, 99% yield); ^{1}H NMR ($CDCl_3$, 500 MHz) δ= 7.71-7.69 (m, 2H), 7.63-7.60 (m, 1H), 7.48 (m, 2H), 7.26 (m, 1H), 6.85 (m, 2H), 3.03 (t, 2H, *J*

= 7.8 Hz), 2.70 (t, 2H, *J* = 7.8 Hz), 2.41 (s, 3H), 2.36 (s, 3H). ^{13}C NMR (126 MHz, $CDCl_3$) δ= 179.02, 169.84, 135.93, 134.89, 134.22, 132.85, 132.18, 129.82, 129.81, 128.84, 124.40, 117.96, 117.31, 114.19, 34.14, 21.44, 19.46, 13.23. HRMS (ESI): m/z calculated for $C_{20}H_{20}N_3$ $[M+H]^+$: 322.1438 found: 322.1433.

3-(1-Benzoyl-2-methyl-5-(trifluoromethoxy)-1*H*-indol-3-yl)propanoic acid (IA 148)

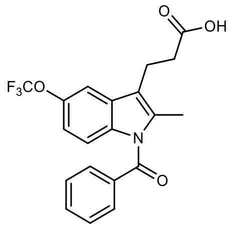

Synthesized by following synthetic route B with 0.27 mmol of ethyl 3-(1-benzoyl-2-methyl-5-(4-(trifluoromethoxy)phenyl)-1*H*-indol-3-yl)propanoate and 0.82 mmol of trimethyltin hydroxide. Purified by flash column chromatography using ethyl acetate/petroleum ether (20 % v/v) to yield the pure product as an off-white powder (50 mg, 46% yield); ^{1}H NMR ($CDCl_3$, 700 MHz) δ= 7.70-7.69 (m, 2H), 7.65-7-63 (m, 1H), 7.52-7.49 (m, 2H), 7.32 (m, 1H), 7.02 (d, 1H, *J* = 9.0 Hz), 6.98-6.91 (m, 1H), 3.03 (t, 2H, *J* = 7.7 Hz), 2.68 (t, 2H, *J* = 7.7 Hz), 2.35 (s, 3H). ^{13}C NMR (176 MHz, $CDCl_3$) δ= 178.79 (d, *J* = 98.5 Hz), 169.67, 144.95 (d, *J*= 4.7), 136.15, 135.06 (d, *J* = 87.5 Hz), 133.36, 130.34, 129.91, 129.05, 120.78 (q, *J* = 256.2 Hz), 115.05 (d, *J* = 11.4 Hz), 117.29, 116.48, 115.05 (d, J = 11.4 Hz), 110.72 (d, *J* = 34.2 Hz), 110.57 (d, *J* = 7.04 Hz), 34.38 (d, *J* = 143.4 Hz), 19.37 (d, *J* = 32.1 Hz), 12.58 (d, *J* = 262.5 Hz). ^{19}F NMR (470 MHz, $CDCl_3$) δ -58.02 (d, *J* = 26.0 Hz). HRMS (ESI): m/z calculated for $C_{20}H_{17}F_3NO_4$ $[M+H]^+$: 392.1105 found: 392.1095.

4-(1-Benzoyl-3-(2-carboxyethyl)-2-methyl-1*H*-indol-5-yl)benzoic acid (IA149)

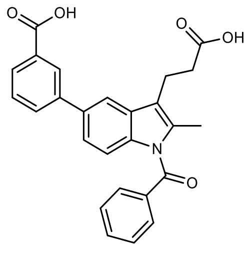

Synthesized by following synthetic route C with 0.09 mmol of 3-(1-benzoyl-3-(3-ethoxy-3-oxopropyl)-2-methyl-1*H*-indol-5-yl)benzoic and 0.26 mmol of trimethyltin hydroxide. Purified by preparative HPLC using acetonitrile and water as eluent to yield the pure product as a white powder (9.9 mg, 26% yield); ^{1}H NMR ($CDCl_3$, 700 MHz) δ= 8.47 (m, 1H), 8.00 (m, 1H), 7.87 (m, 1H), 7.81 (d, 1H, *J* = 1.6 Hz), 7.76 (dd, 2H, *J*=8.2 Hz, *J*= 1.2 Hz) 7.67-7.65 (m, 1H), 7.54-7.51 (m, 3H), 7.35 (dd, 1H, *J* = 8.6 Hz, 1.8 Hz), 7.04 (d, 1H, *J* = 8.6 Hz), 3.16 (t, 2H, *J*= 8.1 Hz), 2.75-2.72 (t, 2H, *J*= 8.2 Hz), 2.42 (s, 3H). ^{13}C NMR (176 MHz, $CDCl_3$) δ 179.43, 172.16, 169.77, 141.41, 136.40, 135.63, 135.01, 134.10, 133.19, 132.33, 130.39, 129.95, 129.86, 129.11, 129.00, 128.63, 128.60, 122.01, 117.79, 116.30, 114.87, 34.59, 19.49, 13.24. HRMS (ESI): m/z calculated for $C_{26}H_{22}NO_5$ $[M+H]^+$: 428.1493 found: 428.1493.

3-(1-Benzoyl-5-(4-carbamoylphenyl)-2-methyl-1*H*-indol-3-yl)propanoic acid (IA152)

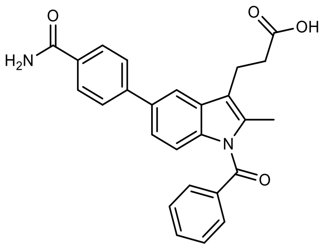

Synthesized by following synthetic route C with 0.08 mmol of ethyl 3-(1-benzoyl-5-(4-carbamoylphenyl)-2-methyl-1*H*-indol-3-yl)propanoate and 0.25 mmol of trimethyltin hydroxide. Purified by preparative HPLC using acetonitrile and water as eluent to yield the product as a white powder (30.3 mg, 79% yield, 94% purity); ^{1}H NMR (700 MHz, DMSO-d_6) δ= 12.14 (s, 1H), 8.01 (s, 1H), 7.96-7.95 (2H, m), 7.92 (m, 1H), 7.80 (d, 2H, *J* = 7.7 Hz), 7.75-7.72 (m, 1H), 7.70-7.68 (m, 2H), 7.61-7.59 (m, 2H), 7.44-7.43 (m, 1H), 7.36-7.30 (m, 1H), 7.04 (d, 1H, *J* = 9.1 Hz), 3.00 (t, 2H, *J* = 7.5 Hz), 2.57 (t, 2H, *J* = 7.5 Hz), 2.26 (s, 3H). ^{13}C NMR (176 MHz, DMSO-d_6) δ= 173.95, 169.13, 167.61, 143.25, 135.70, 135.25, 134.21, 133.73, 133.12, 132.53, 129.94, 129.37, 129.01, 128.07, 126.53, 126.19, 122.00, 118.15, 116.73, 114.09, 34.05, 18.97, 12.91. HRMS (ESI): m/z calculated for $C_{26}H_{23}N_2O_4$ $[M+H]^+$: 427.1652 found: 427.1653.

3-(1-Benzoyl-2-methyl-5-(pyridin-4-yl)-1*H*-indol-3-yl)propanoic acid (IA153)

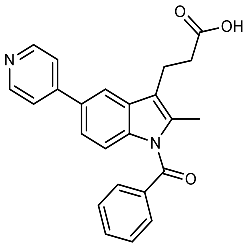

Synthesized by following synthetic route C with 0.05 mmol of ethyl 3-(1-benzoyl-2-methyl-5-(pyridin-4-yl)-1*H*-indol-3-yl)propanoate and 0.19 mmol of trimethyltin hydroxide. Purified by preparative HPLC using acetonitrile and water as eluent to yield the pure product as a white powder (5.03 mg, 27% yield); ^{1}H NMR (CD_3OD, 500 MHz) δ= 8.77-8.76 (m, 2H), 8.36 (dd, 2H, *J*= 5.5 Hz, *J*=1.5 Hz) 8.22 (d, 1H, *J*= 1.8), 7.74-7.72 (m, 3H), 7.66 (dd, 1H, *J* = 8.7 Hz, 2.0 Hz), 7.61-7.57 (m, 2H), 7.24 (d, 1H, *J*= 8.7 Hz), 3.16 (t, 2H, *J*= 7.3 Hz), 2.70 (t, 2H, *J*= 7.5 Hz), 2.37 (s, 3H). ^{13}C NMR (126 MHz, CD_3OD) δ= 176.68, 171.01, 143.48, 139.92, 137.30, 136.53, 134.63, 131.94, 130.89, 130.19, 130.13, 129.46, 124.91, 123.59, 119.59, 119.42, 116.10, 35.22, 20.36, 13.18. HRMS (ESI): m/z calculated for $C_{24}H_{21}N_2O_3$ $[M+H]^+$: 385.1553 found: 385.1547.

3-(1-Benzoyl-2-methyl-5-(1*H*-pyrazol-4-yl)-1*H*-indol-3-yl)propanoic acid (IA158)

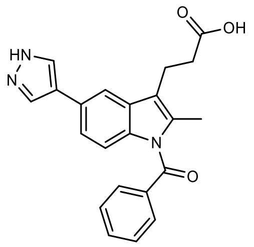

Synthesized by following synthetic route C with 0.05 mmol of ethyl 3-(1-benzoyl-2-methyl-5-(1*H*-pyrazol-4-yl)-1*H*-indol-3-yl)propanoate and 0.24 mmol of trimethyltin hydroxide. Purified by preparative HPLC using acetonitrile and water as eluent to yield the pure product as a white powder (2.5 mg, 14% yield); ^{1}H NMR ($CDCl_3$, 700 MHz) δ= 7.80 (s, 2H), (dd, *J* = 8.4, 1.4 Hz, 2H), 7.65 – 7.63 (m, 2H), 7.50 (t, *J* = 7.7 Hz, 2H), 7.11 (dd, *J* = 8.5, 1.6 Hz, 1H), 6.97 (d, *J* = 8.5 Hz, 1H), 3.14 (t, *J* = 7.2 Hz, 2H), 2.69 (t, *J* = 7.2 Hz, 2H), 2.39 (s, 3H). ^{13}C NMR (176 MHz, $CDCl_3$) δ= 176.80, 169.73, 135.76, 135.69, 135.00, 133.13, 130.53, 129.98, 129.89, 128.97, 125.71, 123.94, 121.09, 118.01, 115.45, 114.84, 34.92, 19.97, 13.24. HRMS (ESI): m/z calculated for $C_{22}H_{20}N_3O_3$ $[M+H]^+$: 374.1499 found:. 374.1499.

3-(1-Benzoyl-5-(2-cyanophenyl)-2-methyl-1*H*-indol-3-yl)propanoic acid (IA159)

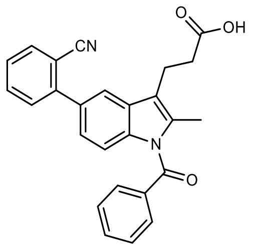

Synthesized by following synthetic route C with 0.07 mmol of ethyl 3-(1-benzoyl-5-(2-cyanophenyl)-2-methyl-1*H*-indol-3-yl)propanoate and 0.21 mmol of trimethyltin hydroxide. Purified by preparative HPLC using acetonitrile and water as eluent to yield the pure product as a white powder (9 mg, 32% yield); ^{1}H NMR ($CDCl_3$, 600 MHz) δ= 7.78-7.77 (m, 2H), 7.75 (d, *J* = 1.3 Hz, 1H), 7.70 (d, *J* = 1.8 Hz, 1H), 7.66-7.63 (m, 2H), 7.55-7.51 (m, 3H), 7.44-7.41 (m, 1H), 7.19 (dd, *J* = 8.6, 1.8 Hz, 1H), 7.10 (d, *J* = 8.5 Hz, 1H), 3.10 (t, *J* = 7.4 Hz, 2H), 2.73 (t, *J* = 7.4 Hz, 2H), 2.40 (s, 3H). ^{13}C NMR (151 MHz, $CDCl_3$) δ 174.70, 169.81, 146.23, 136.71, 135.56, 135.39, 133.89, 133.26, 132.99, 132.59, 130.46, 129.99, 129.91, 129.03, 127.35, 123.86, 119.40, 118.74, 117.78, 114.62, 111.42, 34.03, 19.78, 13.25. HRMS (ESI): m/z calculated for $C_{26}H_{21}N_2O_3$ $[M+H]^+$: 409.1547 found: 409.1547.

3-(5-(5-Acetylthiophen-2-yl)-1-benzoyl-2-methyl-1*H*-indol-3-yl)propanoic acid (IA162)

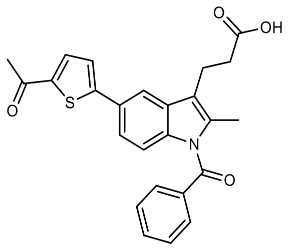

Synthesized by following synthetic route C with 0.05 mmol of ethyl 3-(5-(5-acetylthiophen-2-yl)-1-benzoyl-2-methyl-1*H*-indol-3-yl)propanoate and 0.21 mmol of trimethyltin hydroxide. Purified by preparative HPLC using acetonitrile and water as eluent to yield the pure product as a brown powder (10 mg, 43% yield), ^{1}H NMR (700 MHz, $CDCl_3$) δ= 7.74 (d, 1H, *J* = 1.6 Hz), 7.72 (dd, 2H, *J* = 8.3, 1.2 Hz), 7.67-7.64 (m, 2H), 7.53-7.50 (m, 2H), 7.33 (m, 1H), 7.30 (d, 1H, *J* = 3.5 Hz), 7.00 (d, 1H, *J* = 8.4 Hz), 3.09 (t, 2H, *J* = 7.7 Hz), 2.72 (t, 2H, *J* = 7.7 Hz), 2.56 (s, 3H), 2.39 (s, 3H). ^{13}C NMR (176 MHz, $CDCl_3$) δ= 190.72, 175.88, 169.64, 153.81, 142.70, 136.96, 135.68, 135.38, 133.74, 133.38, 130.19, 129.98, 129.04, 128.08, 123.62, 121.67, 117.53, 115.93, 114.90, 33.69, 26.67, 19.35, 13.24. HRMS (ESI): m/z calculated for $C_{25}H_{21}NO_4SNa$ $[M+Na]^+$: 454.1084 found: 454.1066.

3-(1-Benzoyl-5-(4-hydroxyphenyl)-2-methyl-1*H*-indol-3-yl)propanoic acid (IA163)

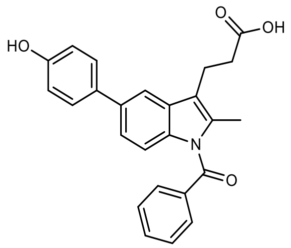

Synthesized by following synthetic route C with 0.11 mmol of ethyl 3-(1-benzoyl-5-(4-hydroxyphenyl)-2-methyl-1*H*-indol-3-yl)propanoate and 0.33 mmol of trimethyltin hydroxide. Purified by flash chromatography using methanol/dichloromethane (4% v/v) to yield the pure product as an off-white powder (34.39 mg, 80% yield); ^{1}H NMR (DMSO, 700 MHz) δ= 12.12 (s, 1H), 9.46 (s, 1H), 7.74-7.72 (m, 2H), 7.69 (d, 2H, *J*=7.7 Hz), 7.60 (t, 1H, *J* = 7.7 Hz), 7.52 (d, 1H, *J* = 9.1 Hz), 7.28 (dd, 1H, *J* = 9.1, *J* = 1.4 Hz), 6.99 (d, 1H, *J* = 8.4 Hz), 6.84 (d, 2H, *J* = 8.4 Hz), 2.98 (t, 2H, *J* = 7.7 Hz), 2.56 (t, 2H, *J* = 7.7 Hz), 2.26 (s, 3H). ^{13}C NMR (176 MHz, DMSO) δ= 173.90, 169.08, 156.70, 135.41, 134.97, 134.80, 133.78, 132.95, 131.42, 129.85, 129.27, 128.95, 127.86, 121.40, 118.10, 115.60, 115.51, 113.93, 34.00, 19.00, 12.91. HRMS (ESI): m/z calculated for $C_{25}H_{22}NO_4$ $[M+H]^+$: 400.1543 found: 400.1542.

3-(1-Benzyl-5-bromo-2-methyl-1H-indol-3-yl)propanoic acid (IA164)

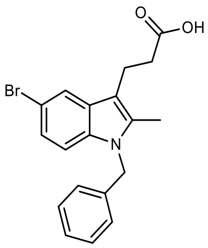

Ethyl 3-(5-bromo-2-methyl-1*H*-indol-3-yl)propanoate (IA99) (1 equiv, 150 mg, 0.48 mmol) was added into a round-bottom flask with 1 mL *N*, *N*-dimethylformamide (DMF). The reaction mixture was maintained under an argon atmosphere at -20˚C. Then sodium hydride (NaH) (60%, 1.5 equiv, 29.01 mg, 0.73 mmol) was carefully added to the reaction mixture and stirred for 15 minutes, followed by the addition of benzyl chloride (2 equiv, 122.43 mg, 0.967 mmol, 111.29 µL). The reaction mixture was allowed to gradually warm to room temperature and stirred overnight. Once the reaction was completed as monitored by TLC and LC-MS, the mixture was carefully quenched using methanol and extracted using ethyl acetate. The organic layers were combined, dried over anhydrous magnesium sulphate, and evaporated *in vacuo* to obtain the crude intermediate (1 equiv. 185 mg, 0.46 mmol) that was dissolved in 1 mL 1,2-dichloroethane (DCE), to which was added trimethyltinhydroxide (1.2 equiv, 100.28 mg, 0.56 mmol). The resulting reaction mixture was refluxed until complete conversion as monitored by TLC and LC-MS. The mixture was evaporated *in vacuo* and the residue was taken up into ethyl acetate and washed organic layer with 5% HCl for 3 times and again with 5 ml brine and dried over anhydrous magnesium sulphate, concentrated and purified by flash chromatography using methanol/dichloromethane (2% v/v) to yield the pure product as a white powder (143 mg, 83% yield); ^{1}H NMR ($CDCl_3$, 500 MHz), δ= 7.65 (d, 1H, *J* = 1.8 Hz), 7.27-7.23 (m, 2H), 7.22-7.21 (m, 1H), 7.17 (dd, 1H, *J* = 8.6, 1.8 Hz), 7.04 (d, 1H, *J* = 8.6 Hz), 6.90 – 6.87 (m, 2H), 5.25 (s, 2H), 3.04 (t, 2H, *J* = 7.6 Hz), 2.66 (t, 2H, *J* = 7.6 Hz), 2.29 (s,3H). ^{13}C NMR (126 MHz, $CDCl_3$) δ= 179.10, 137.57, 135.34, 134.81, 129.34, 128.98, 127.54, 125.93, 123.85, 120.57, 112.66, 110.73, 109.78, 46.80, 35.11, 19.87, 10.40. HRMS (ESI): m/z calculated for $C_{19}H_{19}BrNO_2$ $[M+H]^+$: 372.0594, found: 372.0568.

3-(5-Bromo-1-(cyclohexanecarbonyl)-2-methyl-1H-indol-3-yl)propanoic acid (IA165)

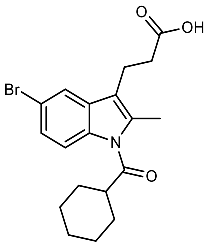

Ethyl 3-(5-bromo-2-methyl-1*H*-indol-3-yl)propanoate (IA99) (1 equiv, 150 mg, 0.48 mmol) was added into a round-bottom flask with 1 mL *N*, *N*-dimethylformamide (DMF). The reaction mixture was maintained under an argon atmosphere at -20˚C. Then sodium hydride (NaH) (60%, 1.5 equiv, 29.01 mg, 0.73 mmol) was carefully added to the reaction mixture and stirred for 15 minutes, followed by the addition of cyclohexyl carbonyl chloride (2 equiv, 141.8 mg, 0.97 mmol, 129.85 µL). The reaction mixture was allowed to gradually warm to room temperature and stirred

overnight. Once the reaction was completed as monitored by TLC and LC-MS, the mixture was carefully quenched using methanol and extracted using ethyl acetate. The organic layers were combined, dried over anhydrous magnesium sulphate, and evaporated *in vacuo* to obtain the crude intermediate (1 equiv. 141 mg, 0.34 mmol) that was dissolved in 1.5 mL 1,2-dichloroethane (DCE), to which was added trimethyltinhydroxide (2 equiv, 121.48 mg, 0.6718 mmol). The resulting reaction mixture was refluxed until complete conversion as monitored by TLC and LC-MS. The mixture was evaporated *in vacuo* and the residue was taken up into ethyl acetate and washed organic layer with 5% HCl for 3 times and again with 5 ml brine and dried over anhydrous magnesium sulphate, concentrated and purified by flash chromatography using methanol/dichloromethane (1% v/v) to yield the pure product as a white powder (120 mg, 91%); ^{1}H NMR ($CDCl_3$, 700 MHz), δ= 7.65 (d, 1H, *J* = 8.8 Hz), 7.58 (d, 1H, *J* = 2.0 Hz), 7.34 (dd, 1H, *J* = 8.8, 2.0 Hz), 3.14 (tt, 1H, *J* = 11.3, 3.3 Hz), 2.98 (t, 2H, *J* = 7.7 Hz), 2.63 (t, 2H, *J* = 7.7 Hz), 2.54 (s, 3H), 1.99-1.97 (m, 2H), 1.88-1.86 (m, 2H), 1.76-1.74 (m, 1H), 1.68-1.62 (m, 2H), 1.39-1.37 (m, 1H), 1.36 – 1.31 (m, 2H). ^{13}C NMR (176 MHz, $CDCl_3$) δ= 177.98, 177.47, 134.76, 134.45, 131.59, 126.48, 120.82, 116.75, 116.02, 115.84, 45.99, 33.96, 29.73, 25.81, 25.72, 19.29, 13.92. HRMS (ESI): m/z calculated for $C_{19}H_{23}BrNO_3$ $[M+H]^+$: 392.0856, found: 392.0848.

Compounds from extended data:

General procedure D:

Synthesized by reported resin-capture-release strategy.[1] Aldehyde resin (500 mg, 0.55 mmol, loading capacity: 0.9 mmol/g) was dried under high vacuum overnight and suspended in 5 mL of dichloroethane (DCE). Hydrazine hydrochloride (2.75 mmol, 5 equiv.) and triethylamine (194 µL, 6 equiv.) were added to the suspension under an argon atmosphere. The mixture was stirred at 45 °C overnight. After cooling, the resin was filtered and washed three times sequentially with 5 mL each of *N,N*-dimethylformamide (DMF), 90/10 (v/v) DMF/H_2O, DMF, dichloromethane, ethyl acetate, and methanol to yield hydrazone resin.

The hydrazone resin (500 mg) was dried under a high vacuum overnight and suspended in 5 mL of pyridine. Acid chloride (3 equiv) was added under an argon atmosphere, and the mixture was stirred at 80 °C overnight. After cooling, the resin was filtered and washed three times sequentially with *N,N*-dimethylformamide (DMF), 90/10 (v/v) DMF/H_2O, DMF, dichloromethane, ethyl acetate, and methanol.

The acylated hydrazone resin (150 mg) was then suspended in 6 mL of a DCE/TFA (1:1) mixture. The corresponding ketone (10 equiv) was added, and the reaction mixture was heated for 15 minutes to 2 hours at 70 °C. After cooling, the reaction was quenched with methanol, and the resin was filtered and washed sequentially with 5 mL of dichloromethane, methanol, ethyl acetate, and methanol. The filtrate was evaporated to dryness, and the crude product was purified using preparative HPLC with acetonitrile and water as eluents.

3-(1-(3-Cyanobenzoyl)-2-methyl-1*H*-indol-3-yl)propanoic acid (IA41)

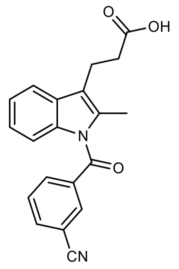

Synthesized by following the general procedure D to yield the pure product as a white powder (7 mg, 16% yield); ^{1}H NMR ($CDCl_3$, 700 MHz) δ= 8.00-7.99 (1H, m), 7.93 (dt, 1H, *J* = 7.7, 1.4 Hz), 7.90 (dt, 1H, *J* = 7.7, 1.4 Hz), 7.64 (t, 1H, *J* = 7.7 Hz), 7.50 (d, 1H, *J* = 7.7 Hz), 7.22-7.20 (m, 1H), 7.08-7.06 (m, 1H), 6.92 (d, 1H, *J* = 8.4 Hz), 3.06 (t, 2H, *J* = 7.7 Hz), 2.71 (t, 2H, *J* = 7.7 Hz), 2.37 (s, 3H). ^{13}C NMR (176 MHz, $CDCl_3$) δ= 176.83, 167.48, 137.11, 136.32, 135.93, 133.82, 133.78, 133.28, 129.95, 129.82, 123.66, 123.27, 118.58, 118.42, 117.70, 114.23, 113.55, 33.59, 19.40, 13.39. HRMS (APCI): m/z calculated for $C_{20}H_{17}N_2O_3$ $[M+H]^+$:333.1234, found: 333.1223.

3-(2-Methyl-1-(thiophene-2-carbonyl)-1*H*-indol-3-yl)propanoic acid (IA42)

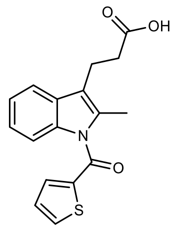

Synthesized by following the general procedure D to yield the pure product as a white powder (17.39 mg, 42% yield); ^{1}H NMR ($CDCl_3$, 700 MHz) δ= 7.74 (1H, dd, *J* = 4.9, 1.4 Hz), 7.54-7.53 (m, 1H), 7.49 (1H, d, *J* = 7.8 Hz), 7.24 (1H, d, *J* = 8.3 Hz), 7.20 – 7.17 (m, 1H), 7.13 (1H, dd, *J* = 4.9, 3.5 Hz), 7.10-7.08 (1H, m), 3.06 (t, 2H, *J* = 7.7 Hz), 2.71 (t, 2H, *J* = 7.7 Hz), 2.43 (3H, s). ^{13}C NMR (176 MHz, $CDCl_3$) δ= 177.13, 163.06, 138.72, 136.72, 135.11, 134.34, 133.91, 129.40, 127.98, 122.93, 122.42, 118.08, 117.05, 113.82, 33.86, 19.51, 12.67. HRMS (APCI): m/z calculated for $C_{17}H_{15}NO_3S$ $[M+H]^+$: 314.0846 , found: 314.0837.

3-(5-Bromo-2-methyl-1-(thiophene-2-carbonyl)-1*H*-indol-3-yl)propanoic acid (IA44)

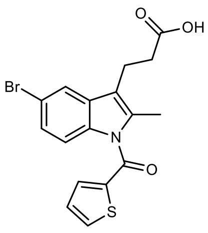

Synthesized by following the general procedure D to yield the pure product as a white powder (16 mg, 34 % yield); ^{1}H NMR ($CDCl_3$, 500 MHz) δ= 7.77 (1H, dd, *J* = 5.0, 1.0 Hz), 7.61 (1H, d, *J* = 1.5 Hz), 7.50 (1H, dd, *J* = 3.5, 1.0 Hz), 7.17 1H, (dd, *J* = 9.0, 2.0 Hz), 7.14 (1H, dd, *J* = 5.0, 3.5 Hz), 7.09 (1H, d, *J* = 9.0 Hz), 3.02 (2H, t, *J* = 7.8 Hz, 2H),

2.69 (2H, t, J = 7.8 Hz), 2.42 (s, 3H). ^{13}C NMR (126 MHz, $CDCl_3$) δ= 176.50, 162.72, 138.28, 135.38, 135.34, 134.87, 131.14, 128.12, 125.67, 120.82, 116.30, 115.73, 115.15, 33.70, 19.37, 12.69. HRMS (APCI): m/z calculated for $C_{17}H_{14}BrNO_3S$ $[M+H]^+$: 391.9951, found: 391.9947.

3-(1-(3-Cyanobenzoyl)-5-fluoro-2-methyl-1*H*-indol-3-yl)propanoic acid (IA47)

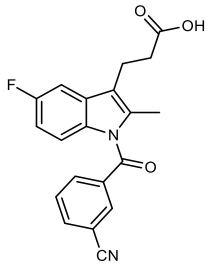

Synthesized by following the general procedure D to yield the pure product as a white powder (6.23 mg, 14% yield); 1H NMR ($CDCl_3$, 600 MHz) δ = 7.98 (s, 1H), 7.92-7.90 (m, 2H), 7.65 (t, 1H, J = 7.8 Hz), 7.15 (dd, 1H, J = 8.4, 2.4 Hz), 6.98 (dd, 1H, J = 9.0, 4.3 Hz), 6.83-6.80 (m, 1H), 3.00 (t, 2H, J = 7.5 Hz), 2.69 (t, 2H, J = 7.5 Hz), 2.31 (s, 3H). ^{13}C NMR (151 MHz, $CDCl_3$) δ 176.72, 167.27, 159.64 (d, J = 240.7 Hz), 136.91, 136.02, 135.41, 133.68, 133.20, 132.63, 130.95 (d, J = 9.3 Hz), 130.03, 118.48 (d, J = 3.8 Hz), 117.61, 115.24 (d, J = 9.2 Hz), 113.63, 111.35 (d, J = 25.2 Hz), 104.24 (d, J = 23.9 Hz), 33.44, 19.37, 13.67. HRMS (APCI): m/z calculated for $C_{20}H_{16}FN_2O_3$ $[M+H]^+$: 351.1140, found: 351.1122.

3-(5-Fluoro-2-methyl-1-(thiophene-2-carbonyl)-1*H*-indol-3-yl)propanoic acid (IA48)

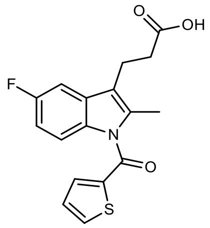

Synthesized by following the general procedure D to yield the pure product as a white powder (13 mg, 30% yield); ^{1}H NMR ($CDCl_3$, 600 MHz) δ= 7.75 (1H, d, J= 4.8 Hz), 7.52 (1H, d, J= 4.2 Hz), 7.18 (1H, dd, J= 9, 4.2 Hz), 7.144-7.125 (2H, m), 6.81 (1H, td, J = 9.0, 2.4 Hz, 1H) 3.02 (t, 2H, J = 7.8 Hz), 2.68 (t, 2H, J = 7.8 Hz), 2.41 (3H, s). ^{13}C NMR (151 MHz, $CDCl_3$) δ= 177.04, 162.83, 159.27 (d, J = 239.2 Hz), 138.43, 135.67, 135.14, 134.56, 133.0, 130.36 (d, J = 9.4 Hz), 128.04, 116.95 (d, J = 3.9 Hz), 114.69 (d, J = 9.2 Hz), 110.73 (d, J = 23.8 Hz), 103.70 (d, J = 23.8 Hz), 33.72, 19.47, 12.87. HRMS (APCI): m/z calculated for $C_{17}H_{14}FNO_3S$ $[M+H]^+$:332.0752, found:332.0740.

3-(5-Chloro-2-methyl-1-(thiophene-2-carbonyl)-1*H*-indol-3-yl)propanoic acid (IA60)

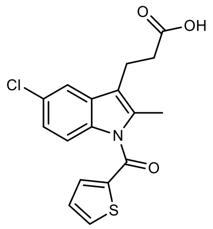

Synthesized by following the general procedure D to yield the pure product as a white powder (10.25, 22% yield); ^{1}H NMR ($CDCl_3$, 600 MHz) δ= 7.76 (dd, 1H, *J* = 4.9, 1.2 Hz), 7.51 (dd, 1H, *J* = 3.6, 1.2 Hz), 7.45 (d, 1H, *J* = 2.0 Hz), 7.15-7.13 (m, 2H), 7.04 (dd, 1H, *J* = 8.8, 2.1 Hz), 3.02 (t, 2H, *J* = 7.7 Hz), 2.69 (t, 2H, *J* = 7.7 Hz), 2.42 (s, 3H). ^{13}C NMR (151 MHz, $CDCl_3$) δ 177.04, 162.74, 138.30, 135.47, 135.30, 135.03, 134.81, 130.63, 128.12, 128.10, 123.01, 117.76, 116.43, 114.76, 33.79, 19.39, 12.74. HRMS (APCI): m/z calculated for $C_{17}H_{15}ClNO_3S$ $[M+H]^+$:348.0456, found: 348.0451.

3-(5-Chloro-1-(3-cyanobenzoyl)-2-methyl-1*H*-indol-3-yl)propanoic acid (IA63)

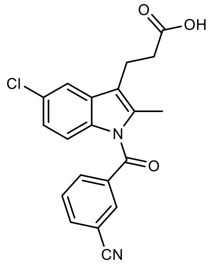

Synthesized by following the general procedure D to yield the pure product as a white powder (8.33 mg, 18% yield); ^{1}H NMR ($CDCl_3$, 700 MHz) δ = 7.98-7.97 (m, 1H), 7.92-7.90 (m, 2H), 7.65 (t, 1H, *J* = 7.7 Hz, 1H), 7.46 (d, 1H, *J* = 2.0 Hz), 7.04 (1H, dd, *J* = 9.1, 2.1 Hz), 6.92 (1H, d, *J* = 8.4 Hz), 3.01 (t, *J* = 7.6 Hz, 1H), 2.70 (t, *J* = 7.6 Hz, 1H), 2.33 (s, 3H). ^{13}C NMR (176 MHz, $CDCl_3$) δ 176.79, 167.24, 136.71, 136.15, 135.25, 134.68, 133.73, 133.25, 131.10, 130.05, 129.05, 123.78, 118.14, 117.97, 117.56, 115.21, 113.68, 33.51, 19.28, 13.54. HRMS (APCI): m/z calculated for $C_{20}H_{16}ClN2O_3$ $[M+H]^+$: 367.0844, found: 367.0849.

<u>Reference compounds used:</u>

Ethyl (*S*)-(6-methyl-5-((3-(2-(piperidin-3-ylamino)pyrimidin-4-yl)pyridin-2-yl)oxy)naphthalen-1-yl)carbamate (G-1749)

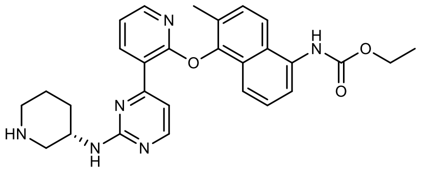

Synthesized by following the reported procedure.[4] An off-white powder (60% yield); ^{1}H NMR (DMSO-d_6, 600 MHz) δ= 9.57 (s, 1H), 8.48 (d, 1H, *J* = 5.4 Hz), 8.05 (dd, 1H, *J* = 4.8, 1.8 Hz), 7.93 (d, 1H, *J* = 9.0 Hz), 7.58 – 7.55 (m, 2H),

7.51 (d, 1H, *J* = 7.2 Hz), 7.49 (d, 1H, *J* = 9.0 Hz), 7.46 (d, 1H, *J* = 8.4 Hz), 7.41 – 7.37 (m, 1H), 7.26 (dd, 1H, *J* = 7.8, 4.8 Hz), 4.25 (s, 1H), 4.17 (q, *J* = 7.1 Hz, 2H), 3.46 (m, 1H), 3.23-3.21 (m, 1H), 2.89-2.84 (m, 2H), 2.21 (s, 3H), 2.03-1.91 (m, 2H), 1.75-1.61 (m, 2H), 1.28 (t, 3H, *J* = 7.2 Hz). ^{13}C NMR (151 MHz, DMSO-d_6) δ= 161.56, 160.39, 158.22, 157.99, 154.86, 148.74, 146.38, 140.21, 134.23, 128.70, 128.27, 127.89, 126.80, 126.28, 120.46, 120.18, 120.16, 119.02, 117.92, 110.60, 60.41, 46.59, 44.72, 43.14, 28.06, 20.74, 16.14, 14.62.

***(S)*-2-Chloro-*N*-(6-methyl-5-((3-(2-(piperidin-3-ylamino)pyrimidin-4-yl)pyridin-2-yl)oxy)naphthalen-1-yl)benzenesulfonamide (AMG-18)**

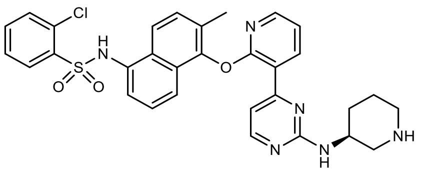

Synthesized by following the literature procedure.[5] An off-white powder (50% yield); ^{1}H NMR (DMSO-d_6, 600 MHz) δ= 10.63 (s, 1H), (d, 1H, *J* = 5.4 Hz), 8.06 (d, 1H, *J* = 8.4 Hz), 8.04 (dd, 1H, *J* = 4.8, 1.8 Hz), 7.85 (dd, 1H, *J* = 7.8, 1.2 Hz), 7.70 (dd, 1H, *J* = 8.4, 1.2 Hz), 7.64-7.61 (m, 1H), 7.54 (d, 1H, *J* = 4.2 Hz), 7.51-7.47 (m, 3H), 7.44 (td, 1H, *J* = 7.8, 1.2 Hz), 7.31-7.28 (m, 1H), 7.26 (dd, 1H, *J* = 7.8, 4.8 Hz), 7.15 (d, 1H, *J* = 7.2 Hz), 4.24 (s, 1H), 3.45-3.43 (m 1H), 3.23-3.20 (m, 1H), 2.91-2.81 (m 2H), 2.17 (s, 3H), 2.02-1.90 (m 2H), 1.74-1.59 (m, 2H). ^{13}C NMR (151 MHz, DMSO-d_6) = 161.54, 160.27, 158.19, 157.96, 148.72, 146.27, 140.23, 137.40, 134.53, 132.24, 131.97, 131.08, 130.89, 129.54, 129.21, 128.44, 127.74, 127.29, 126.19, 122.36, 120.44, 120.18, 119.96, 119.08, 110.63, 46.59, 44.71, 43.13, 28.04, 20.73, 16.12.

NMR SPECTRA

Supplementary Fig. 12. ^{1}H NMR of ethyl 3-(5-methoxy-2-methyl-1*H*-indol-3-yl)propanoate (400 MHz, $CDCl_3$)

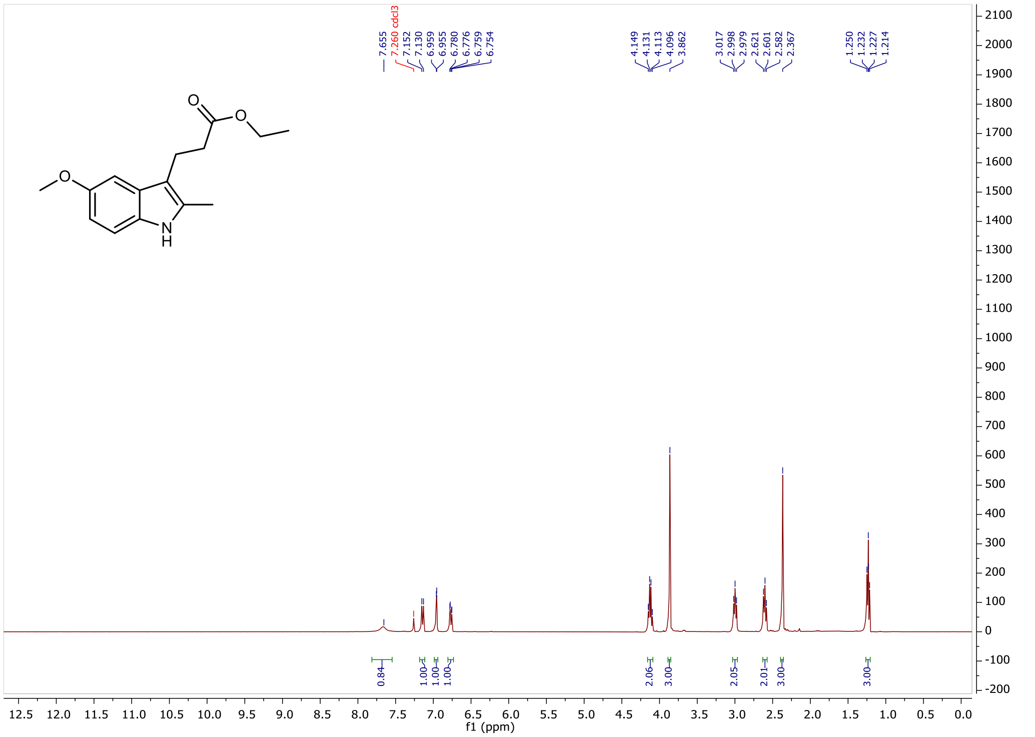

Supplementary Fig. 13. ^{1}H NMR of ethyl 3-(5-bromo-2-methyl-1*H*-indol-3-yl)propanoate (600 MHz, $CDCl_3$)

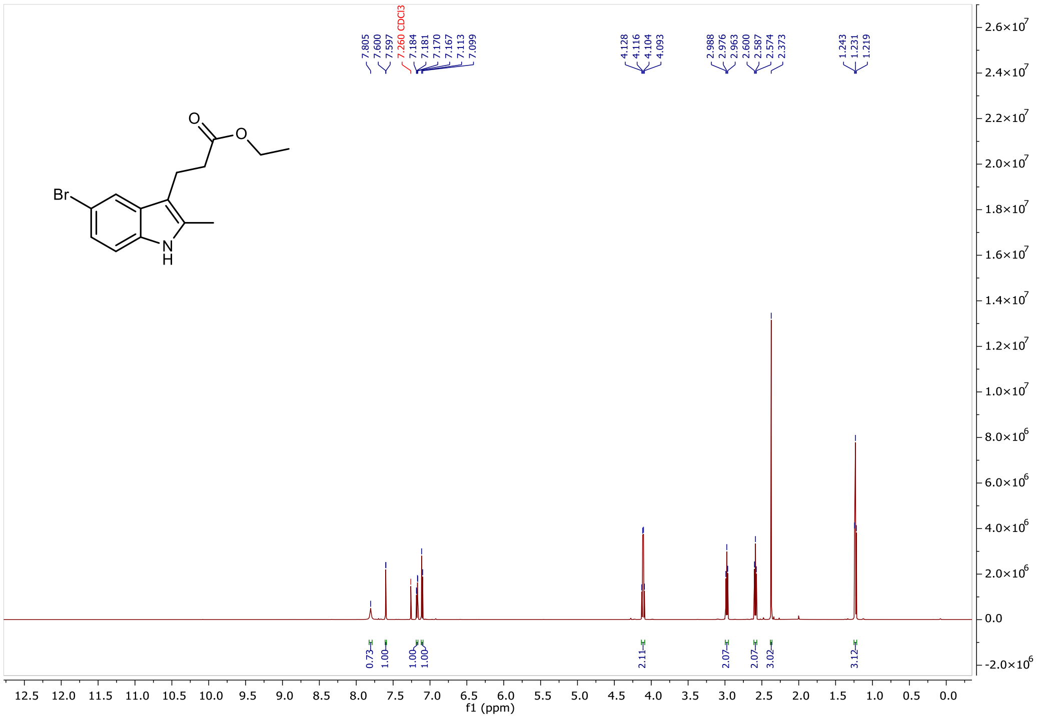

Supplementary Fig. 14. ^{13}C NMR of ethyl 3-(5-bromo-2-methyl-1*H*-indol-3-yl)propanoate (151 MHz, $CDCl_3$)

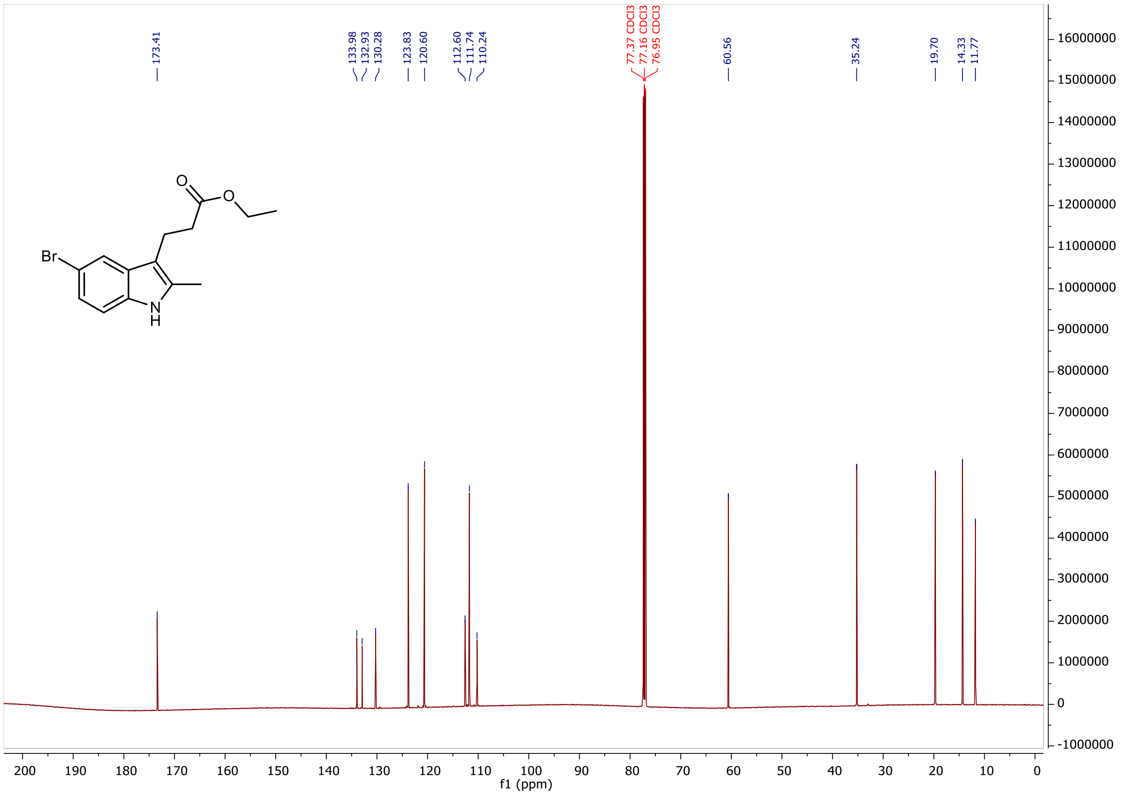

Supplementary Fig. 15. ^{1}H NMR of IA105 (700 MHz, $CDCl_3$)

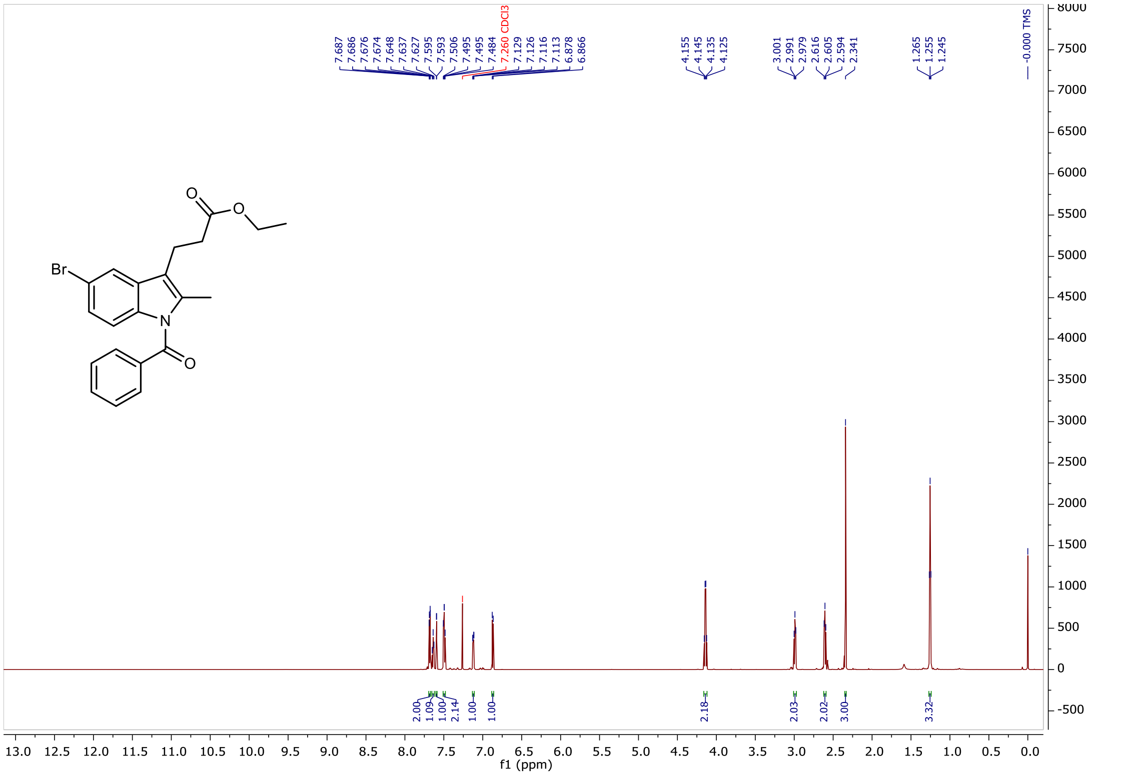

Supplementary Fig. 16. ^{13}C NMR of IA105 (176 MHz, $CDCl_3$)

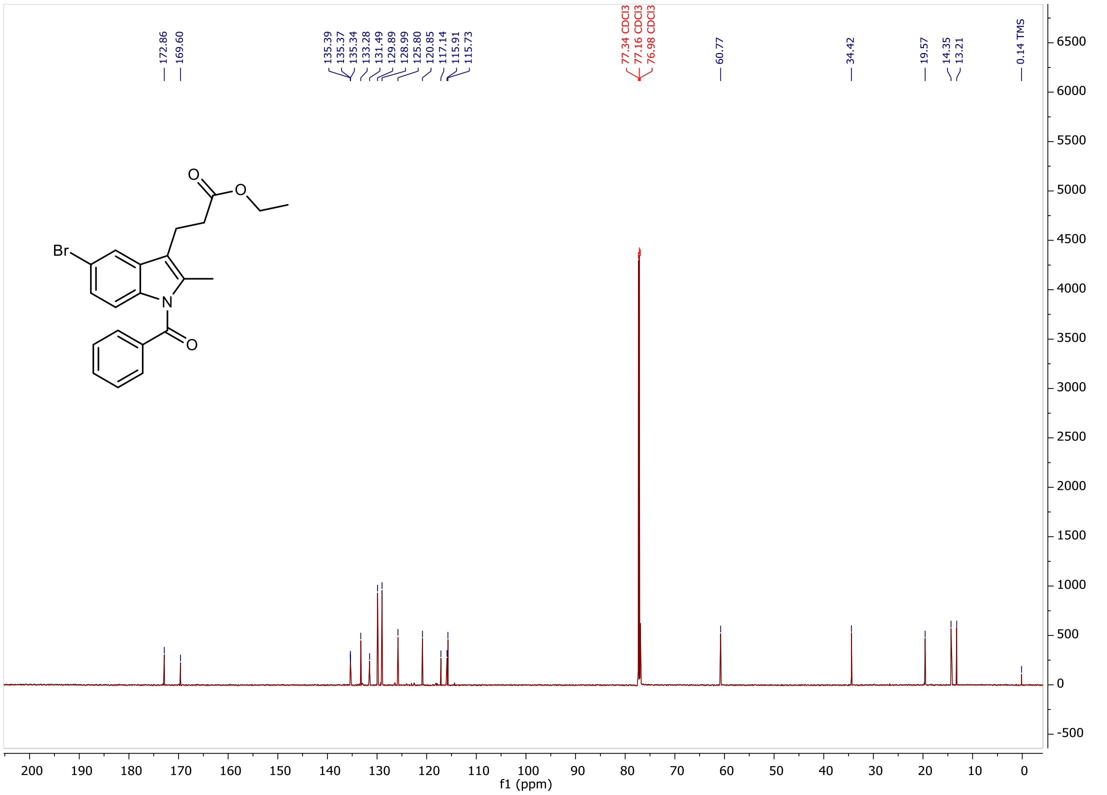

Supplementary Fig. 17. ^{1}H NMR of IA01 (500 MHz, DMSO-d_6)

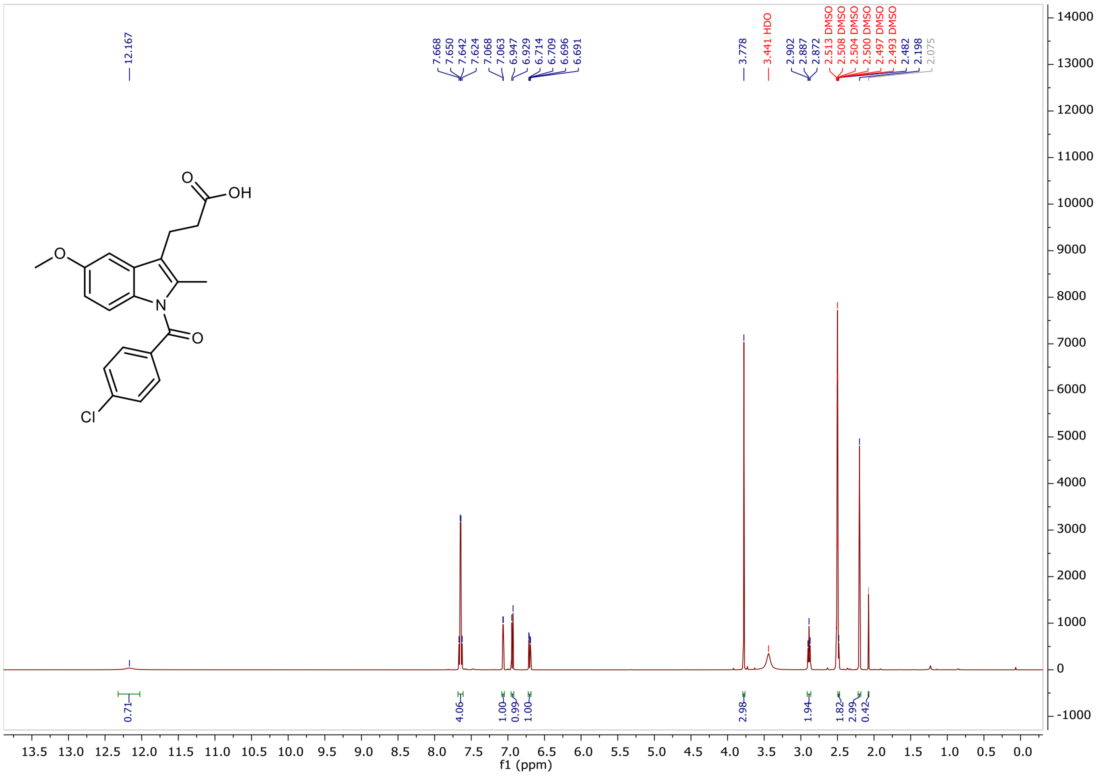

Supplementary Fig. 18. ^{1}H NMR of IA03 (600 MHz, $CDCl_3$)

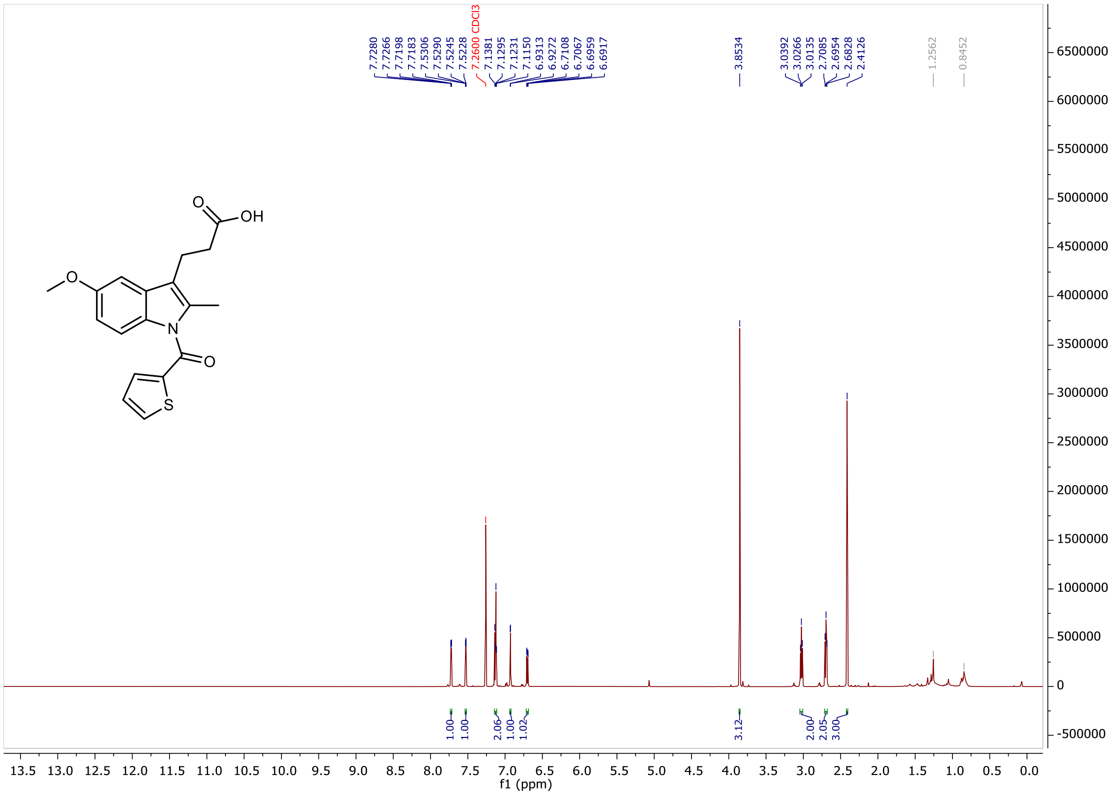

Supplementary Fig. 19. ^{1}H NMR of IA06 (500 MHz, $CDCl_3$)

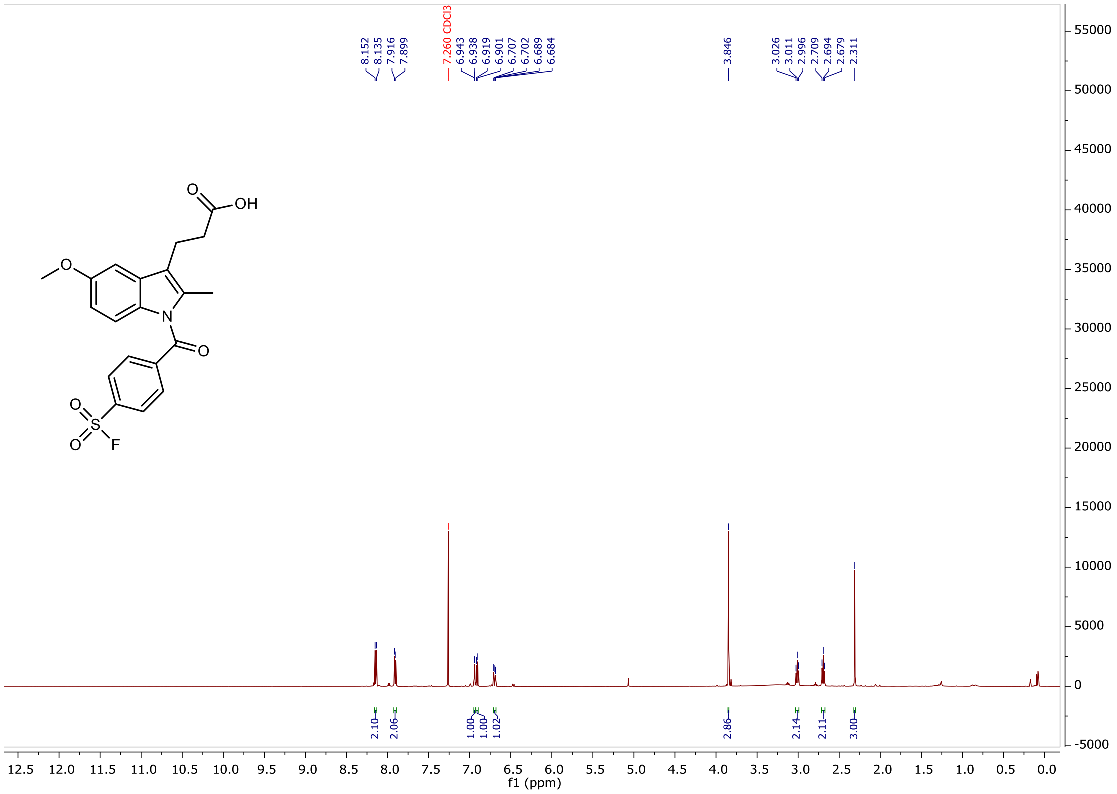

Supplementary Fig. 20. ^{13}C NMR of IA06 (126 MHz, $CDCl_3$)

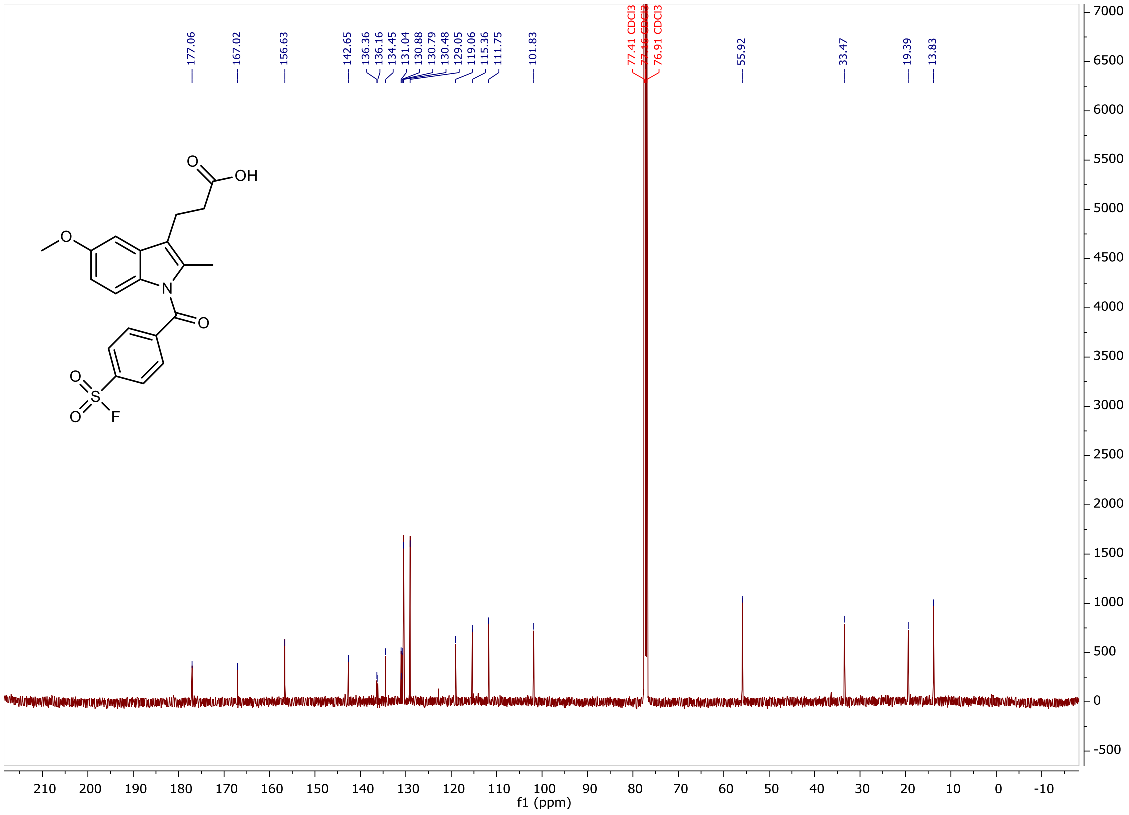

Supplementary Fig. 21. ^{19}F NMR of IA06 (470 MHz, $CDCl_3$)

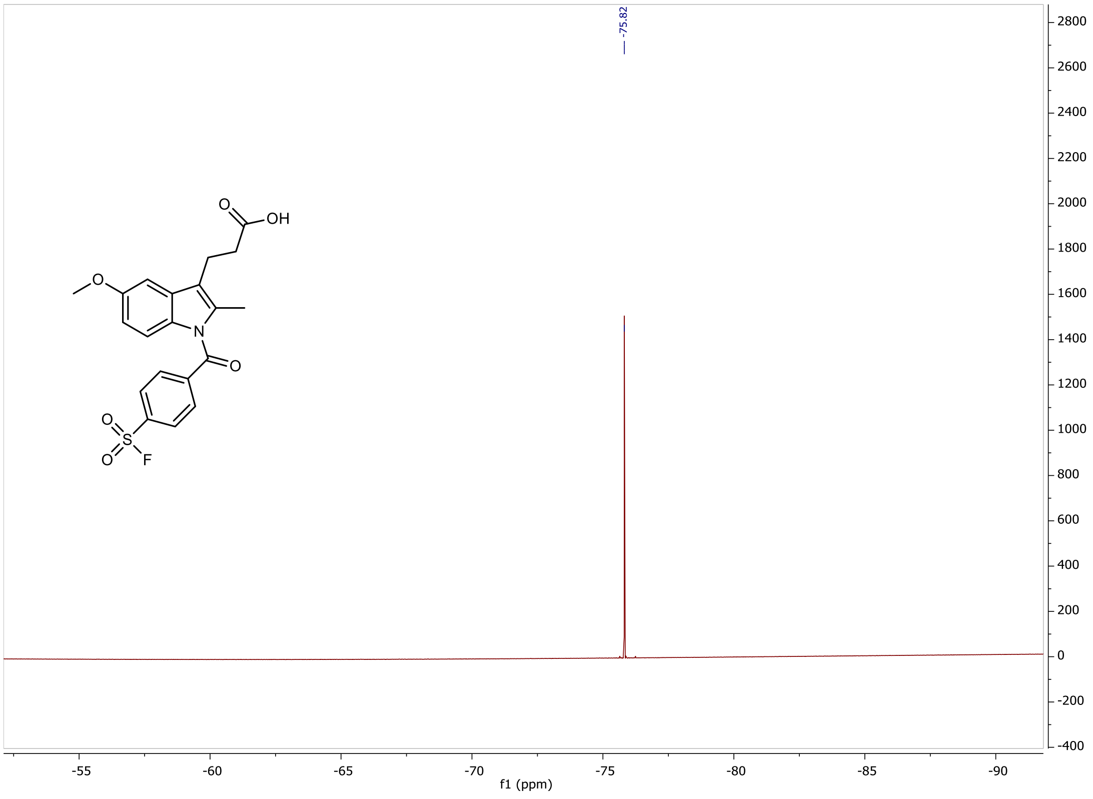

Supplementary Fig. 22. ^{1}H NMR of IA10 (500 MHz, $CDCl_3$)

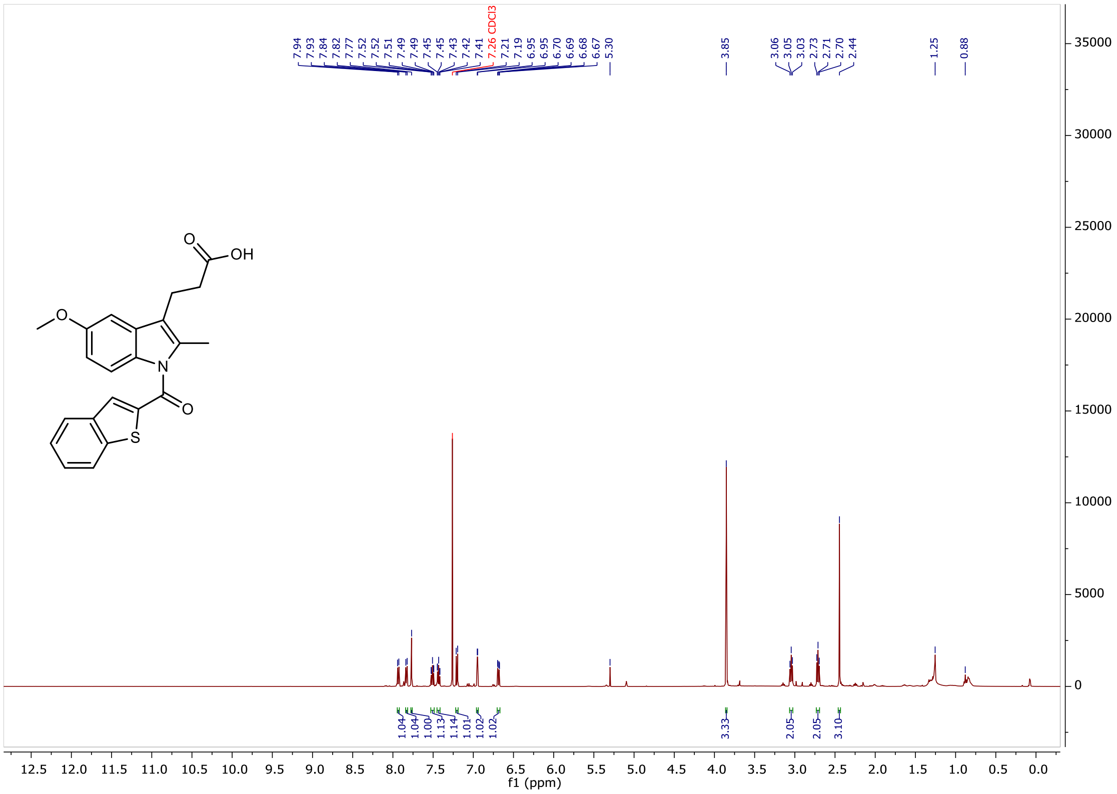

Supplementary Fig. 23. ^{13}C NMR of IA10 (126 MHz, $CDCl_3$)

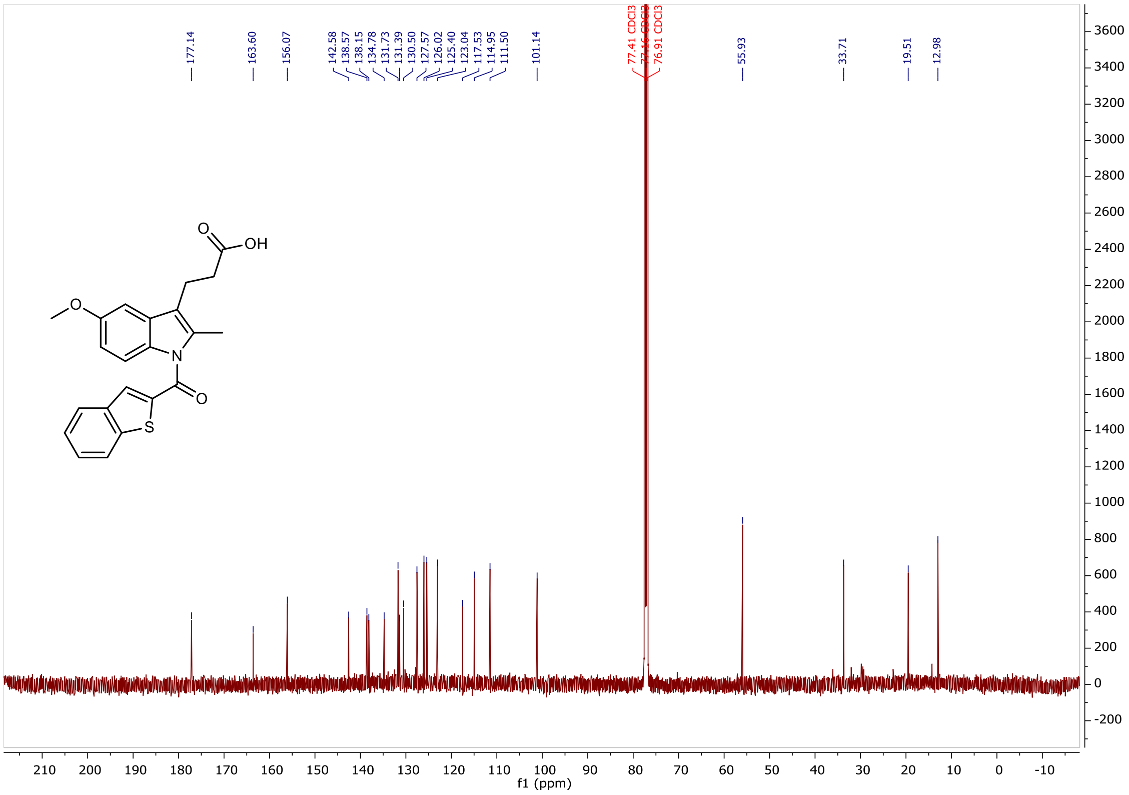

Supplementary Fig. 24. ^{1}**H NMR of IA30 (500 MHz, CDCl**$_3$**)**

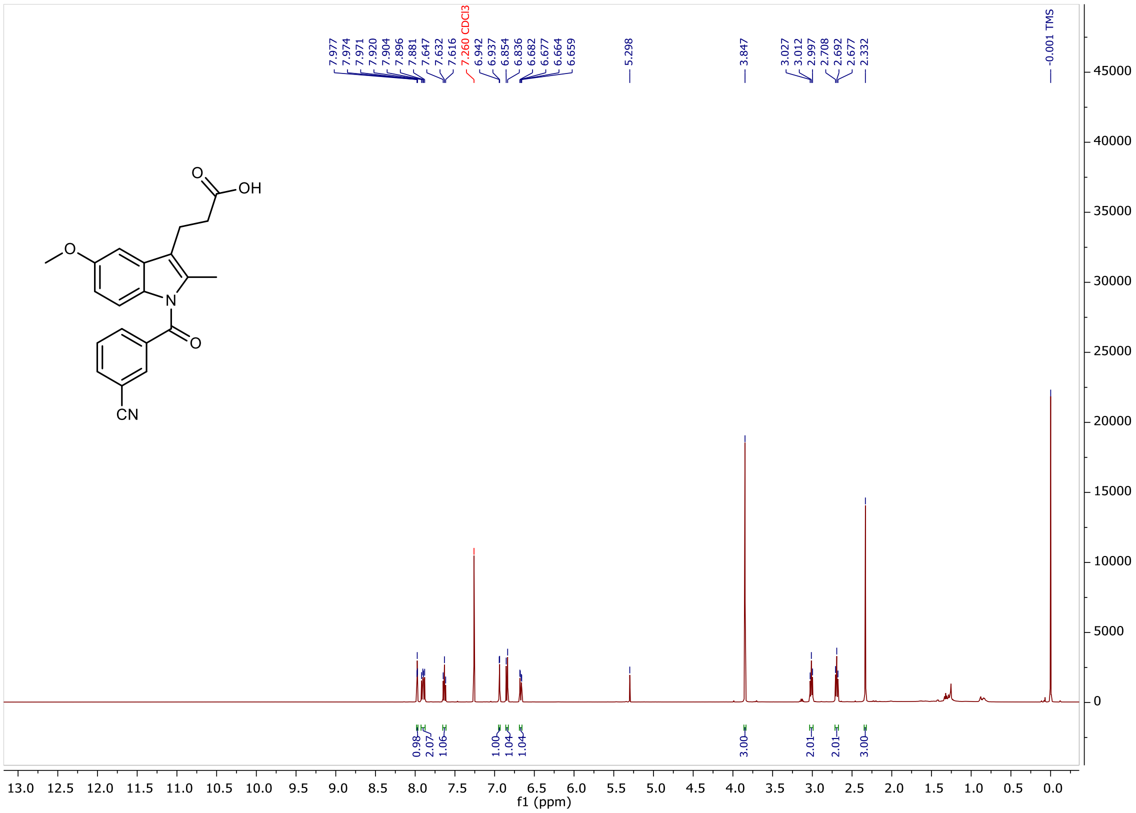

Supplementary Fig. 25. ^{13}**C NMR of IA30 (126 MHz, CDCl**$_3$**)**

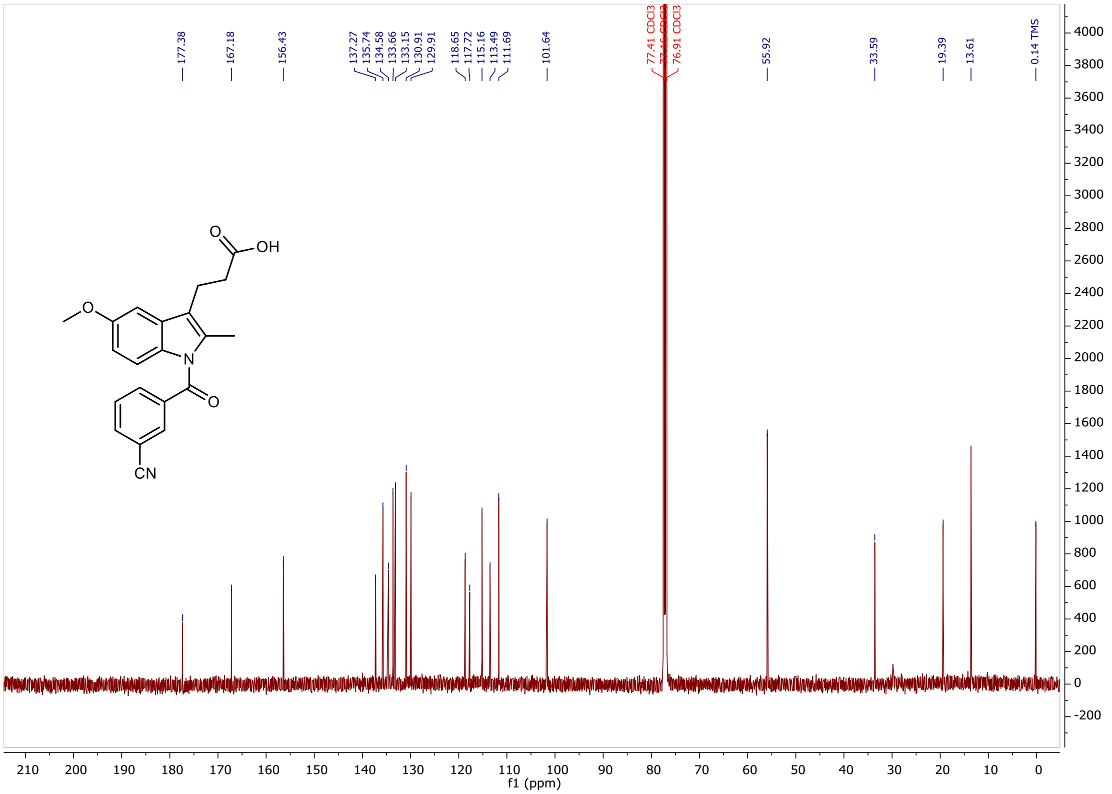

Supplementary Fig. 26. ^{1}H NMR of IA32 (600 MHz, $CDCl_3$)

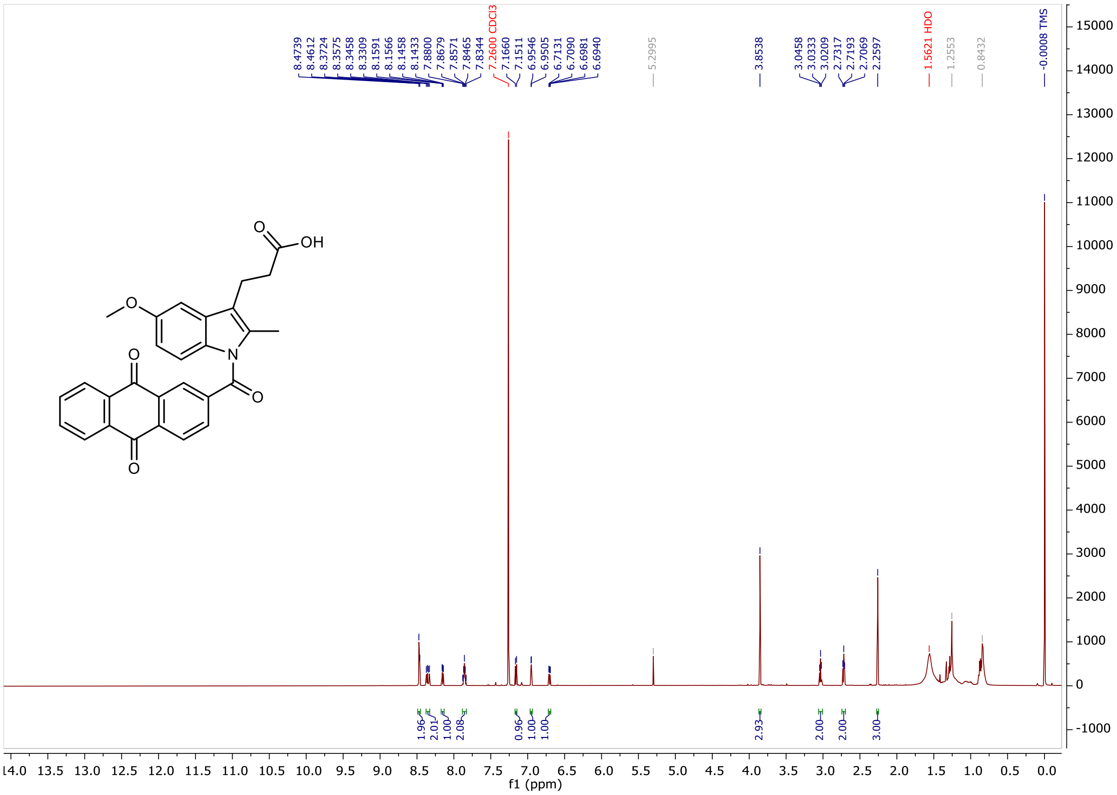

Supplementary Fig. 27. ^{13}C NMR of IA32 (151 MHz, $CDCl_3$)

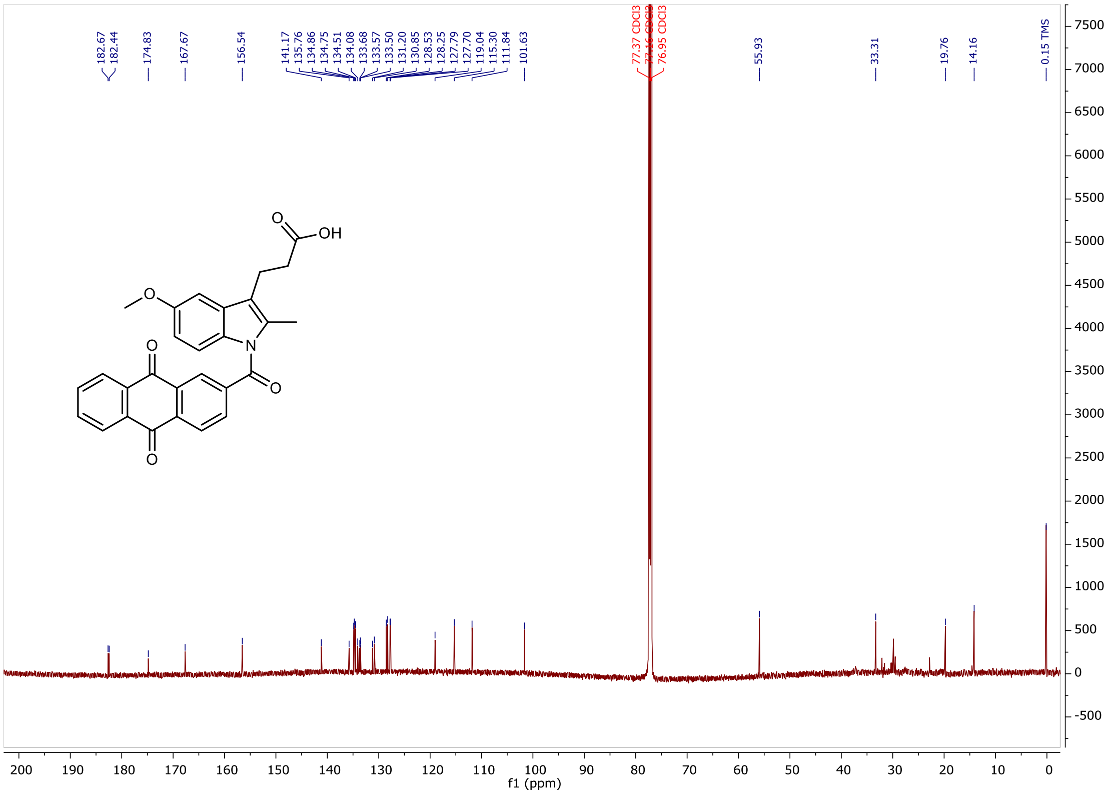

Supplementary Fig. 28. ^{1}H NMR of IA34 (500 MHz, $CDCl_3$)

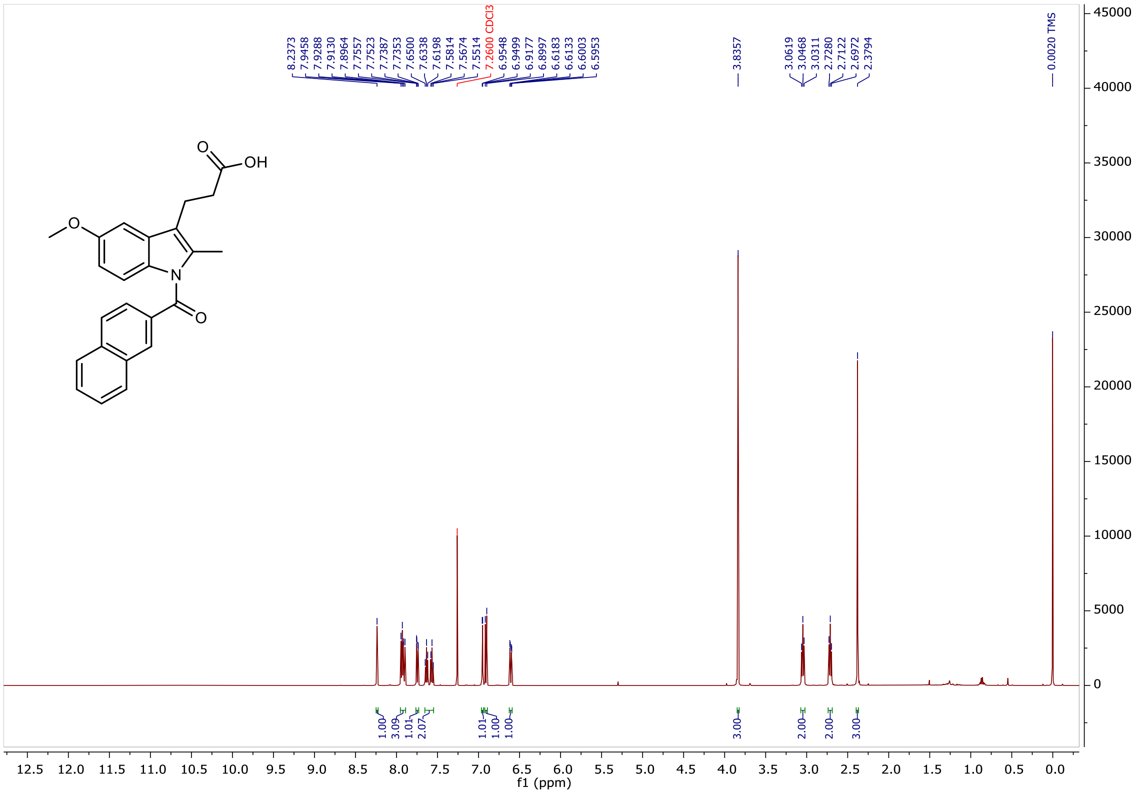

Supplementary Fig. 29. ^{13}C NMR of IA34 (126 MHz, $CDCl_3$)

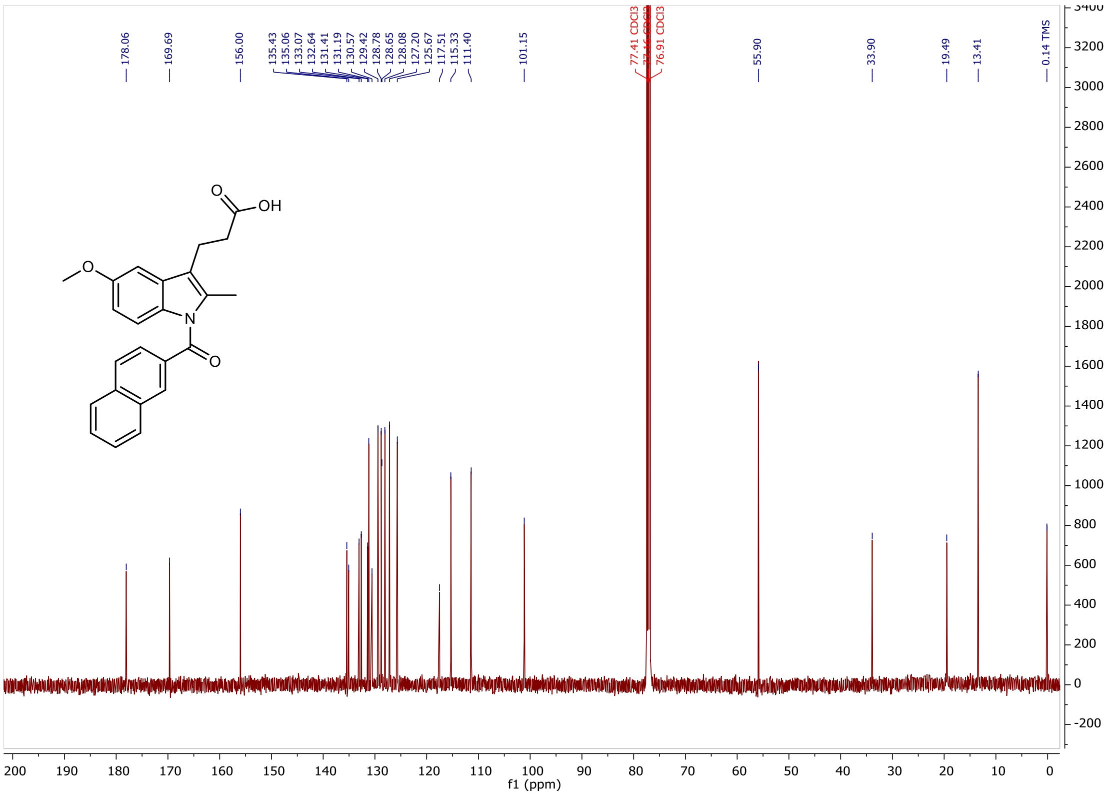

Supplementary Fig. 30. ^{1}H NMR of IA36 (600 MHz, $CDCl_3$)

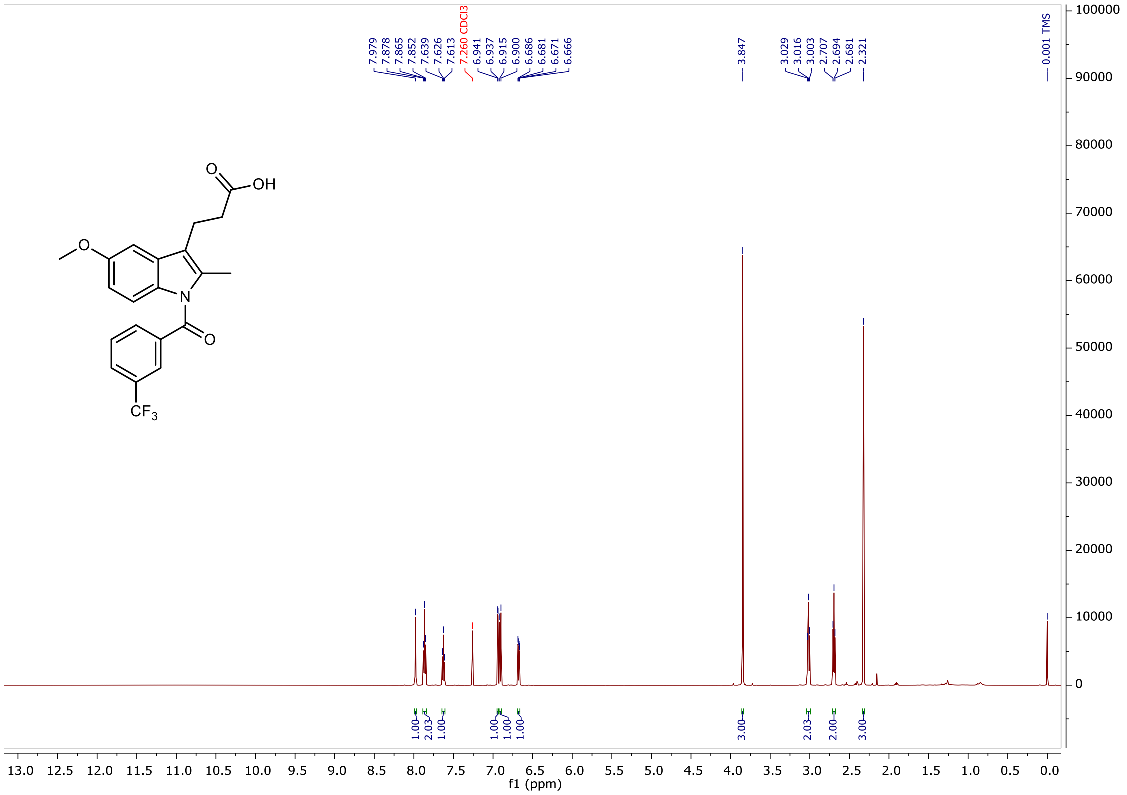

Supplementary Fig. 31. ^{13}C NMR of IA36 (151 MHz, $CDCl_3$)

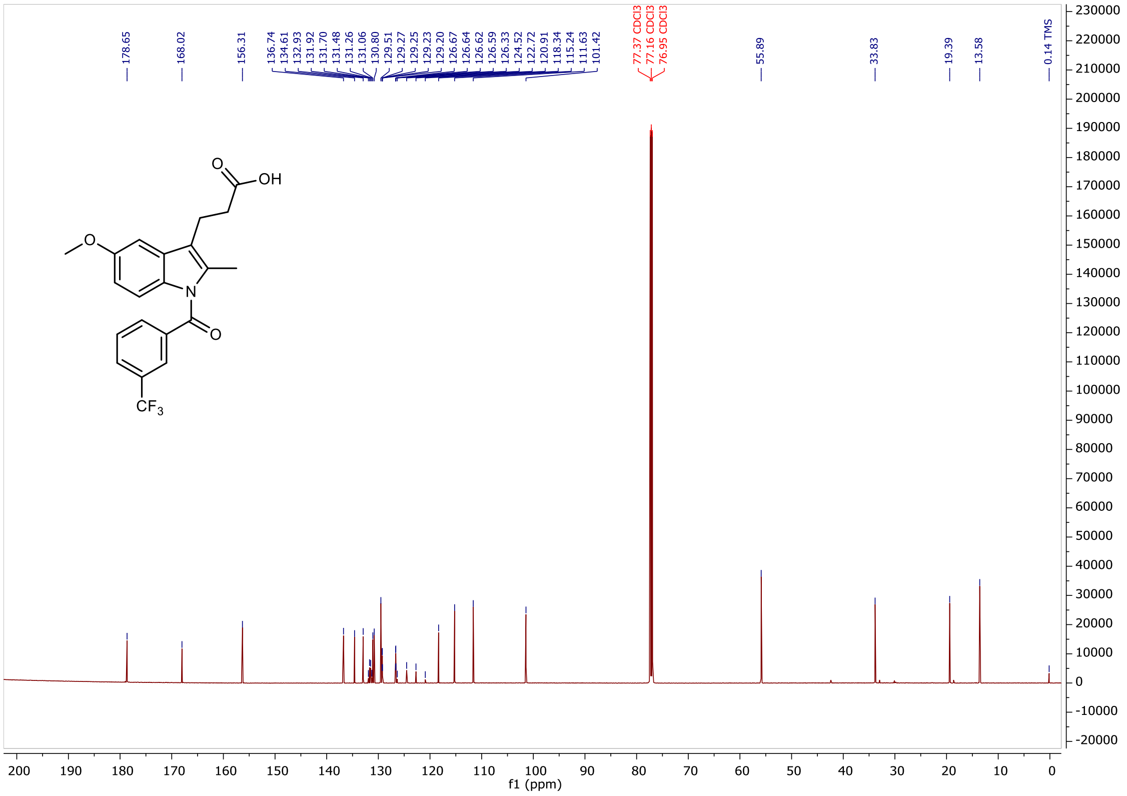

Supplementary Fig. 32. ^{19}**F NMR of IA36 (565 MHz, CDCl₃)**

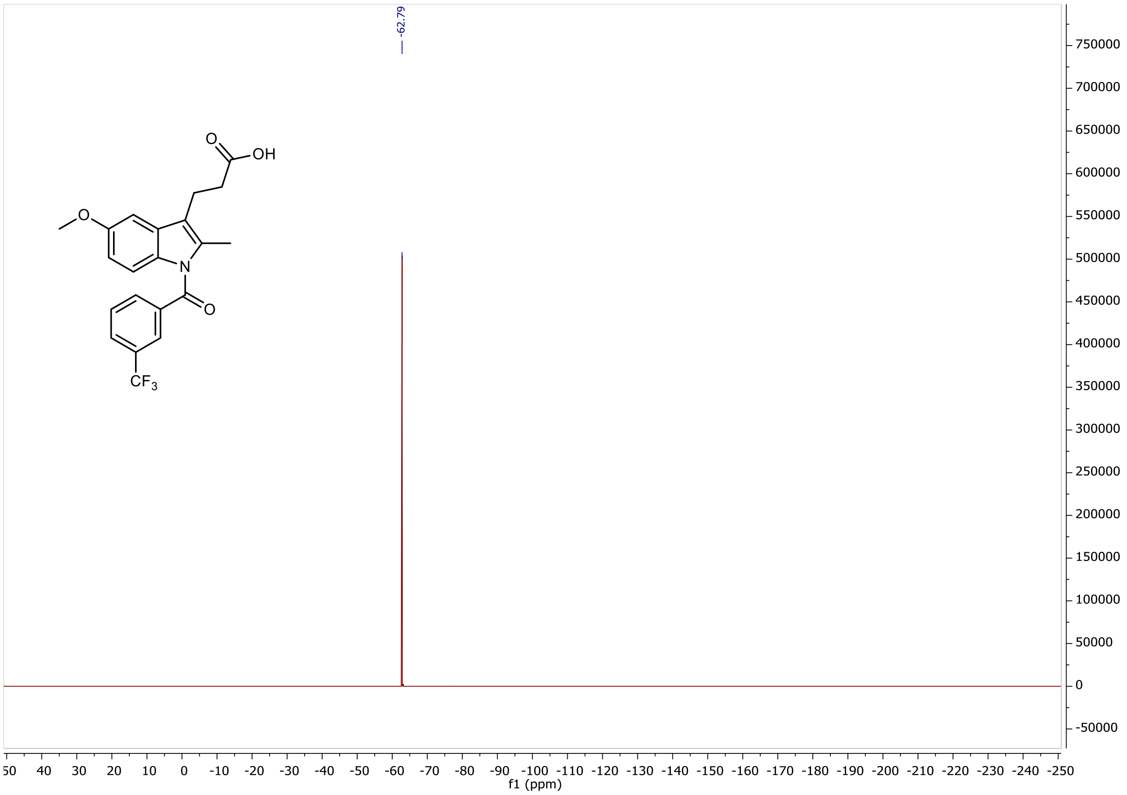

Supplementary Fig. 33. ^{1}**H NMR of IA64 (700 MHz, CDCl₃)**

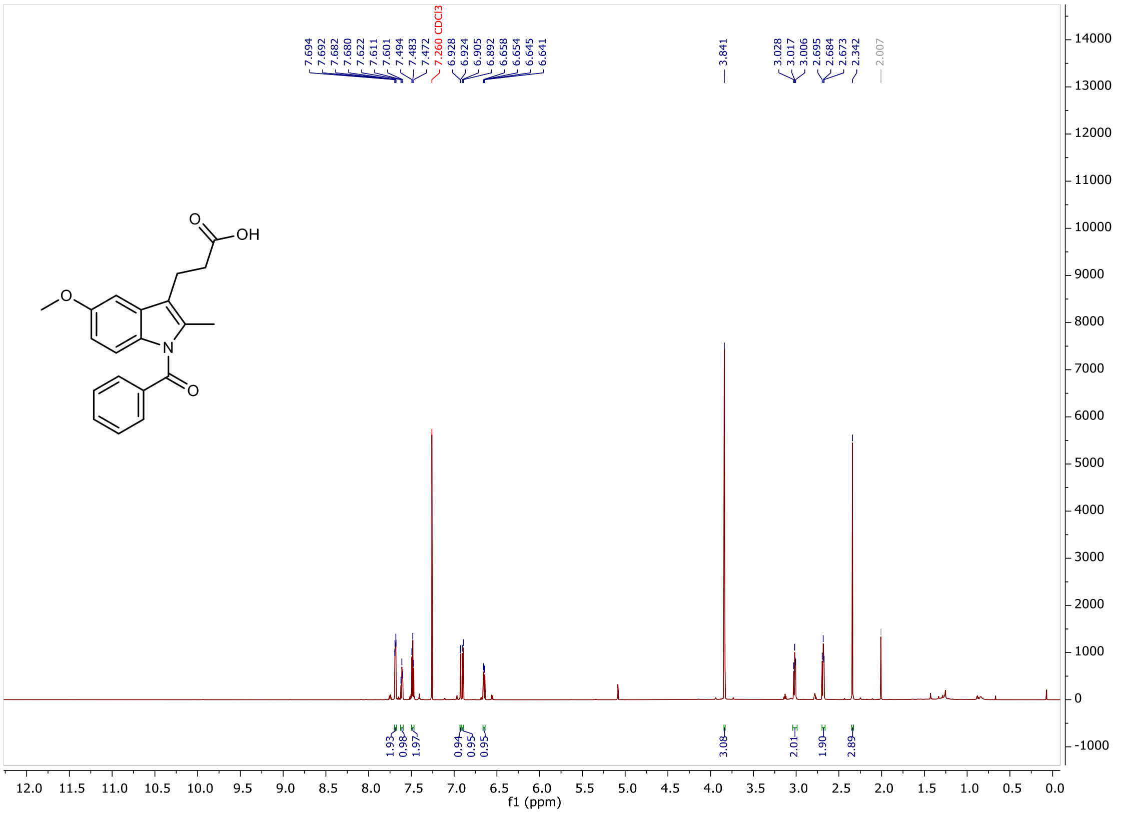

Supplementary Fig. 34. ^{13}C NMR of IA64 (176 MHz, $CDCl_3$)

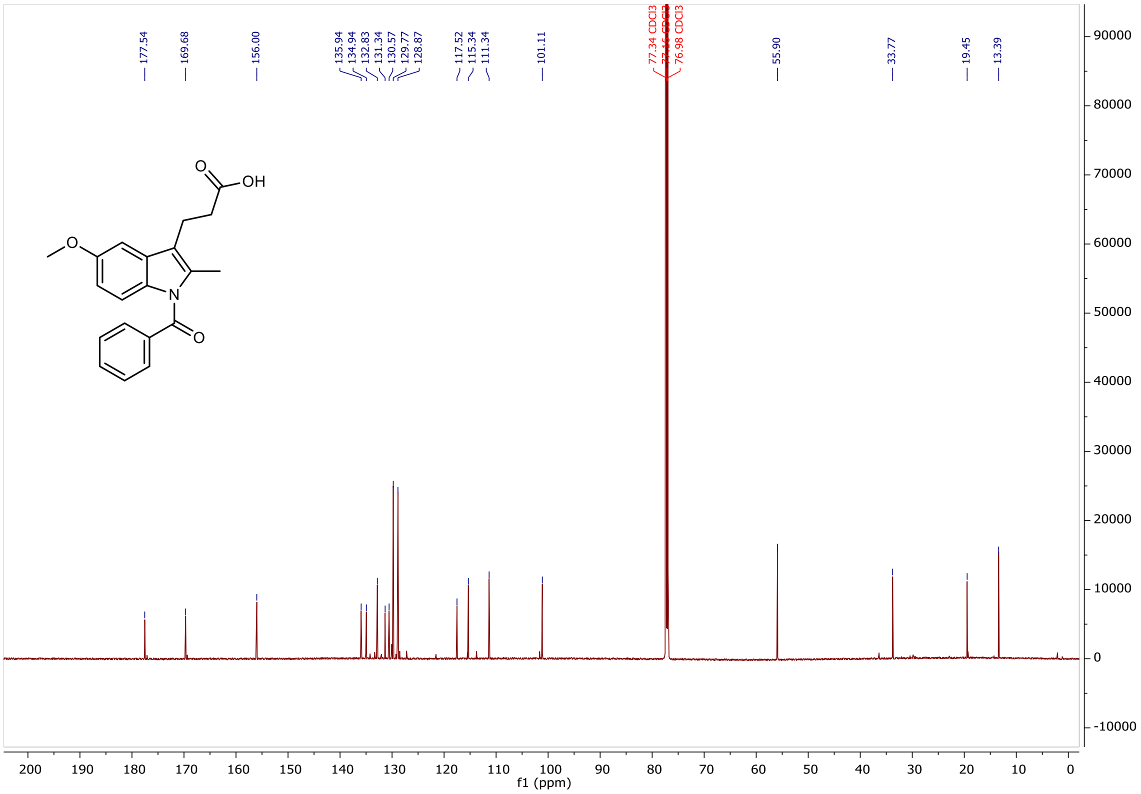

Supplementary Fig. 35. ^{1}H NMR of IA66 (600 MHz, $CDCl_3$)

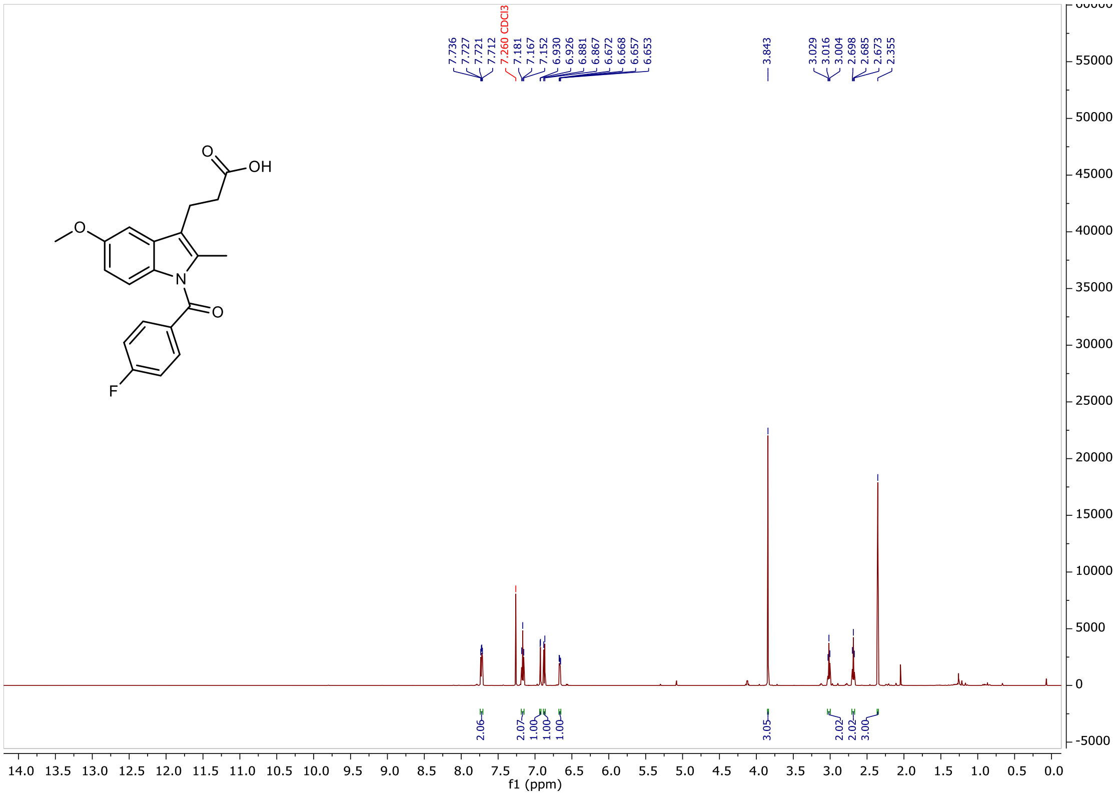

Supplementary Fig. 36. ^{13}C NMR of IA66 (151 MHz, $CDCl_3$)

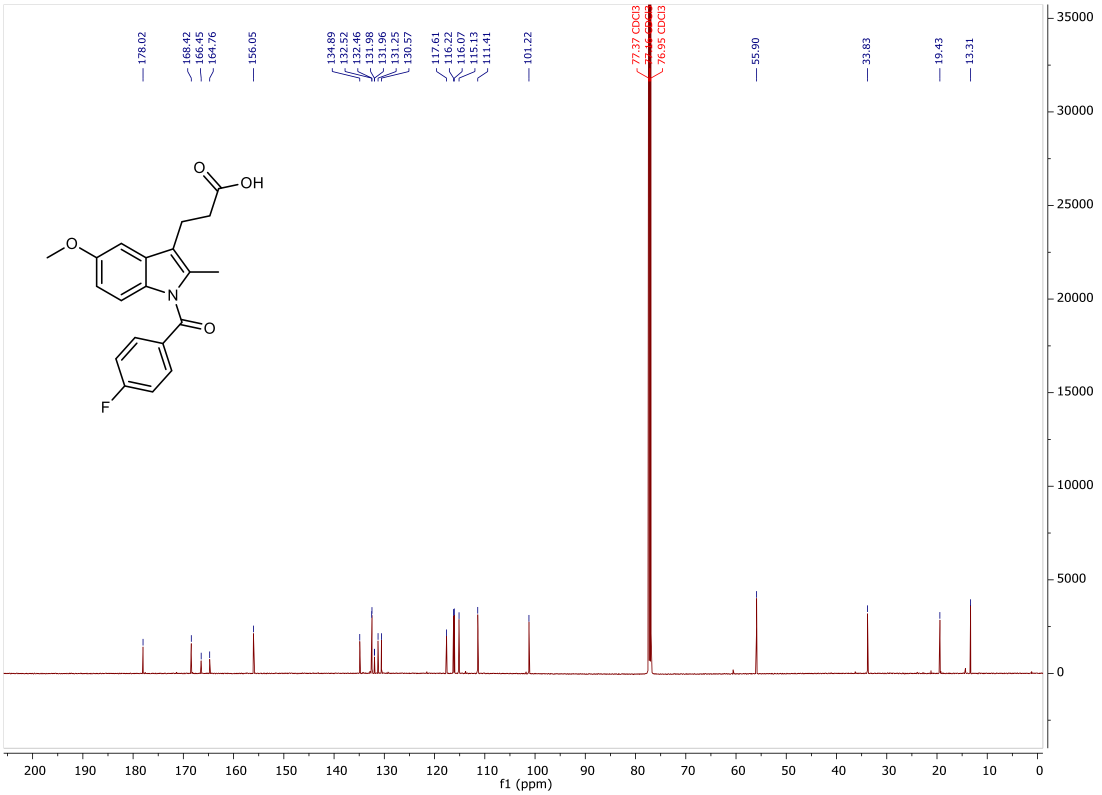

Supplementary Fig. 37. ^{1}H NMR of IA70 (700 MHz, $CDCl_3$)

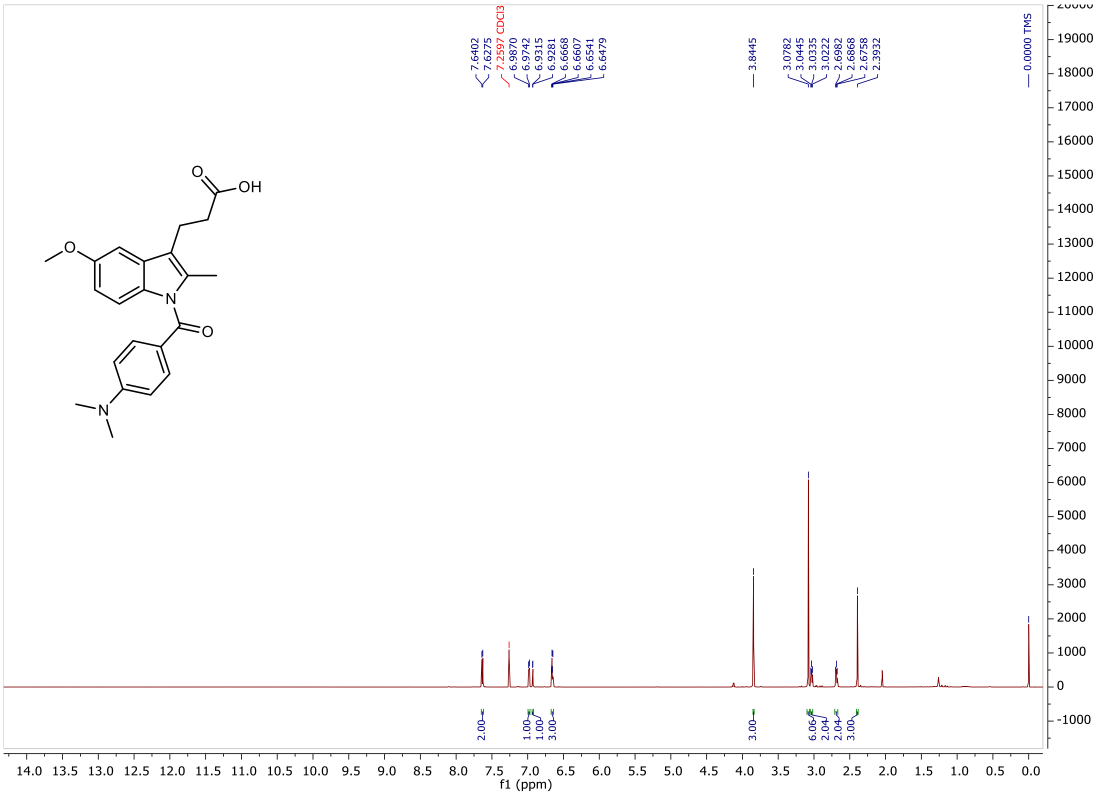

Supplementary Fig. 38. ^{13}C NMR of IA70 (176 MHz, CDCl$_3$)

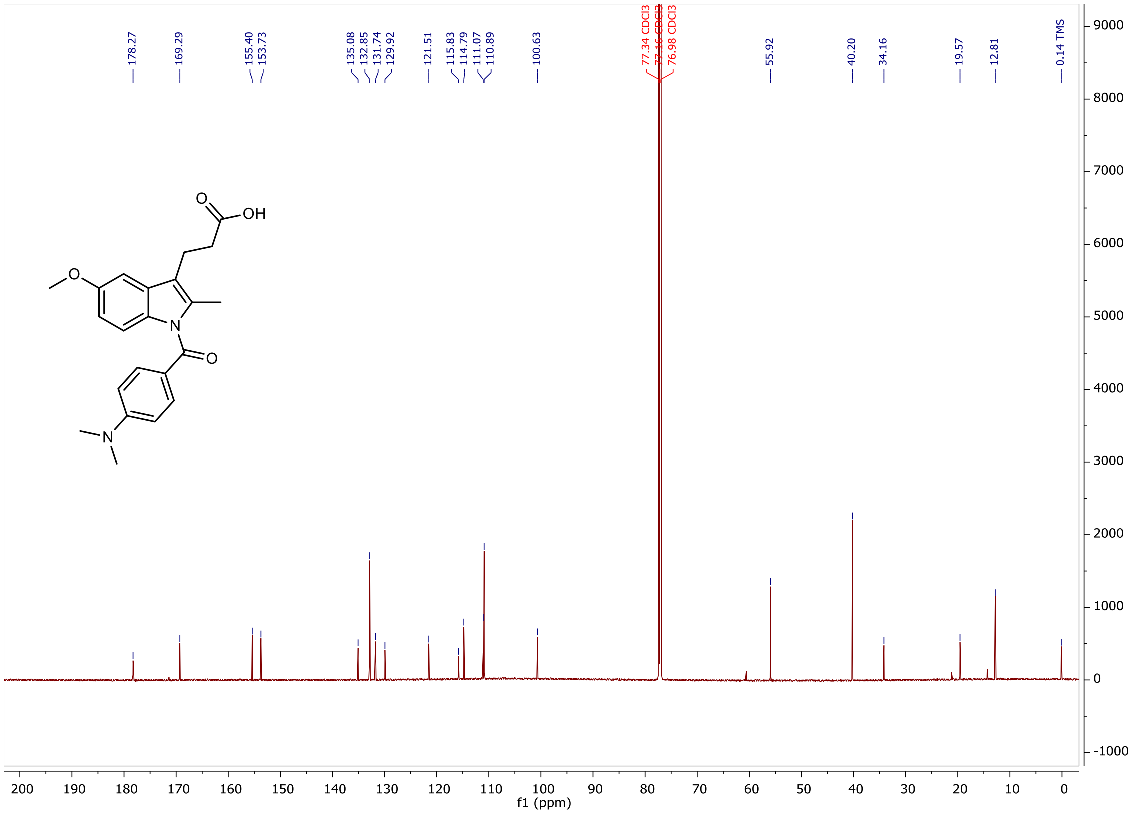

Supplementary Fig. 39. ^{1}H NMR of IA75 (700 MHz, CDCl$_3$)

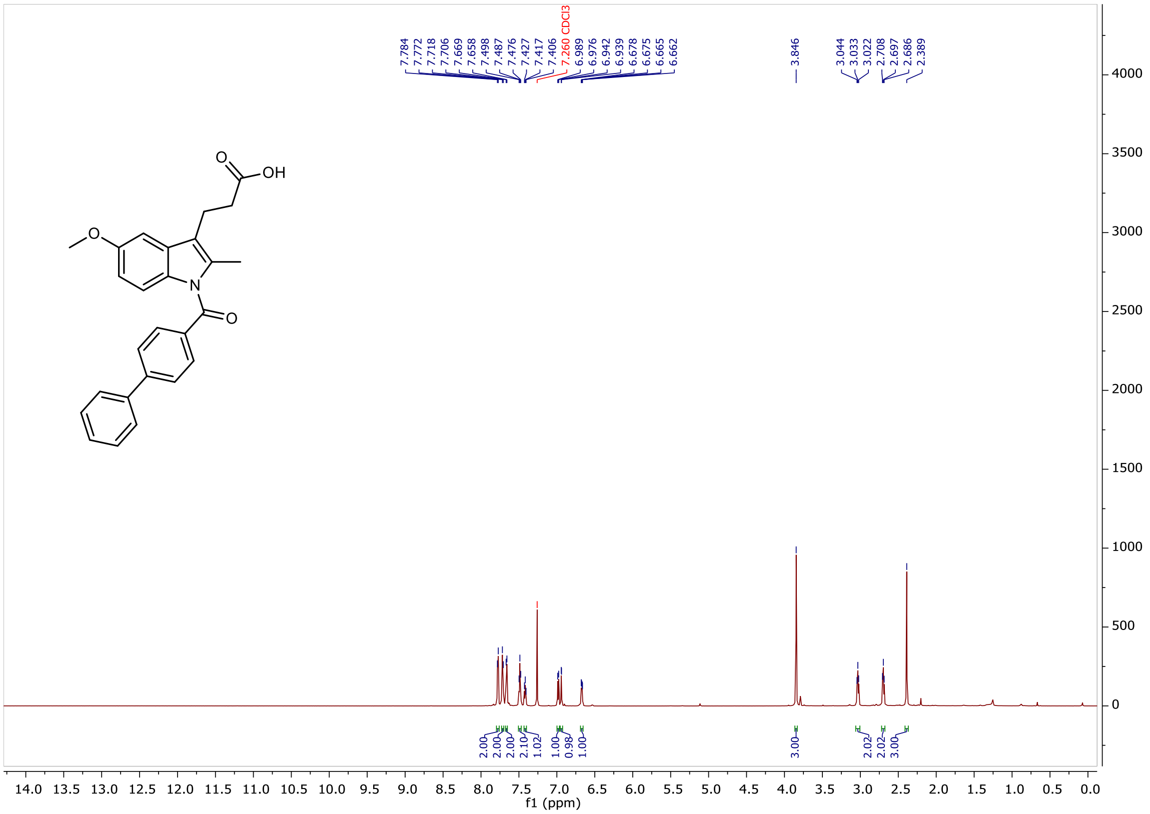

Supplementary Fig. 40. ^{13}C NMR of IA75 (176 MHz, $CDCl_3$)

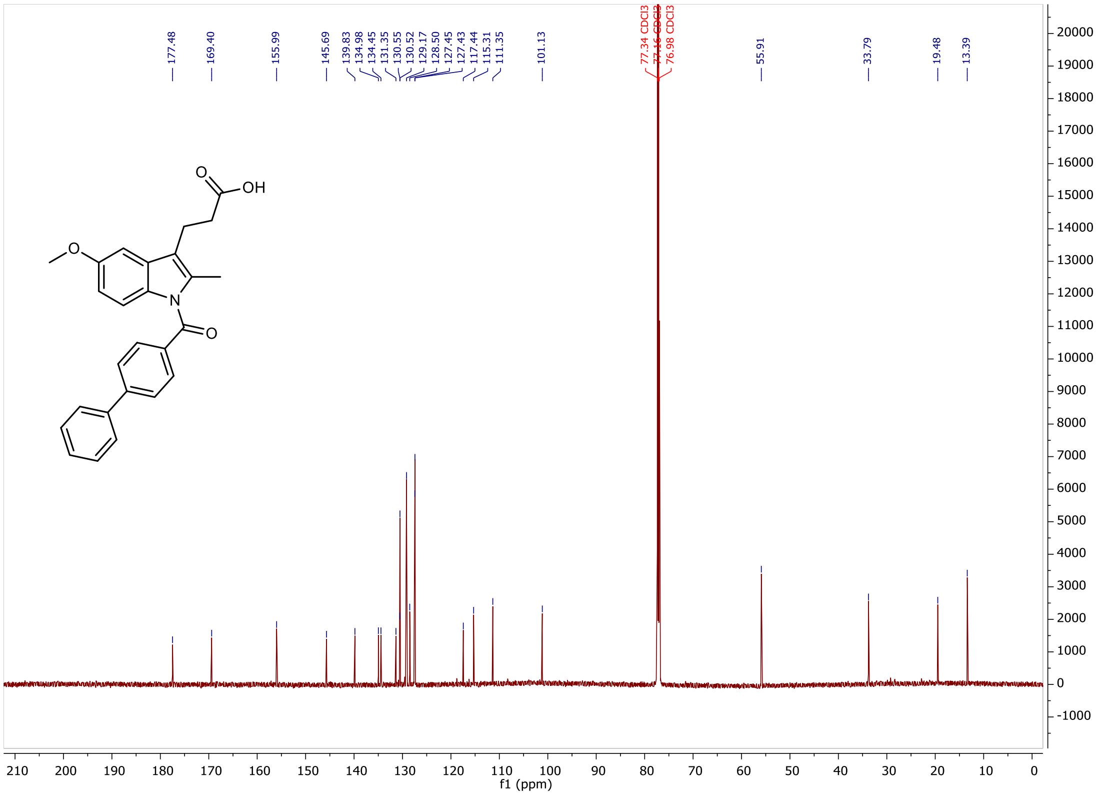

Supplementary Fig. 41. ^{1}H NMR of IA76 (500 MHz, $CDCl_3$)

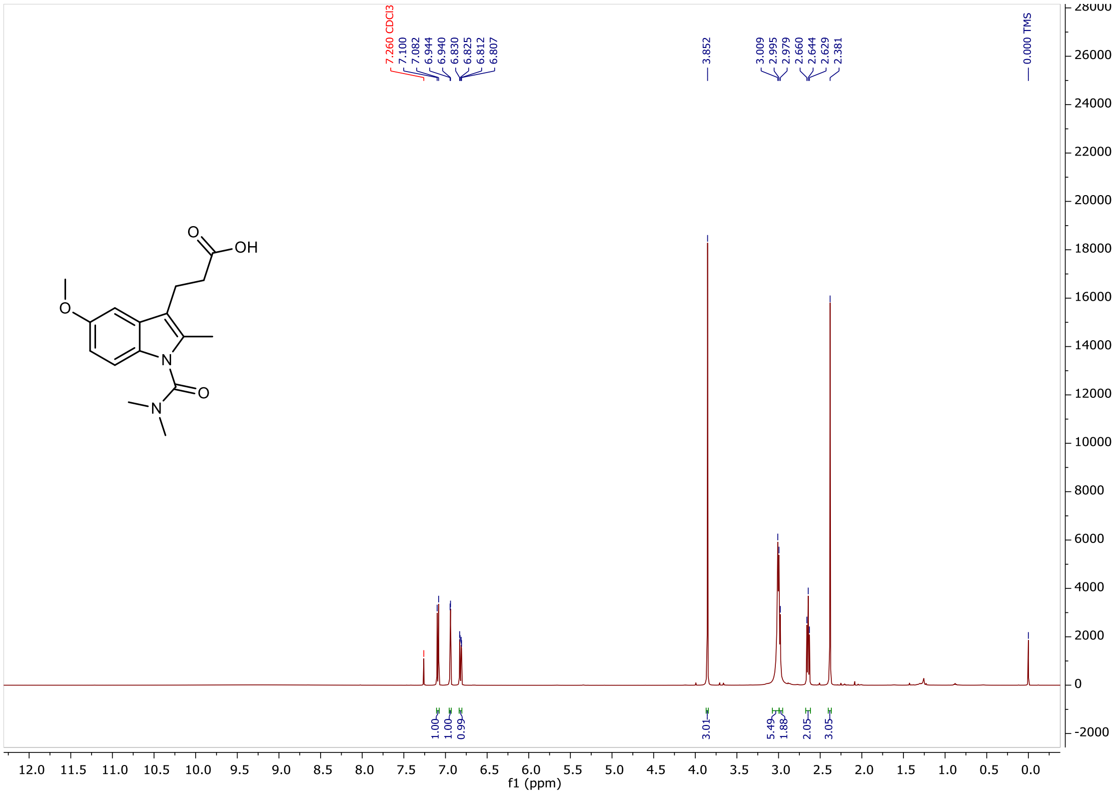

Supplementary Fig. 42. ^{13}**C NMR of IA76 (126 MHz, CDCl$_3$)**

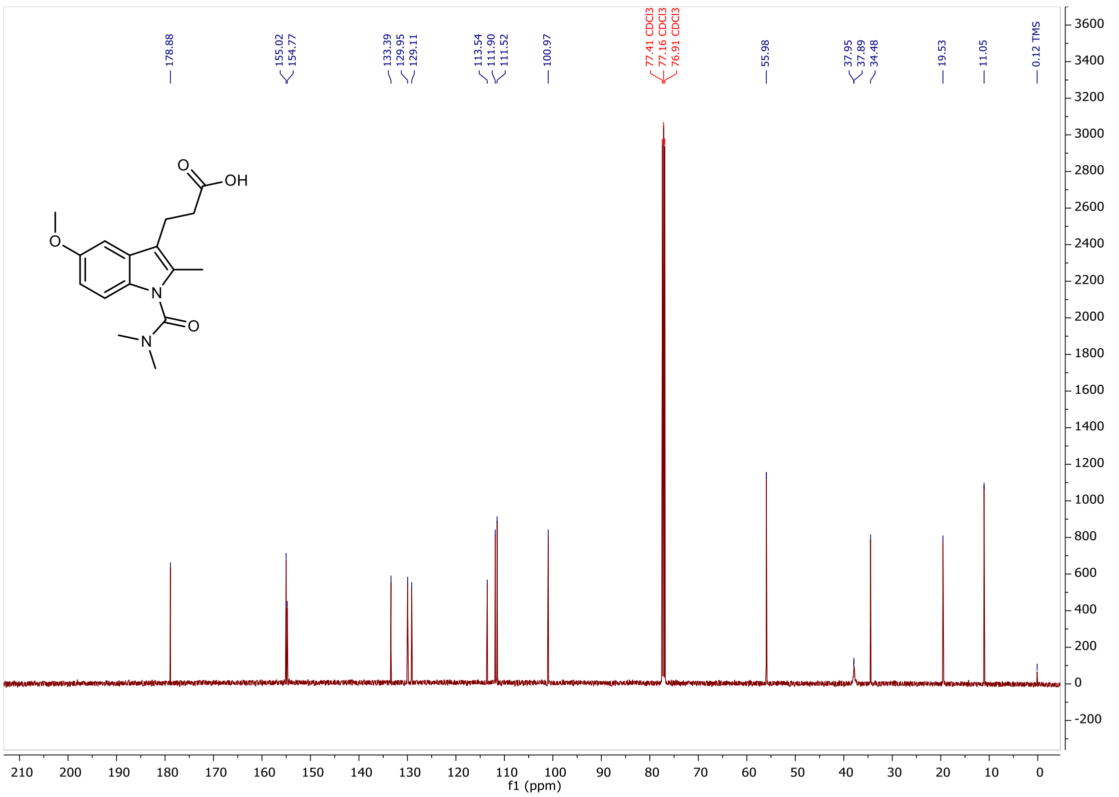

Supplementary Fig. 43. ^{1}**H NMR of IA77 (600 MHz, CDCl$_3$)**

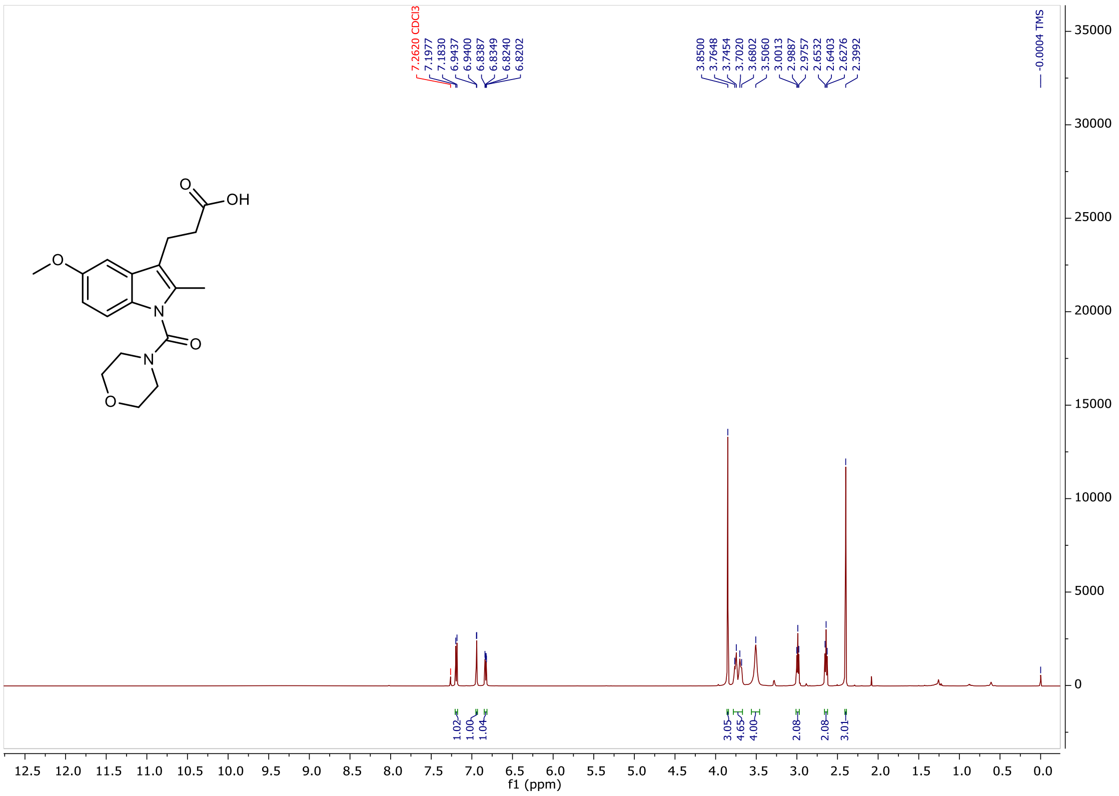

Supplementary Fig. 44. ^{13}C NMR of IA77 (151 MHz, $CDCl_3$)

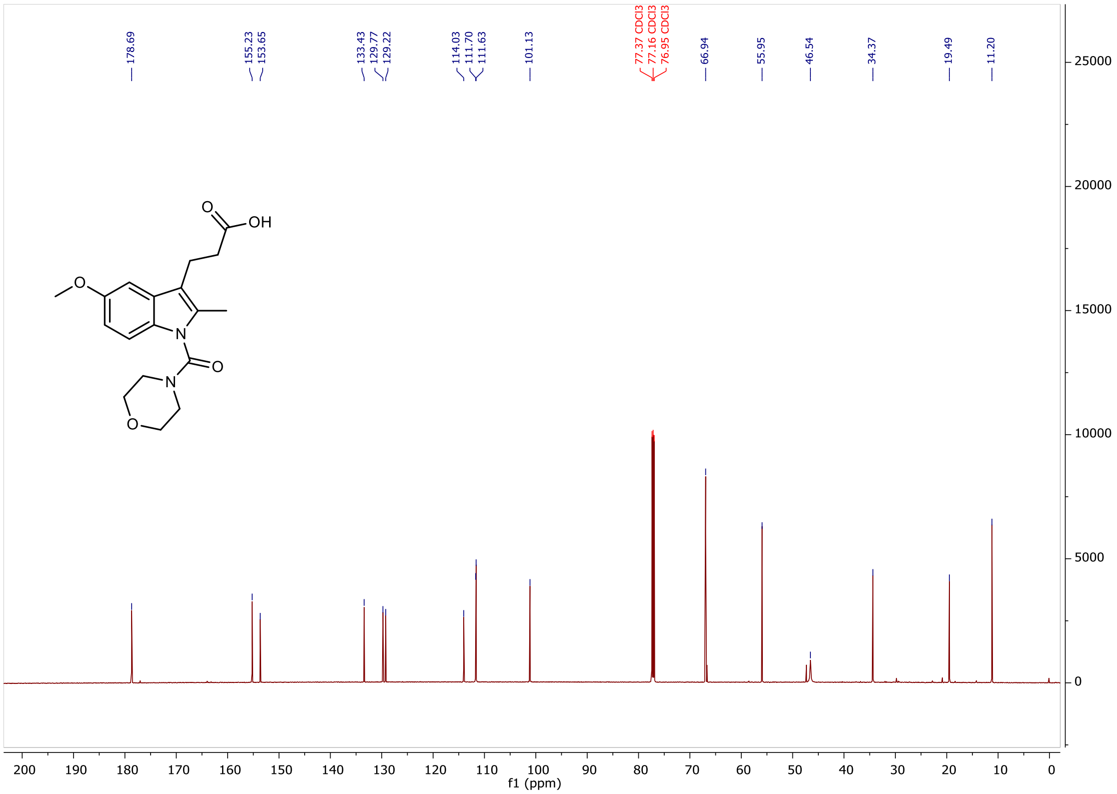

Supplementary Fig. 45. ^{1}H NMR of IA81 (500 MHz, $CDCl_3$)

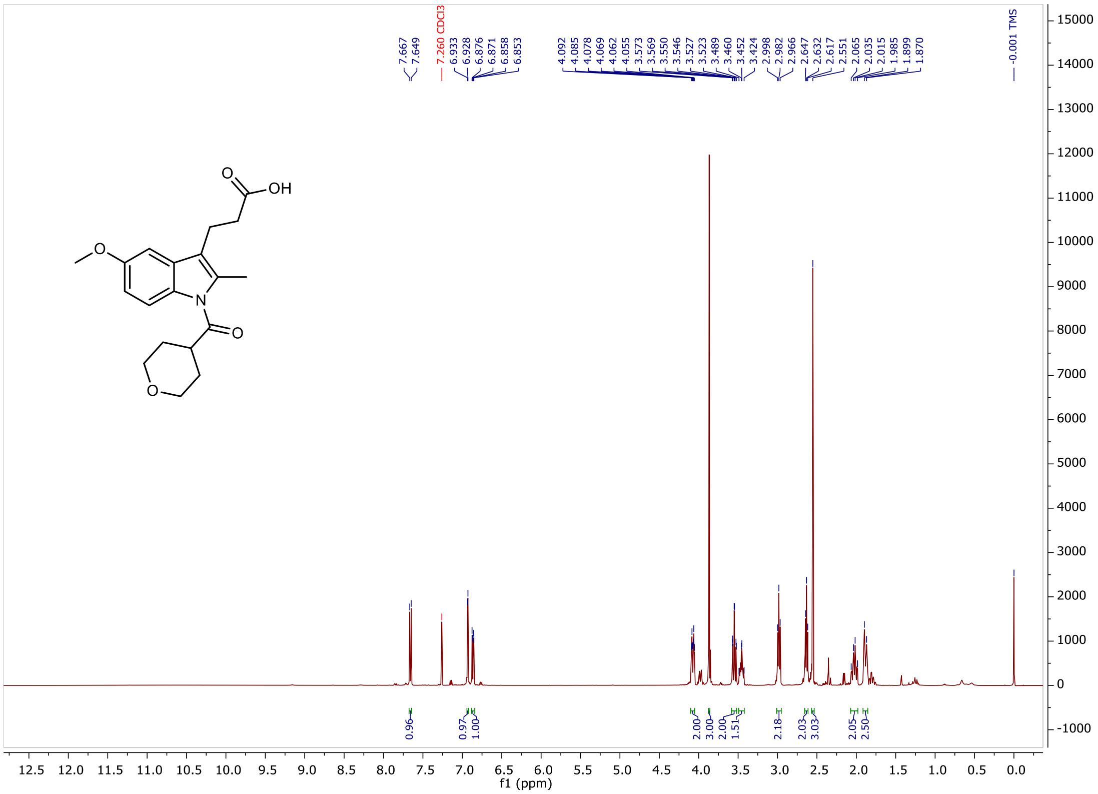

Supplementary Fig. 46. ^{13}C NMR of IA81 (126 MHz, $CDCl_3$)

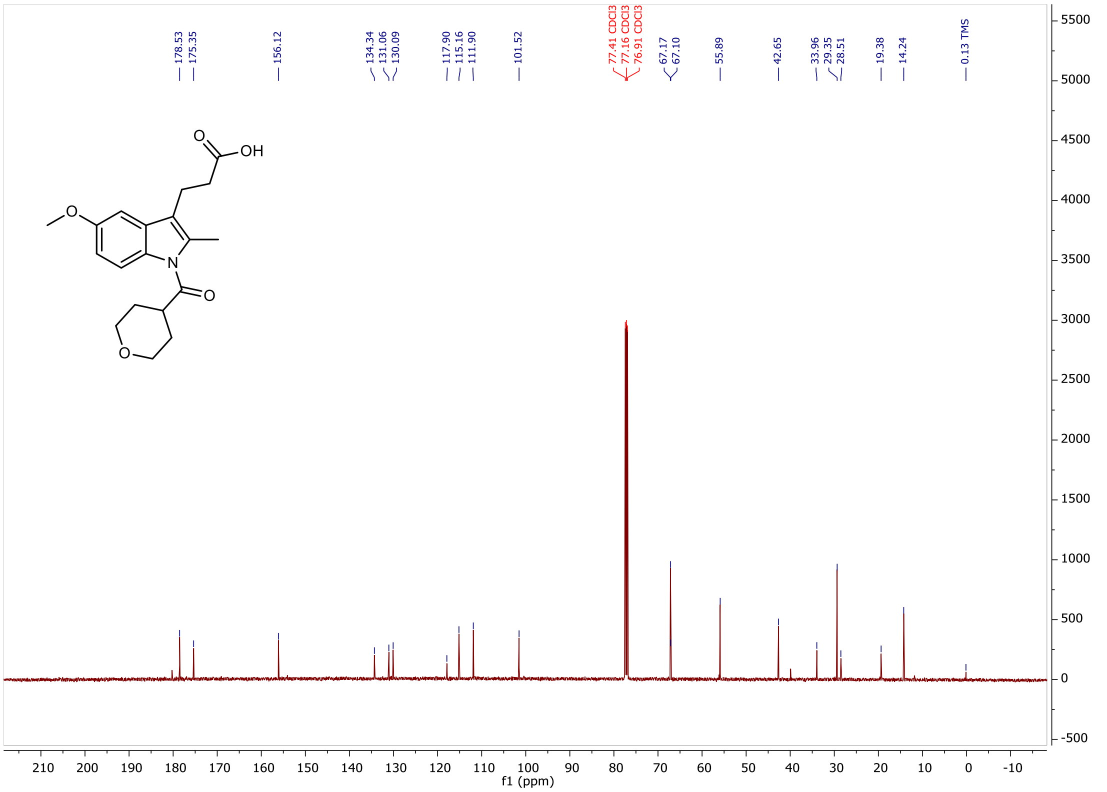

Supplementary Fig. 47. ^{1}H NMR of IA84 (700 MHz, $CDCl_3$)

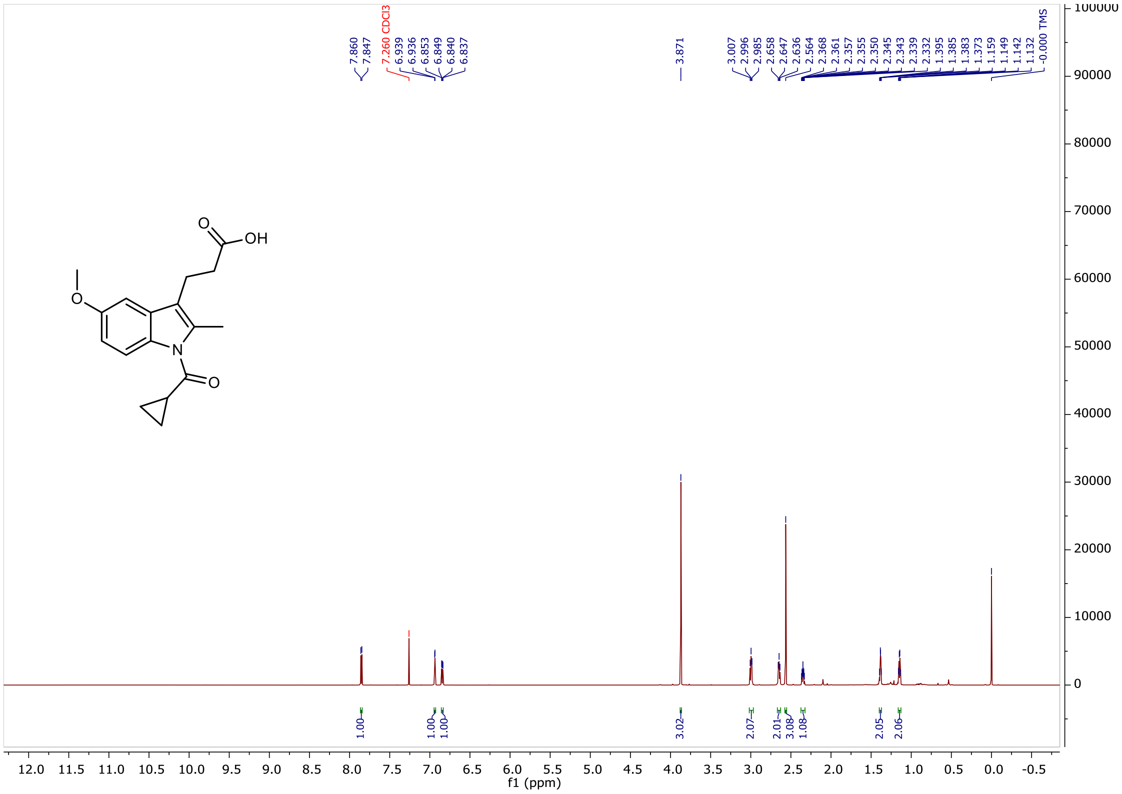

Supplementary Fig. 48. ^{13}C NMR of IA84 (176 MHz, CDCl$_3$)

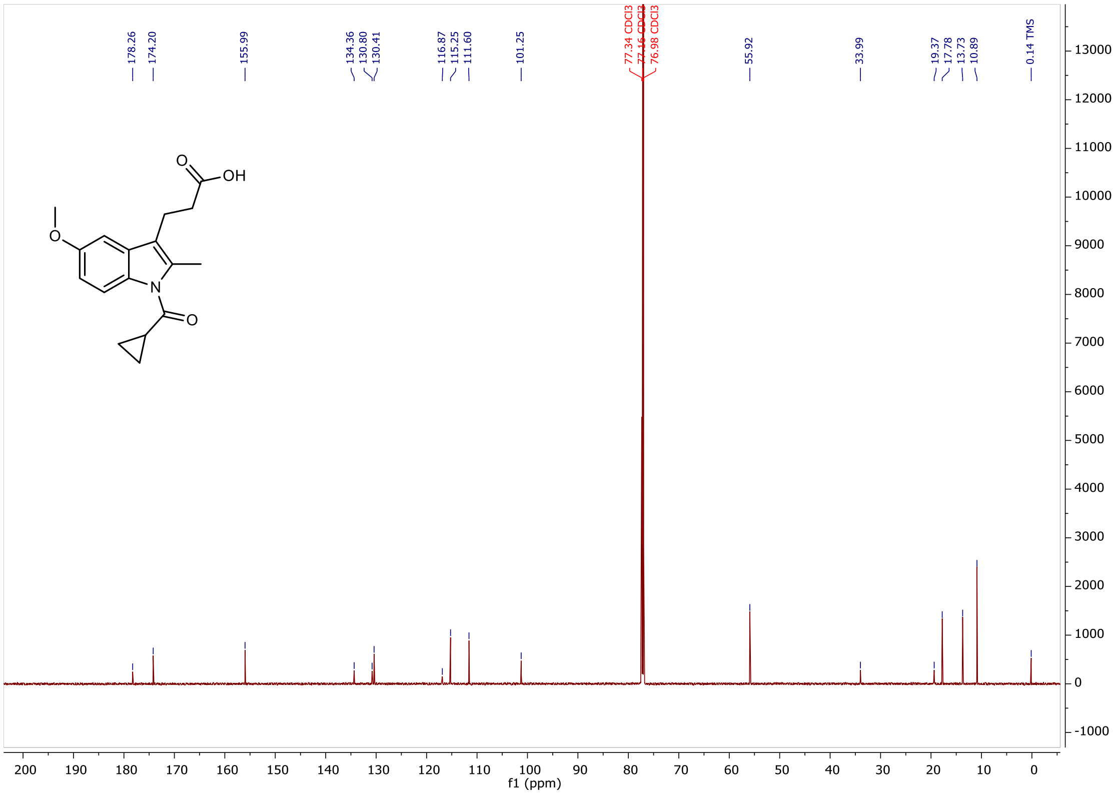

Supplementary Fig. 49. ^{1}H NMR of IA89 (500 MHz, CDCl$_3$)

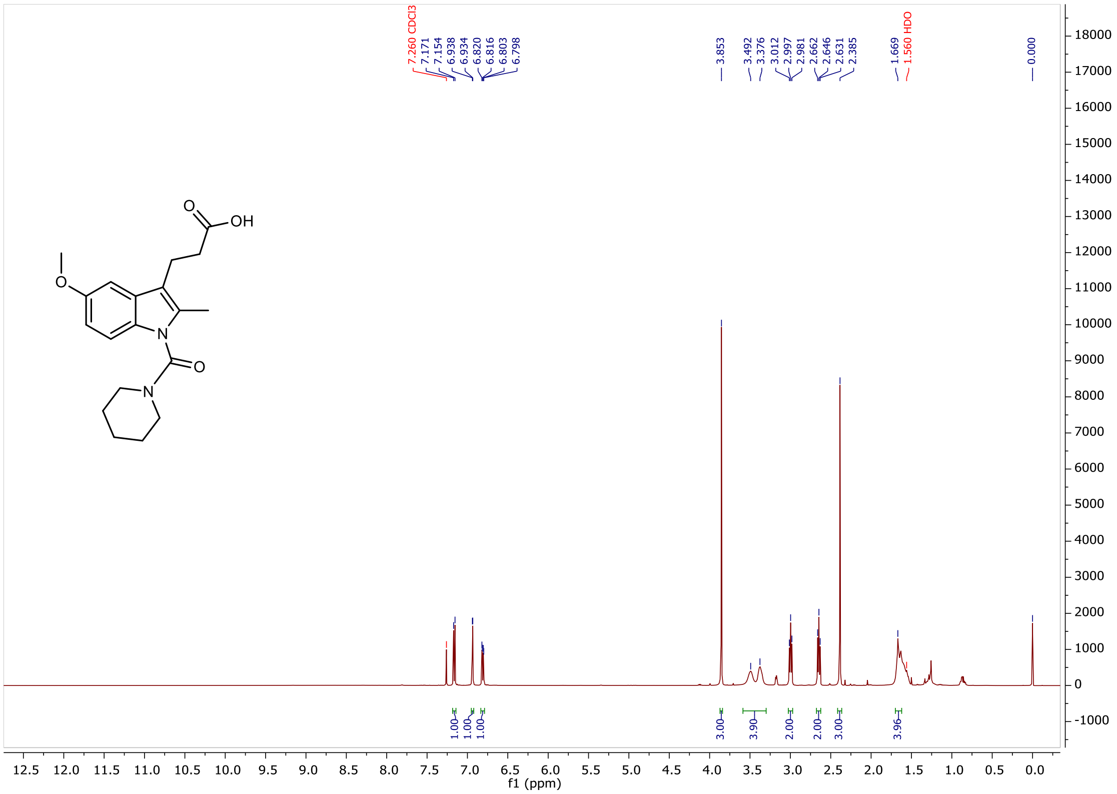

Supplementary Fig. 50. ^{13}C NMR of IA89 (126 MHz, CDCl$_3$)

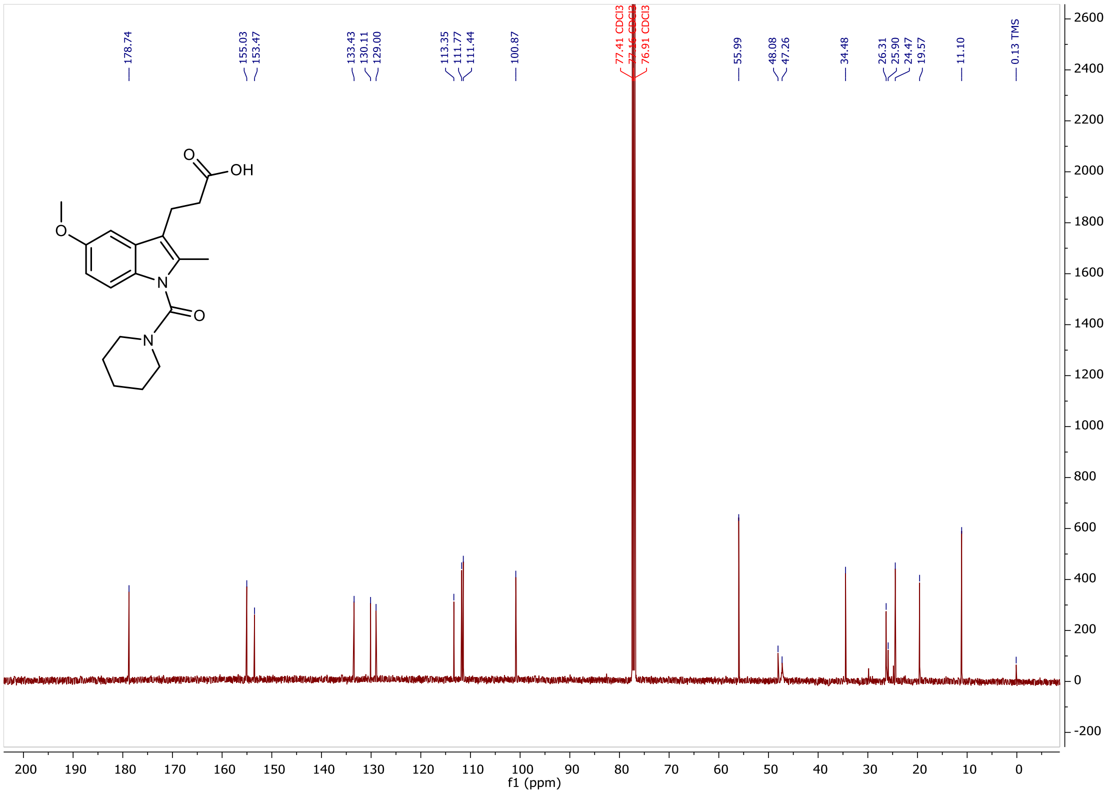

Supplementary Fig. 51. ^{1}H NMR of IA92 (700 MHz, CDCl$_3$)

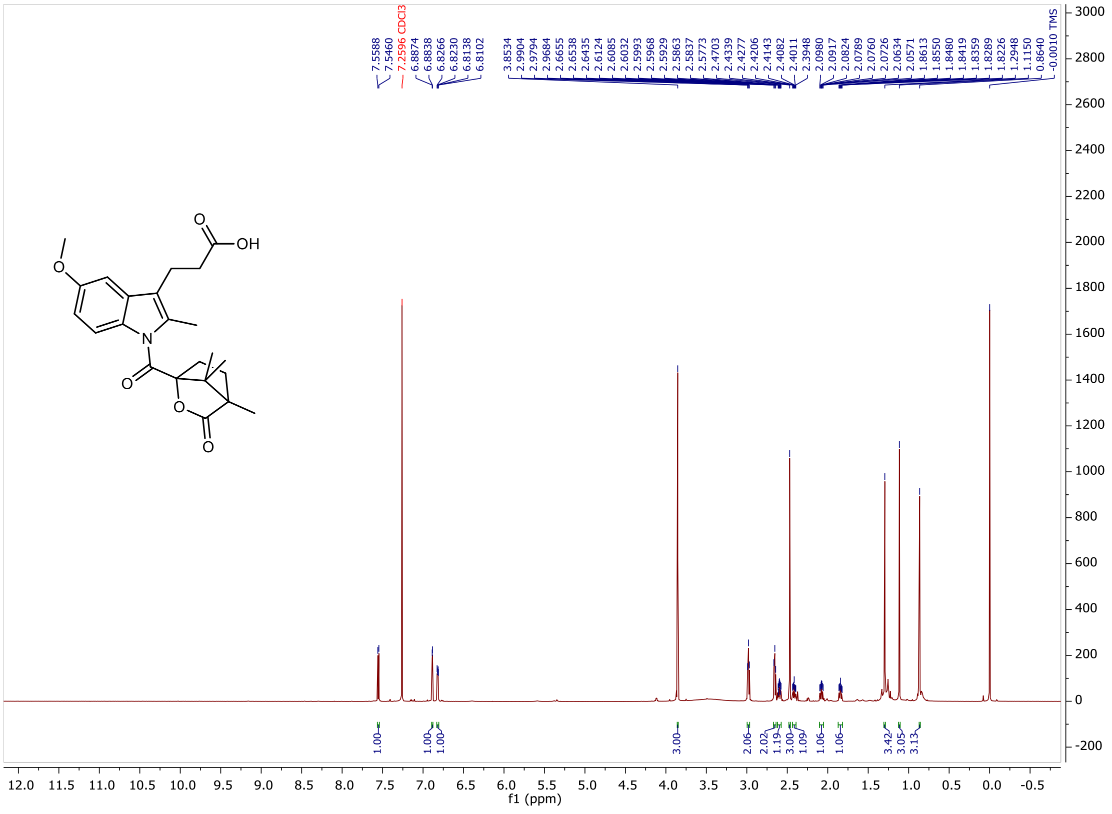

Supplementary Fig. 52. ^{13}C NMR of IA92 (176 MHz, CDCl$_3$)

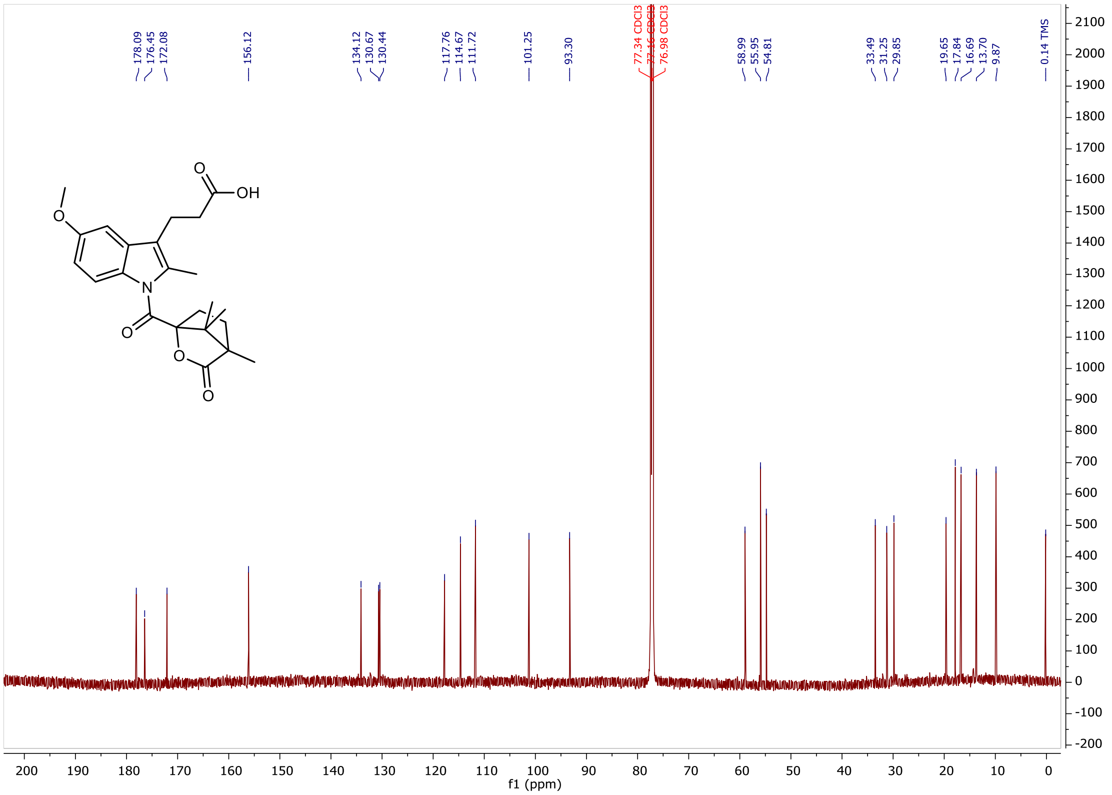

Supplementary Fig. 53. ^{1}H NMR of IA93 (600 MHz, CDCl$_3$)

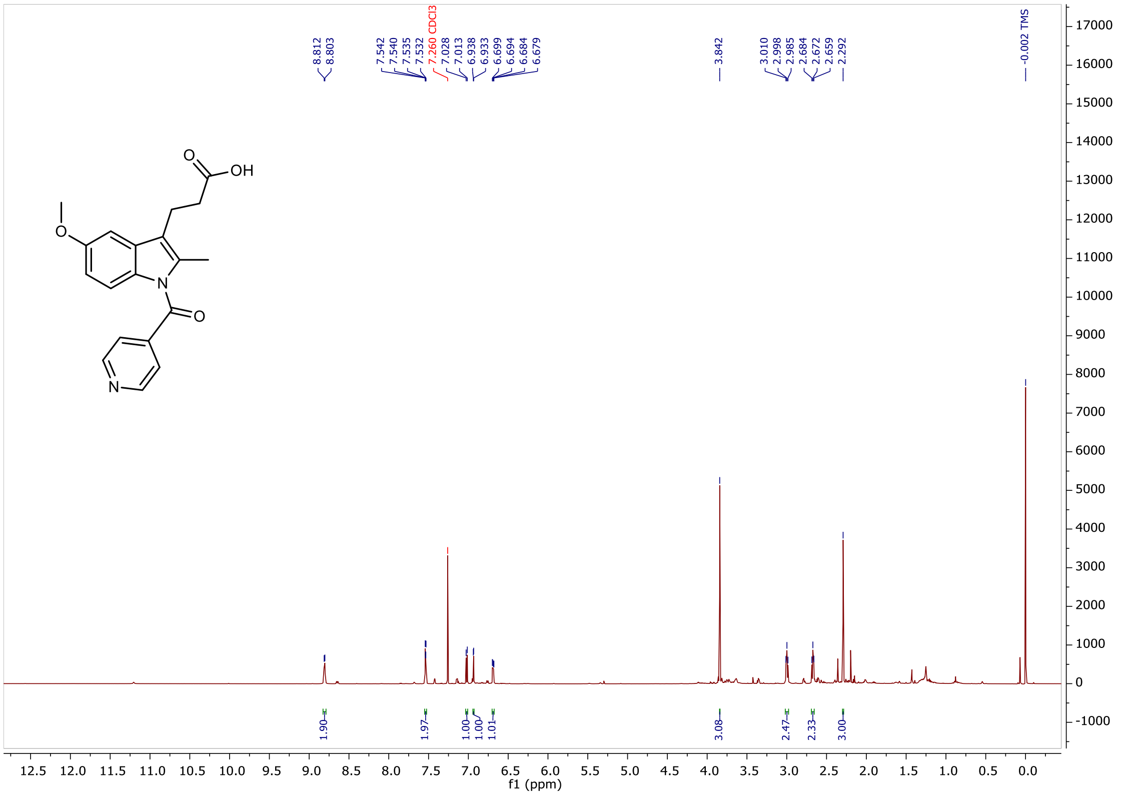

Supplementary Fig. 54. ^{13}C NMR of IA93 (151 MHz, CDCl$_3$)

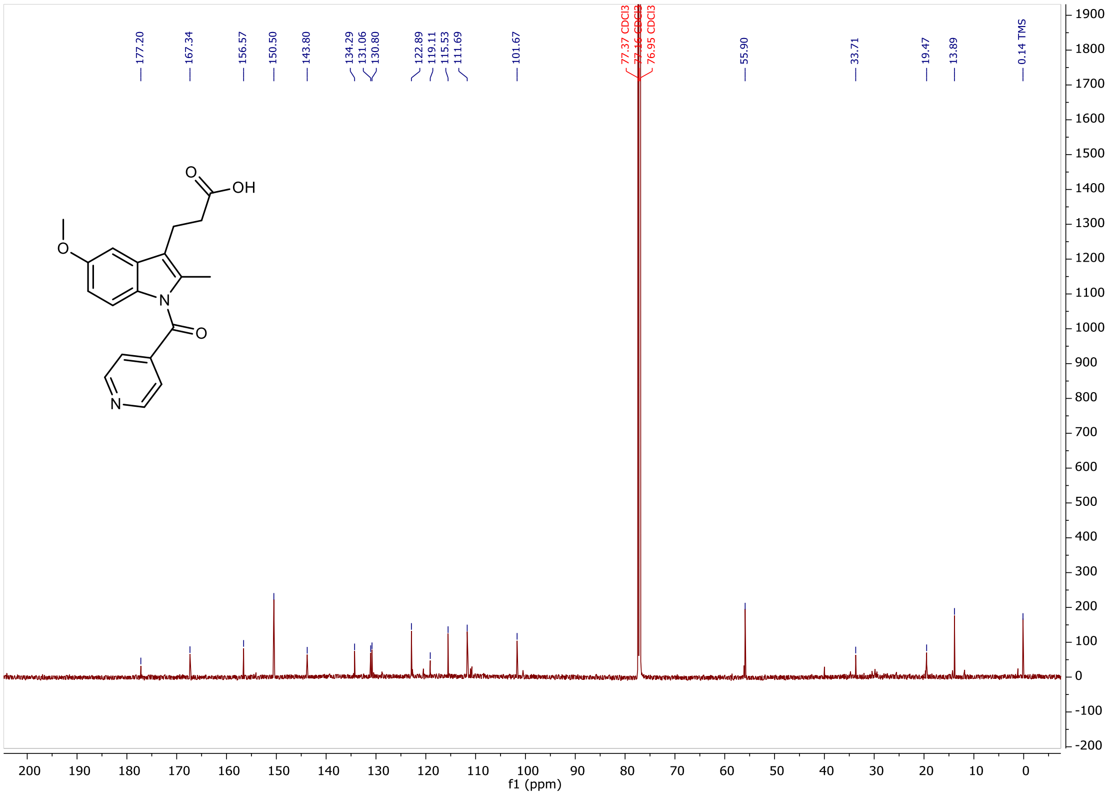

Supplementary Fig. 55. ^{1}H NMR of IA96 (500 MHz, CDCl$_3$)

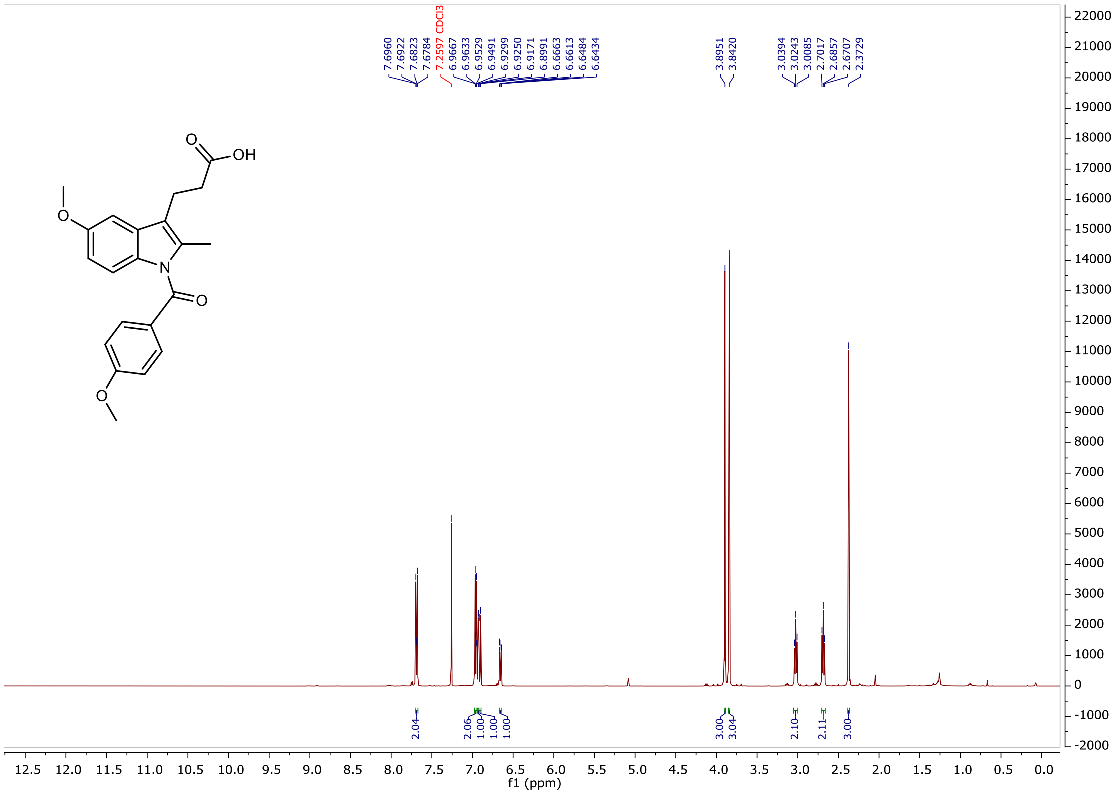

Supplementary Fig. 56. ^{13}C NMR of IA96 (126 MHz, CDCl$_3$)

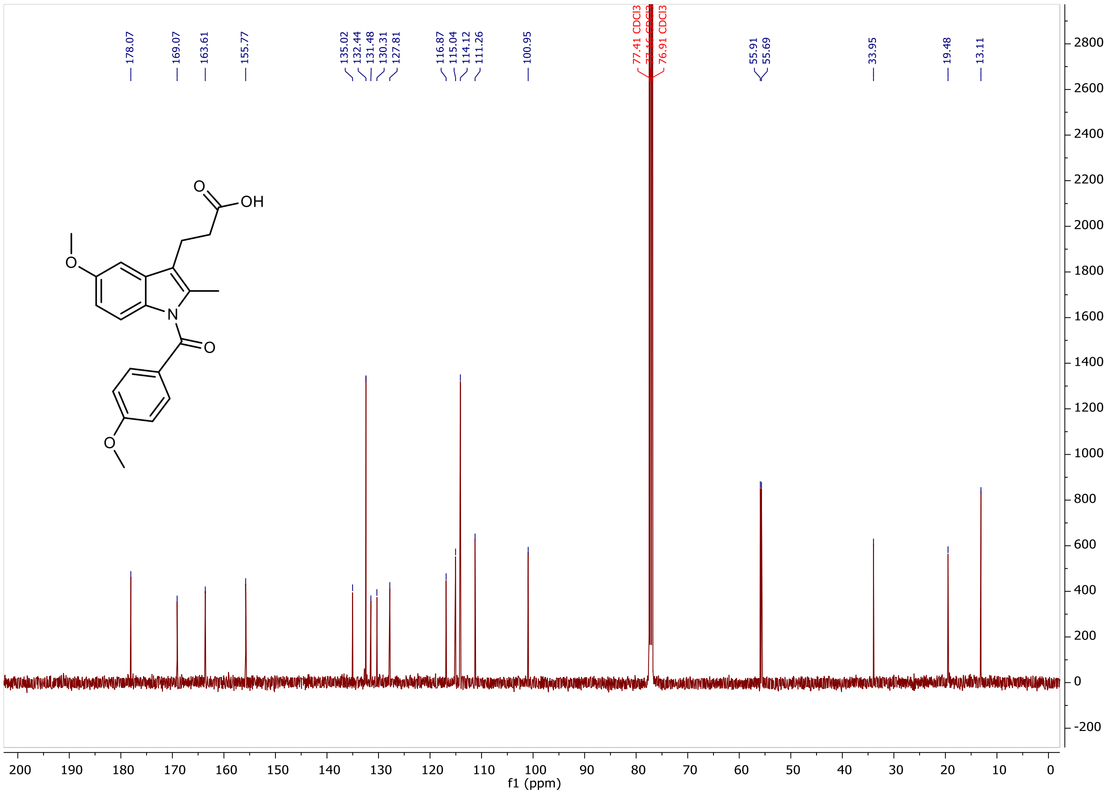

Supplementary Fig. 57. ^{1}H NMR of IA104 (500 MHz, CDCl$_3$)

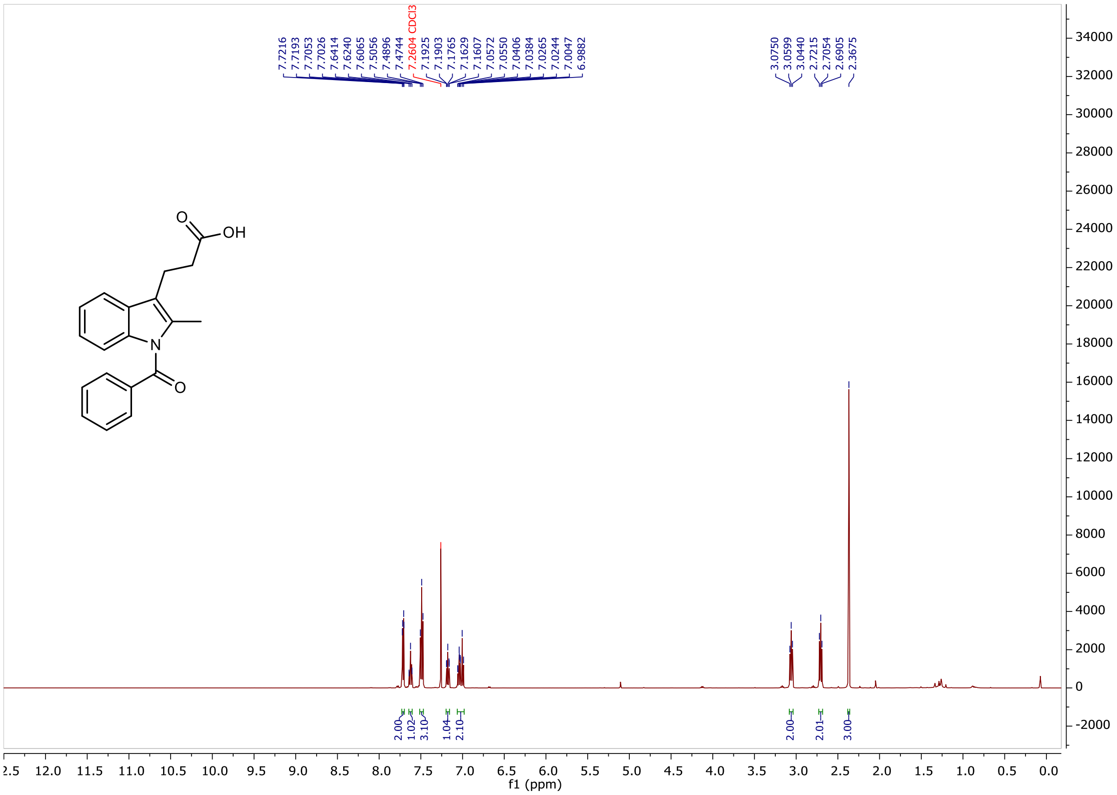

Supplementary Fig. 58. ^{13}C NMR of IA104 (126 MHz, $CDCl_3$)

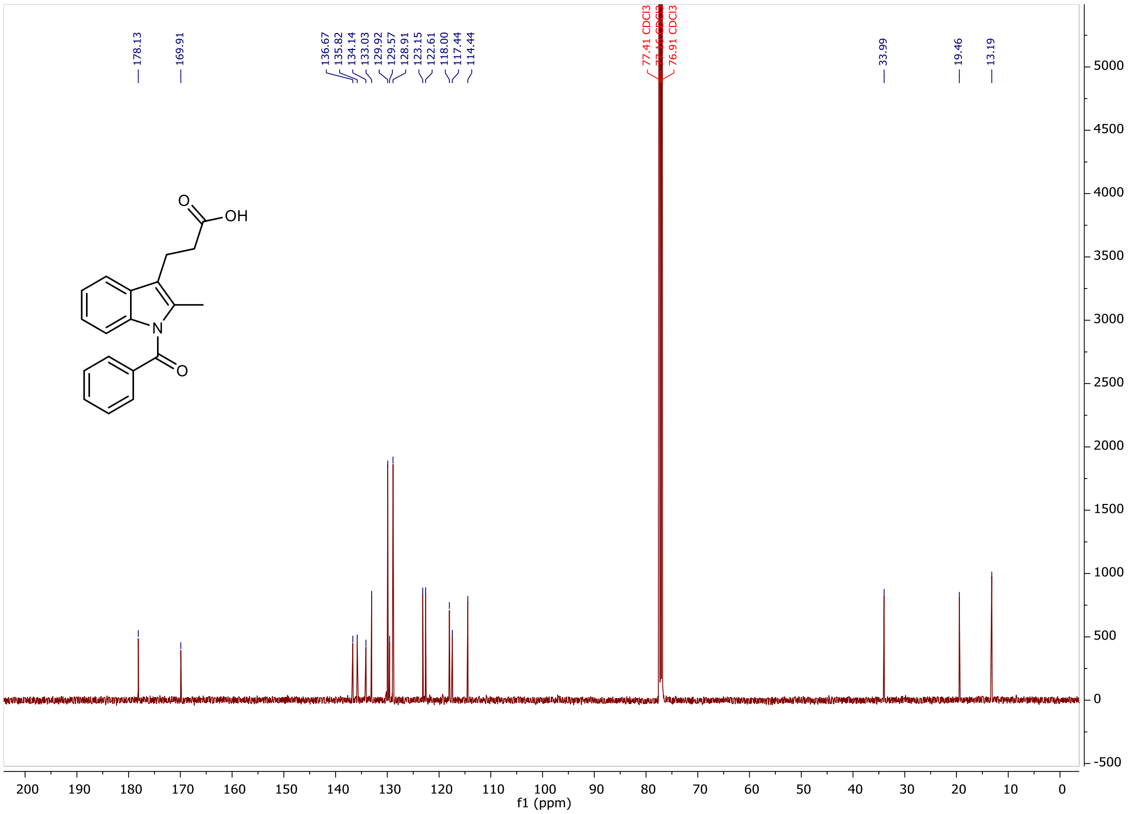

Supplementary Fig. 59. ^{1}H NMR of IA107 (500 MHz, $CDCl_3$)

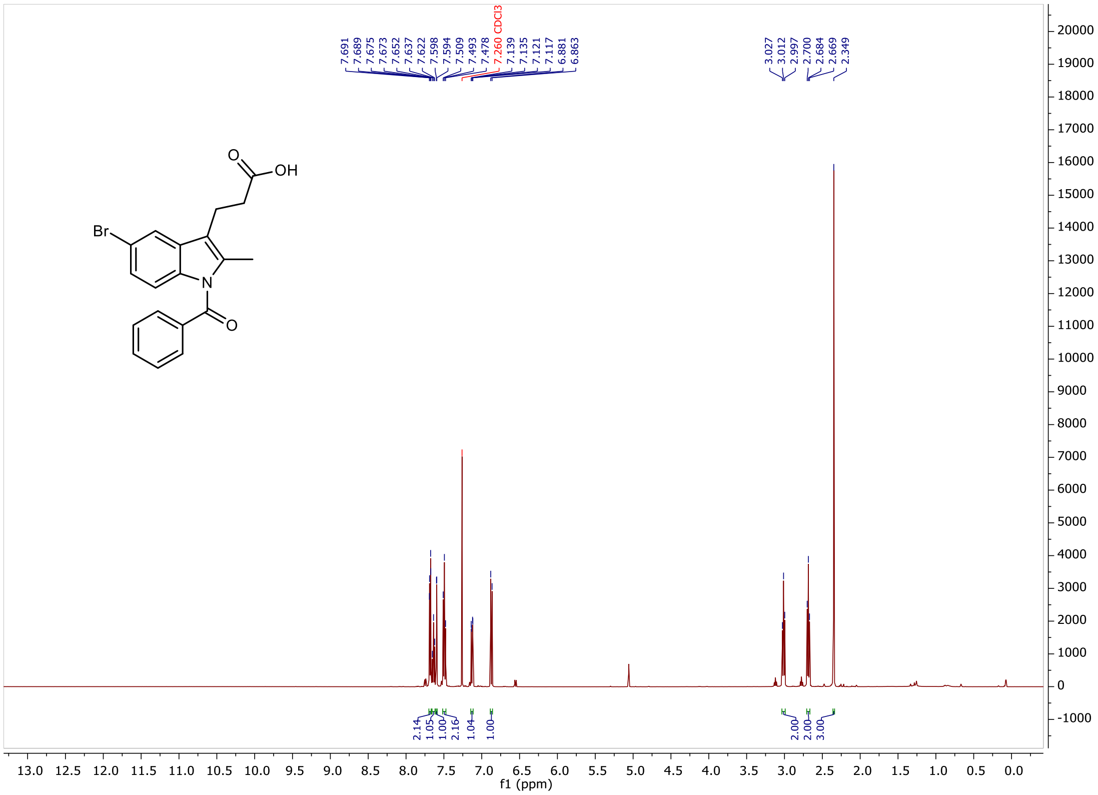

Supplementary Fig. 60. ^{13}C NMR of IA107 (126 MHz, CDCl$_3$)

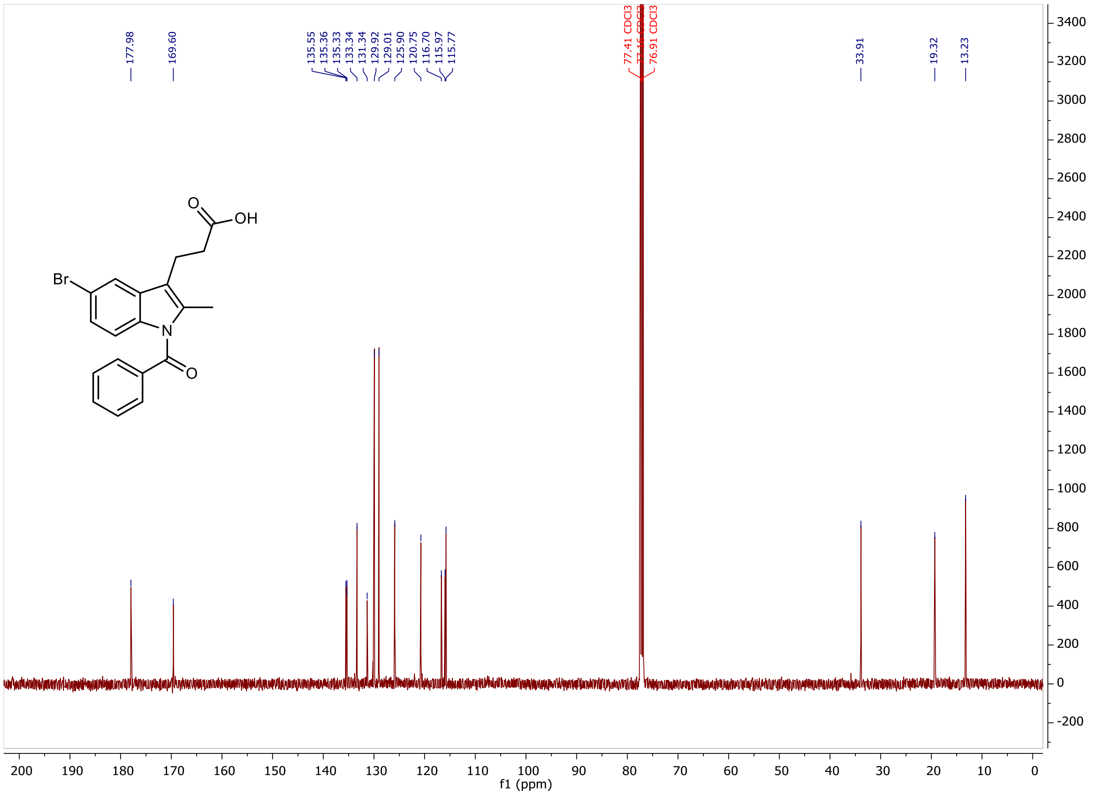

Supplementary Fig. 61. ^{1}H NMR of IA110 (600 MHz, CDCl$_3$)

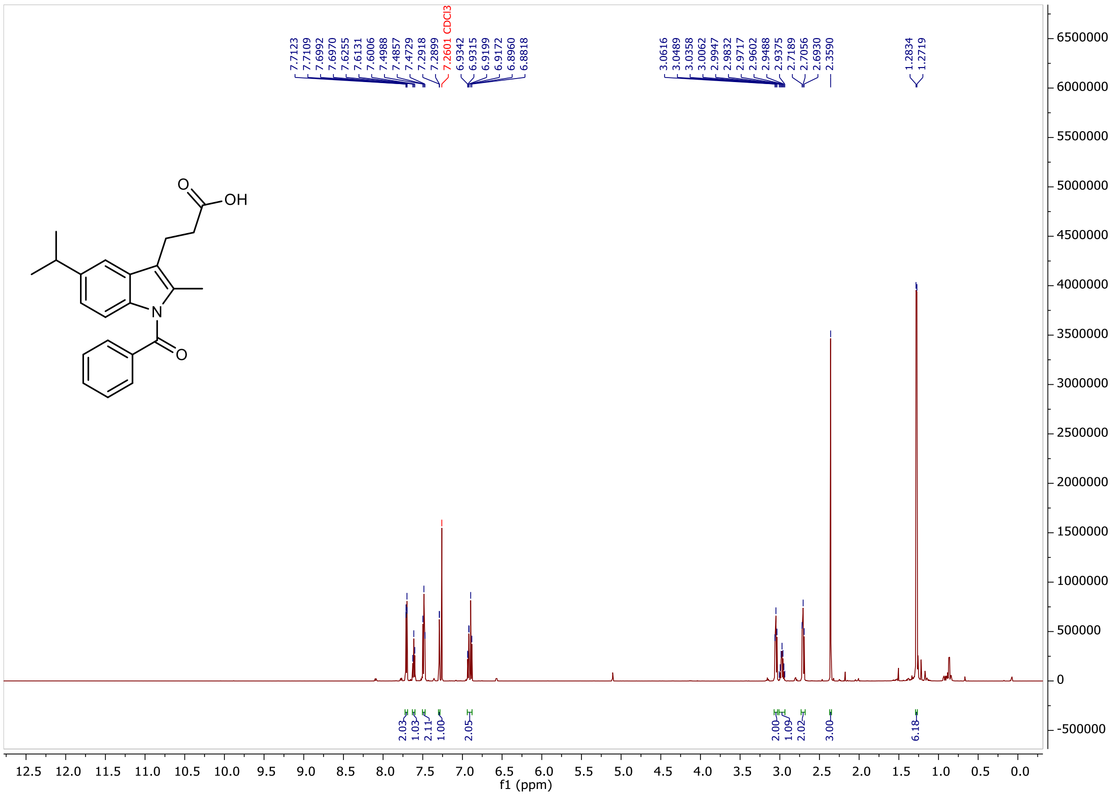

Supplementary Fig. 62. ^{13}**C NMR of IA110 (151 MHz, CDCl$_3$)**

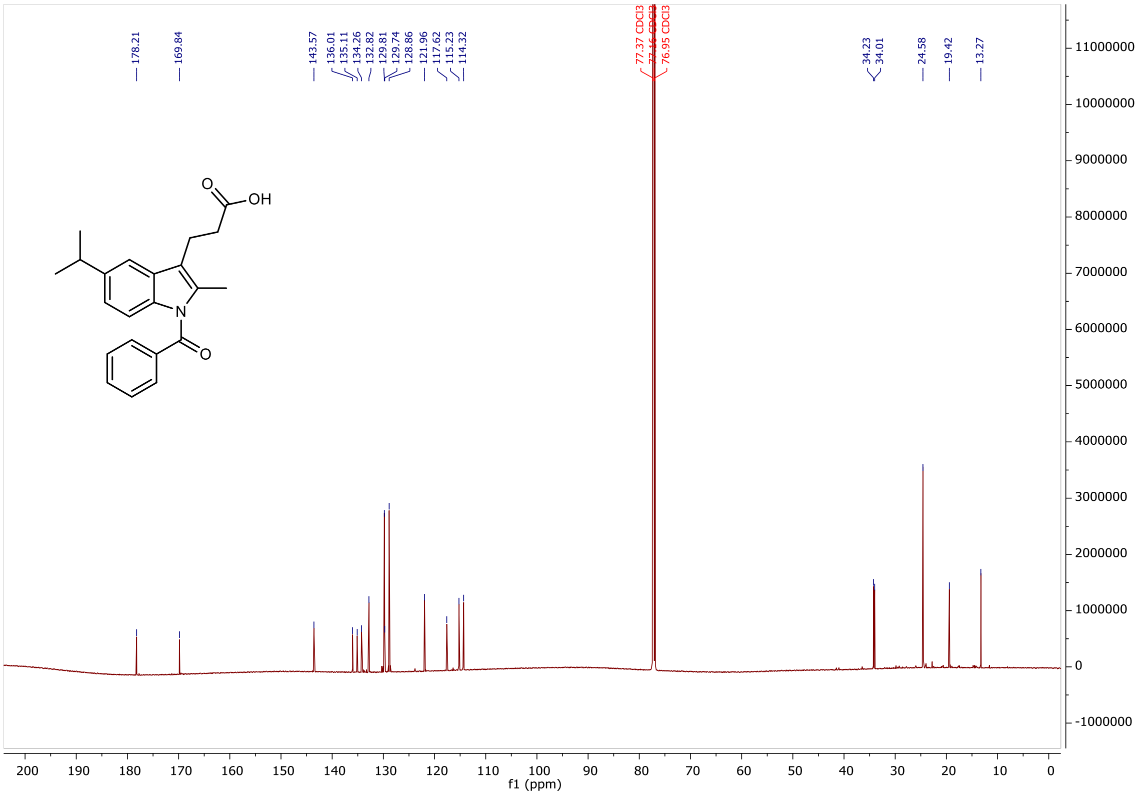

Supplementary Fig. 63. ^{1}**H NMR of IA137 (500 MHz, CDCl$_3$)**

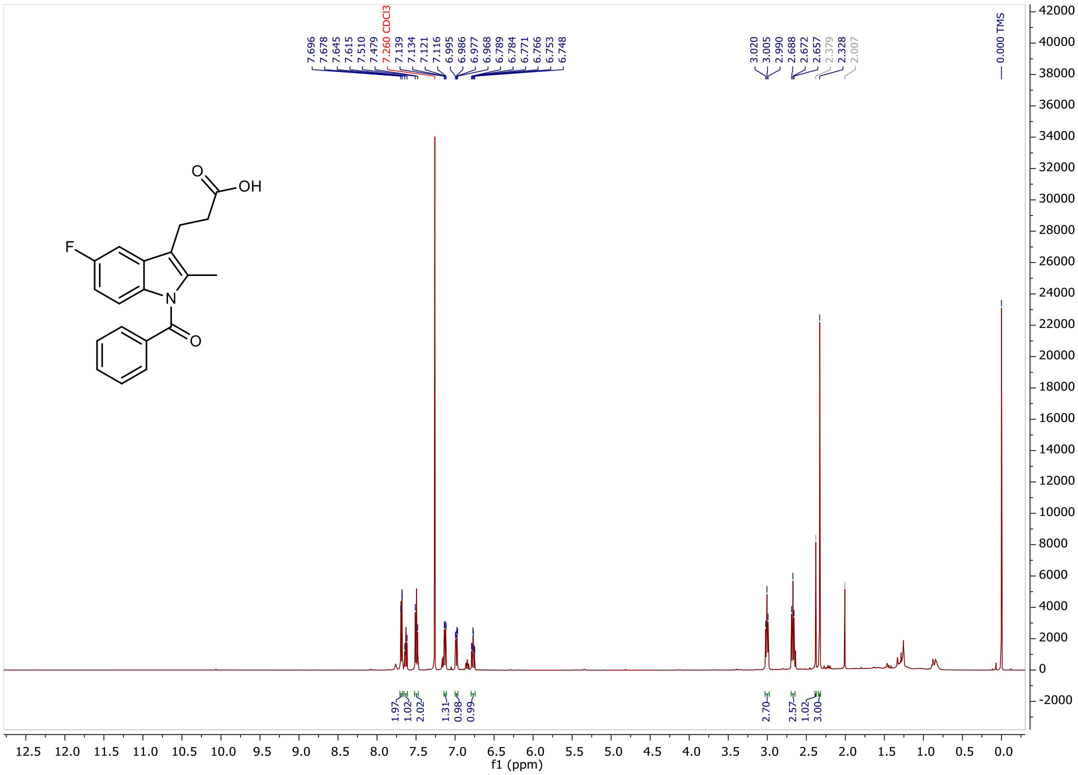

Supplementary Fig. 64. ^{13}C NMR of IA137 (126 MHz, $CDCl_3$)

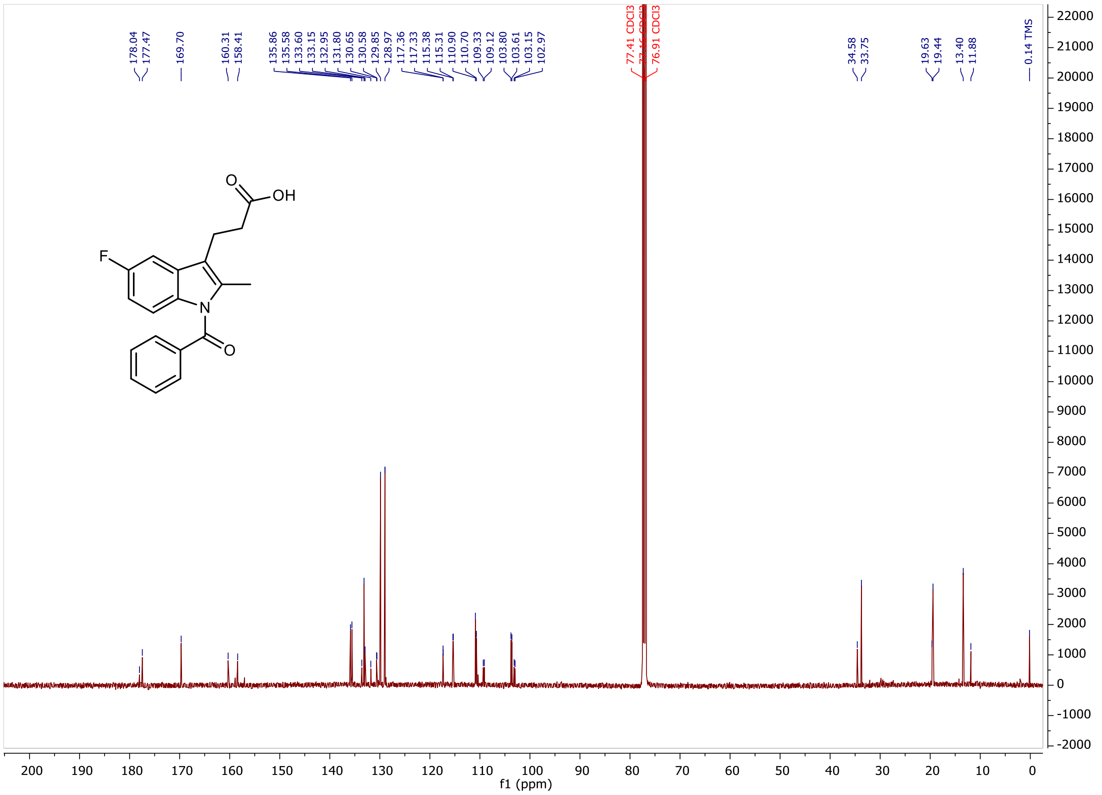

Supplementary Fig. 65. ^{19}F NMR of IA137 (470 MHz, $CDCl_3$)

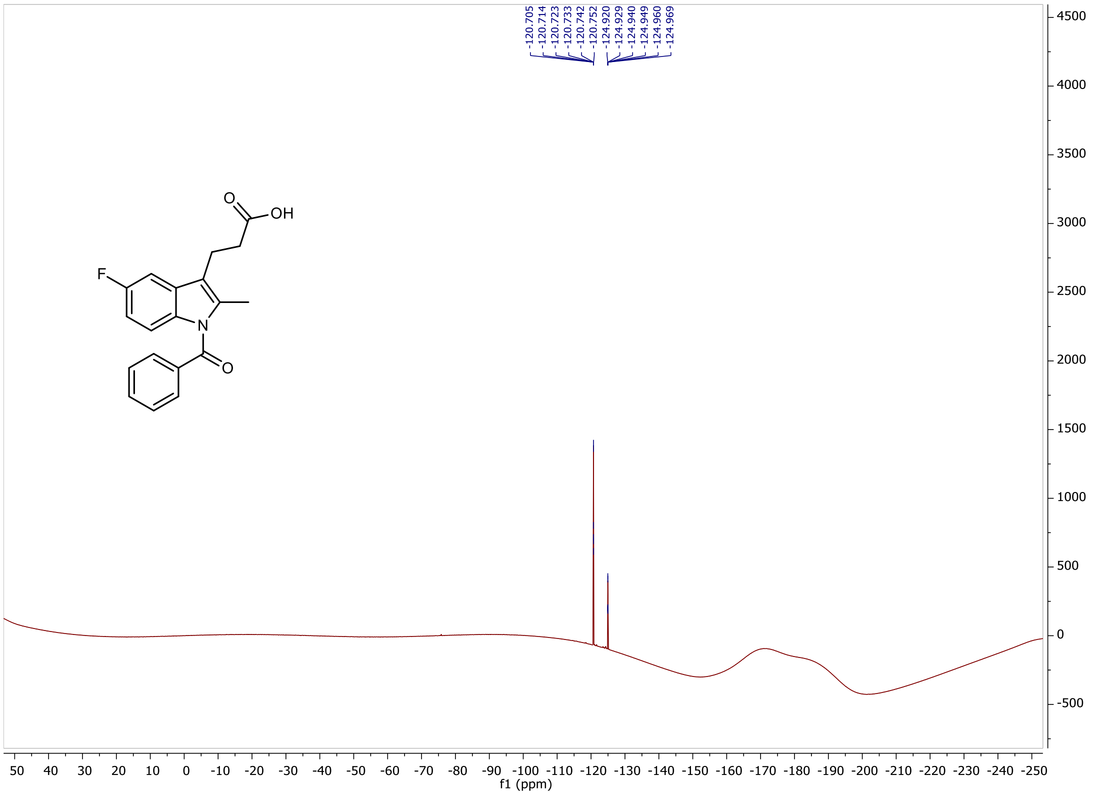

Supplementary Fig. 66. ^{1}H NMR of IA138 (700 MHz, $CDCl_3$)

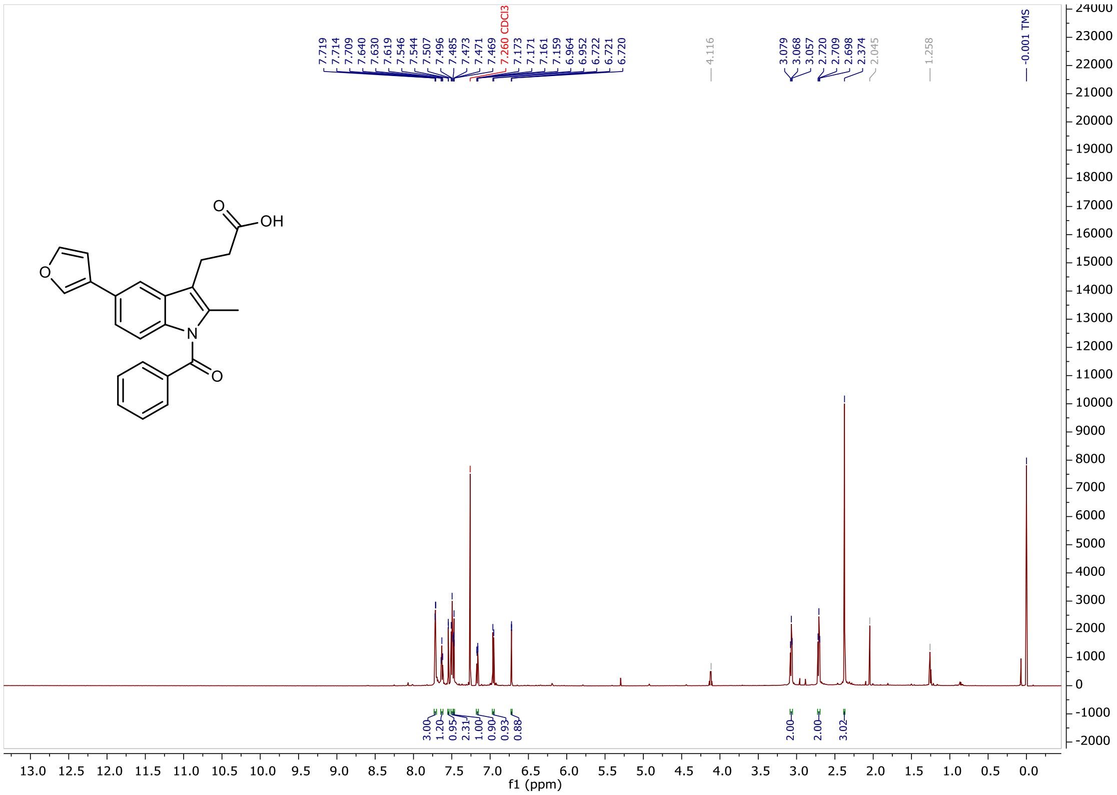

Supplementary Fig. 67. ^{13}C NMR of IA138 (176 MHz, $CDCl_3$)

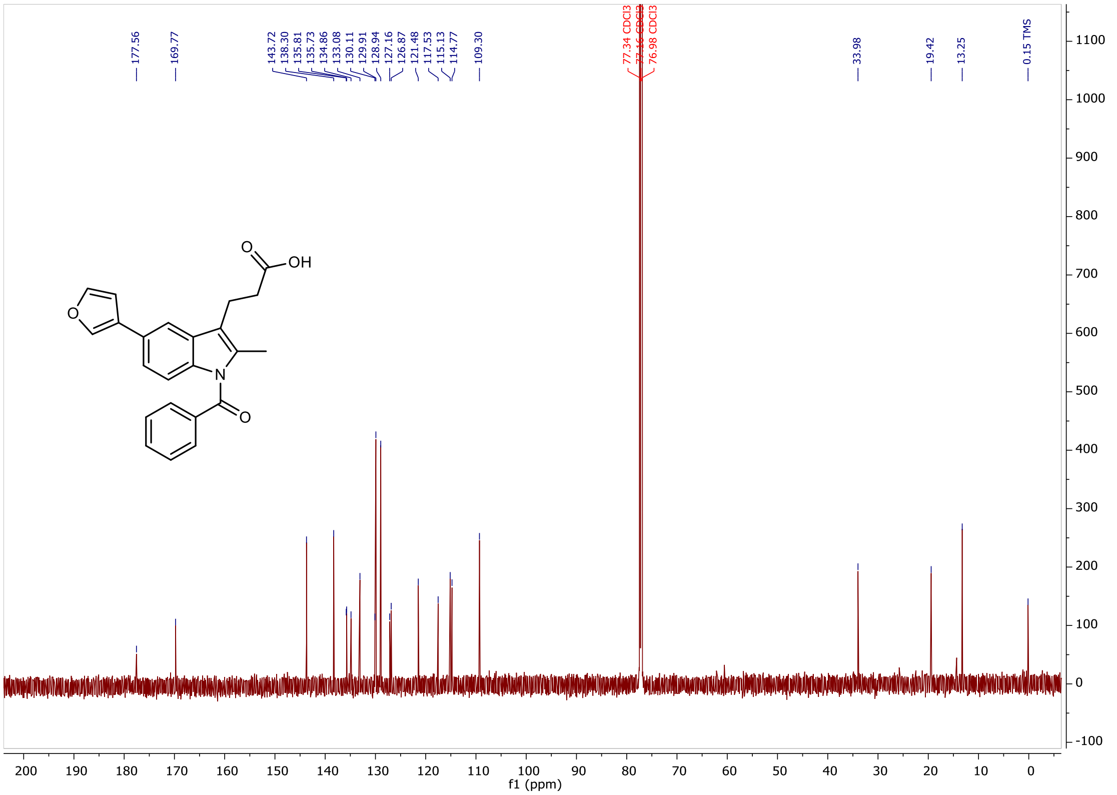

Supplementary Fig. 68. ^{1}H NMR of IA140 (700 MHz, $CDCl_3$)

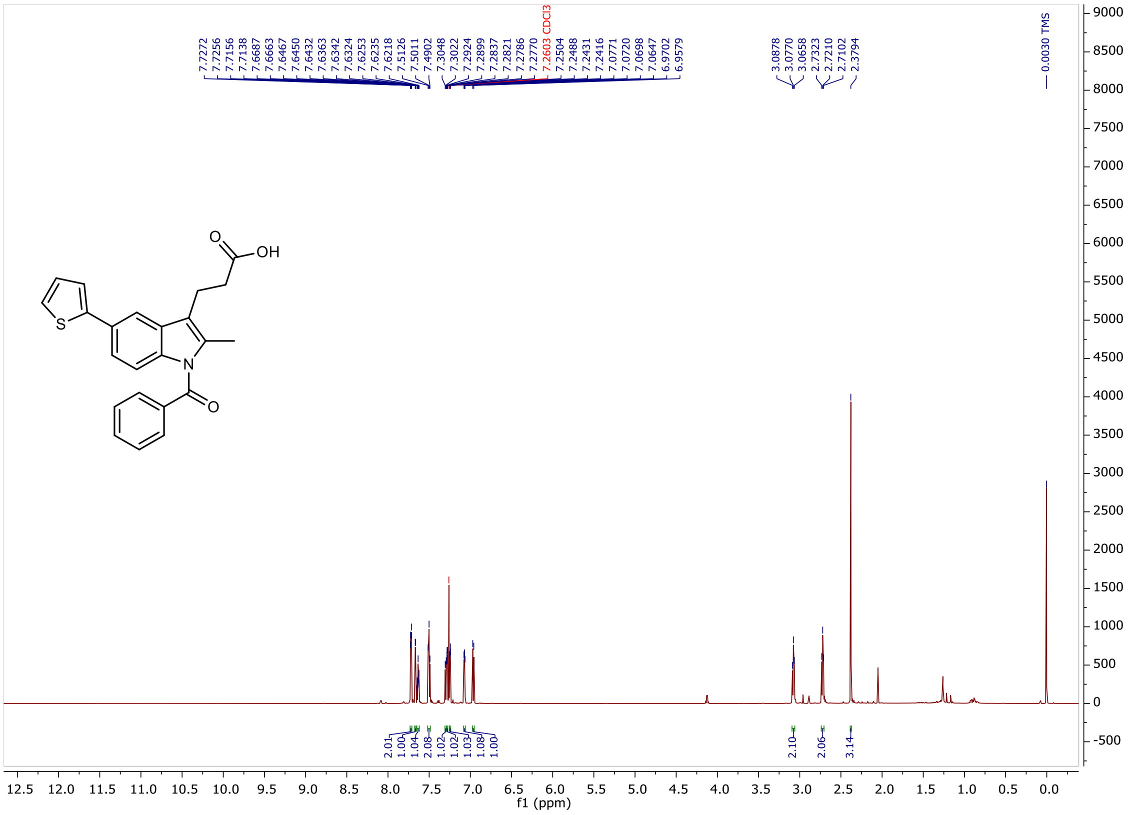

Supplementary Fig. 69. ^{13}C NMR of IA140 (176 MHz, $CDCl_3$)

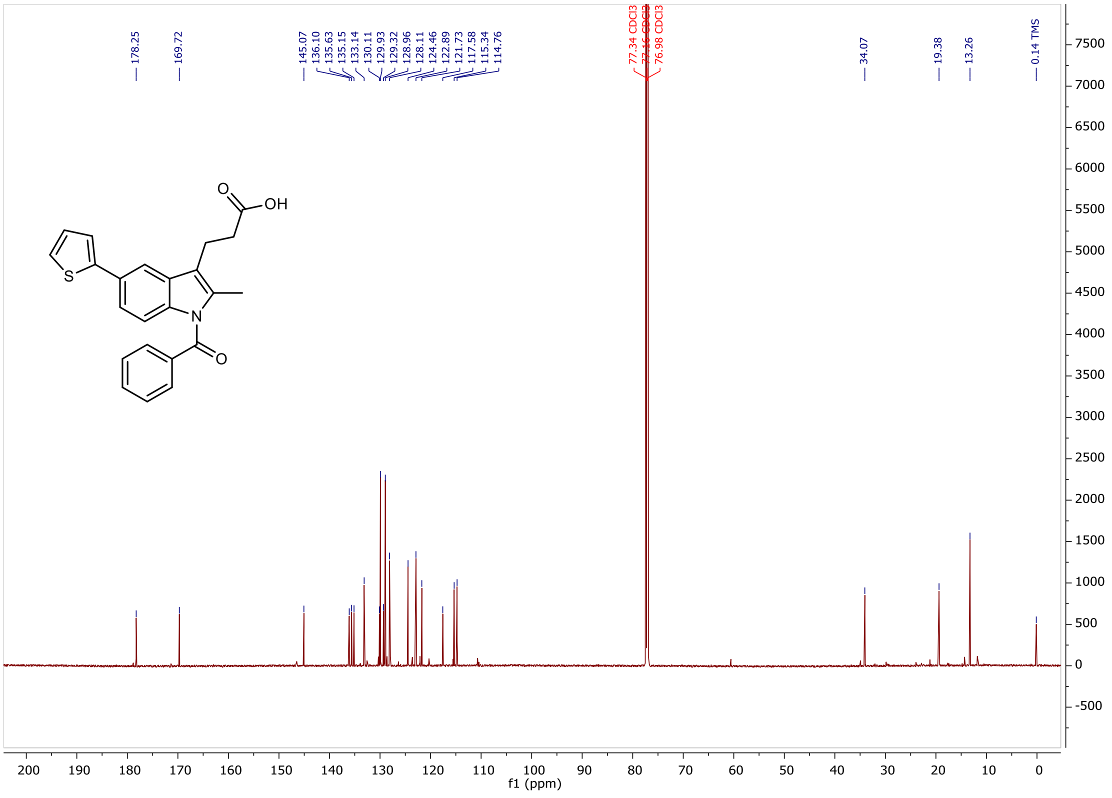

Supplementary Fig. 70. ^{1}H NMR of 141 (500 MHz, $CDCl_3$)

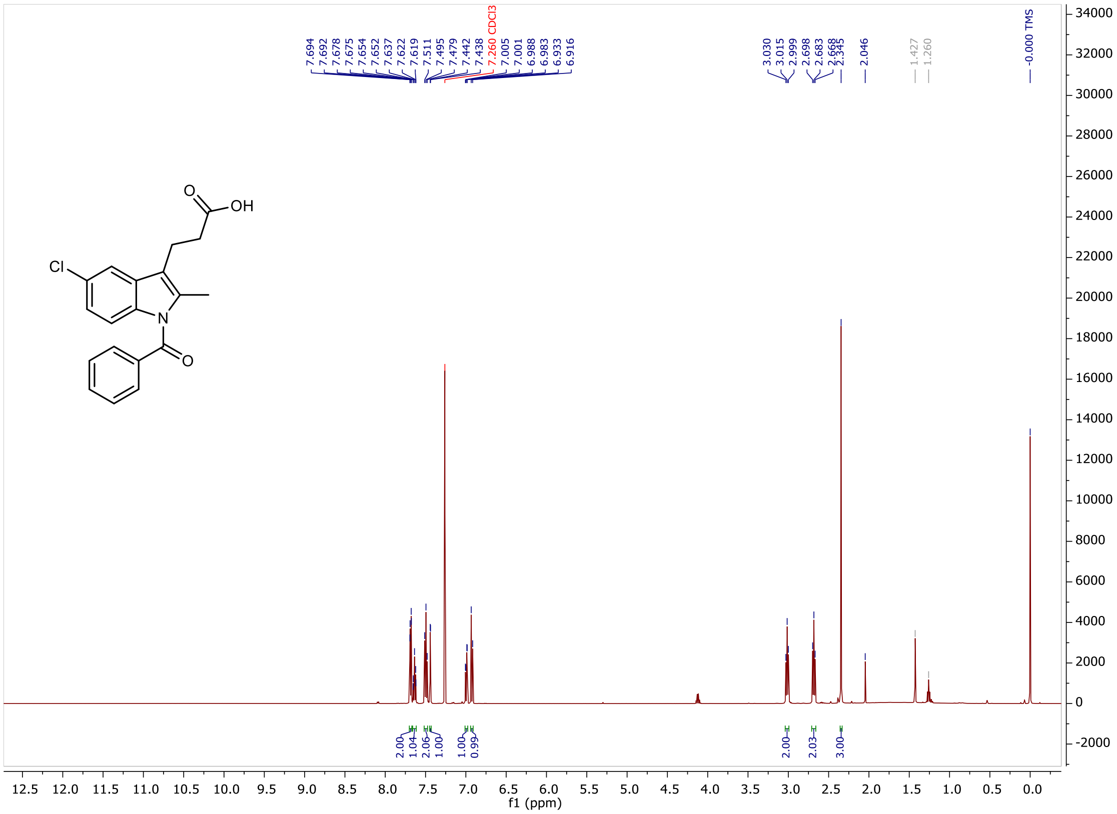

Supplementary Fig. 71. ^{13}C NMR of IA141 (126 MHz, $CDCl_3$)

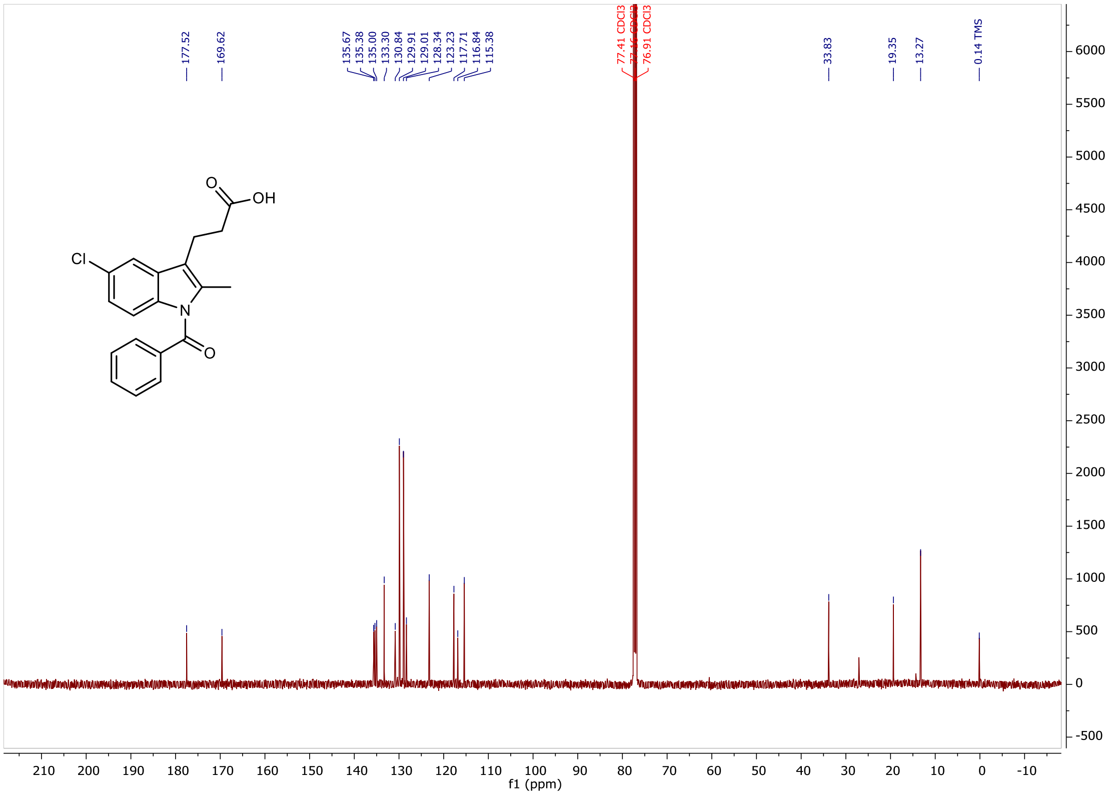

Supplementary Fig. 72. ^{1}**H NMR of 142 (500 MHz, CDCl$_3$)**

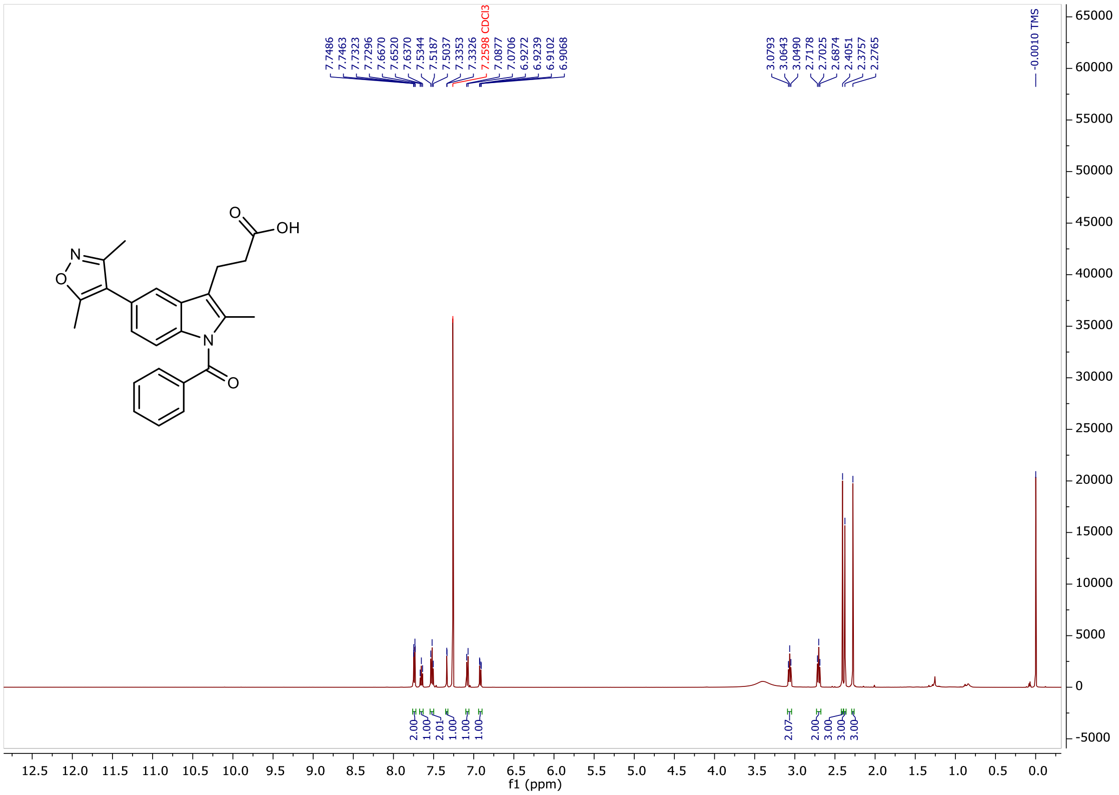

Supplementary Fig. 73. ^{13}**C NMR of IA142 (126 MHz, CDCl$_3$)**

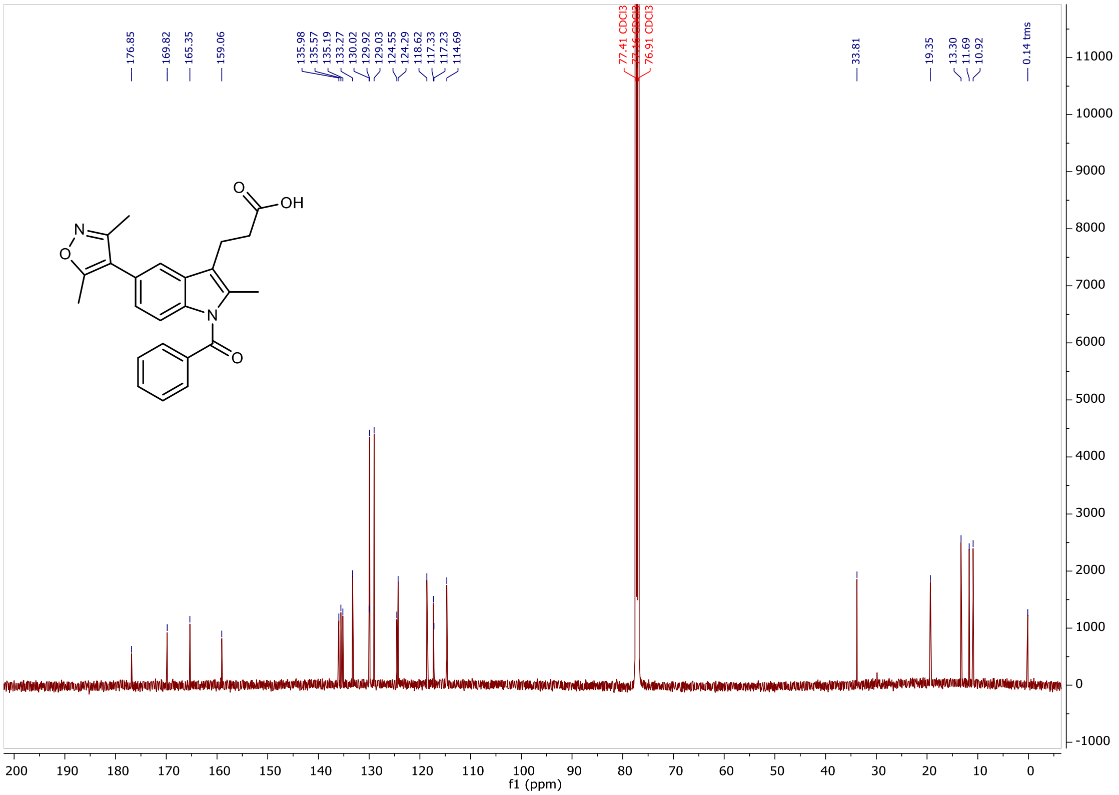

Supplementary Fig. 74. ^{1}H NMR of 143 (700 MHz, $CDCl_3$)

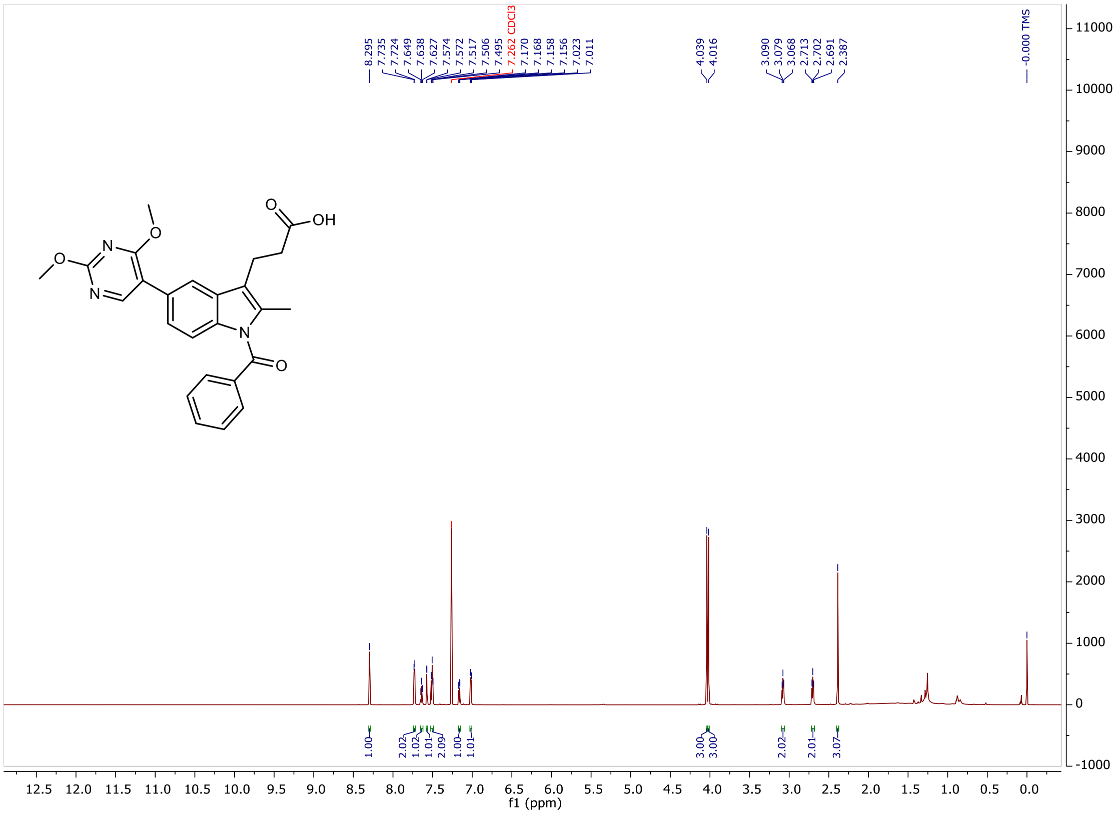

Supplementary Fig. 75. ^{13}C NMR of IA143 (176 MHz, $CDCl_3$)

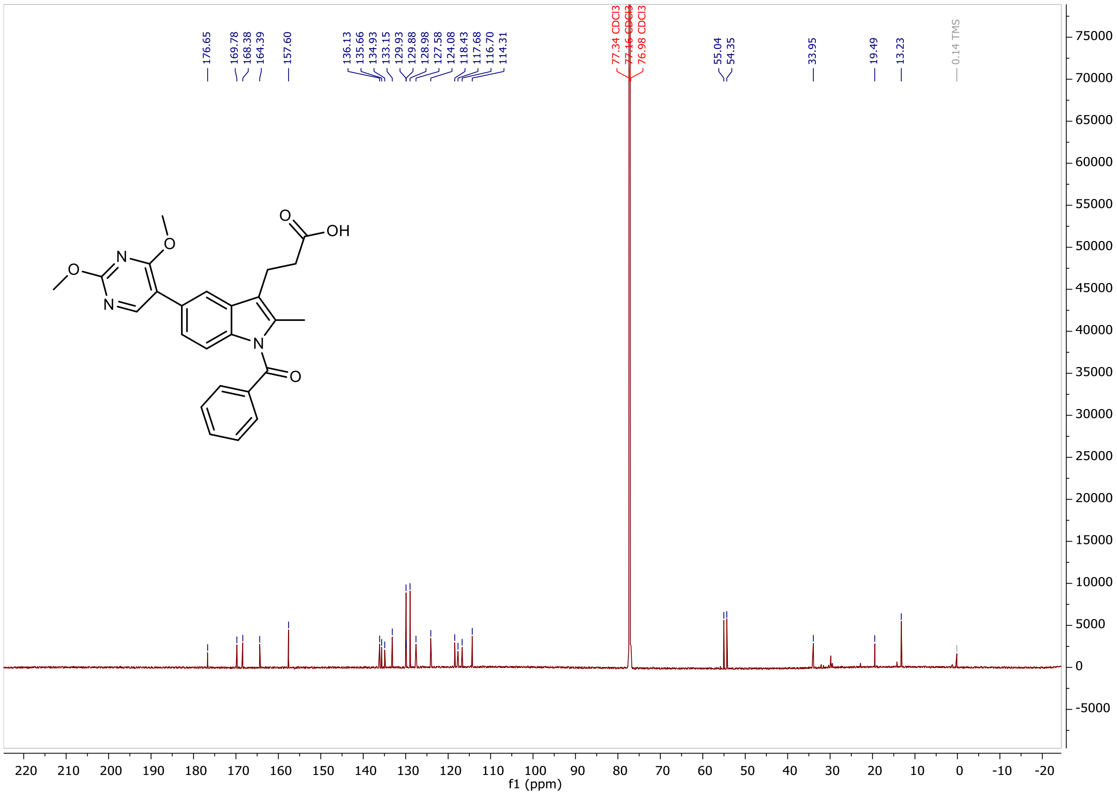

Supplementary Fig. 76. ¹H NMR of 145 (500Mz, CDCl₃)

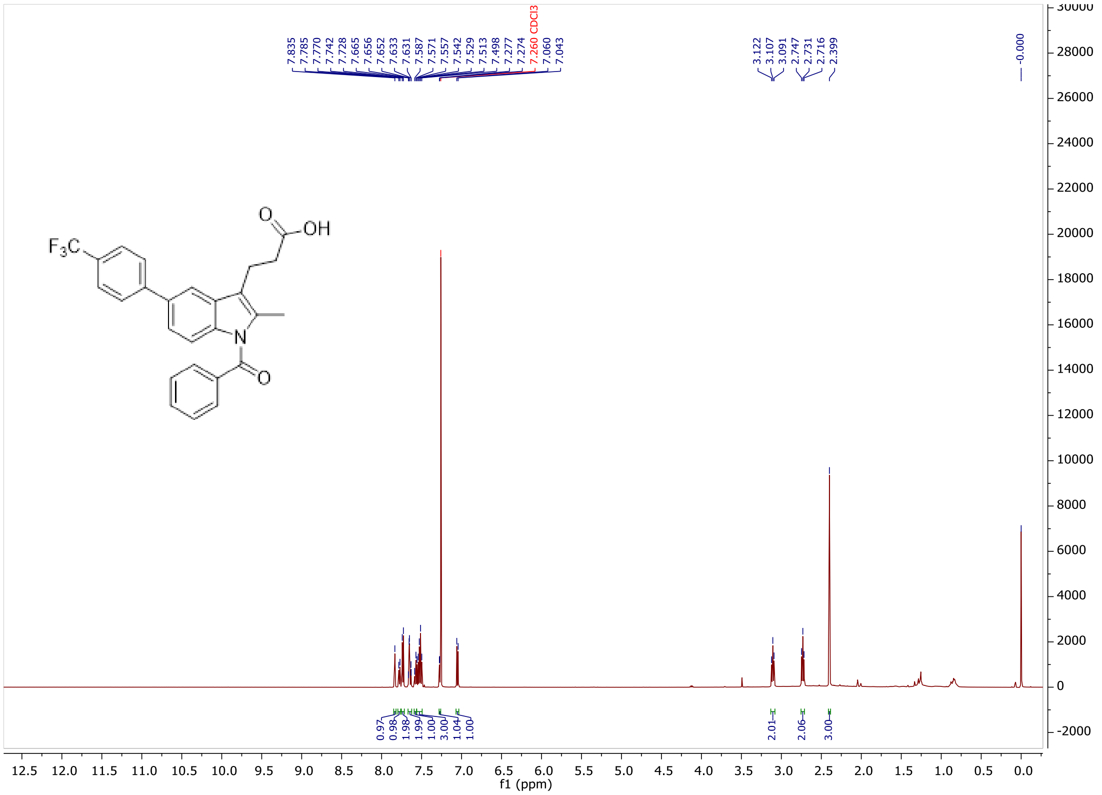

Supplementary Fig. 77. ¹³C NMR of IA145 (126 MHz, CDCl₃)

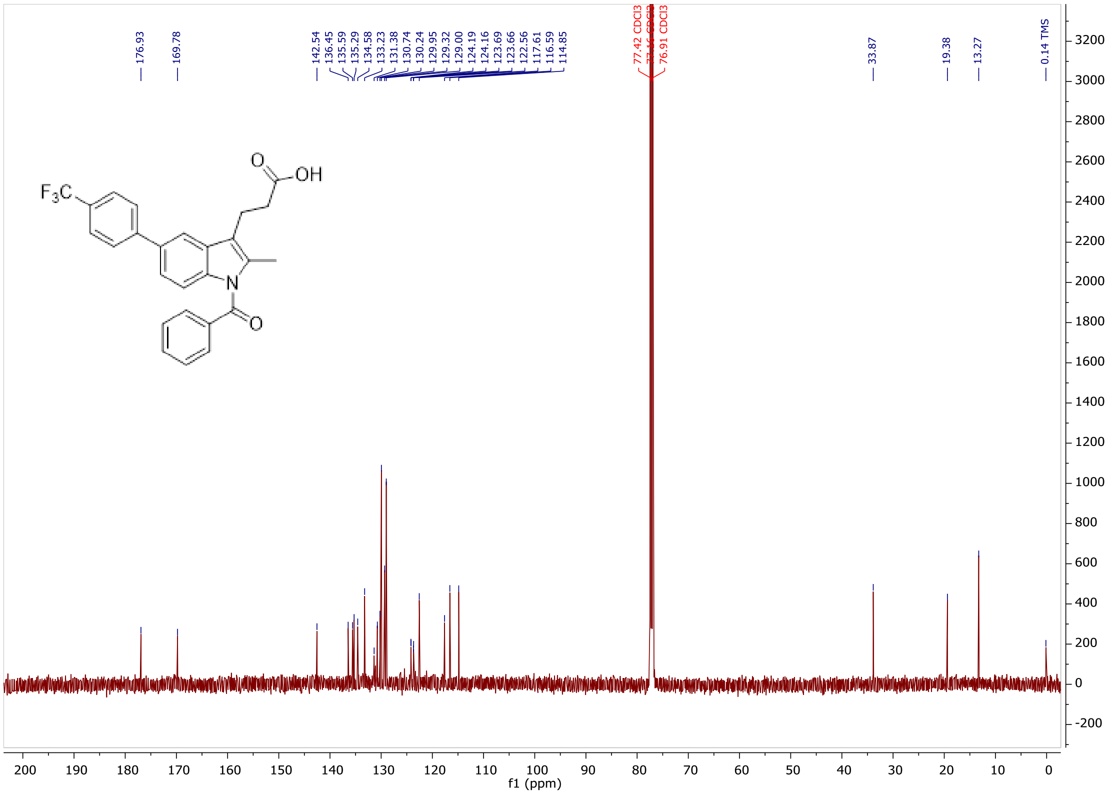

Supplementary Fig. 78. ^{19}F NMR of IA145 (470 MHz, $CDCl_3$)

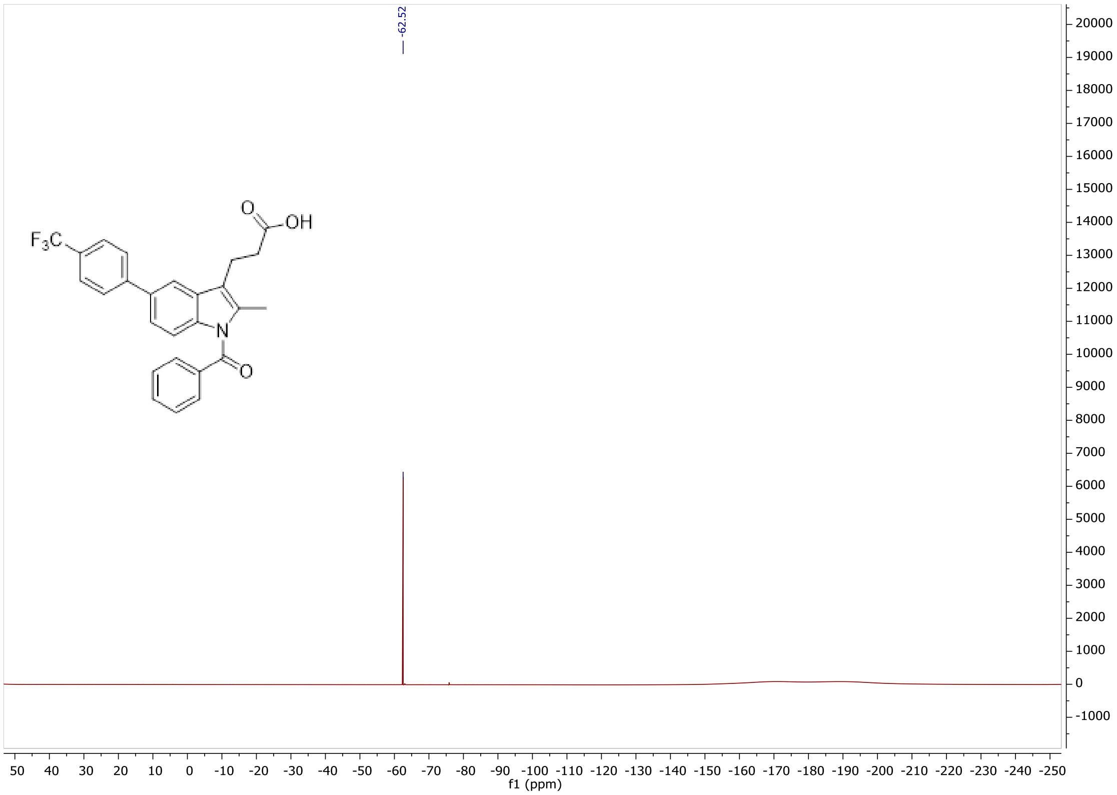

Supplementary Fig. 79. ^{1}H NMR of 146 (700 MHz, $CDCl_3$)

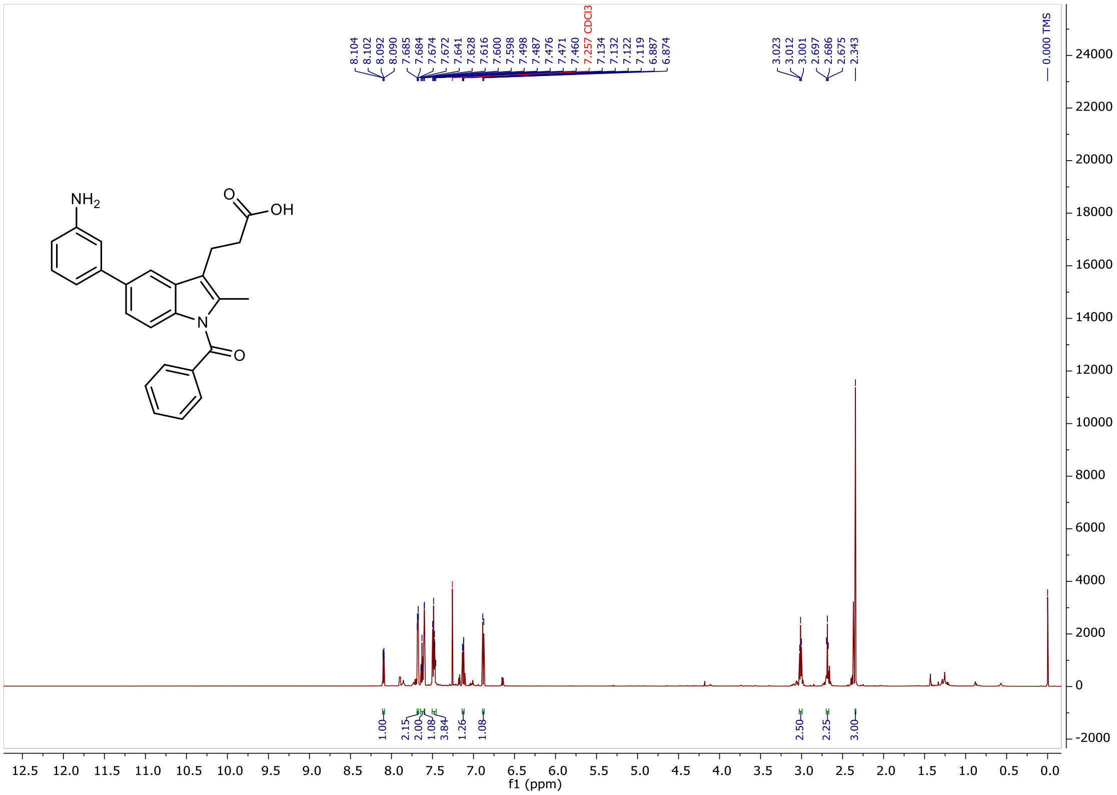

Supplementary Fig. 80. ^{13}C NMR of IA146 (176 MHz, $CDCl_3$)

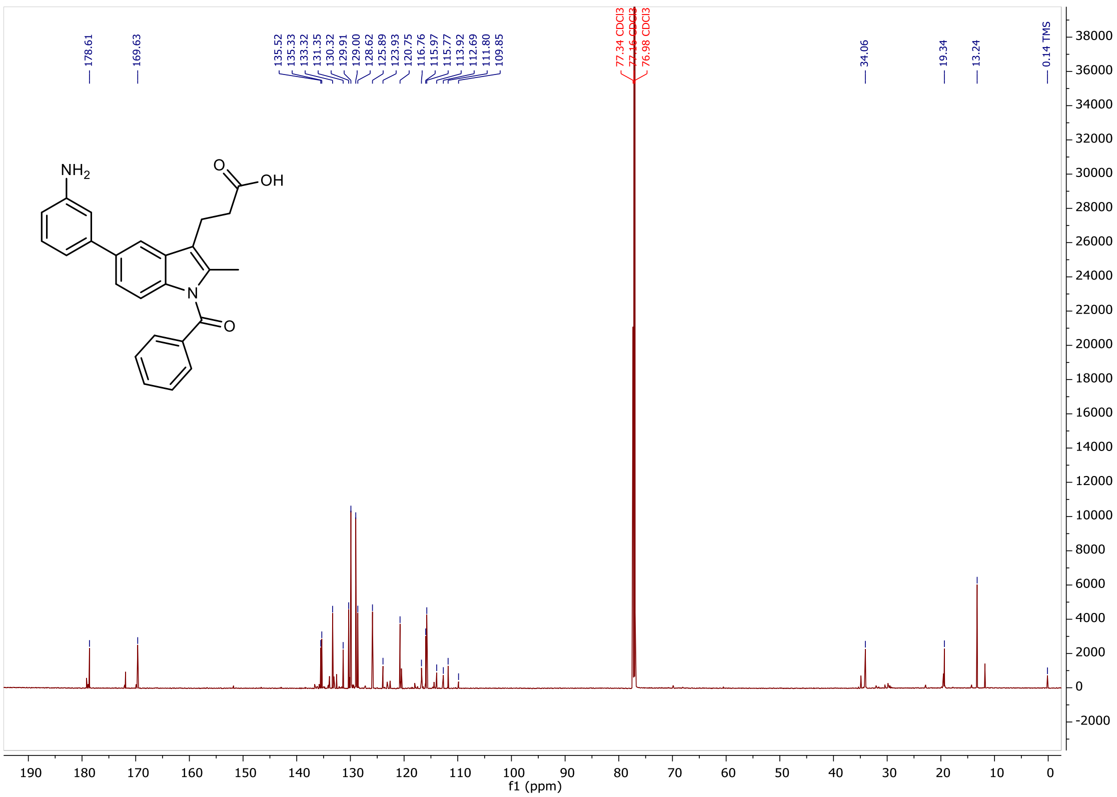

Supplementary Fig. 81. ^{1}H NMR of IA147 (500Mz, $CDCl_3$)

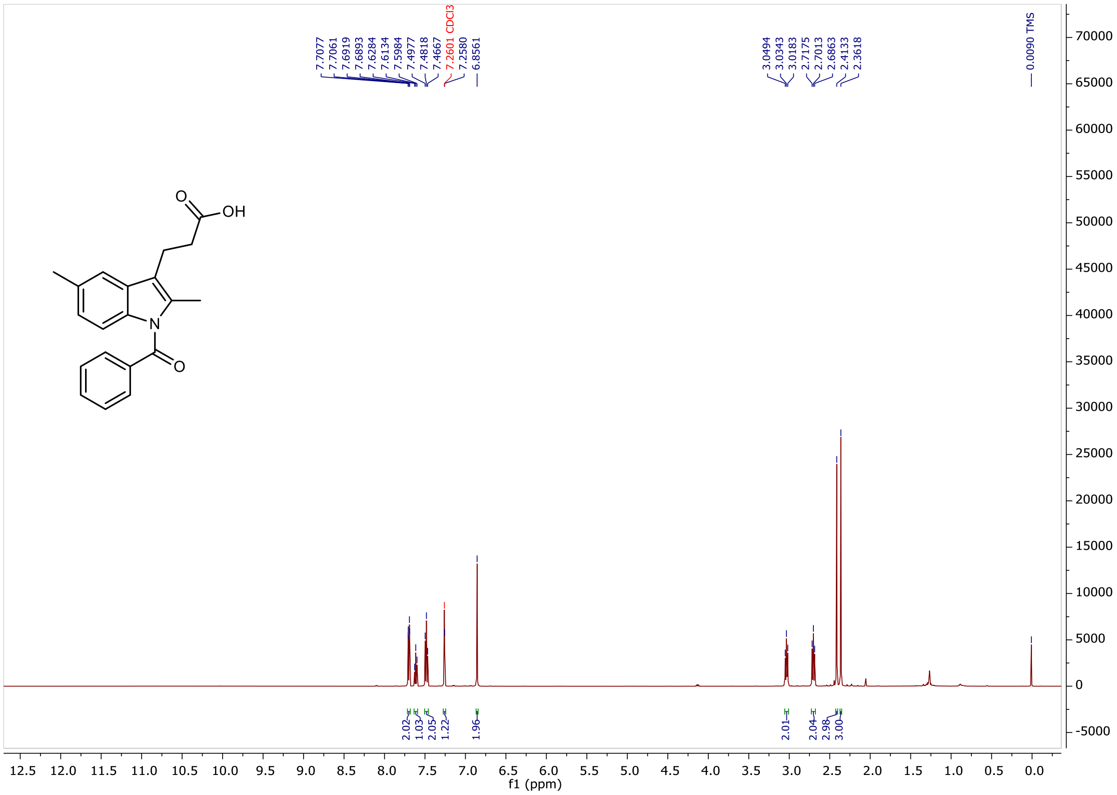

Supplementary Fig. 82. ^{13}**C NMR of IA147 (126 MHz, CDCl₃)**

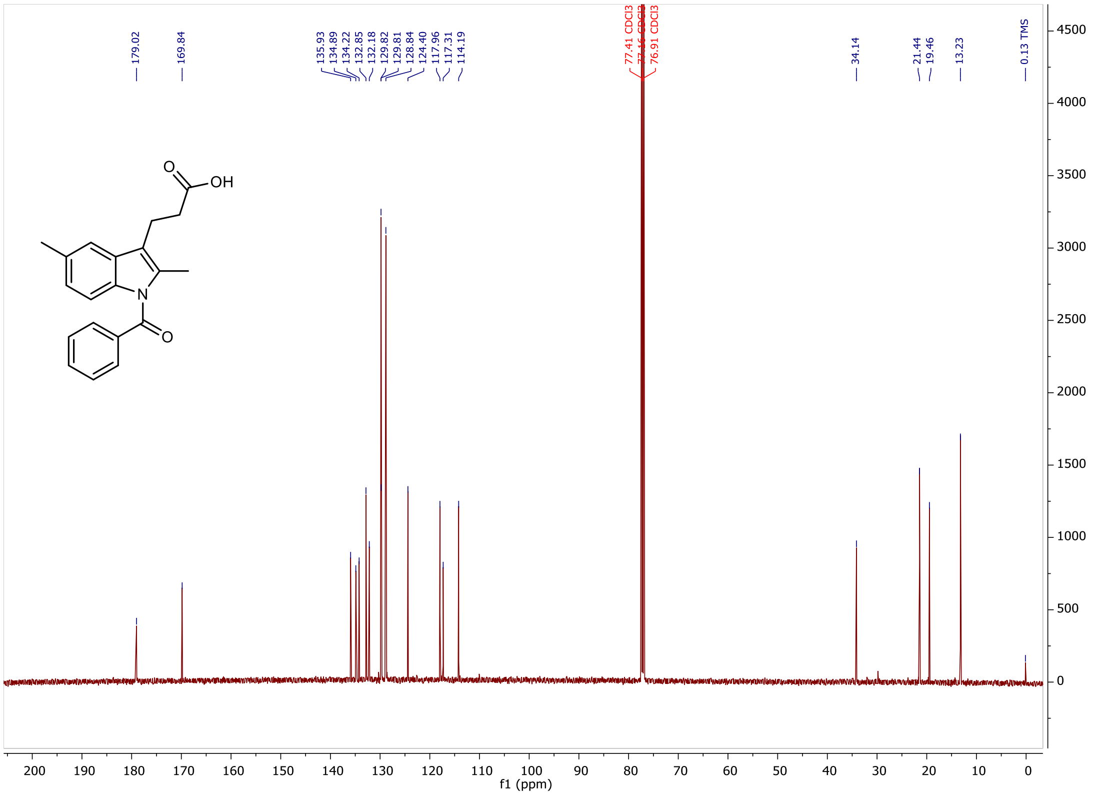

Supplementary Fig. 83. ^{1}**H NMR of IA148 (700 MHz, CDCl₃)**

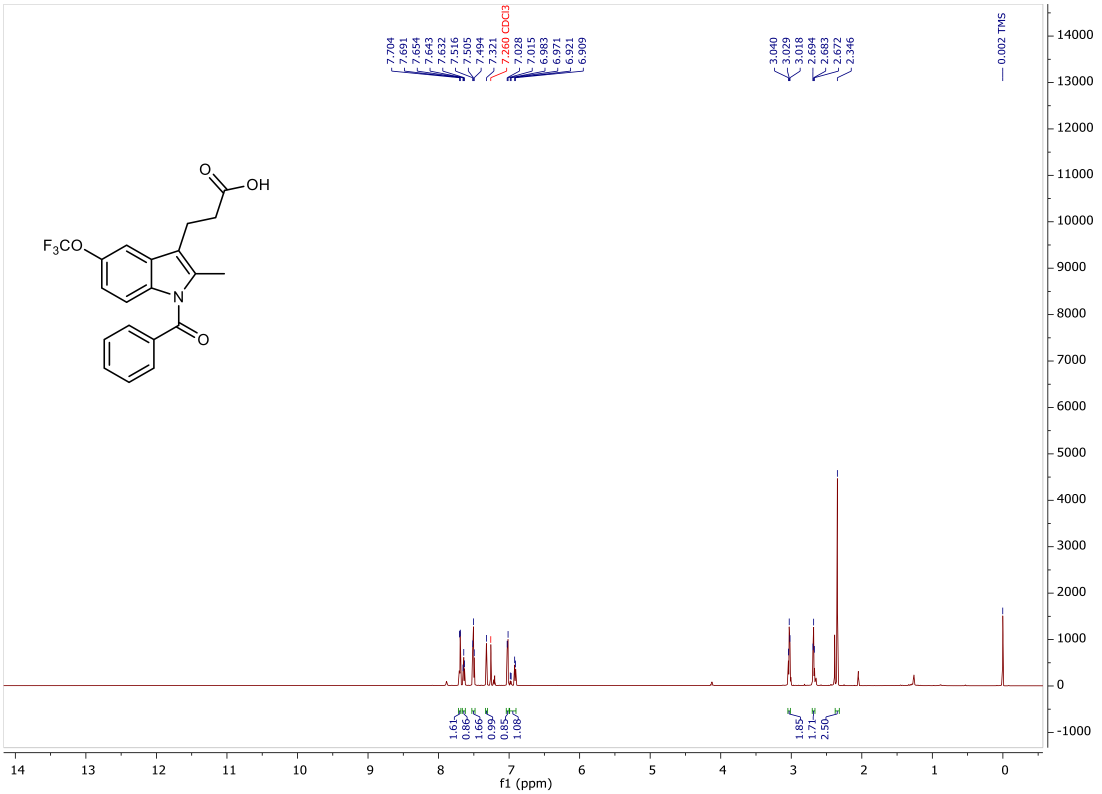

Supplementary Fig. 84. **^{13}C NMR of IA148 (126 MHz, $CDCl_3$)**

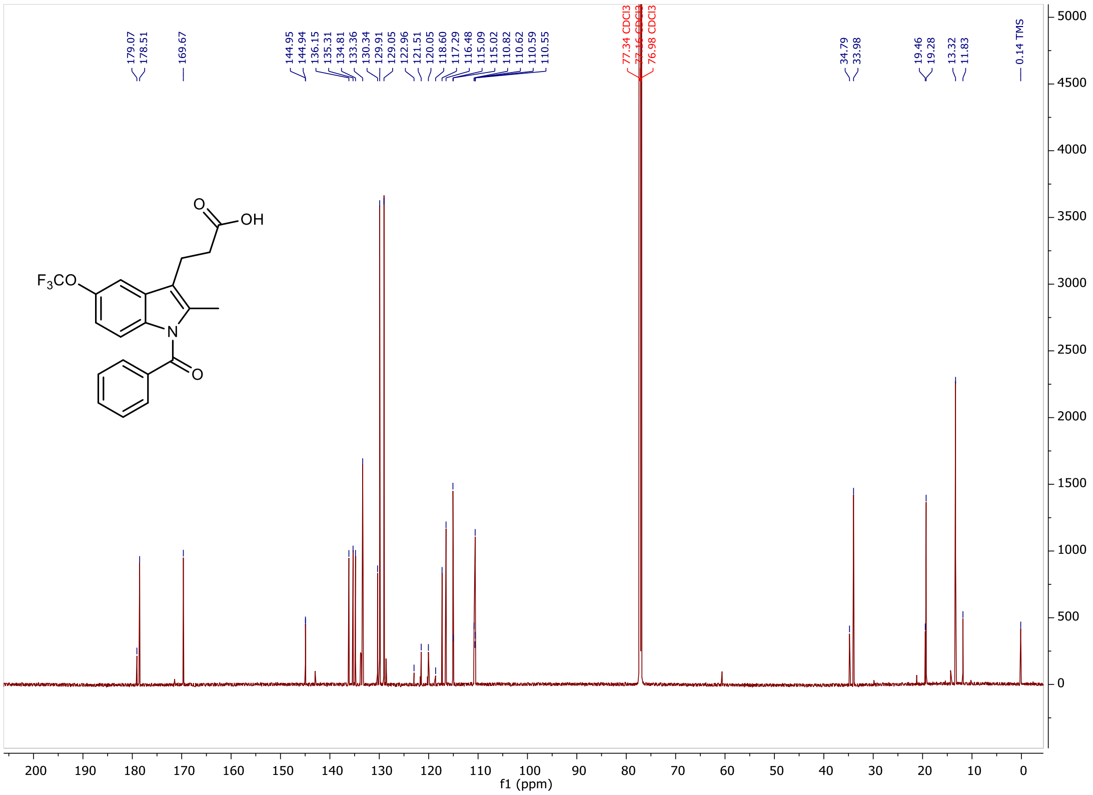

Supplementary Fig. 85. **^{19}F NMR of IA148 (470 MHz, $CDCl_3$)**

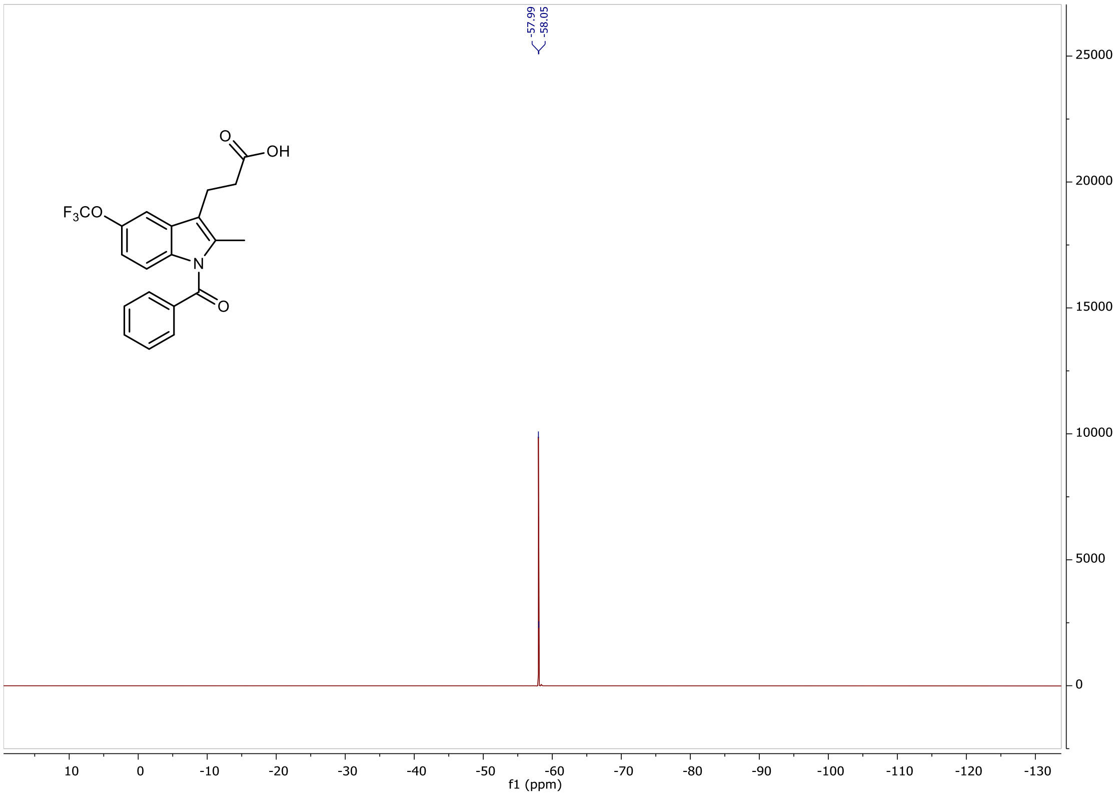

Supplementary Fig. 86. ^{1}H NMR of 149 (700 MHz, $CDCl_3$)

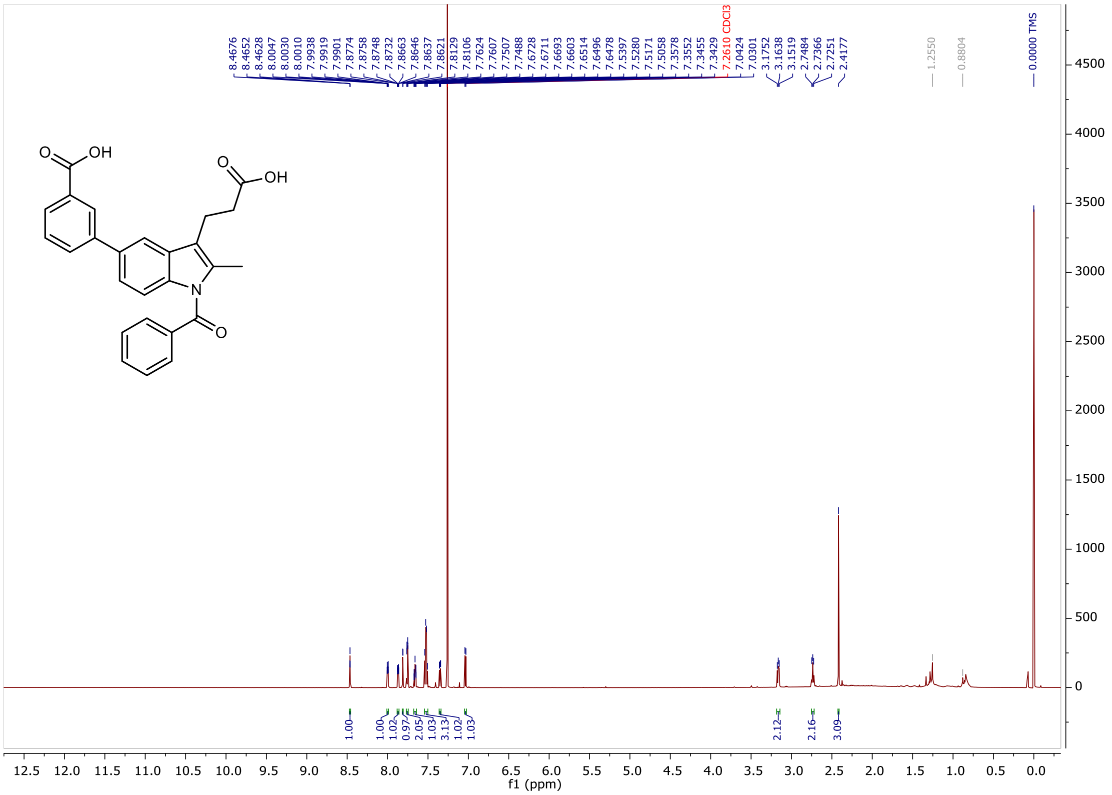

Supplementary Fig. 87. ^{13}C NMR of IA149 (176 MHz, $CDCl_3$)

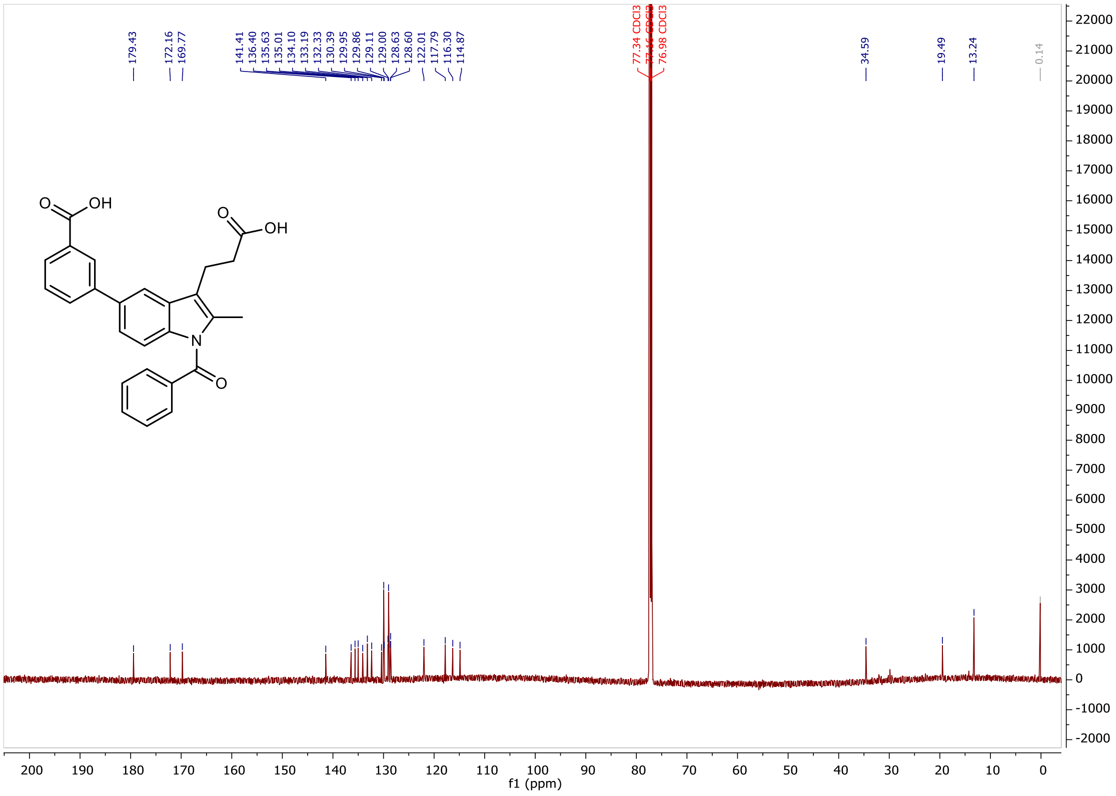

Supplementary Fig. 88. ^{1}H NMR of 152 (700 MHz, DMSO-d_6)

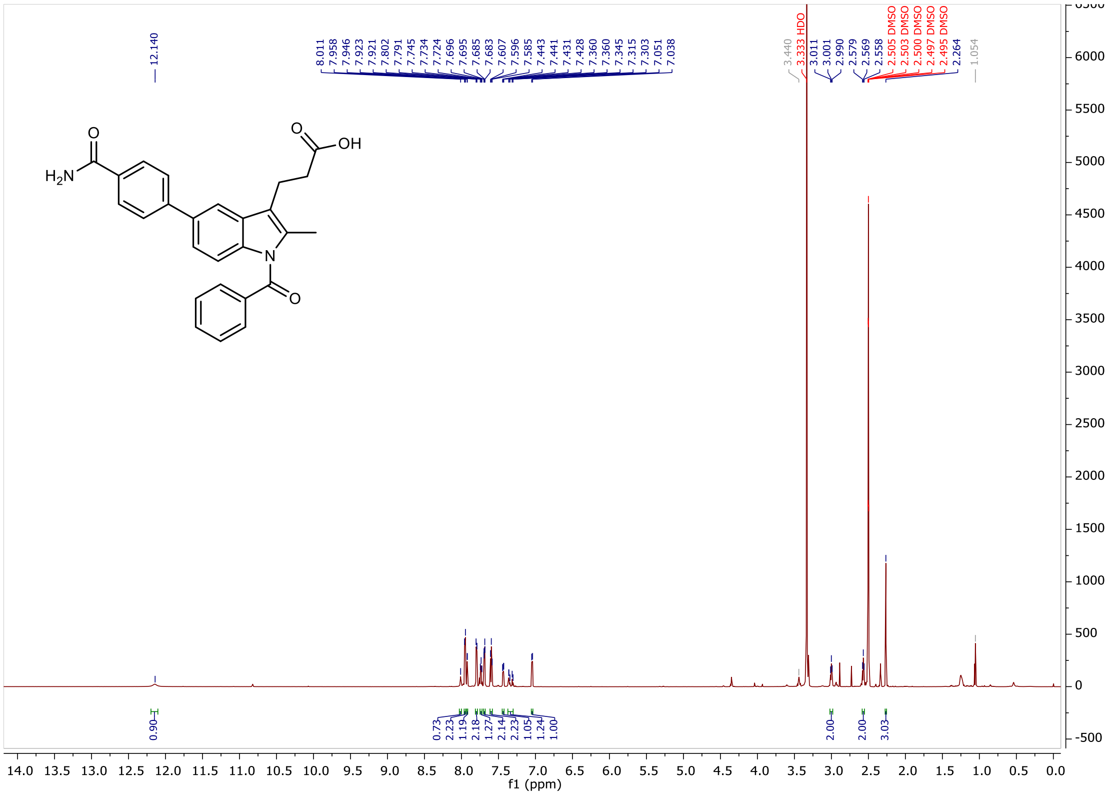

Supplementary Fig. 89. ^{13}C NMR of IA152 (176 MHz, DMSO-d_6)

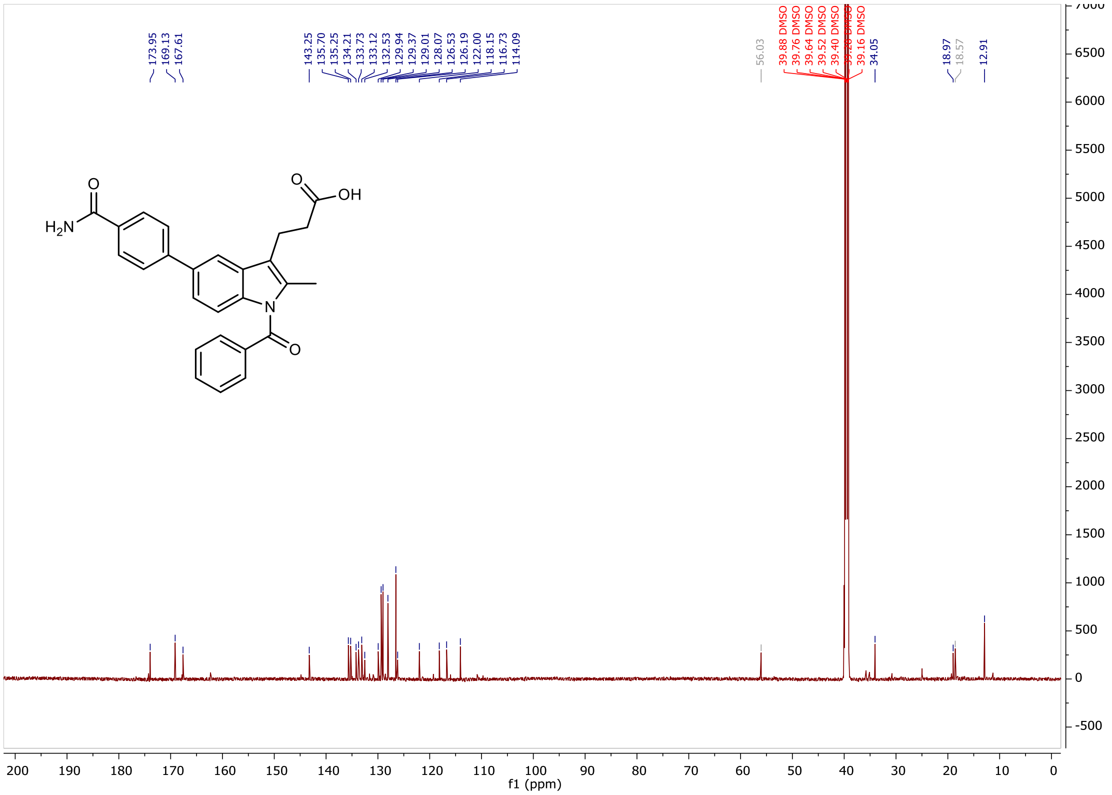

Supplementary Fig. 90. ^{1}H NMR of 153 (500 MHz, CD_3OD)

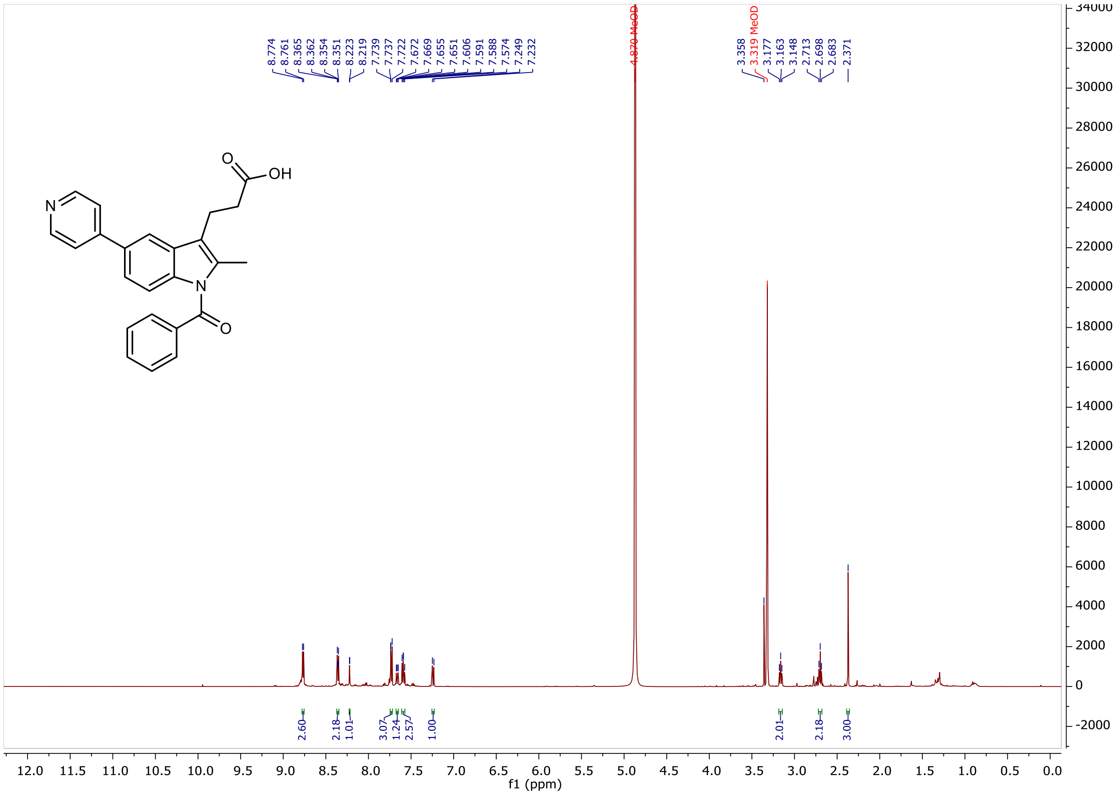

Supplementary Fig. 91. ^{13}C NMR of IA153 (126 MHz, CD_3OD)

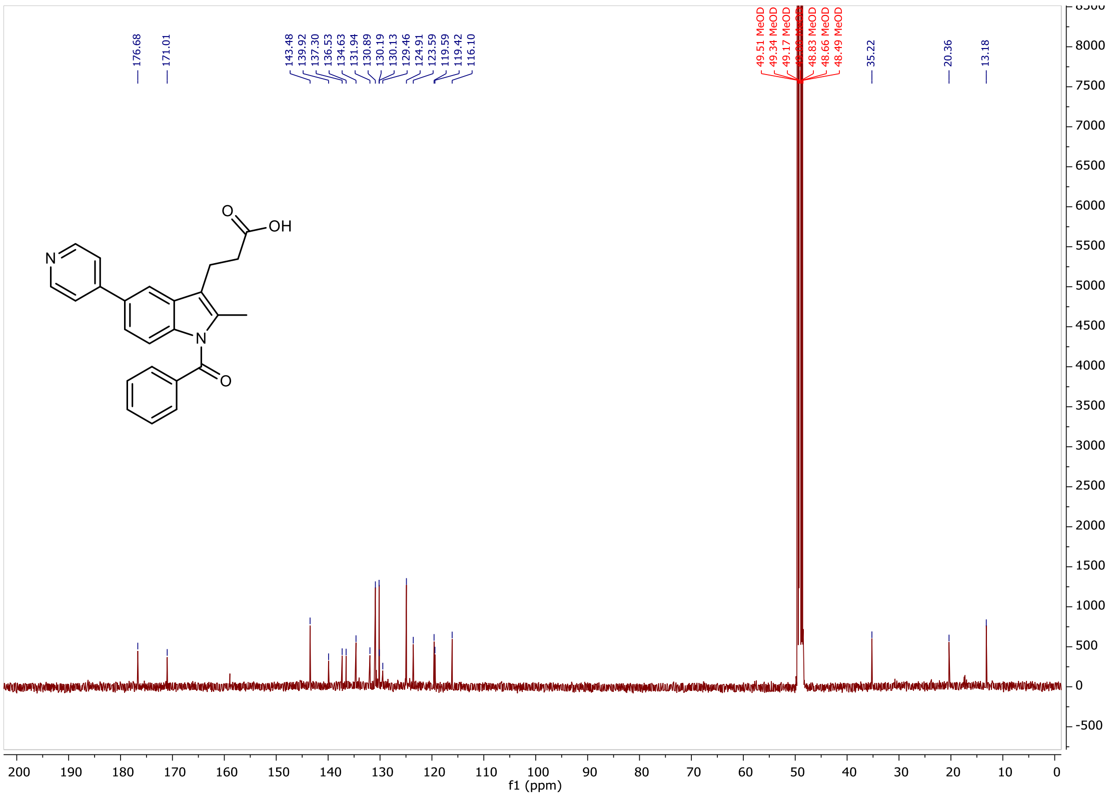

Supplementary Fig. 92. ^{1}H NMR of 158 (700 MHz, $CDCl_3$)

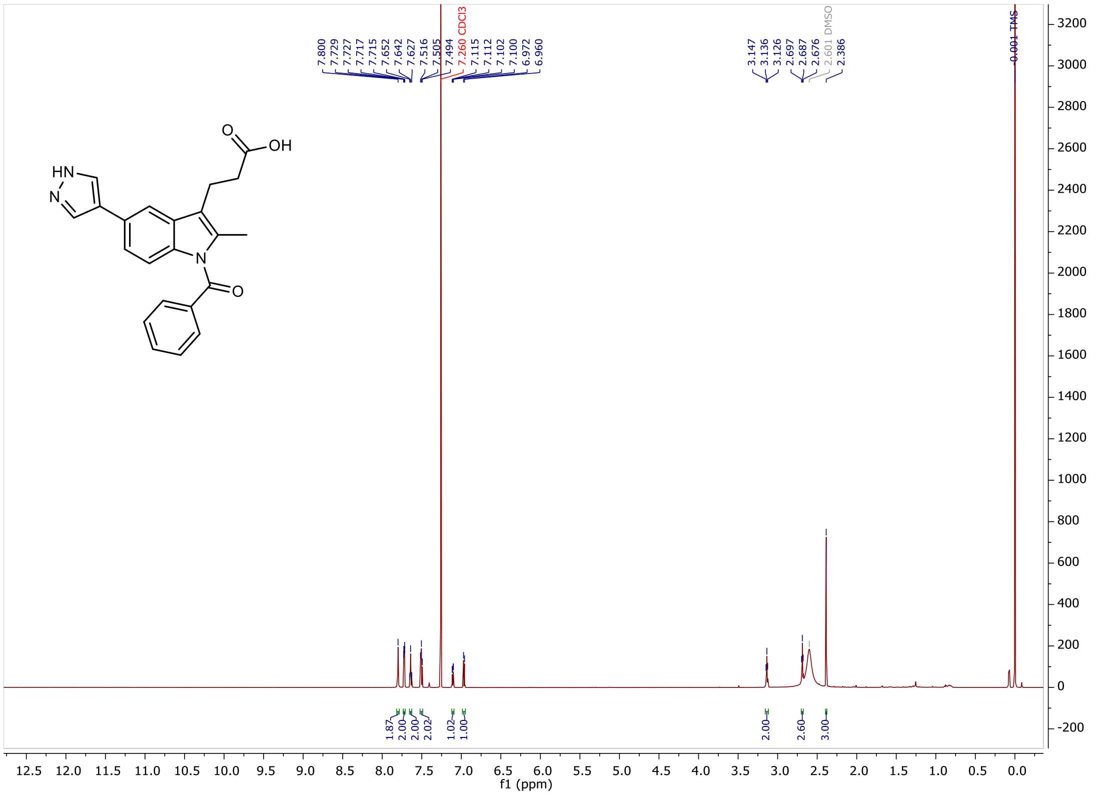

Supplementary Fig. 93. ^{13}C NMR of IA158 (176 MHz, $CDCl_3$)

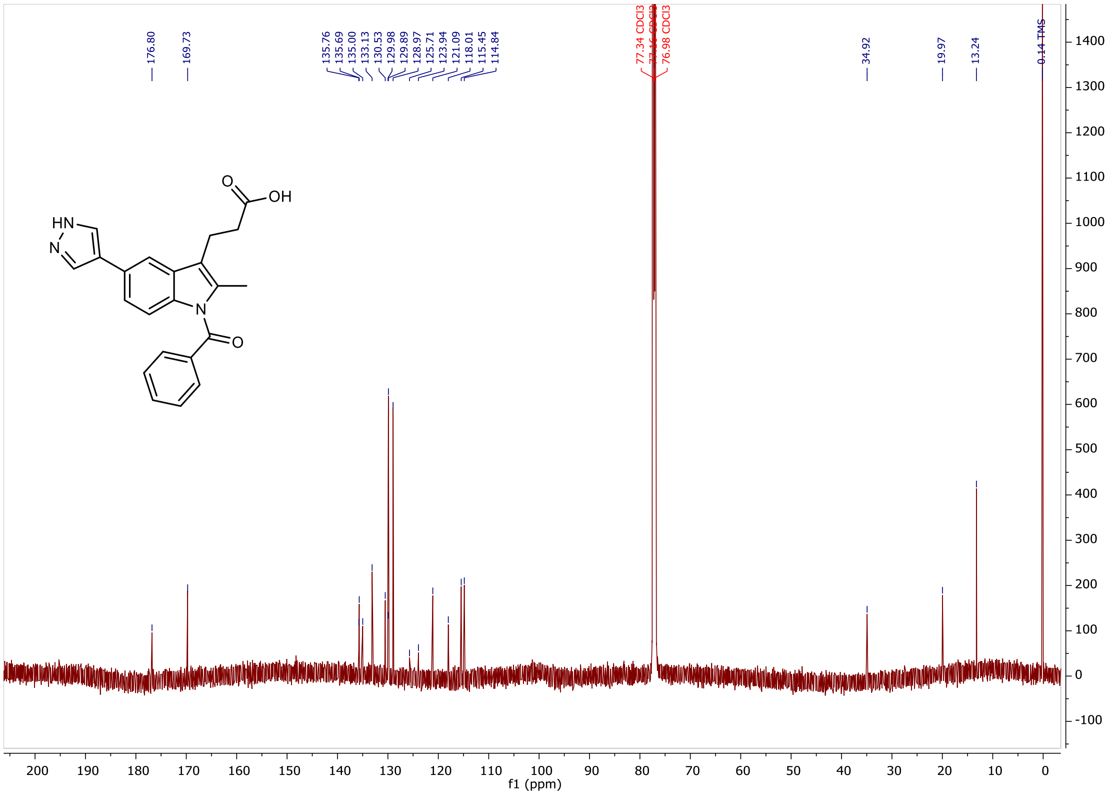

Supplementary Fig. 94. ^{1}H NMR of 159 (600 MHz, $CDCl_3$)

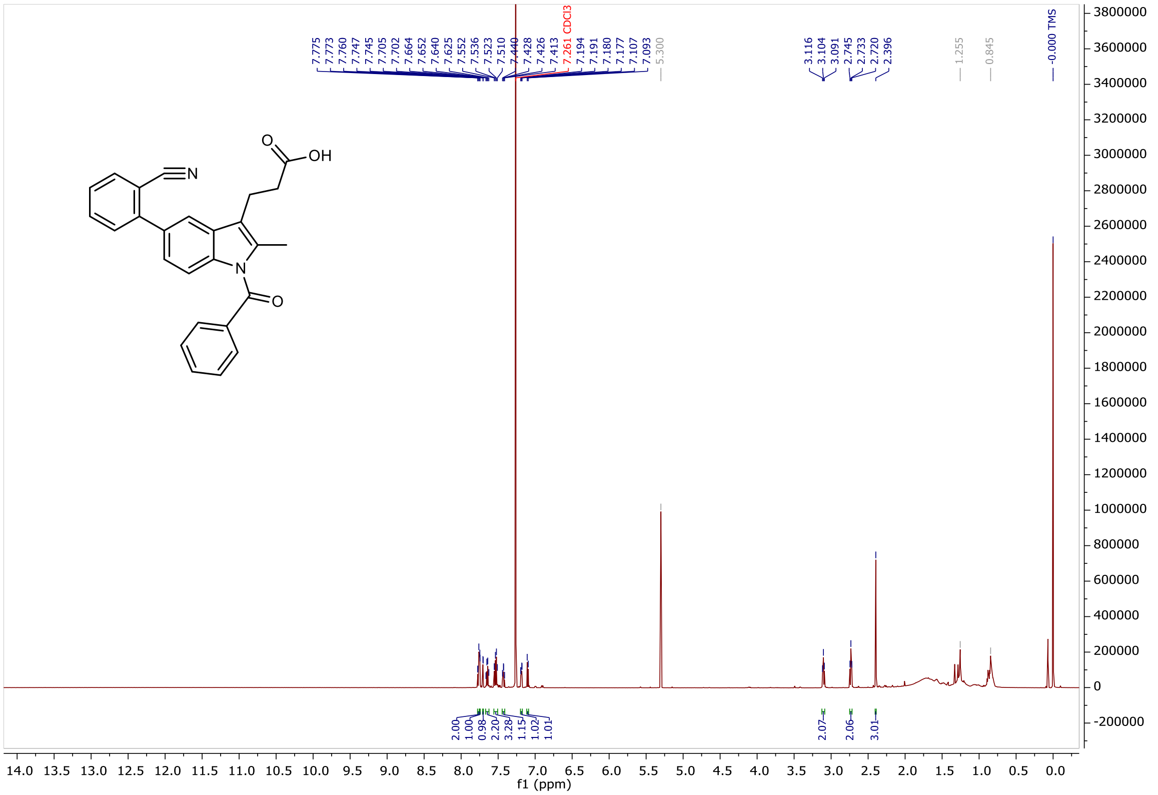

Supplementary Fig. 95. ^{13}C NMR of IA159 (151 MHz, $CDCl_3$)

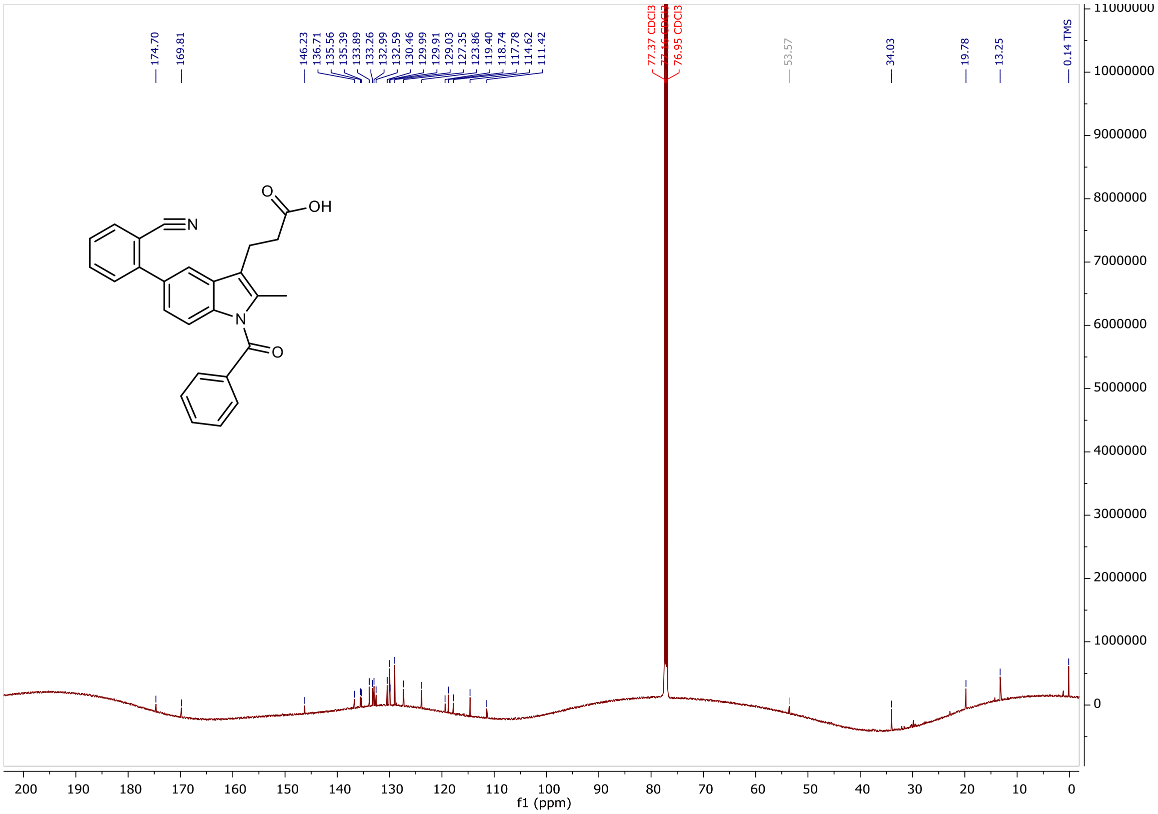

Supplementary Fig. 96. ^{1}H NMR of 162 (700 MHz, $CDCl_3$)

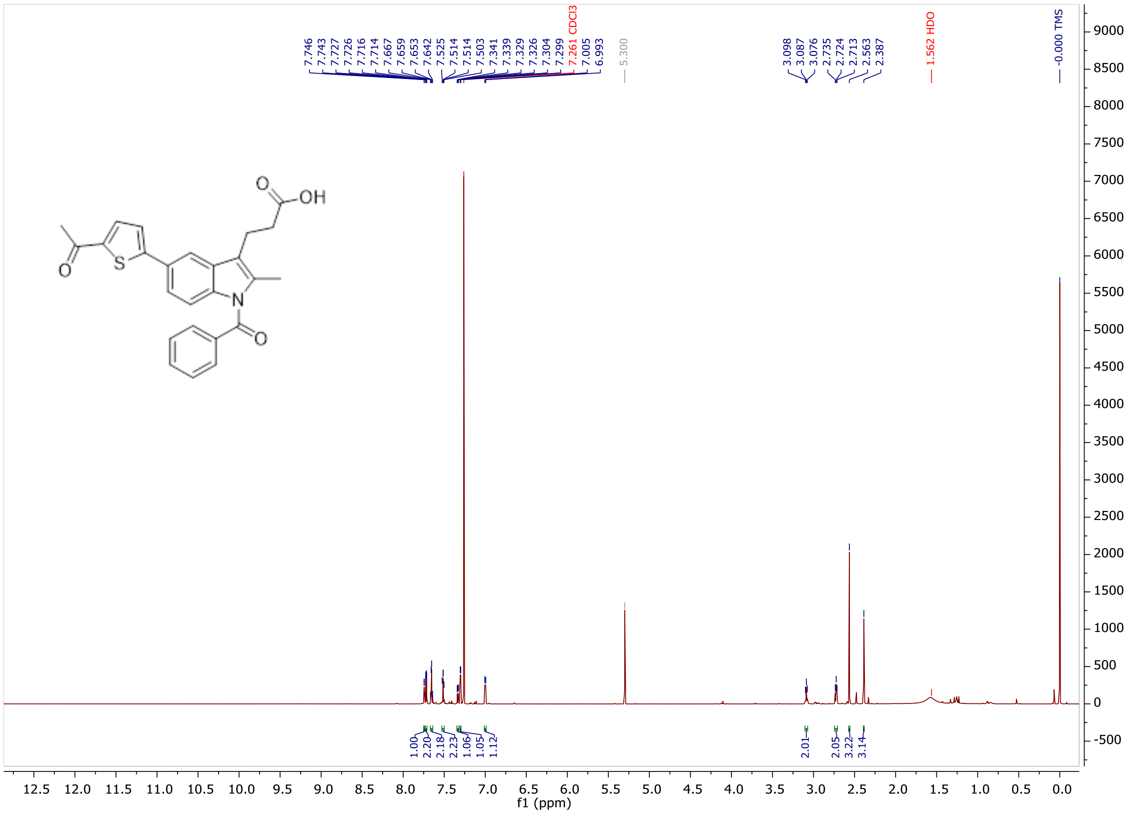

Supplementary Fig. 97. ^{13}C NMR of IA162 (176 MHz, $CDCl_3$)

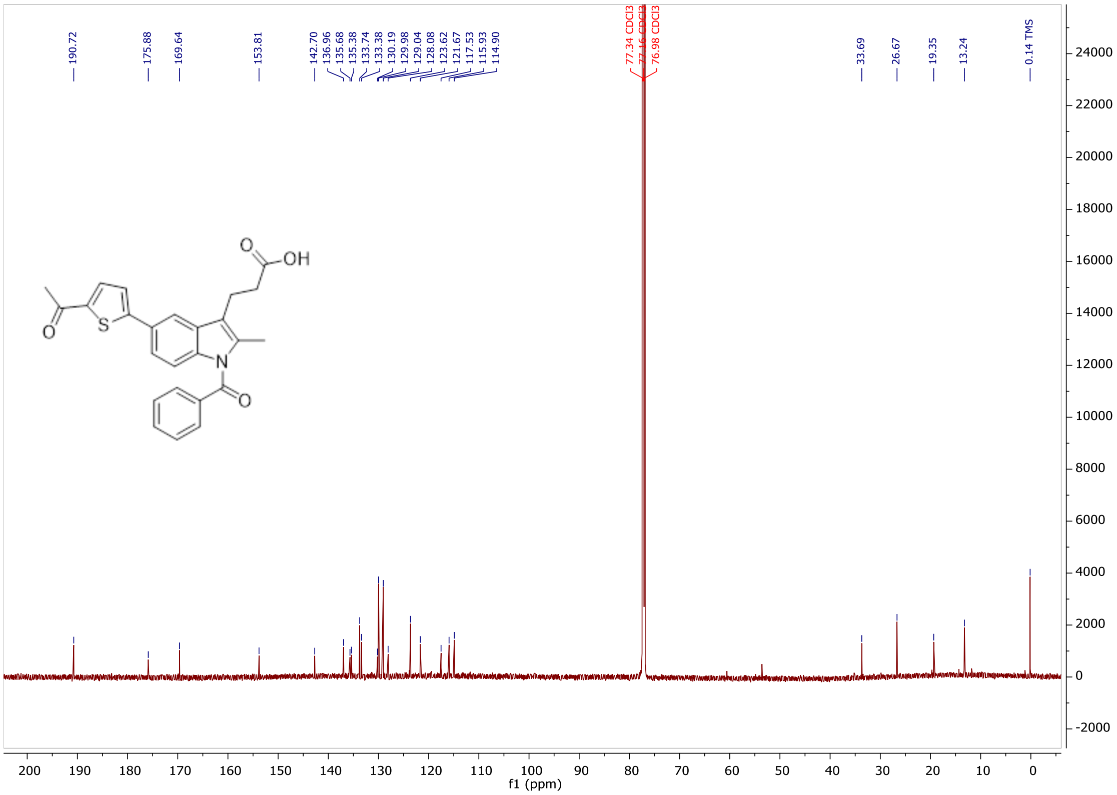

Supplementary Fig. 98. ^{1}H NMR of 163 (700 MHz, DMSO-d_6)

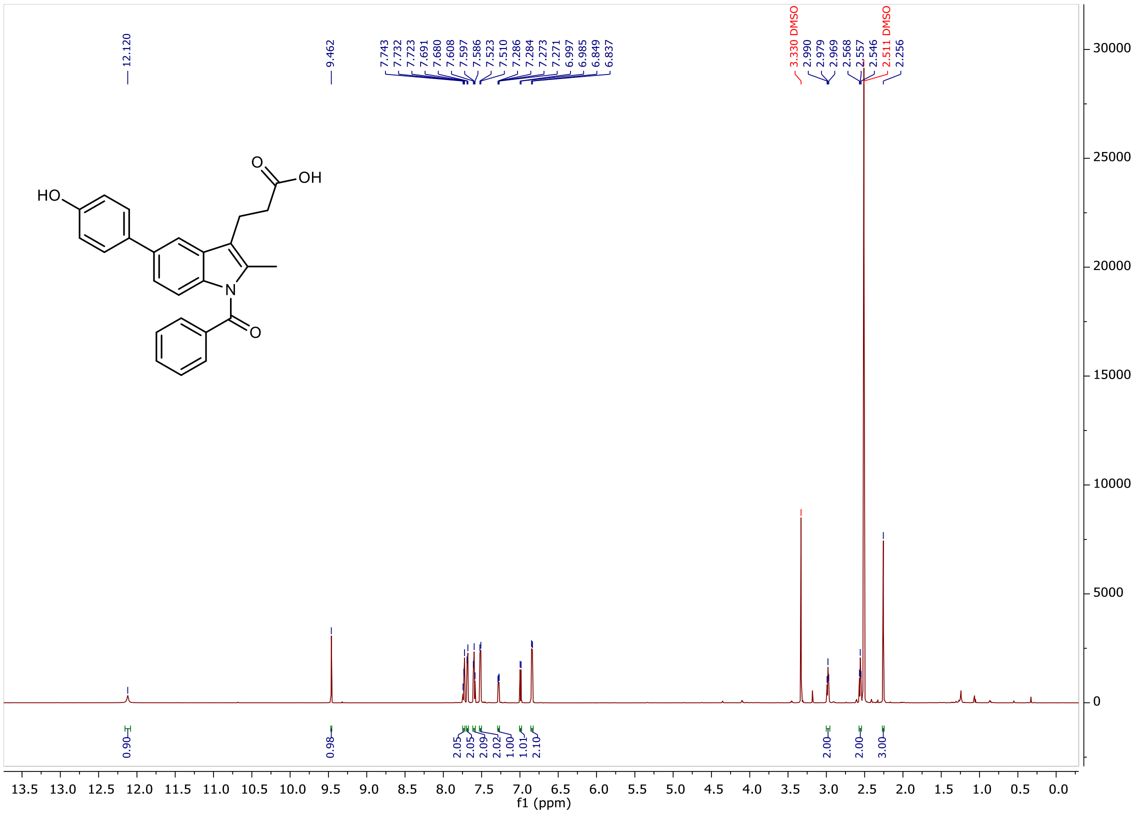

Supplementary Fig. 99. ^{13}C NMR of IA163 (176 MHz, DMSO-d_6)

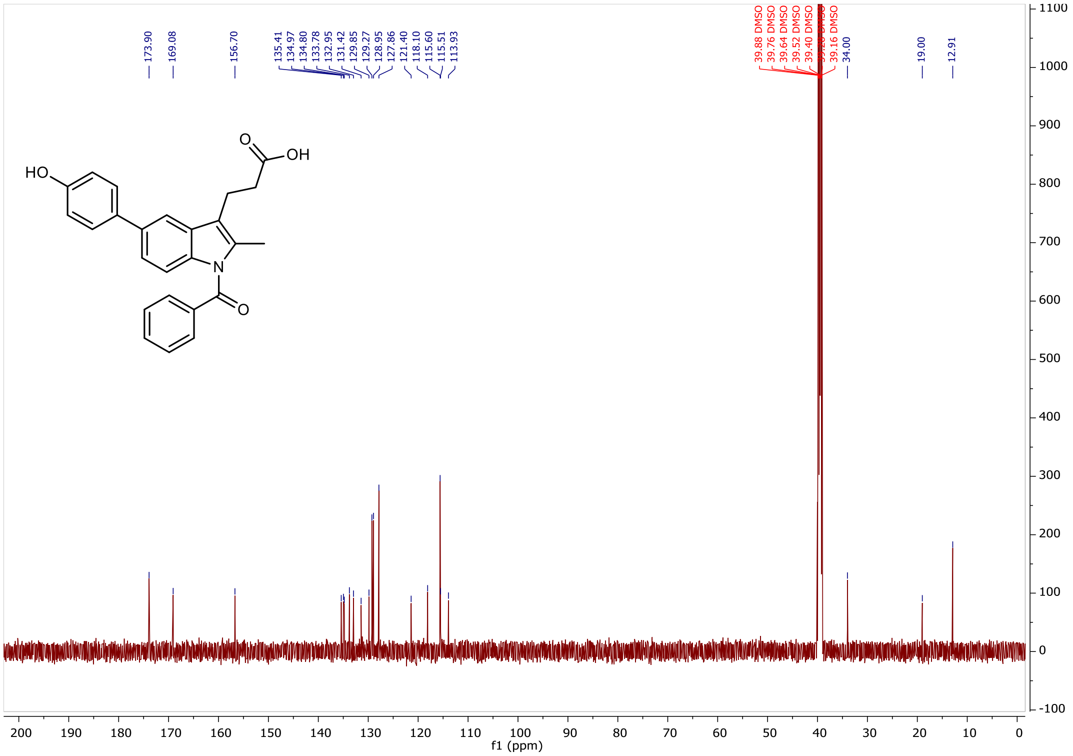

Supplementary Fig. 100. ^{1}H NMR of IA164 (500 MHz, $CDCl_3$)

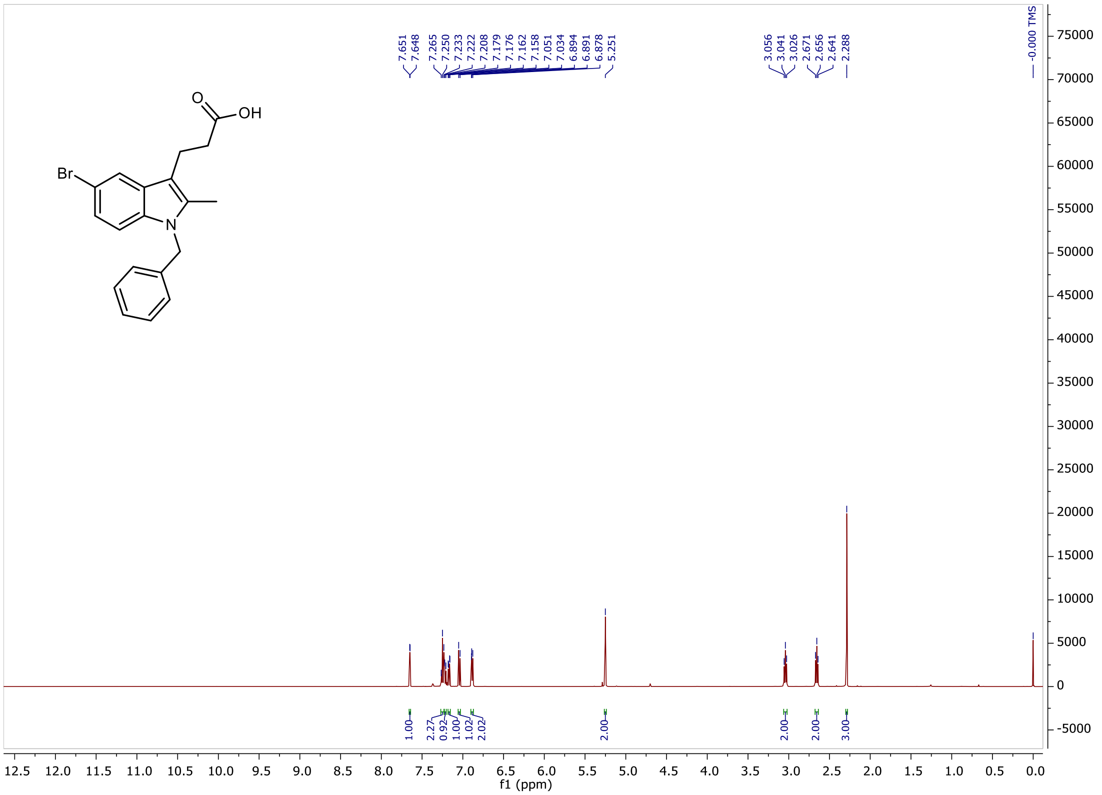

Supplementary Fig. 101. ^{13}C NMR of IA164 (126 MHz, $CDCl_3$)

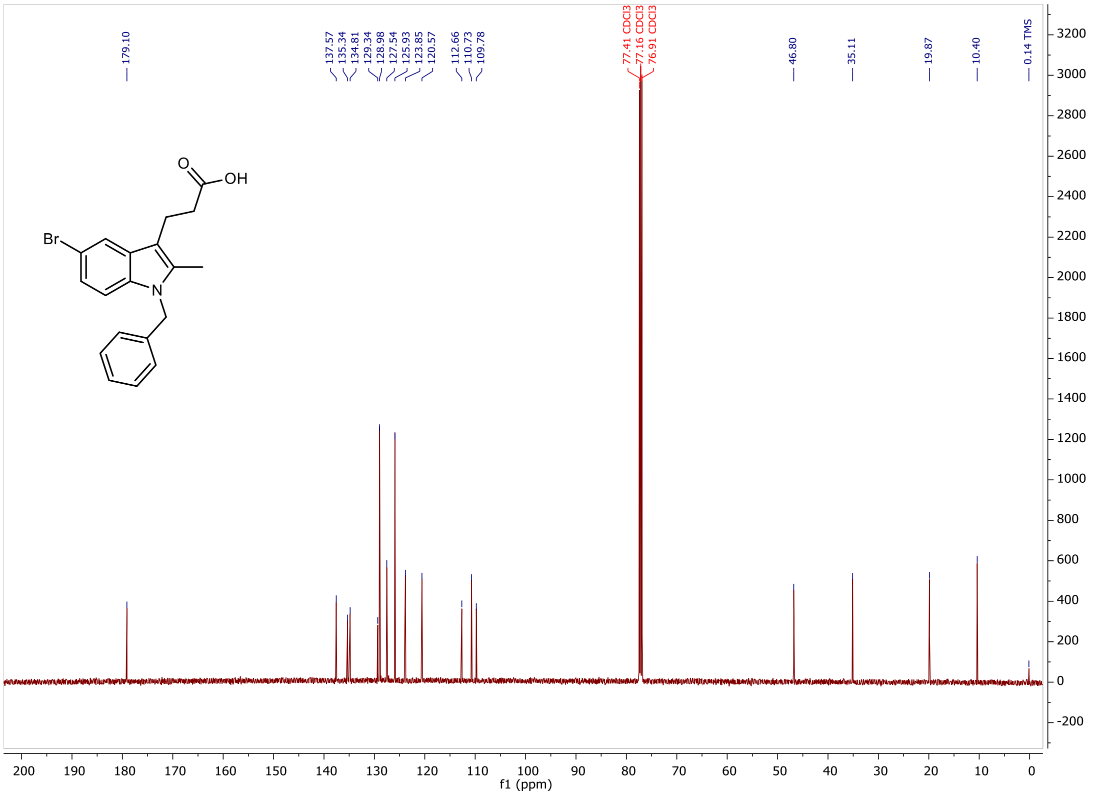

Supplementary Fig. 102. ¹H NMR of IA165 (700 MHz, $CDCl_3$)

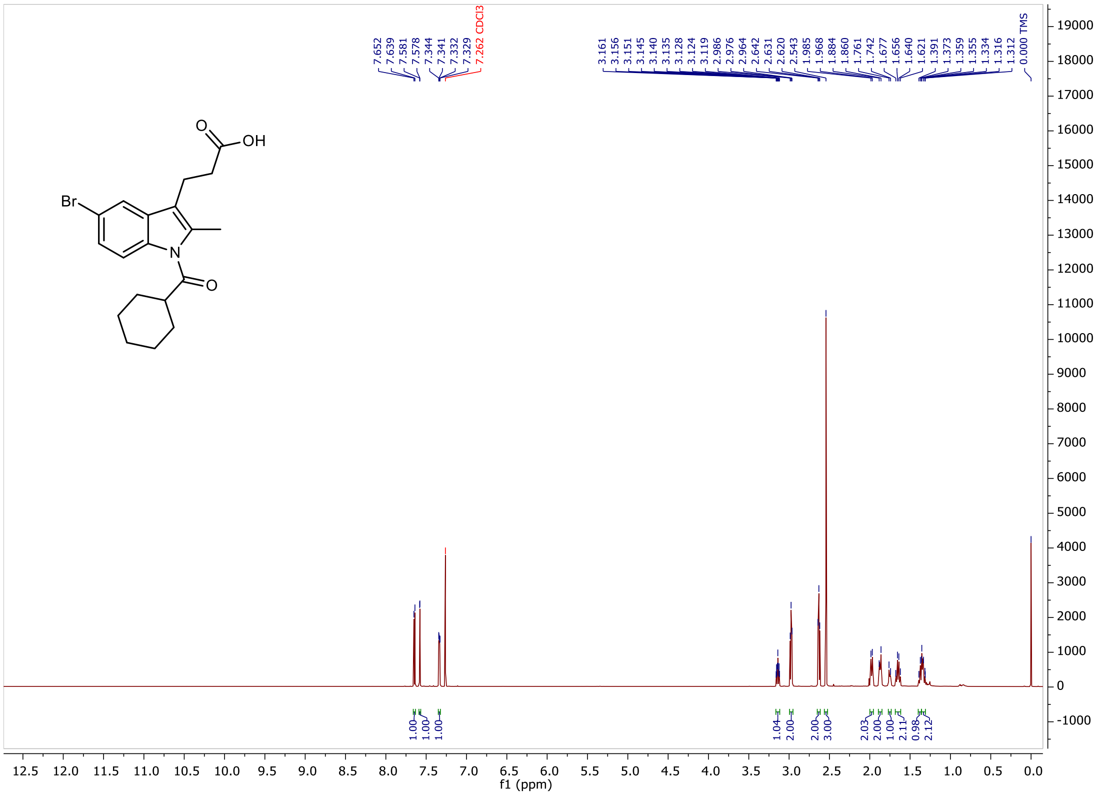

Supplementary Fig. 103. ¹³C NMR of IA165 (176 MHz, $CDCl_3$)

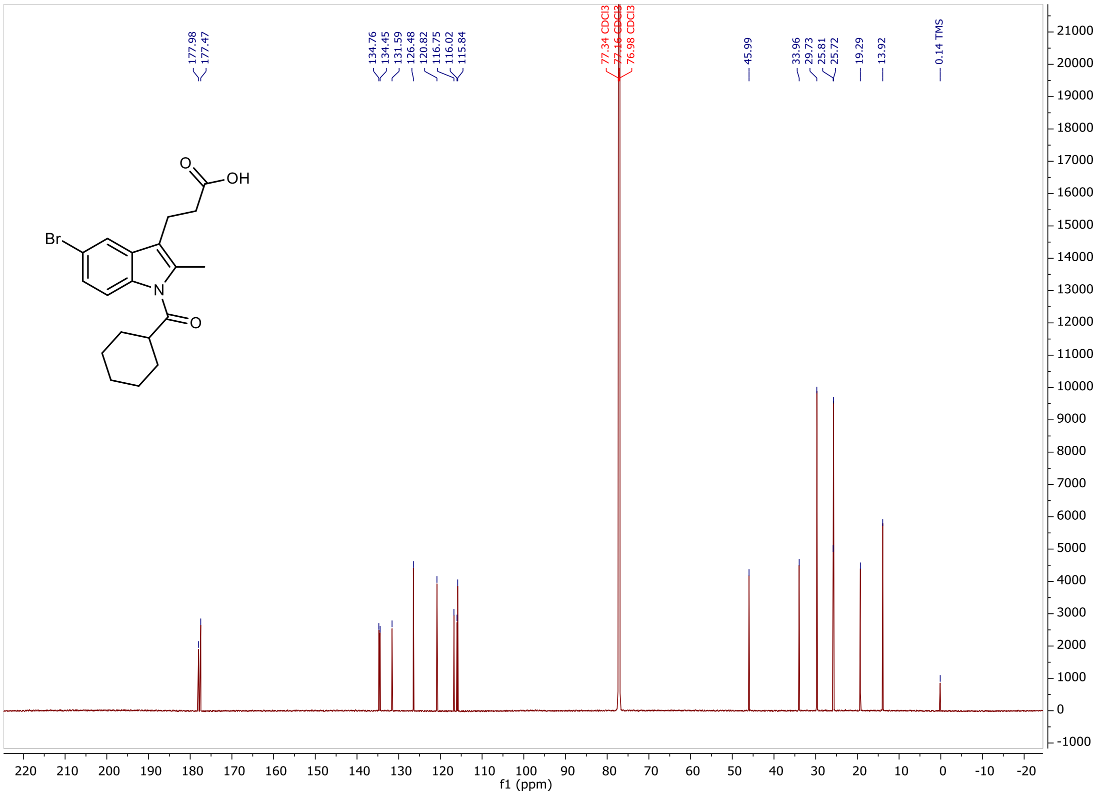

Supplementary Fig. 104. ^{1}H NMR of IA41 (700 MHz, $CDCl_3$)

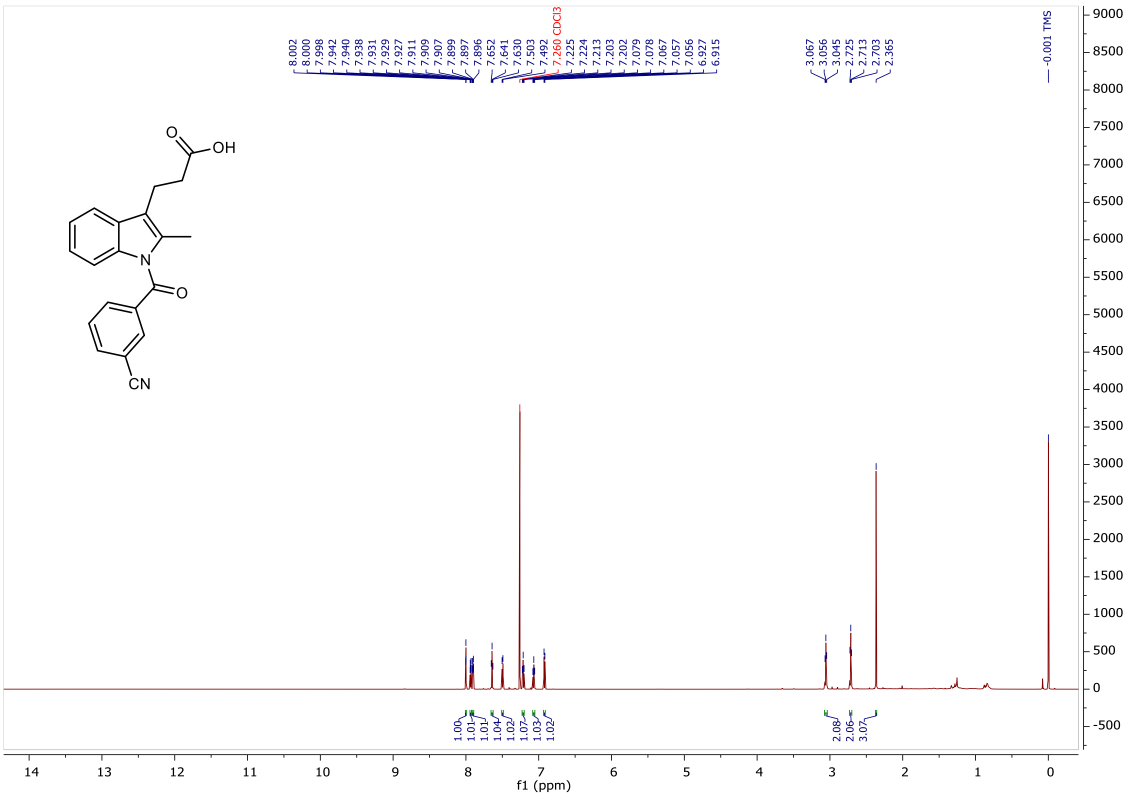

Supplementary Fig. 105. ^{13}C NMR of IA41 (176 MHz, $CDCl_3$)

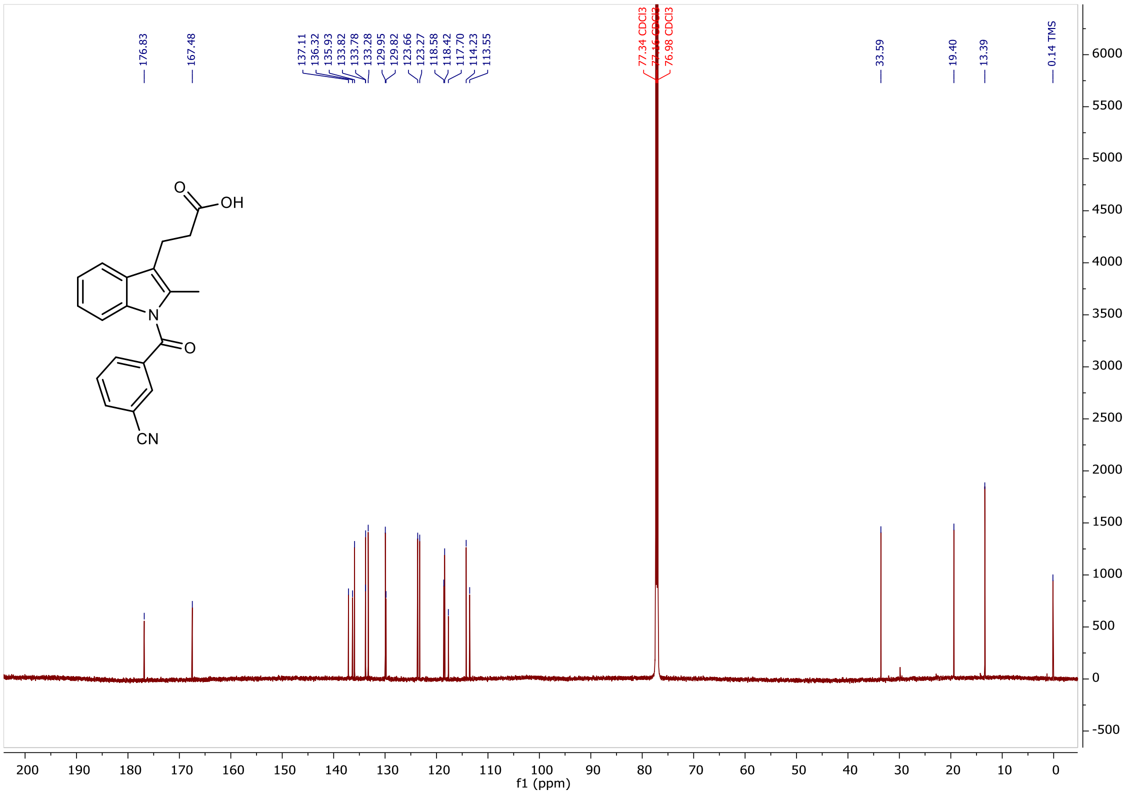

Supplementary Fig. 106. ^{1}H NMR of IA42 (700 MHz, $CDCl_3$)

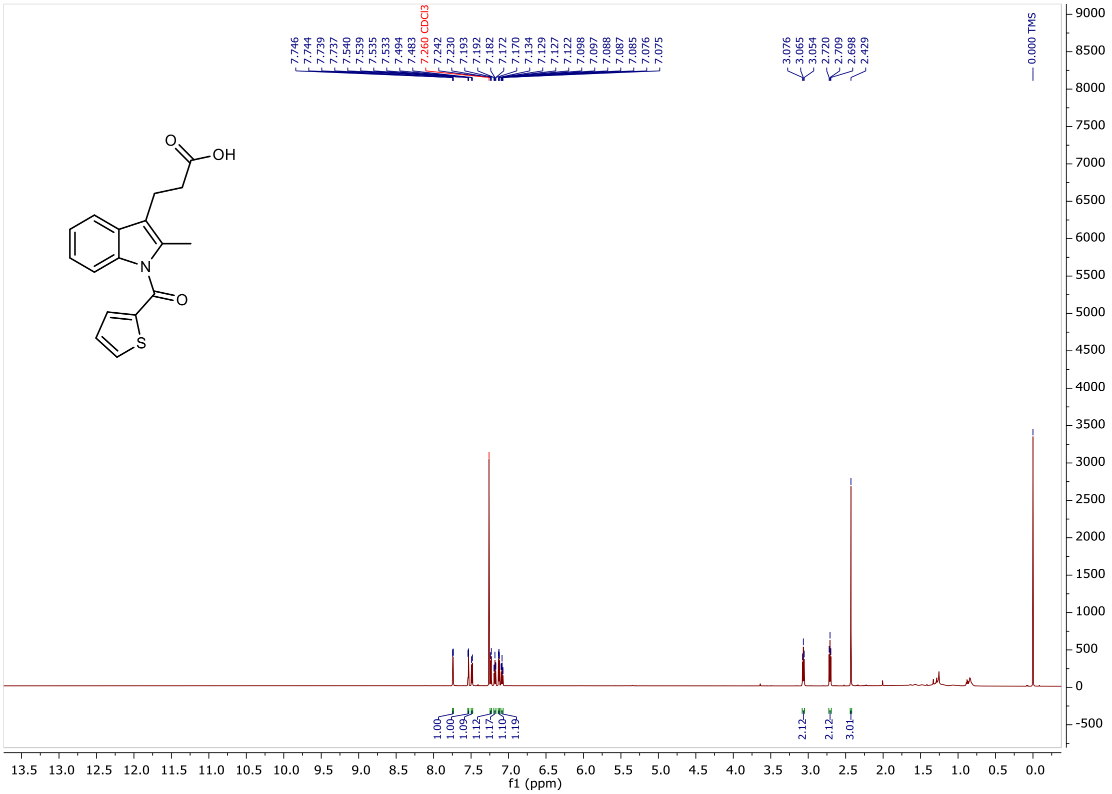

Supplementary Fig. 107. ^{13}C NMR of IA42 (176 MHz, $CDCl_3$)

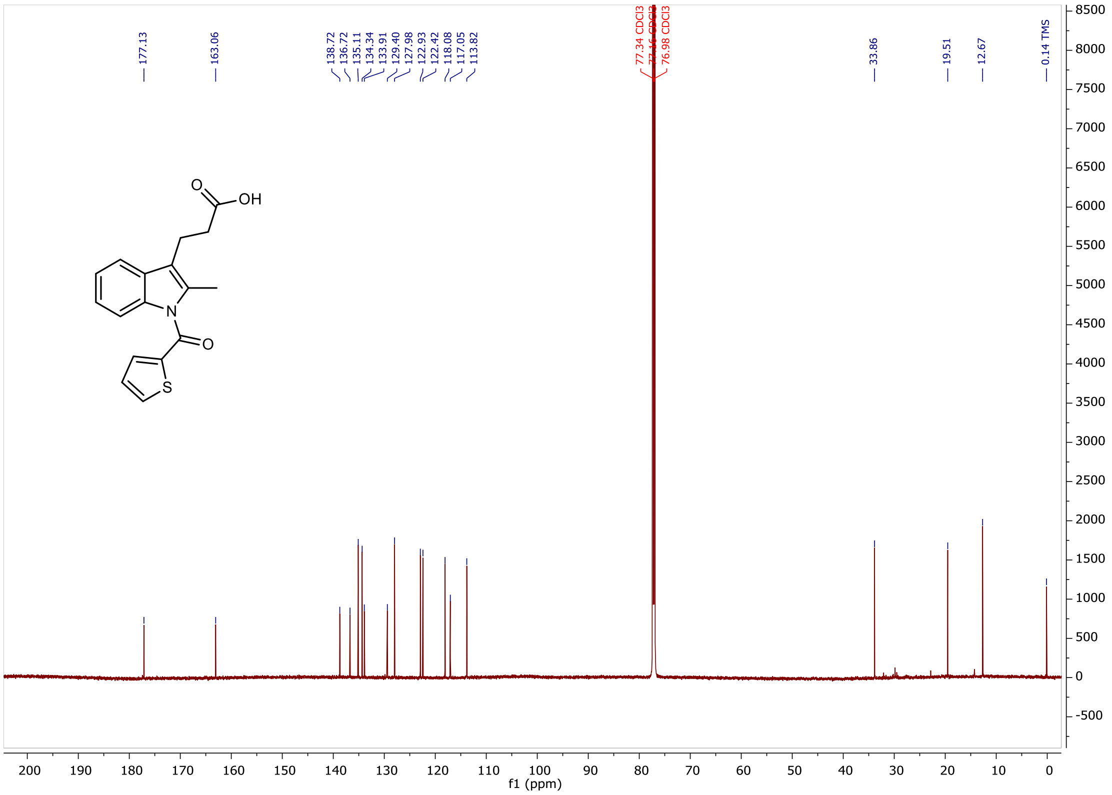

Supplementary Fig. 108. ^{1}H NMR of IA44 (500 MHz, $CDCl_3$)

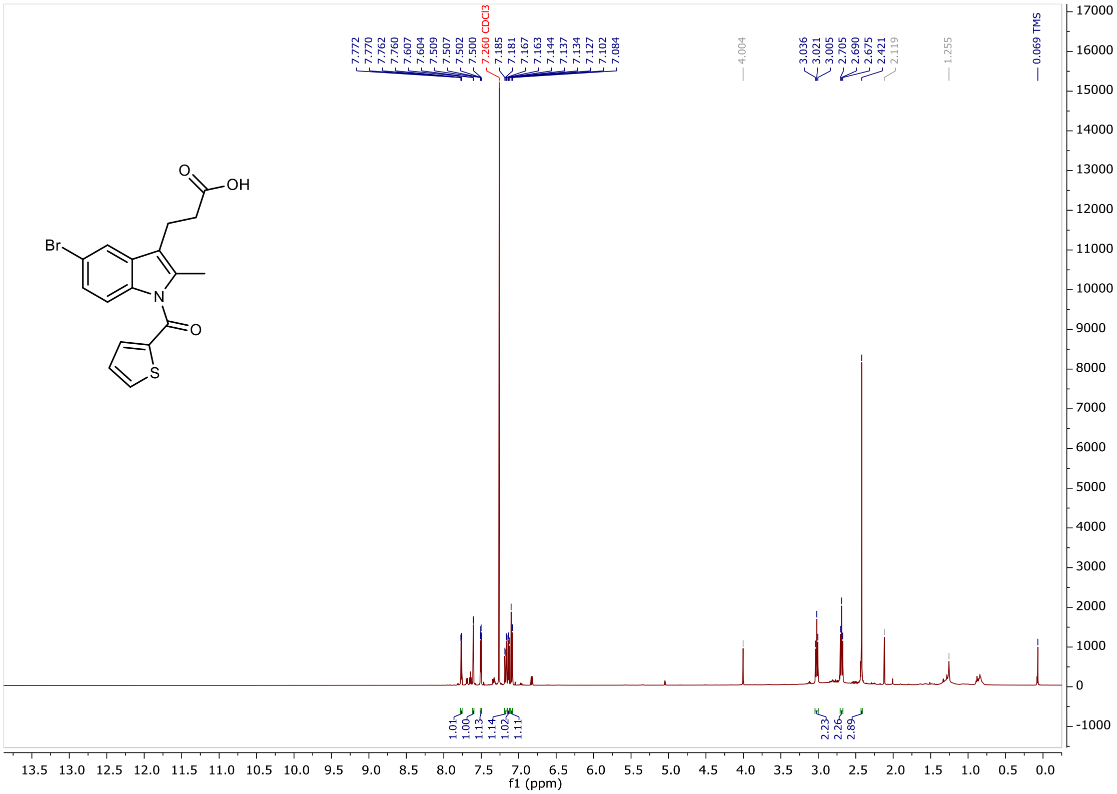

Supplementary Fig. 109. ^{13}C NMR of IA44 (126 MHz, $CDCl_3$)

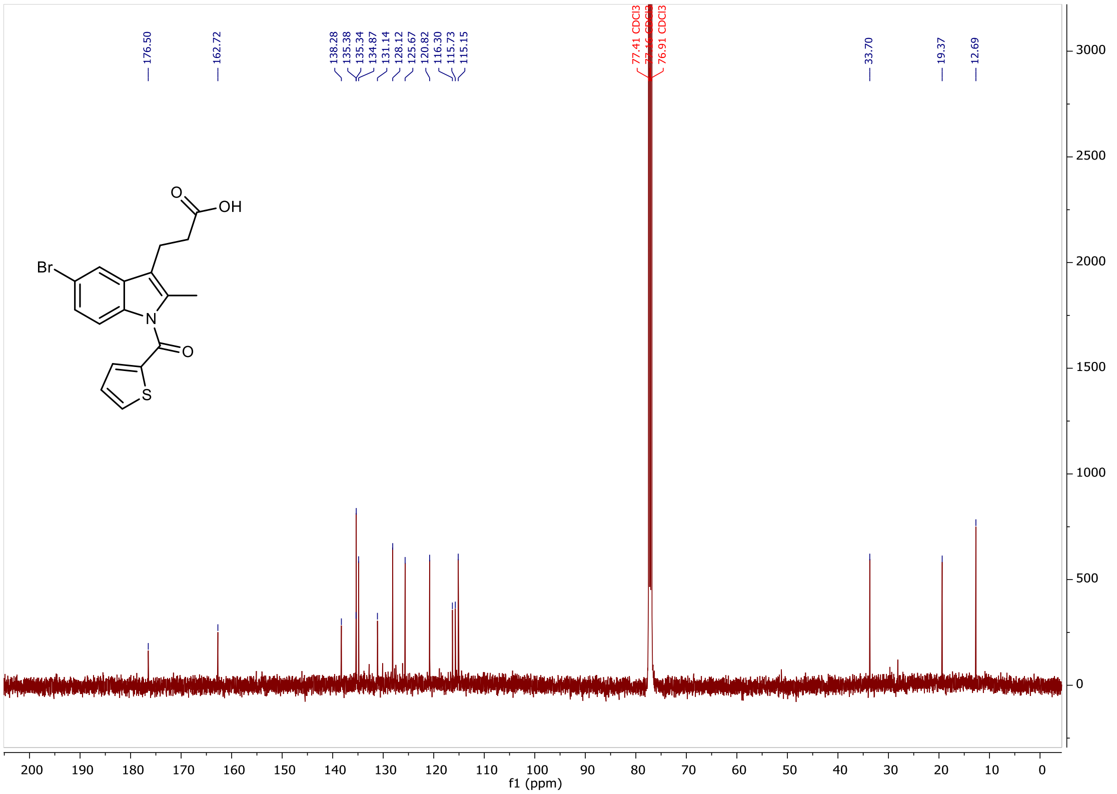

Supplementary Fig. 110. ^{1}H NMR of IA47 (600 MHz, $CDCl_3$)

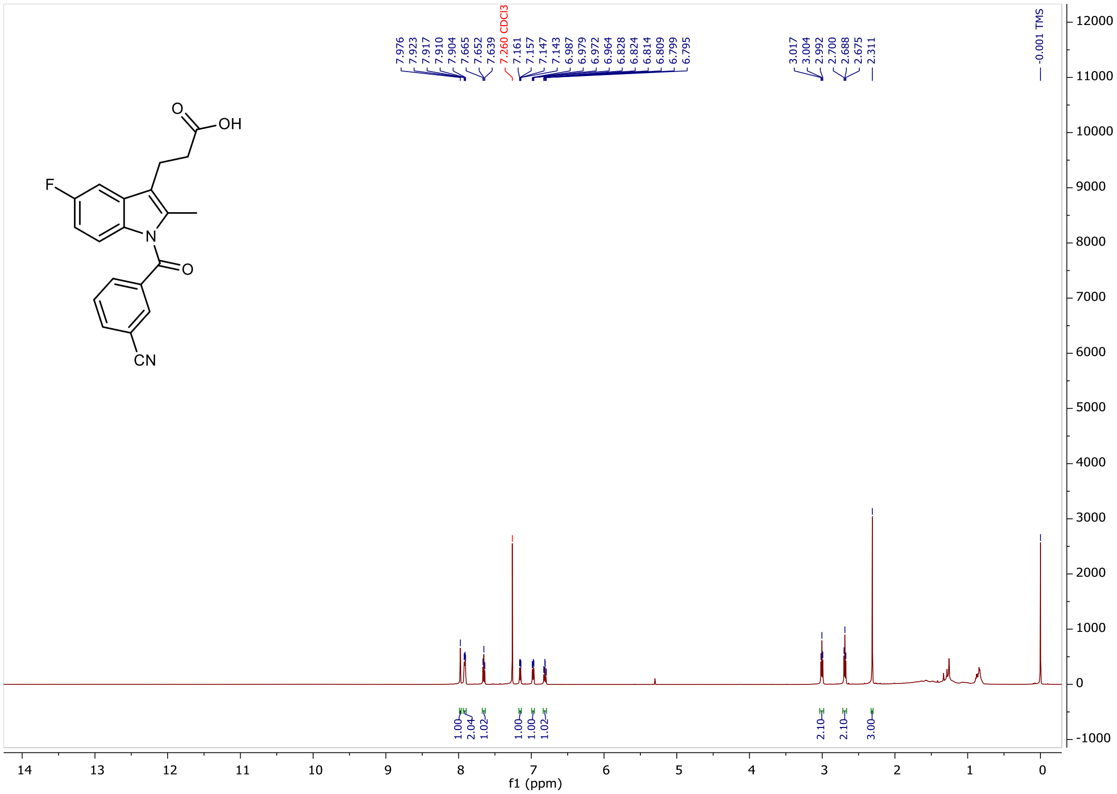

Supplementary Fig. 111. ^{13}C NMR of IA47 (151 MHz, $CDCl_3$)

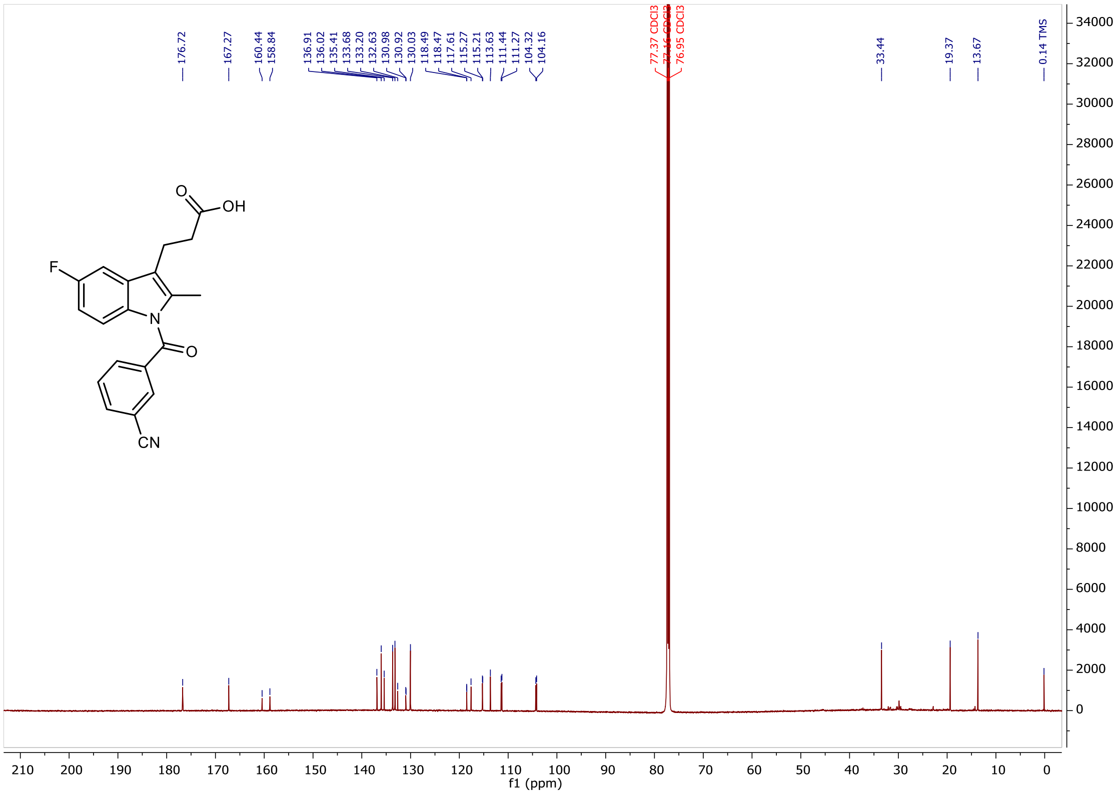

Supplementary Fig. 112. ^{1}H NMR of IA48 (600 MHz, $CDCl_3$)

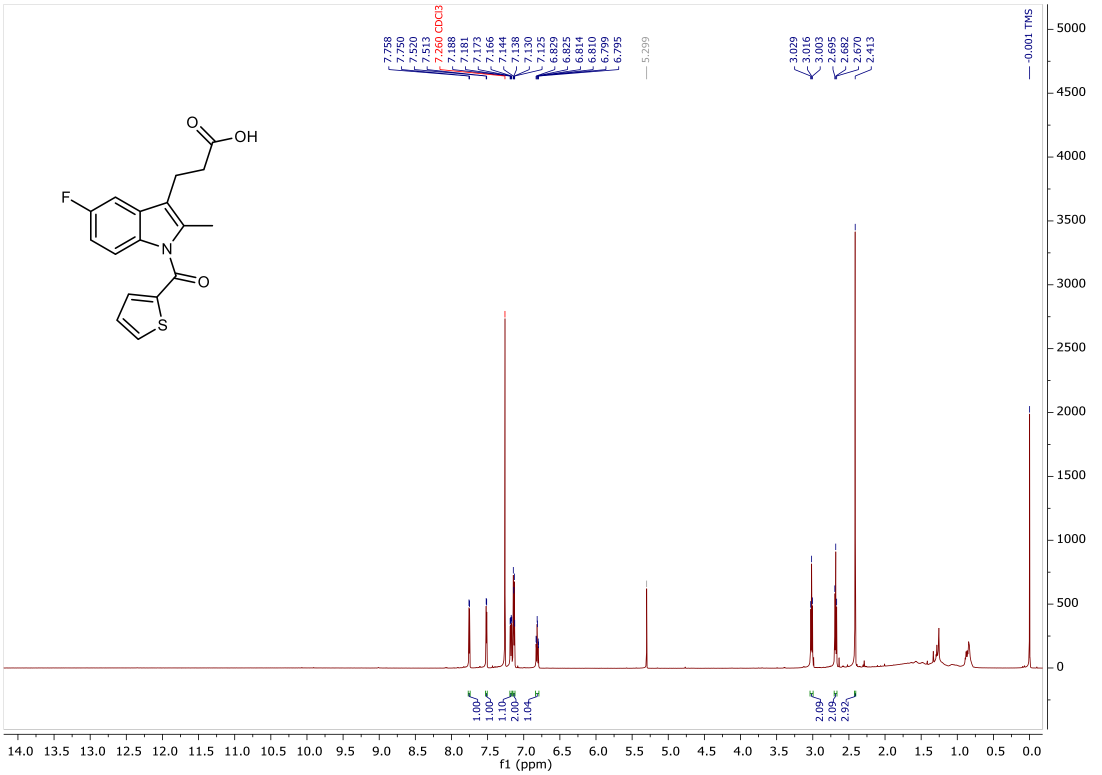

Supplementary Fig. 113. ^{13}C NMR of IA48 (151 MHz, $CDCl_3$)

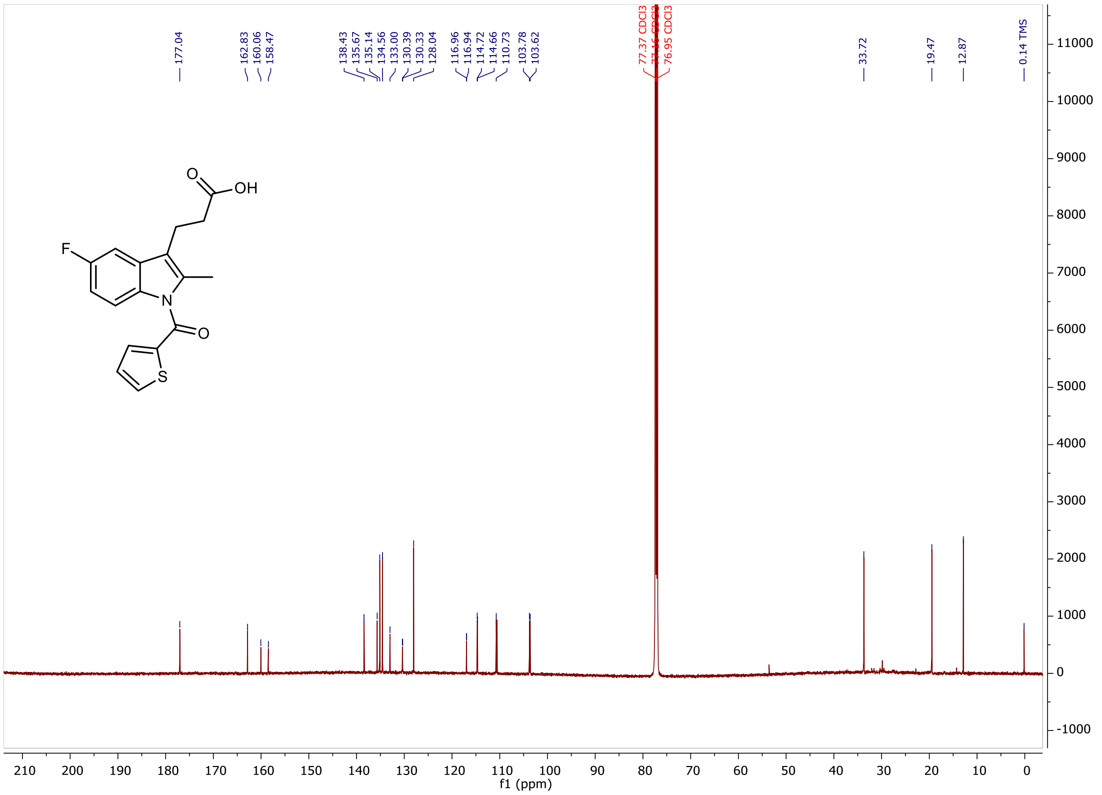

Supplementary Fig. 114. ^{1}**H NMR of IA60 (600 MHz,** $CDCl_3$**)**

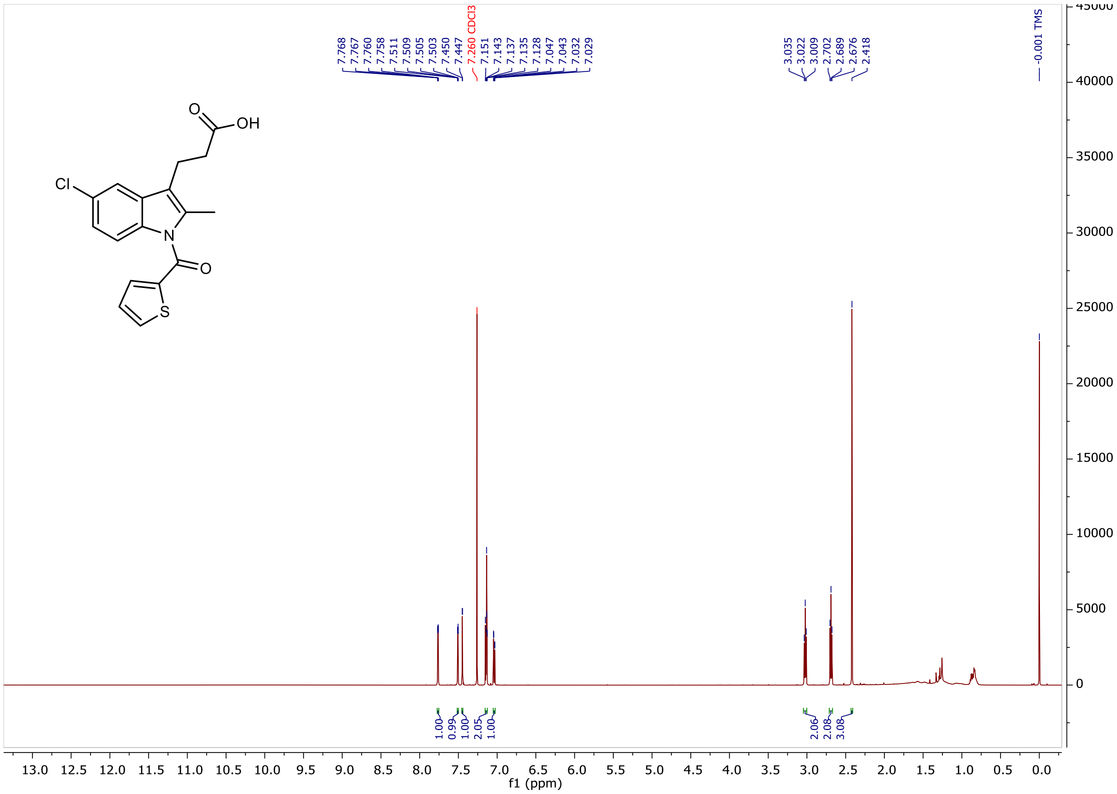

Supplementary Fig. 115. ^{13}**C NMR of IA60 (151 MHz,** $CDCl_3$**)**

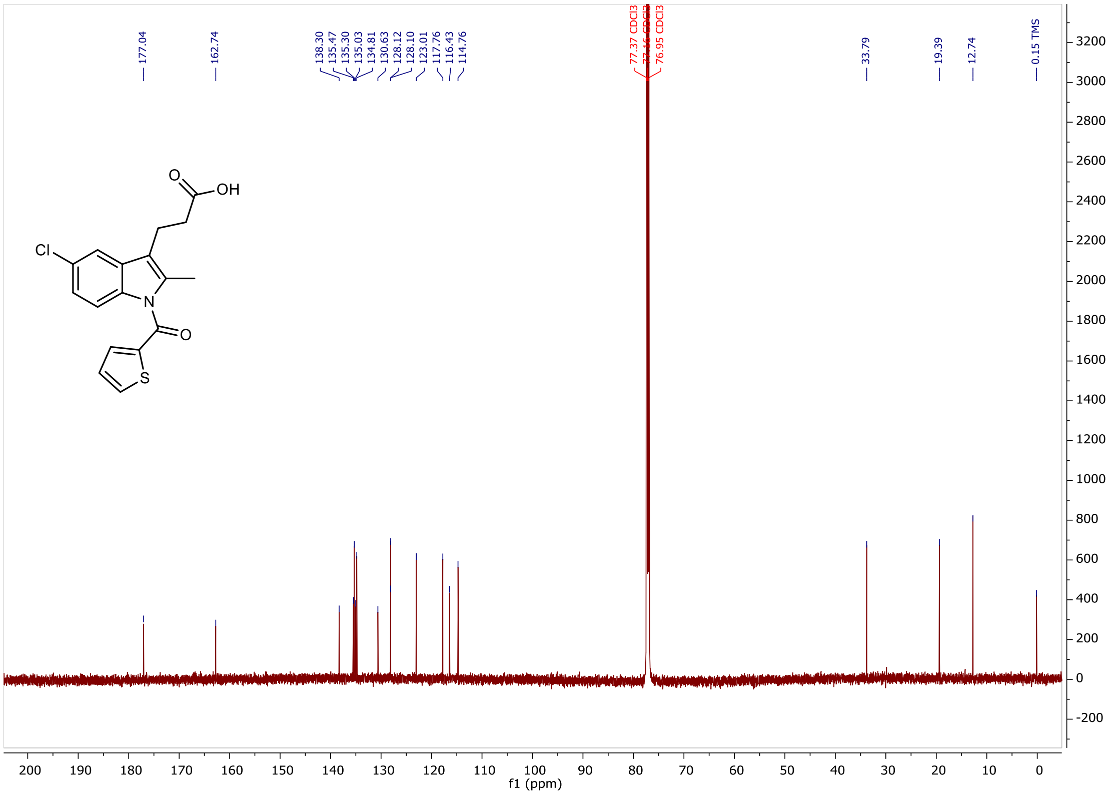

Supplementary Fig. 114. ^{1}H NMR of IA63 (700 MHz, $CDCl_3$)

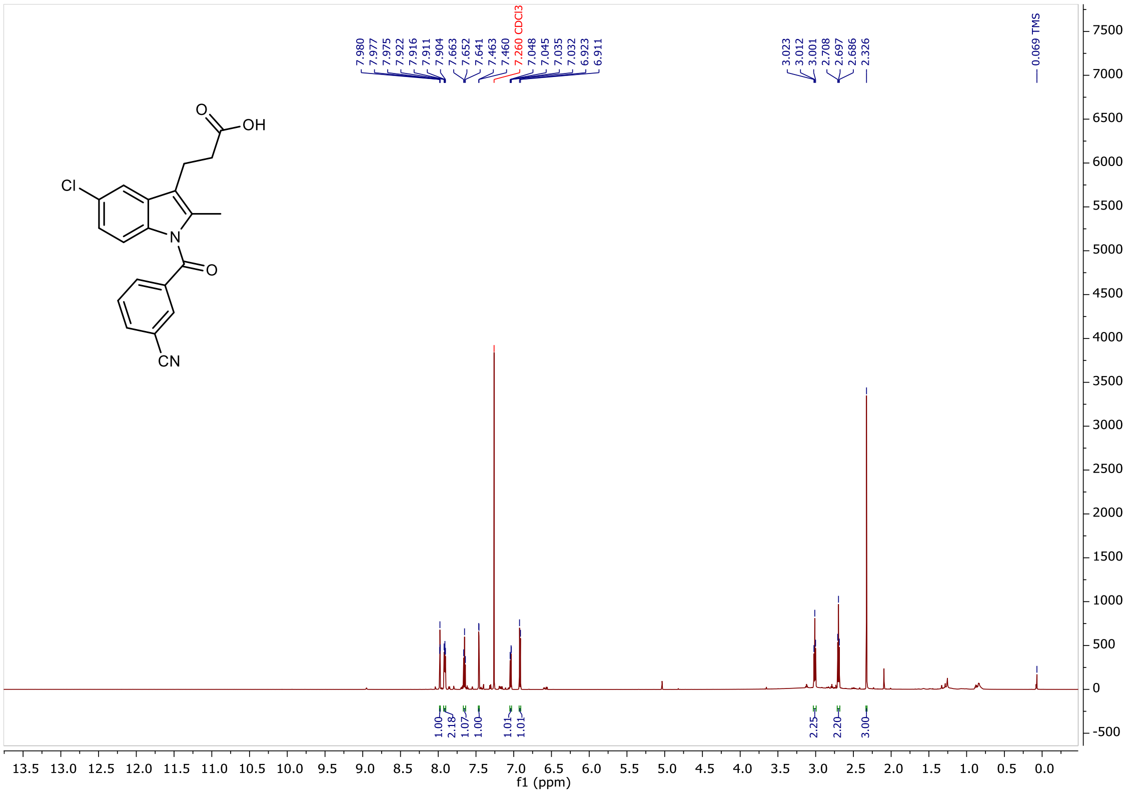

Supplementary Fig. 115. ^{13}C NMR of IA63 (176 MHz, $CDCl_3$)

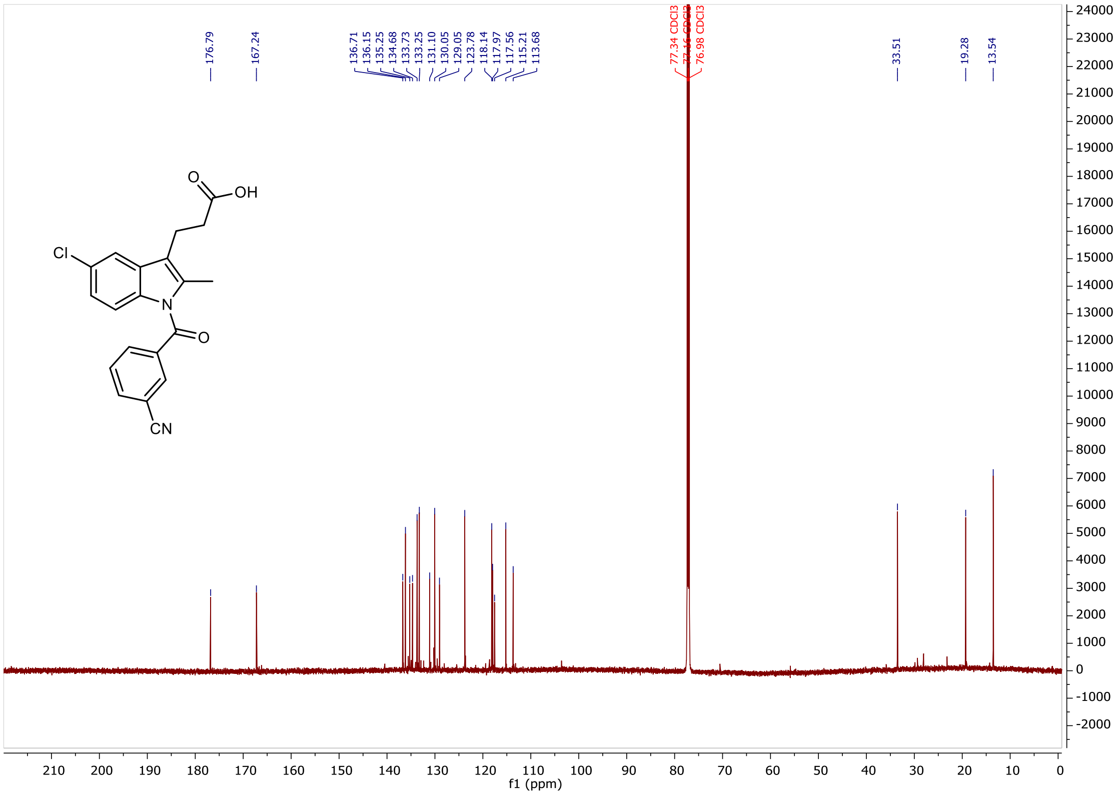

Supplementary Fig. 116. ^{1}H NMR of G-1749 (600 MHz, DMSO-d_6)

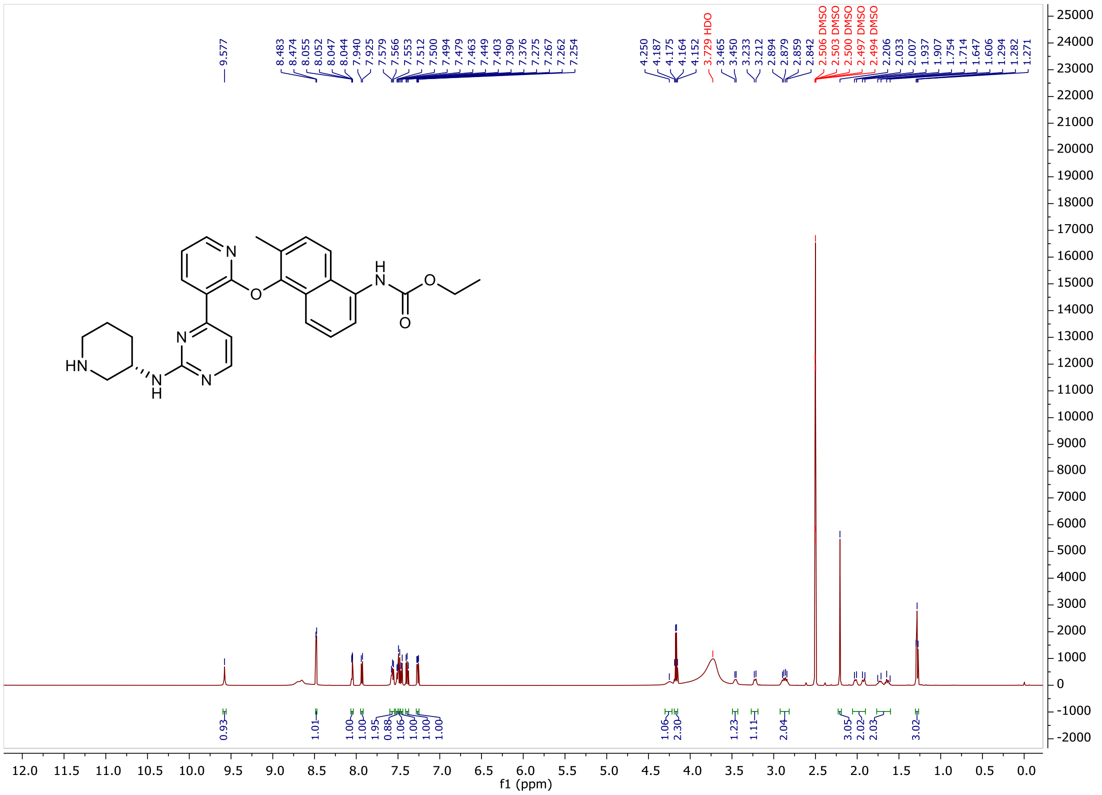

Supplementary Fig. 117. ^{13}C NMR of G-1749 (151 MHz, DMSO-d_6)

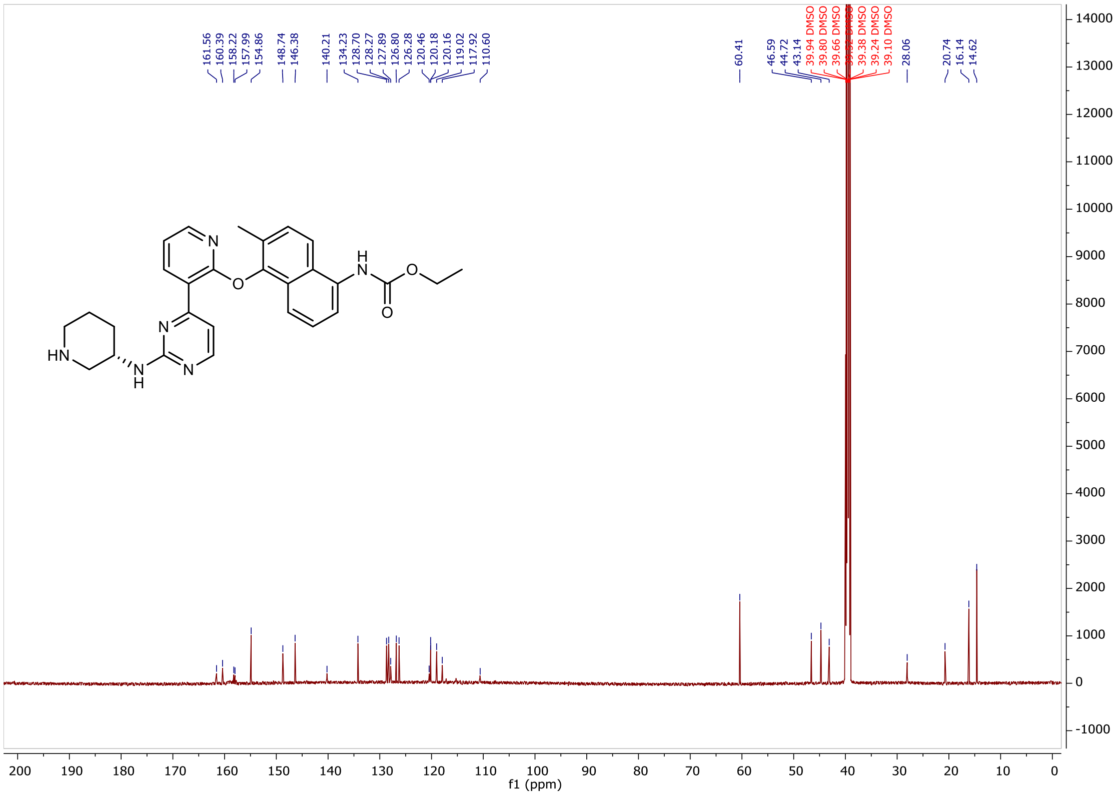

Supplementary Fig. 118. ^{1}H NMR of AMG-18 (600 MHz, DMSO-d_6)

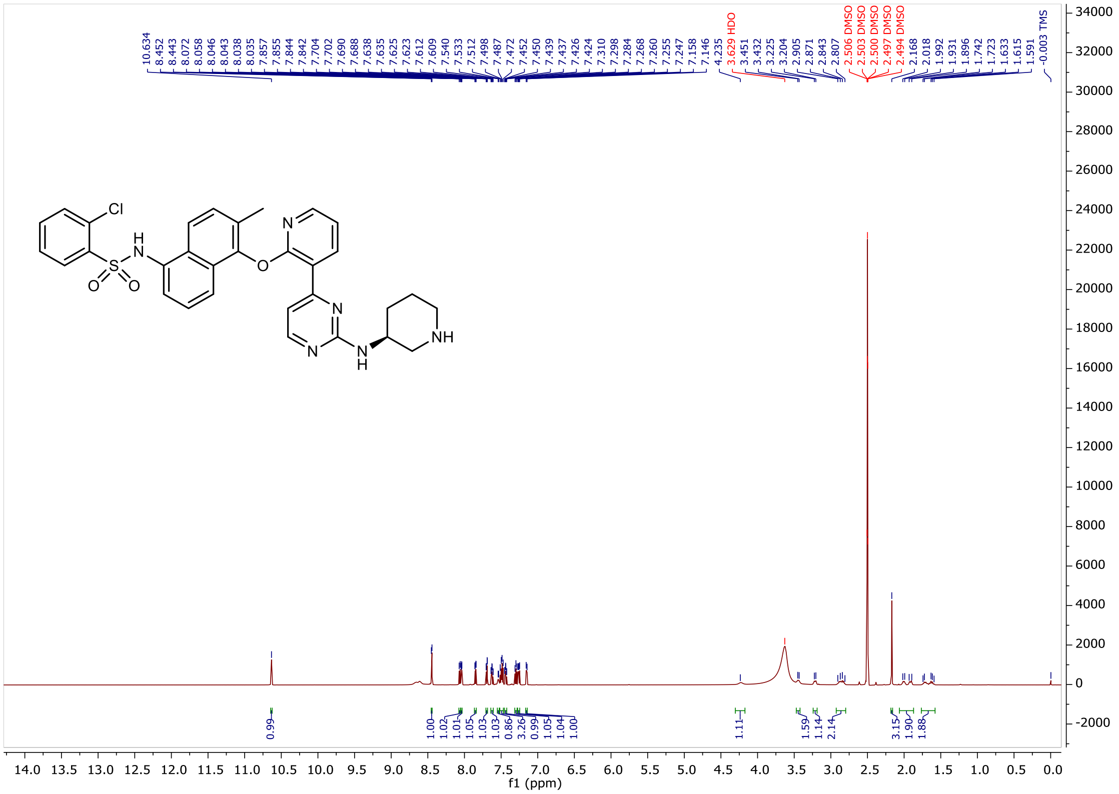

Supplementary Fig. 119. ^{13}C NMR of AMG-18 (151 MHz, DMSO-d_6)

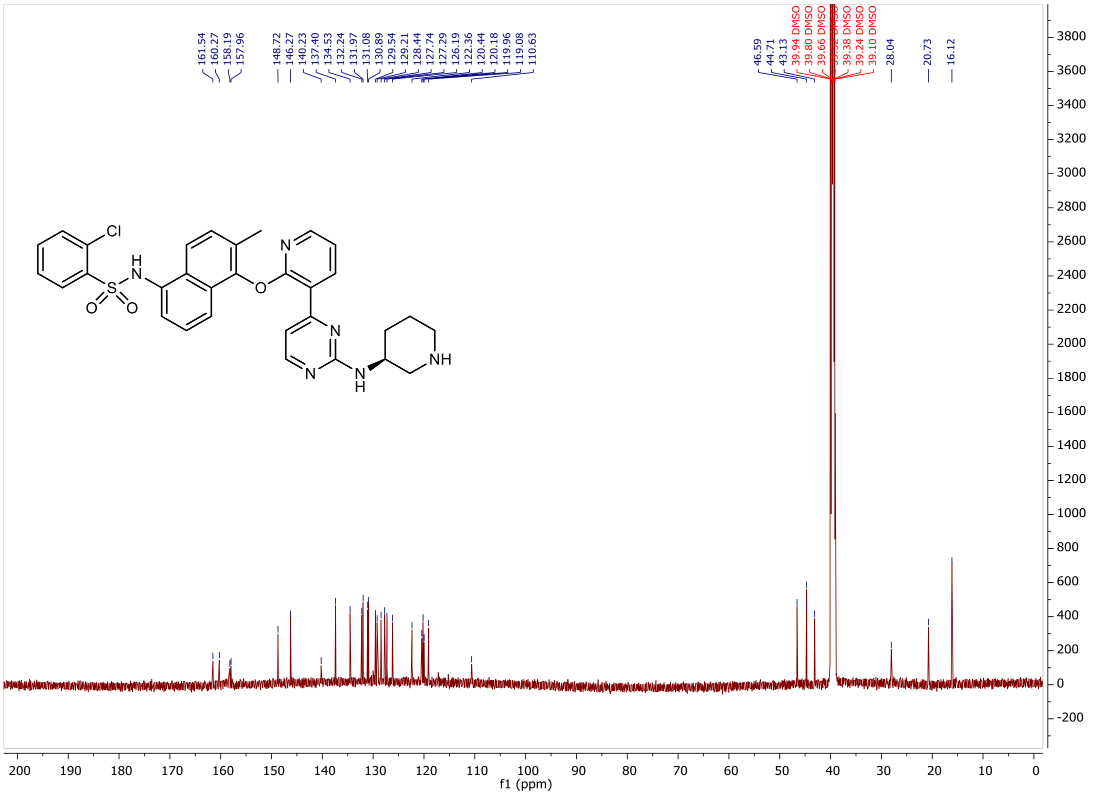

LC TRACES

Supplementary Fig. 120. LC of IA01 [Injection Acquisition Method C18_C1_10-100% (Water/Acetonitrile) _6min.amx]

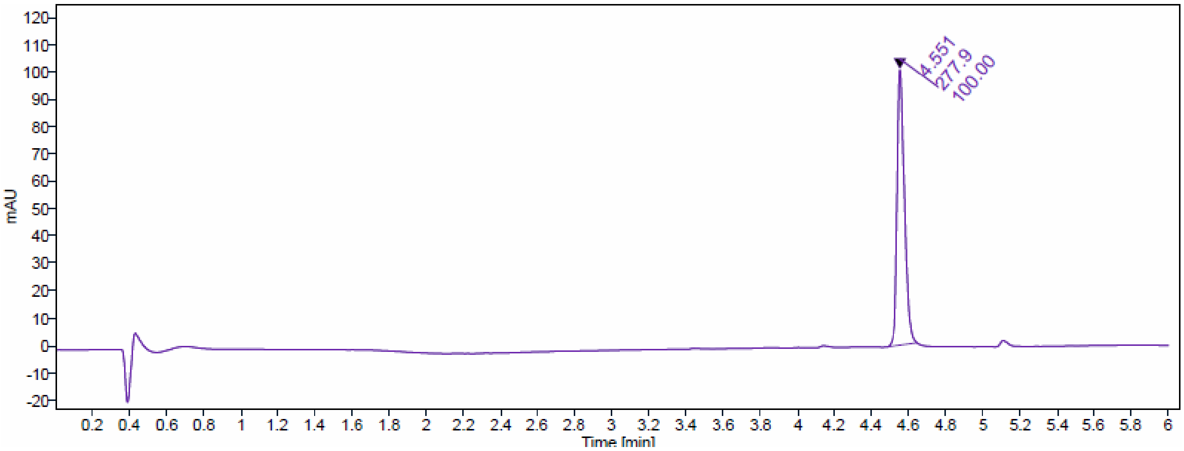

Supplementary Fig. 121. LC of IA03 [Injection Acquisition Method C18_C1_10-100% (Water/Acetonitrile) _6min.amx]

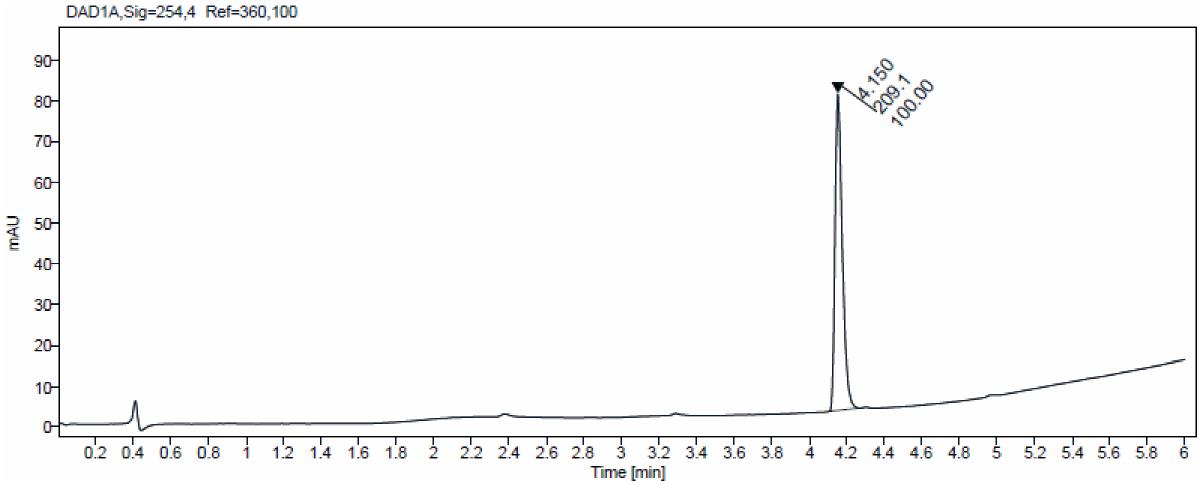

Supplementary Fig. 122. LC of IA06 [Injection Acquisition Method C18_C1_10-100% (Water/Acetonitrile) _6min.amx]

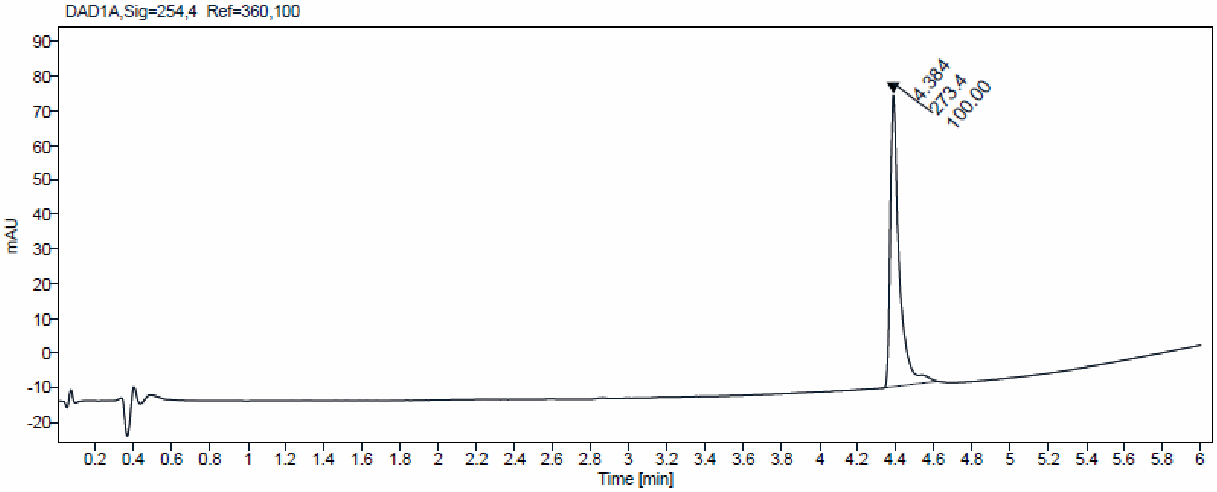

Supplementary Fig. 123. LC of IA10 [Injection Acquisition Method C18_C1_10-100% (Water/Acetonitrile) _6min.amx]

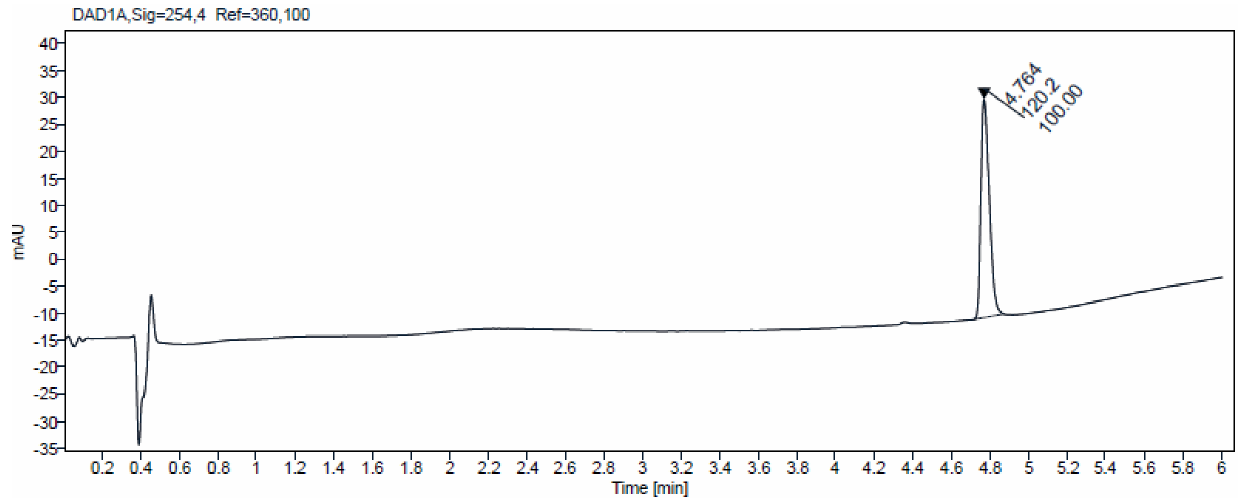

Supplementary Fig. 124. LC of IA30 [Injection Acquisition Method C18_C1_10-100% (Water/Acetonitrile) _6min.amx]

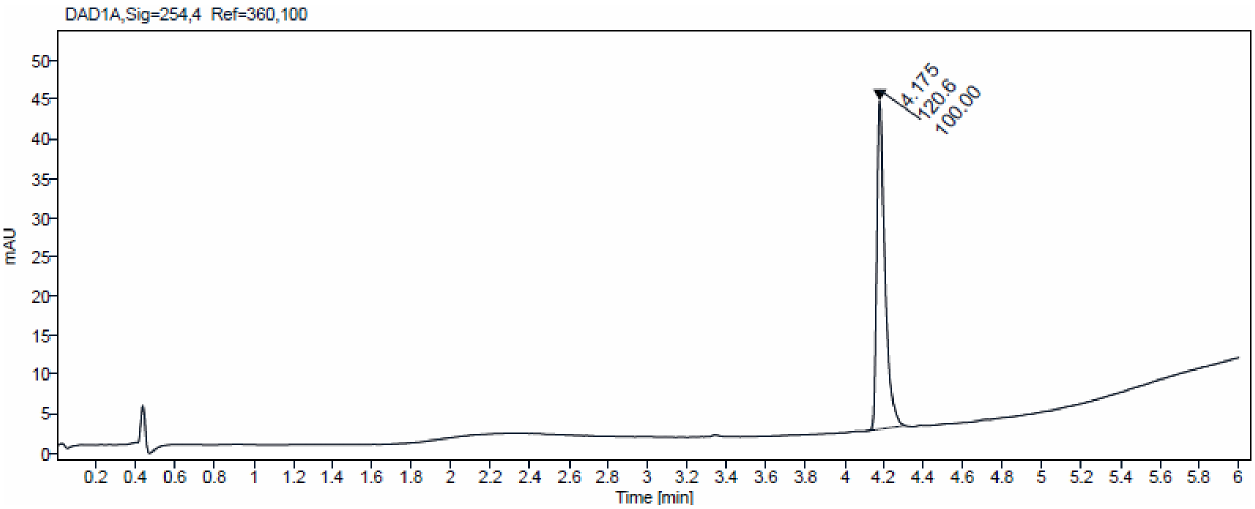

Supplementary Fig. 125. LC of IA32 [Injection Acquisition Method C18_C1_10-100% (Water/Acetonitrile) _6min.amx]

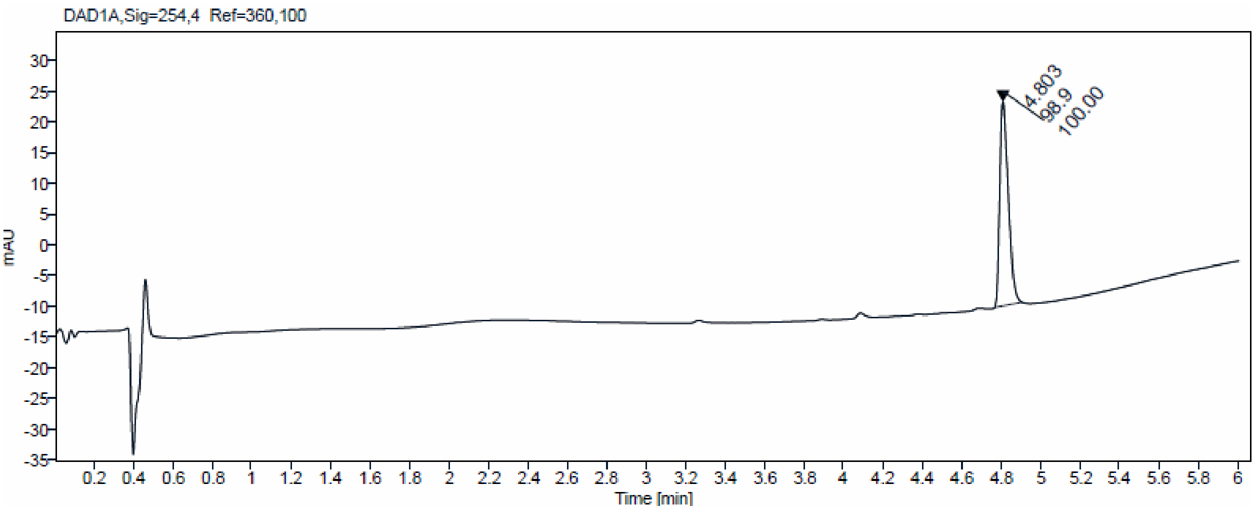

Supplementary Fig. 126. LC of IA34 [Injection Acquisition Method C18_C1_10-100% (Water/Acetonitrile) _6min.amx]

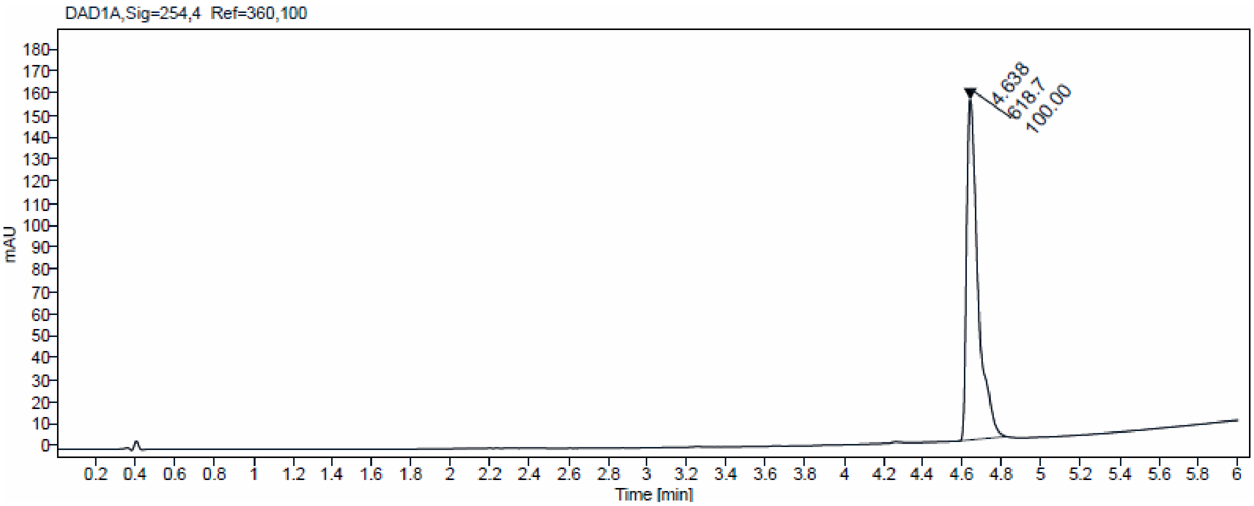

Supplementary Fig. 127. LC of IA36 [Injection Acquisition Method C18_C1_10-100% (Water/Acetonitrile) _6min.amx]

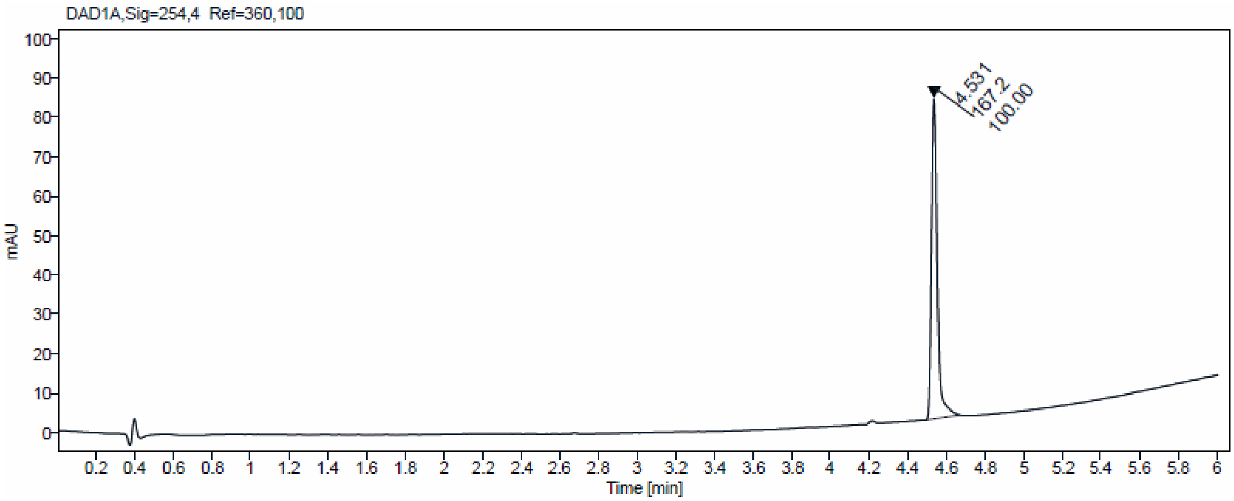

Supplementary Fig. 128. LC of IA64 [Injection Acquisition Method C18_C1_10-100% (Water/Acetonitrile) _6min.amx]

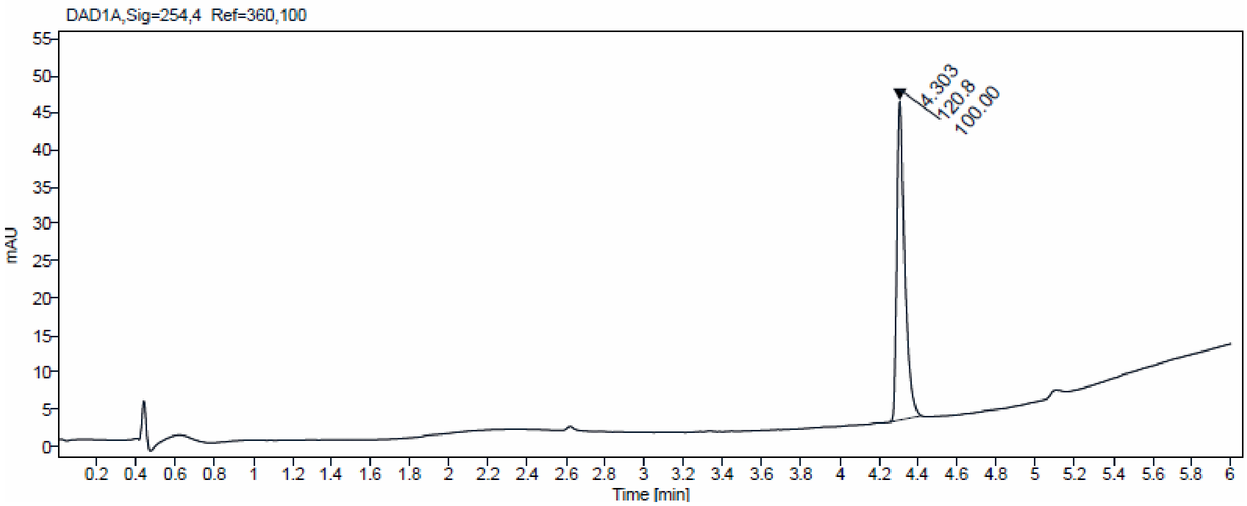

Supplementary Fig. 129. LC of IA66 [Injection Acquisition Method C18_C1_10-100% (Water/Acetonitrile) _6min.amx]

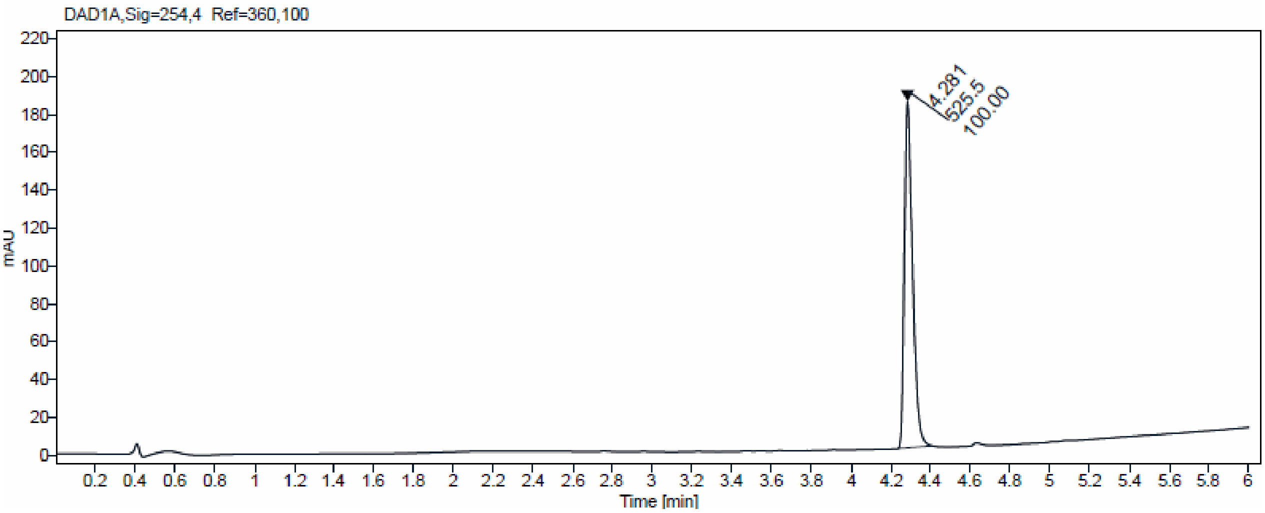

Supplementary Fig. 130. LC of IA70 [Injection Acquisition Method C18_C1_10-100% (Water/Acetonitrile) _6min.amx]

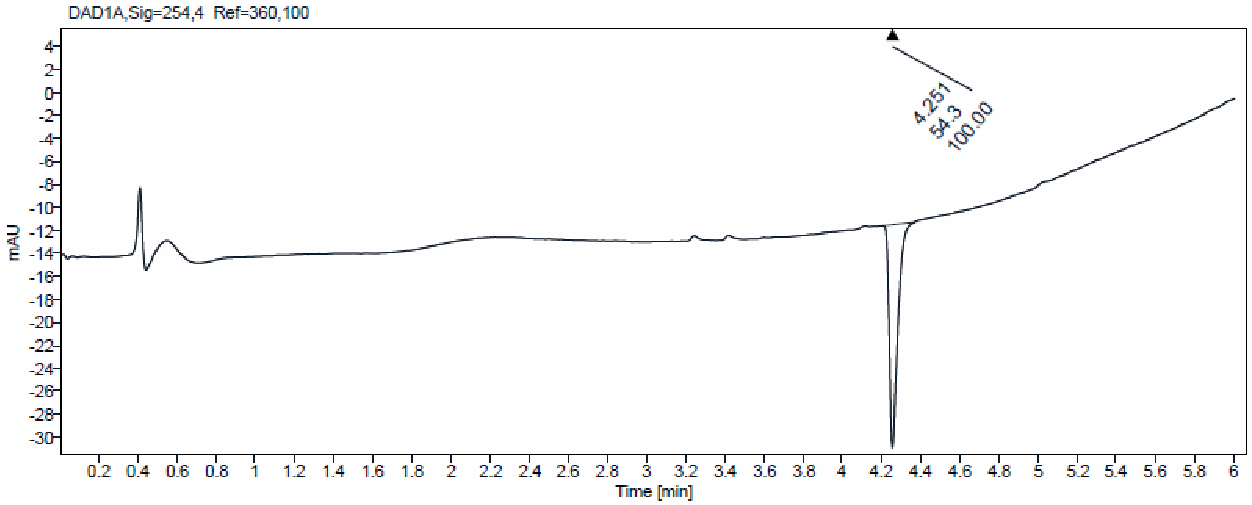

Supplementary Fig. 131. LC of IA72 [Injection Acquisition Method C18_C1_10-100% (Water/Acetonitrile) _6min.amx]

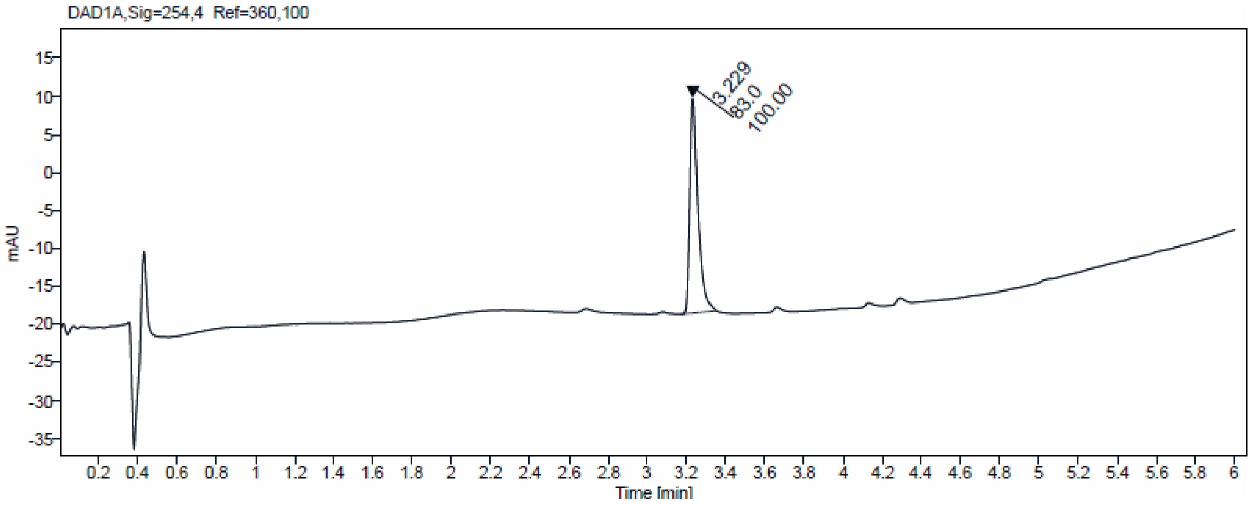

Supplementary Fig. 132. LC of IA75 [Injection Acquisition Method C18_C1_10-100% (Water/Acetonitrile) _6min.amx]

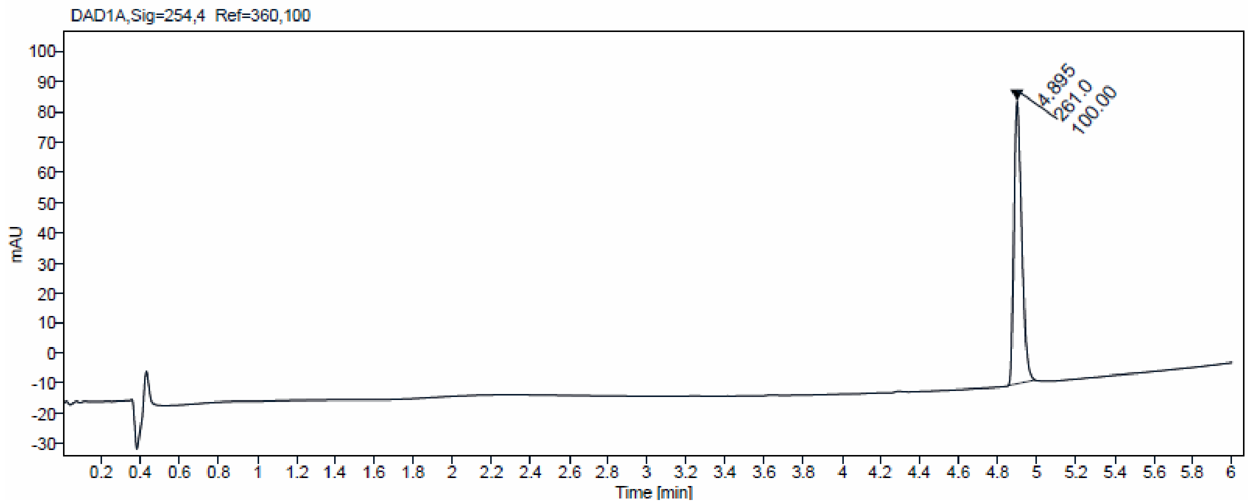

Supplementary Fig. 133. LC of IA76 [Injection Acquisition Method C18_C1_10-100% (Water/Acetonitrile) _6min.amx]

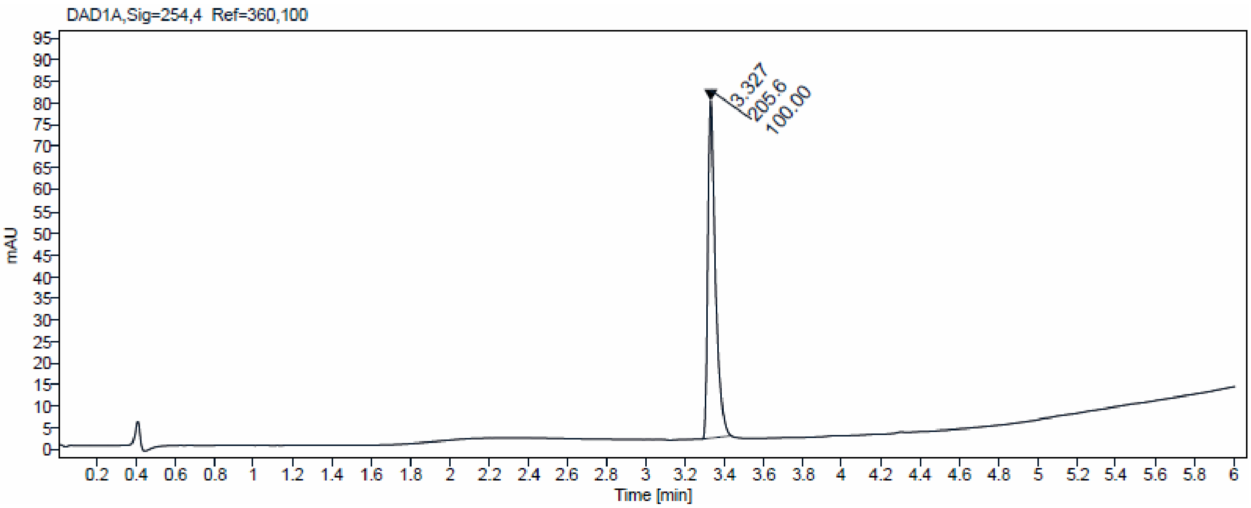

Supplementary Fig. 134. LC of IA77 [Injection Acquisition Method C18_C1_10-100% (Water/Acetonitrile) _6min.amx]

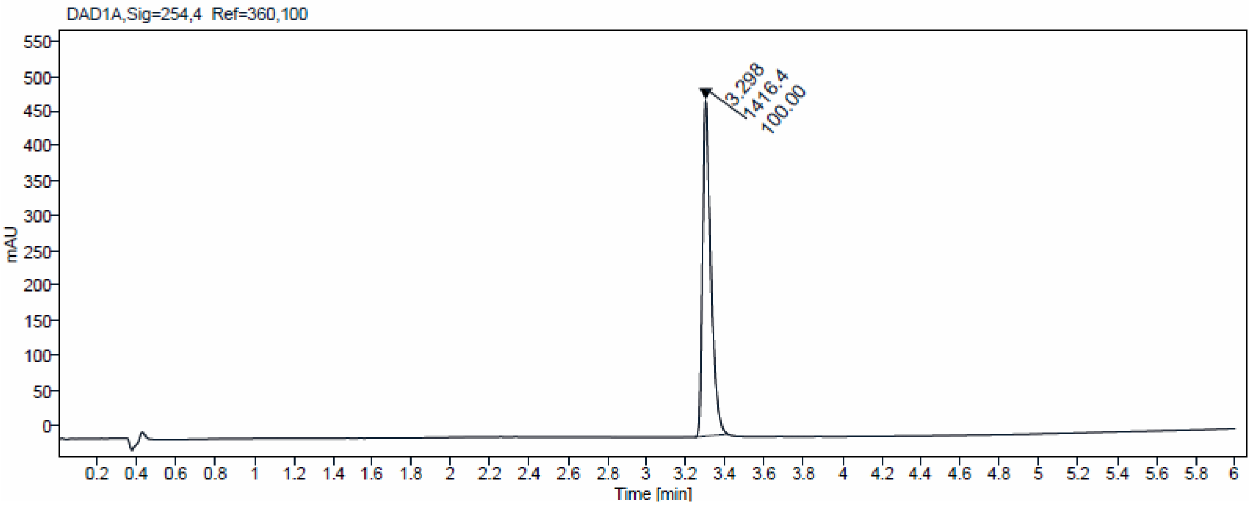

Supplementary Fig. 135. LC of IA81 [Injection Acquisition Method C18_C1_10-100% (Water/Acetonitrile) _6min.amx]

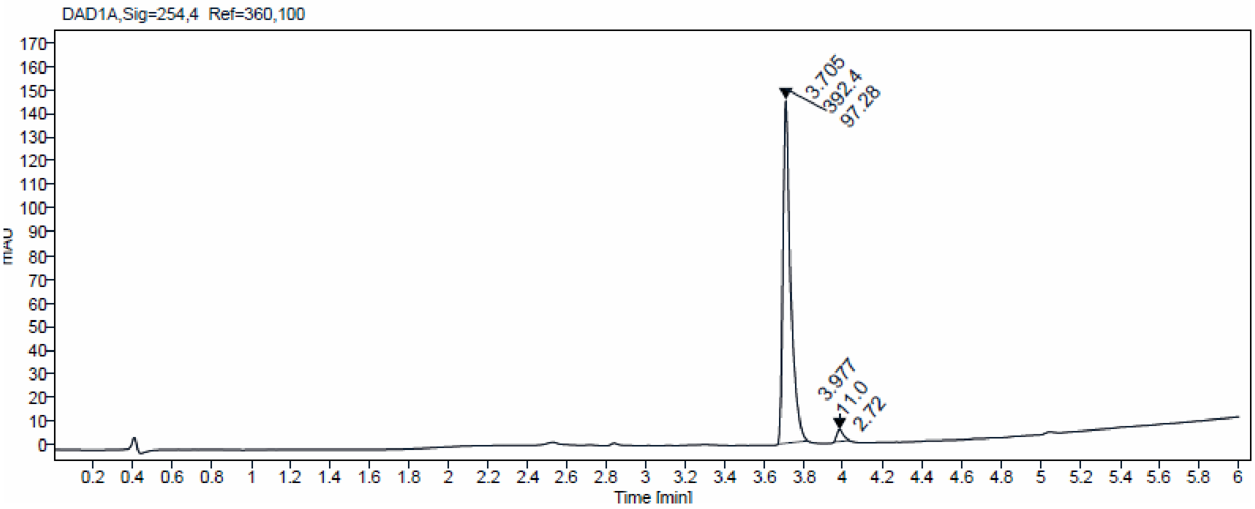

Supplementary Fig. 136. LC of IA84 [Injection Acquisition Method C18_C1_10-100% (Water/Acetonitrile) _6min.amx]

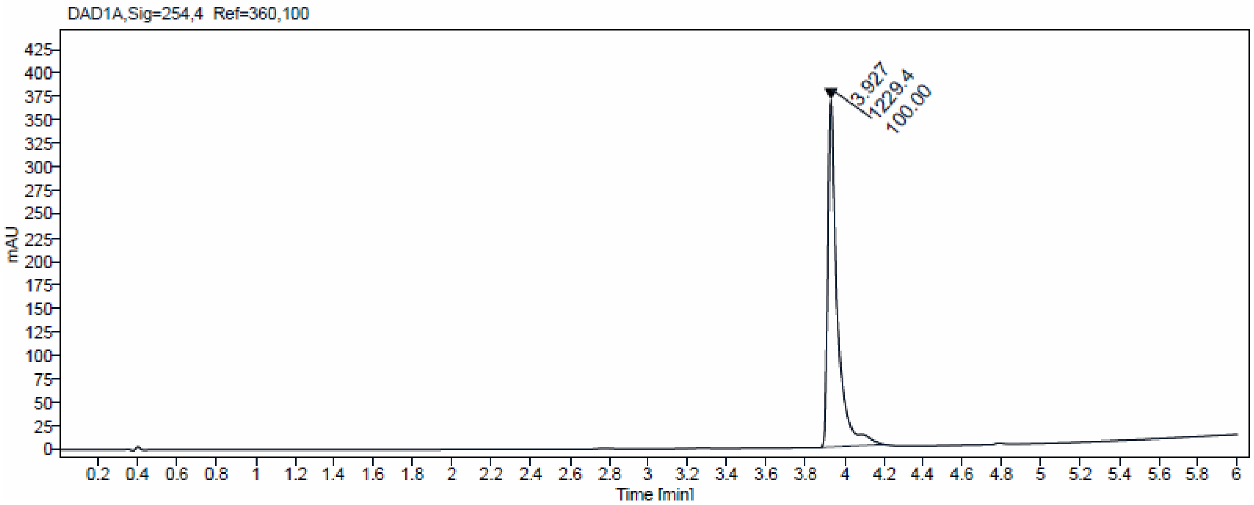

Supplementary Fig. 137. LC of IA89 [Injection Acquisition Method C18_C1_10-100% (Water/Acetonitrile) _6min.amx]

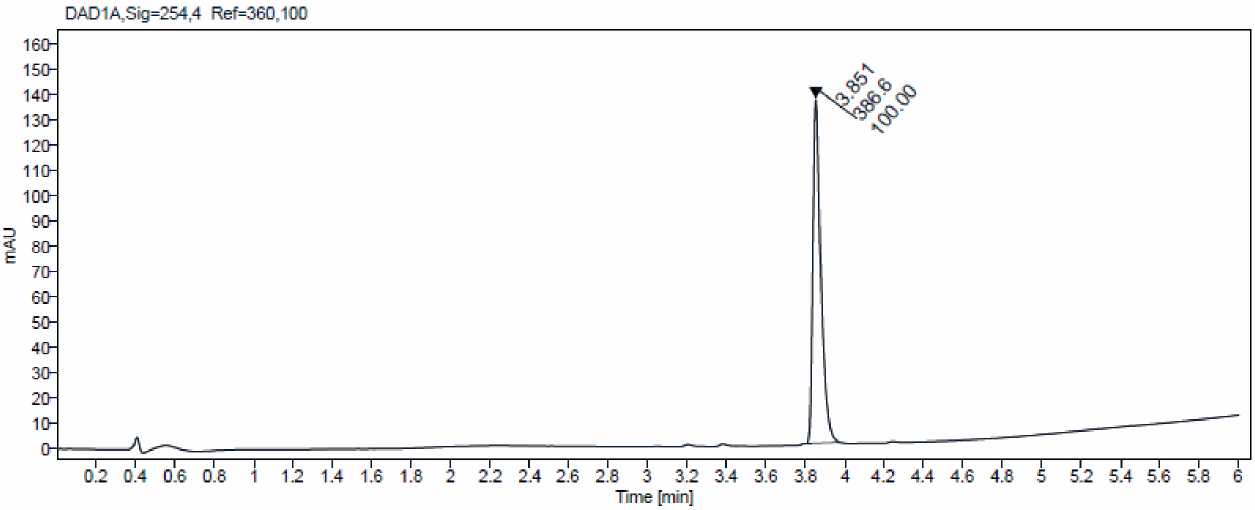

Supplementary Fig. 138. LC of IA92 [Injection Acquisition Method C18_C1_10-100% (Water/Acetonitrile) _6min.amx]

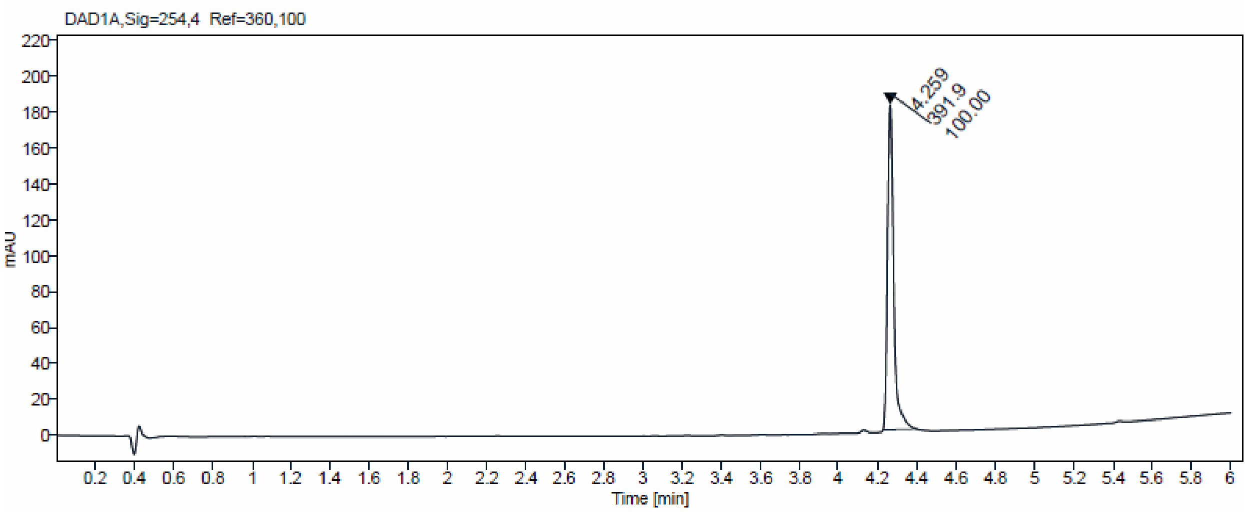

Supplementary Fig. 139. LC of IA93 [Injection Acquisition Method C18_C1_10-100% (Water/Acetonitrile) _6min.amx]

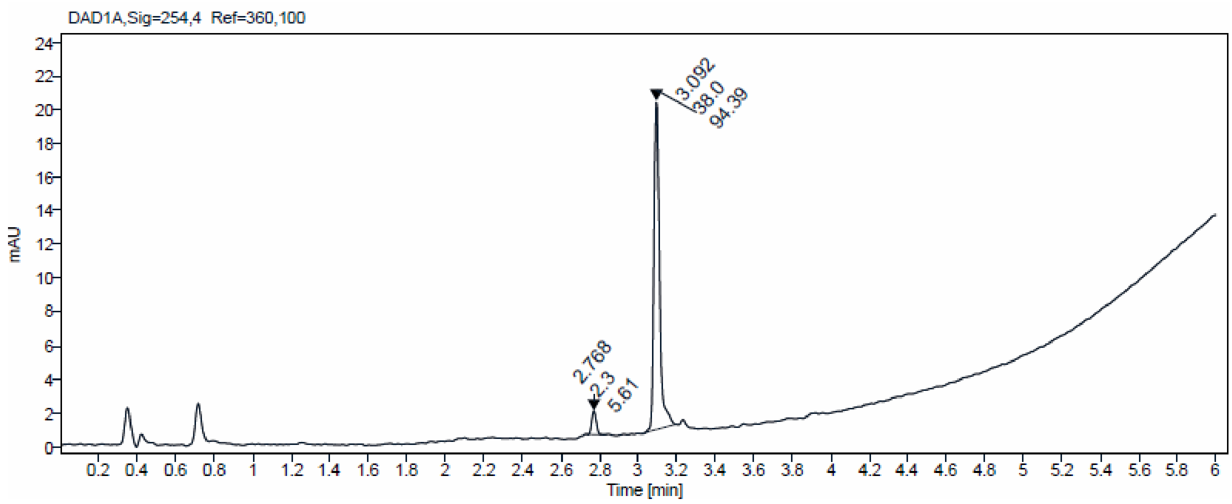

Supplementary Fig. 140. LC of IA96 [Injection Acquisition Method C18_C1_10-100% (Water/Acetonitrile) _6min.amx]

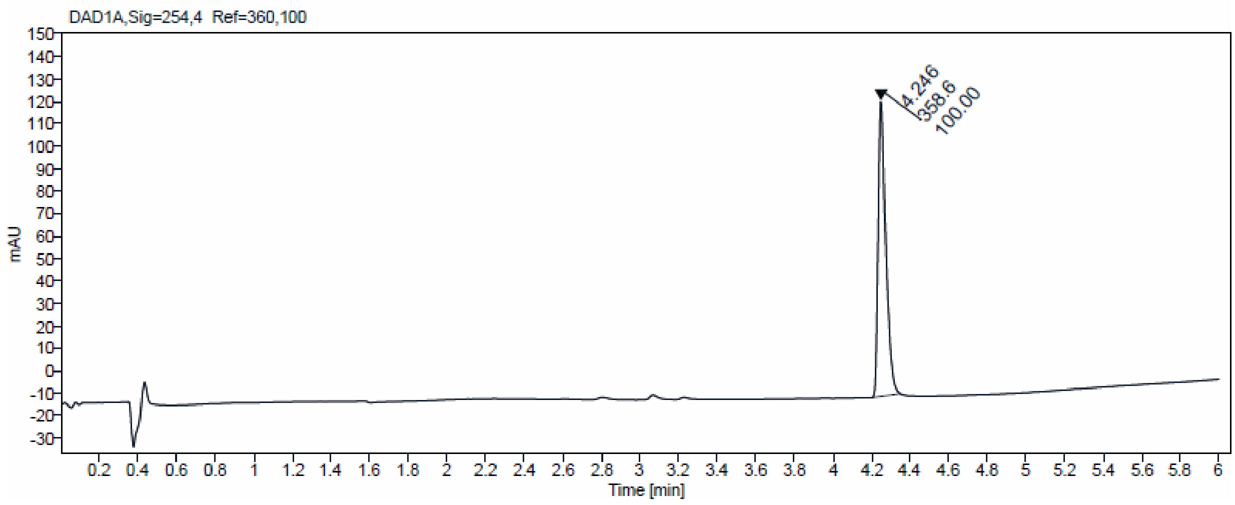

Supplementary Fig. 141. LC of IA104 [Injection Acquisition Method C18_C1_10-100% (Water/Acetonitrile) _6min.amx]

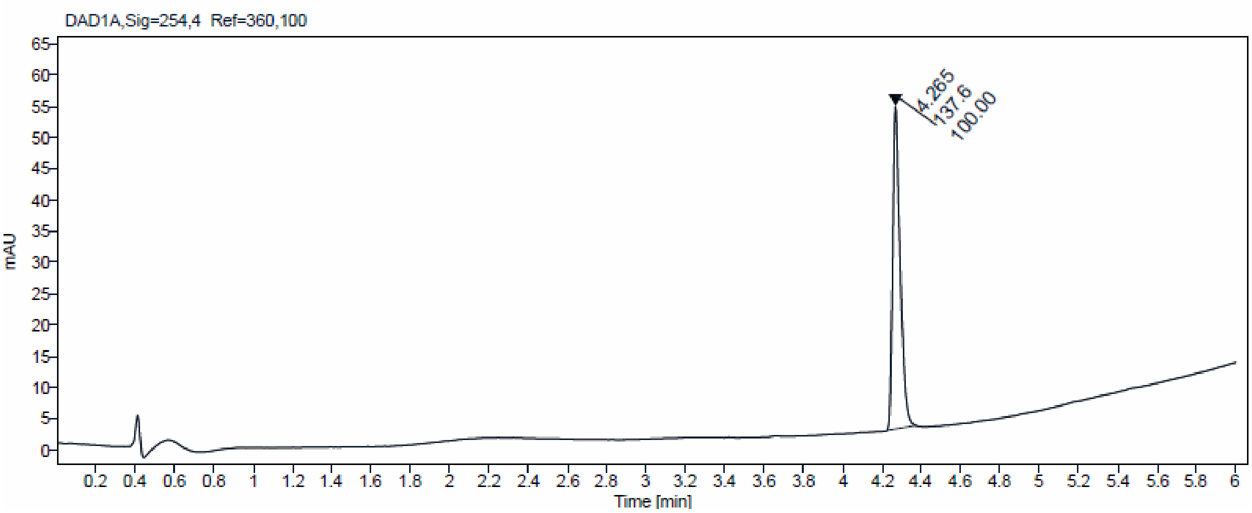

Supplementary Fig. 142. LC of IA107 [Injection Acquisition Method C18_C1_10-100% (Water/Acetonitrile) _6min.amx]

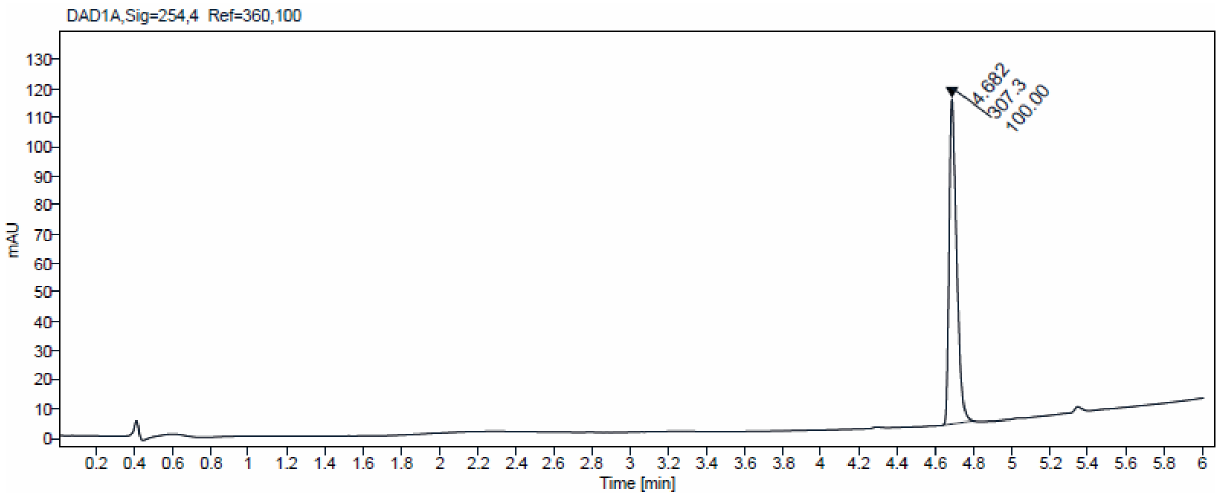

Supplementary Fig. 143. LC of IA110 [Injection Acquisition Method C18_C1_10-100% (Water/Acetonitrile) _6min.amx]

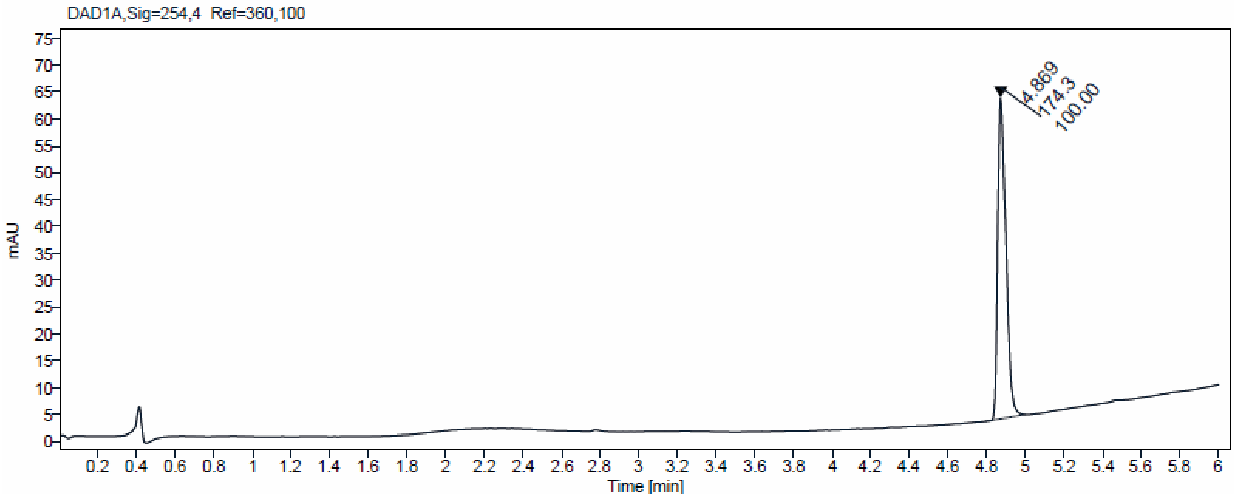

Supplementary Fig. 144. LC of IA137 [Injection Acquisition Method C18_C1_10-100% (Water/Acetonitrile) _6min.amx]

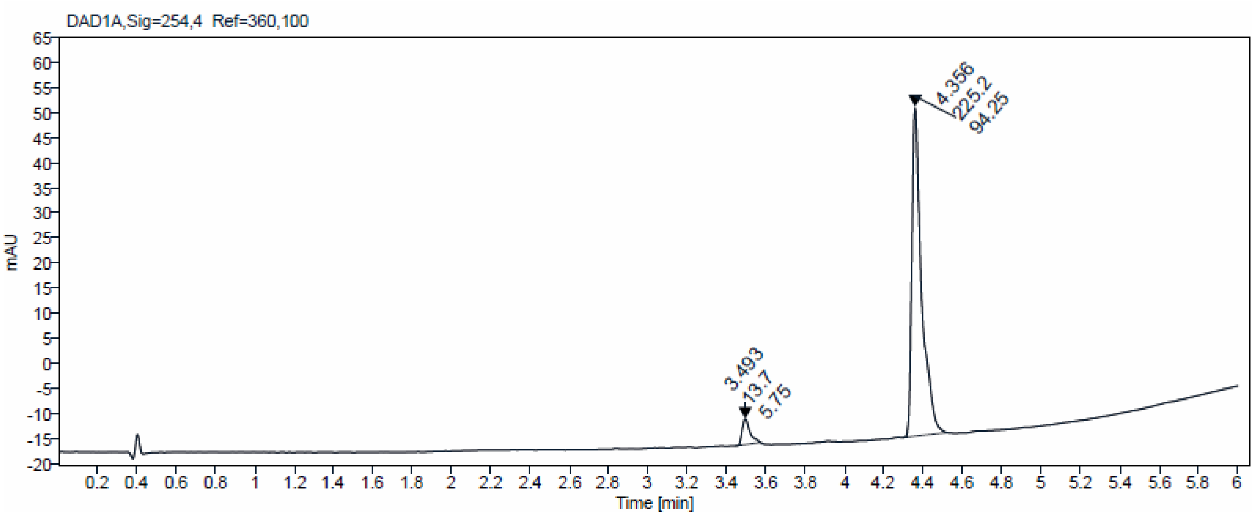

Supplementary Fig. 145. LC of IA138 [Injection Acquisition Method C18_C2_5-95% (Water/Acetonitrile) _20min.amx]

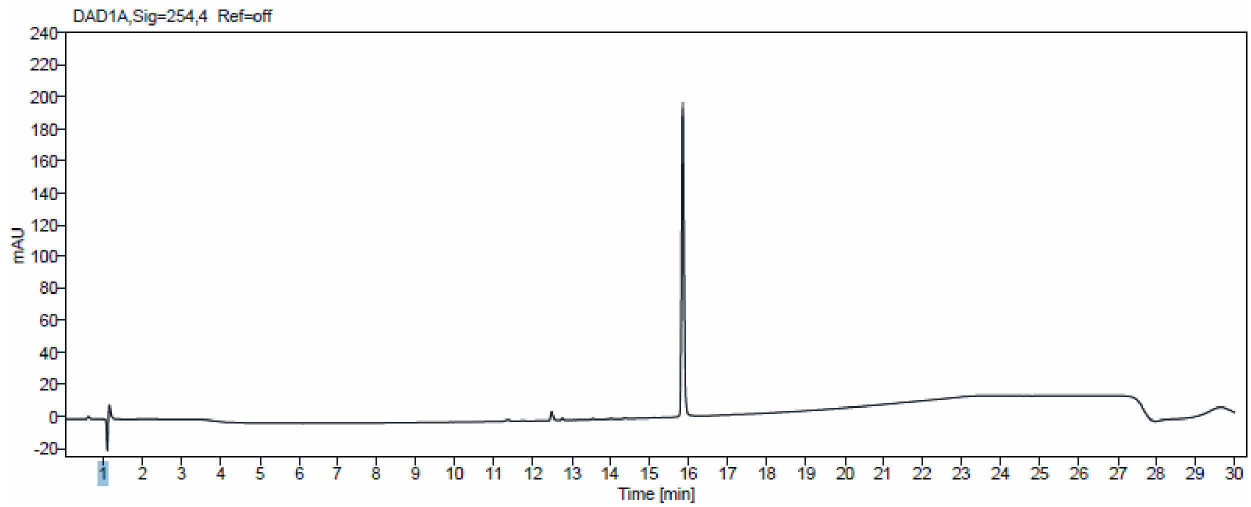

Supplementary Fig. 146. LC of IA140 [Injection Acquisition Method C18_C2_5-95% (Water/Acetonitrile) _20min.amx]

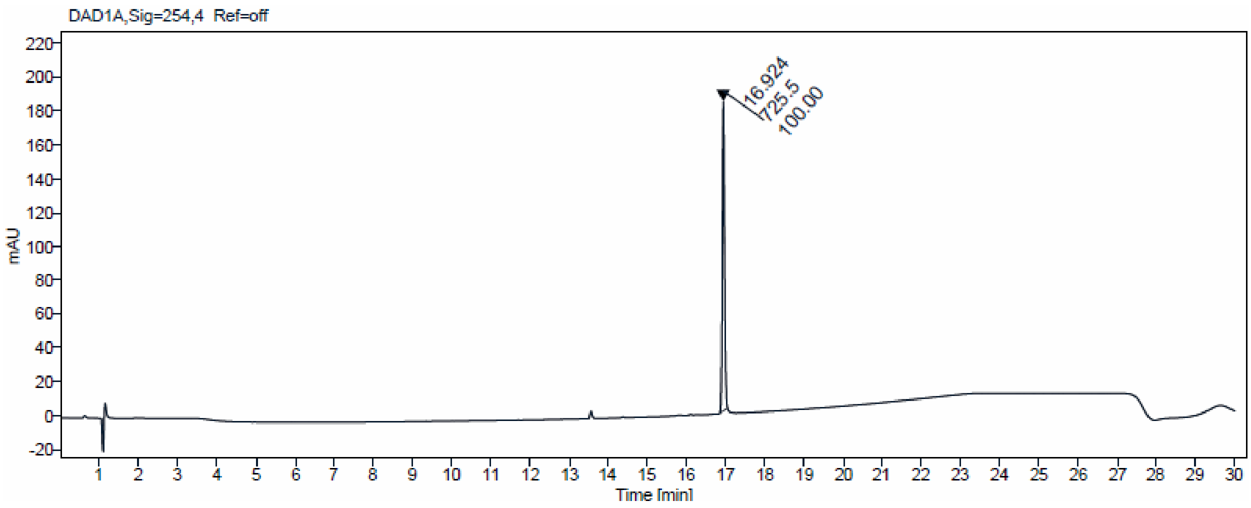

Supplementary Fig. 147. LC of IA141 [Injection Acquisition Method C18_C1_10-100% (Water/Acetonitrile) _6min.amx]

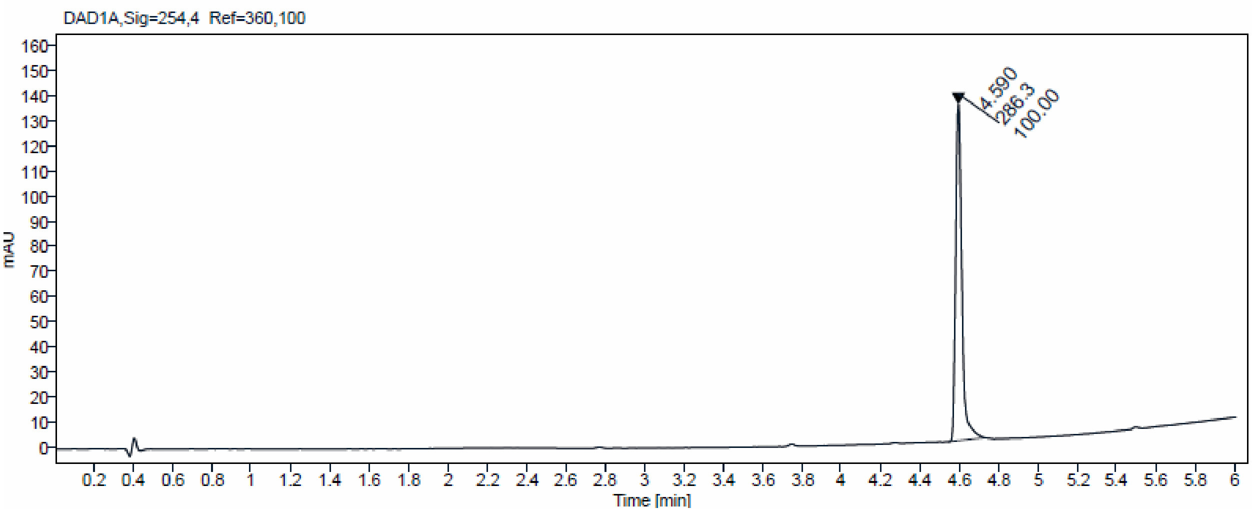

Supplementary Fig. 148. LC of IA142 [Injection Acquisition Method C18_C1_10-100% (Water/Acetonitrile) _6min.amx]

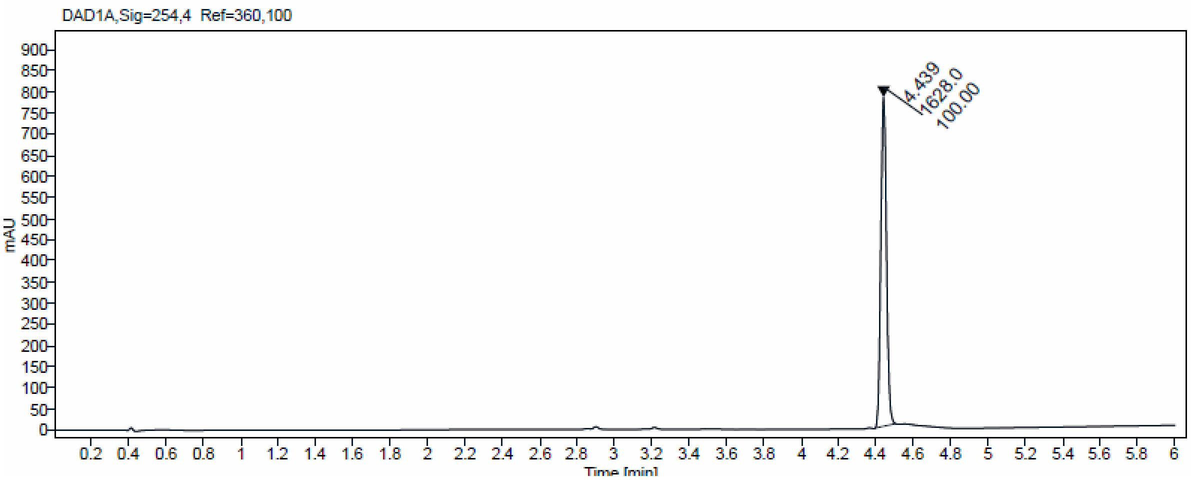

Supplementary Fig. 149. LC of IA143 [Injection Acquisition Method C18_C1_10-100% (Water/Acetonitrile) _6min.amx]

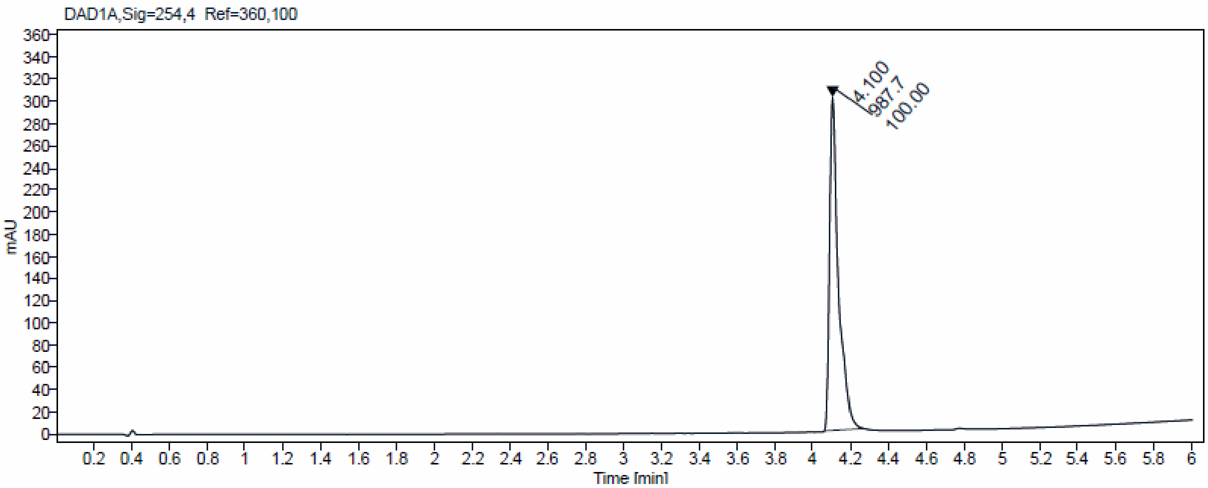

Supplementary Fig. 150. LC of IA145 [Injection Acquisition Method C18_C1_10-100% (Water/Acetonitrile) _6min.amx]

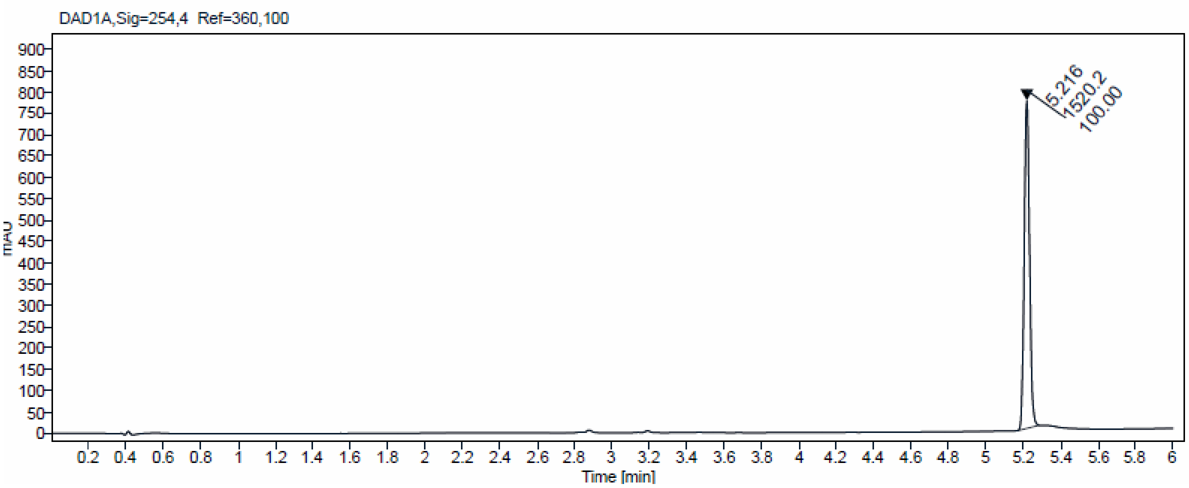

Supplementary Fig. 151. LC of IA146 [Injection Acquisition Method C18_C1_10-100% (Water/Acetonitrile) _6min.amx]

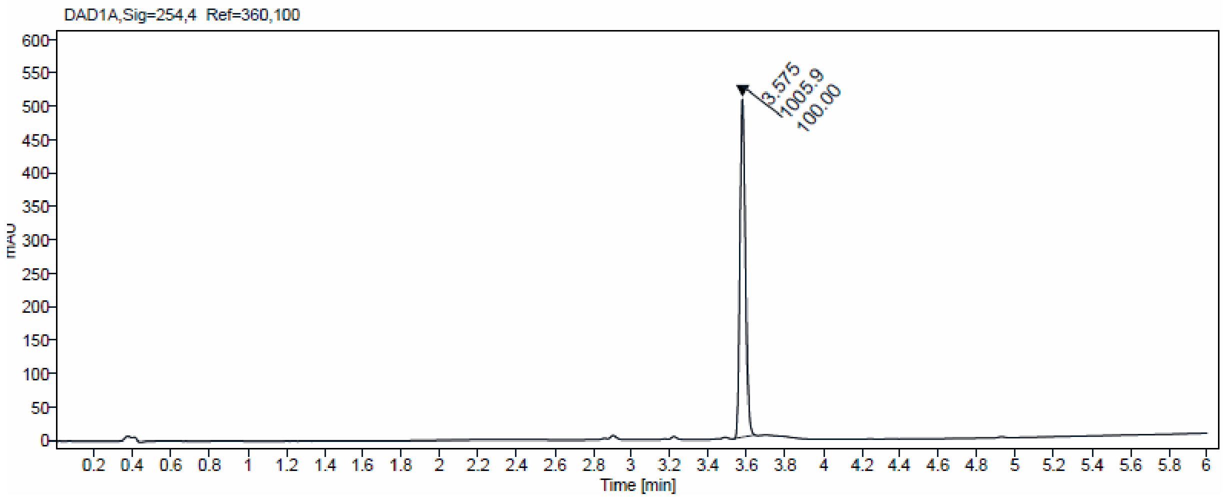

Supplementary Fig. 152. LC of IA147 [Injection Acquisition Method C18_C1_10-100% (Water/Acetonitrile) _6min.amx]

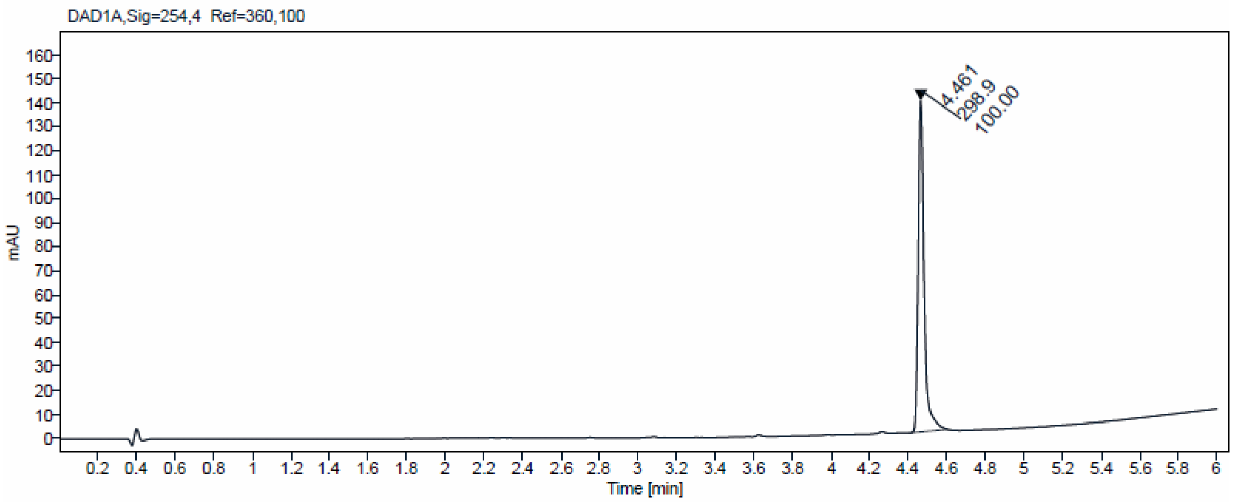

Supplementary Fig. 153. LC of IA148 [Injection Acquisition Method C18_C1_10-100% (Water/Acetonitrile) _6min.amx]

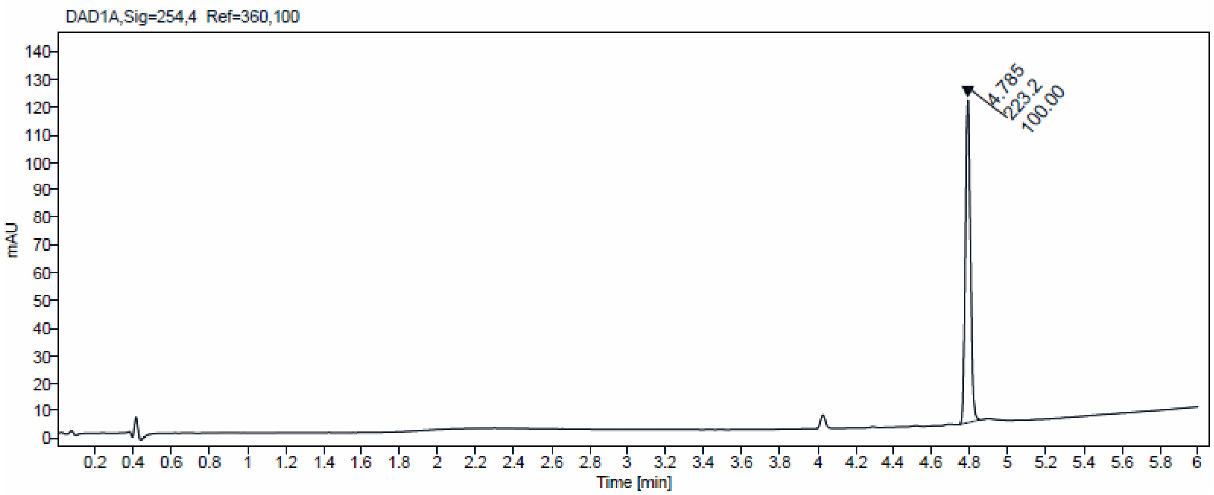

Supplementary Fig. 154. LC of IA149 [Injection Acquisition Method C18_C2_5-95% (Water/Acetonitrile) _20min.amx]

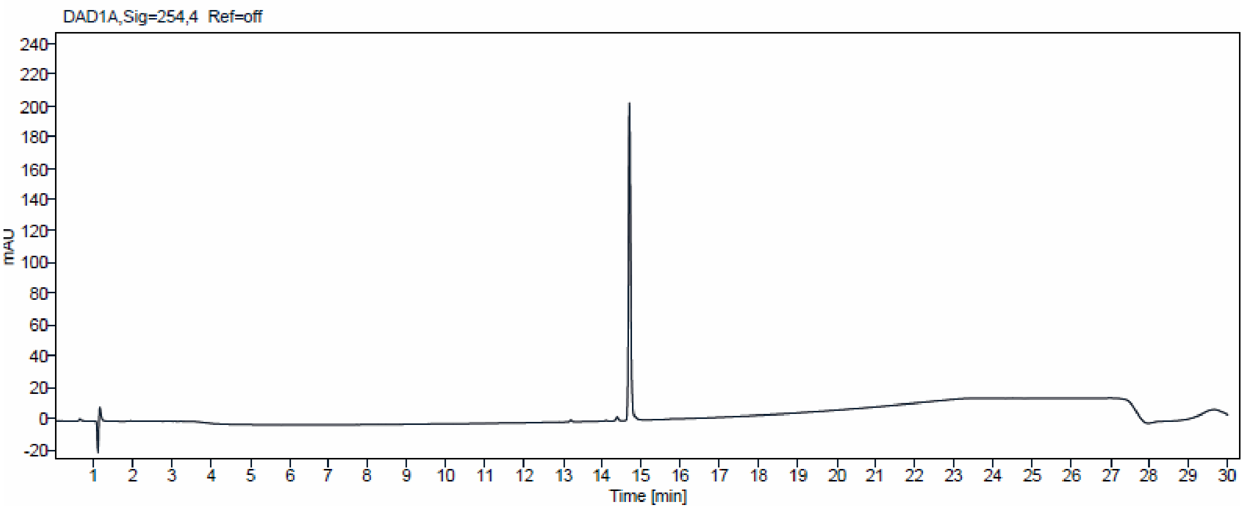

Supplementary Fig. 155. LC of IA152 [Injection Acquisition Method C18_C1_10-100% (Water/Acetonitrile) _6min.amx]

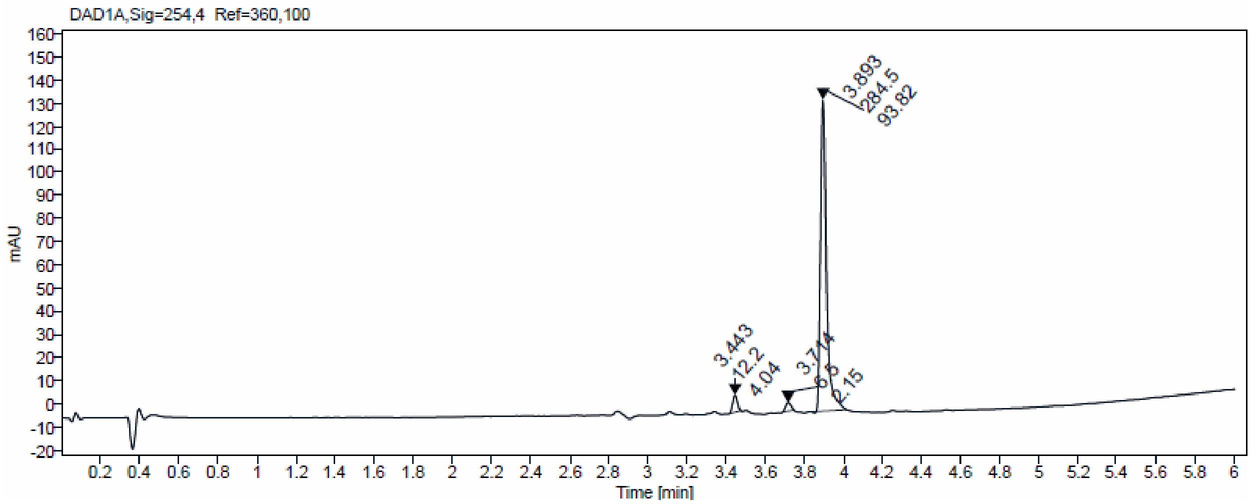

Supplementary Fig. 156. LC of IA153 [Injection Acquisition Method C18_C1_10-100% (Water/Acetonitrile) _6min.amx]

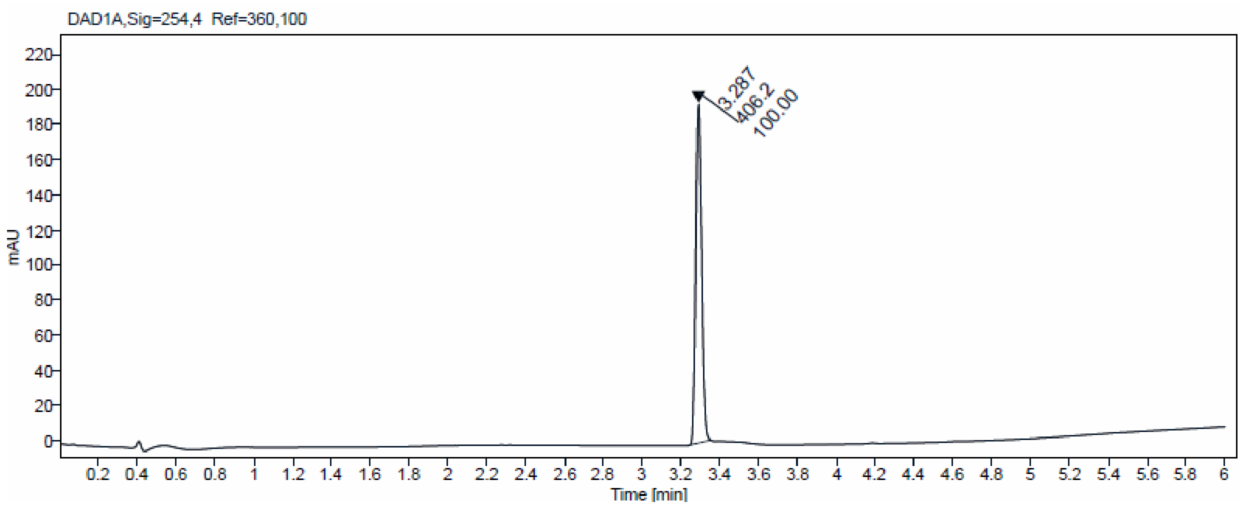

Supplementary Fig. 157. LC of IA158 [Injection Acquisition Method C18_C1_10-100% (Water/Acetonitrile) _6min.amx]

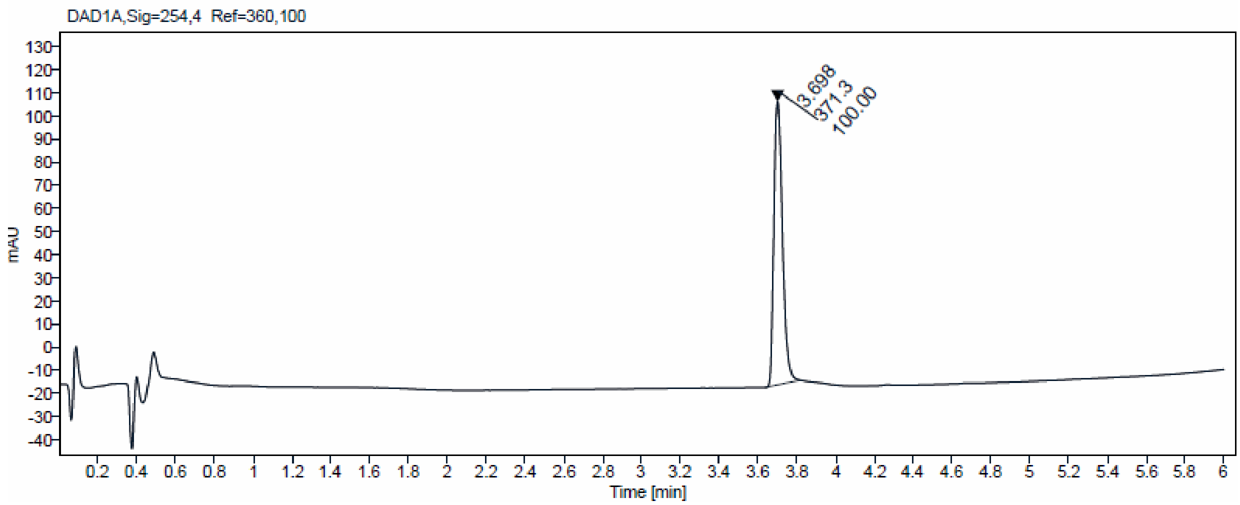

Supplementary Fig. 158. LC of IA159 [Injection Acquisition Method C18_C1_10-100% (Water/Acetonitrile) _6min.amx]

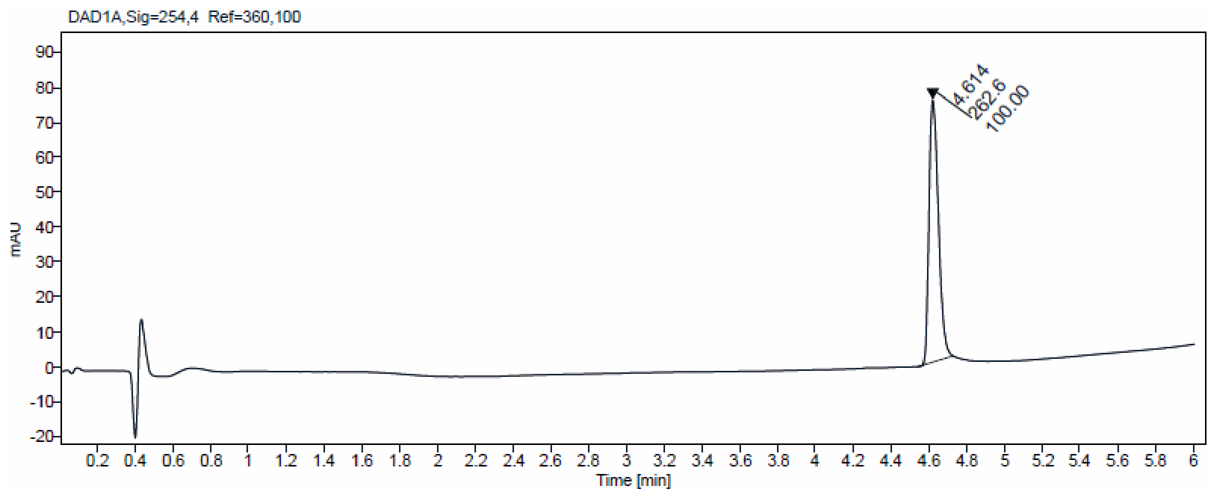

Supplementary Fig. 159. LC of IA162 [Injection Acquisition Method C18_C1_10-100% (Water/Acetonitrile) _6min.amx]

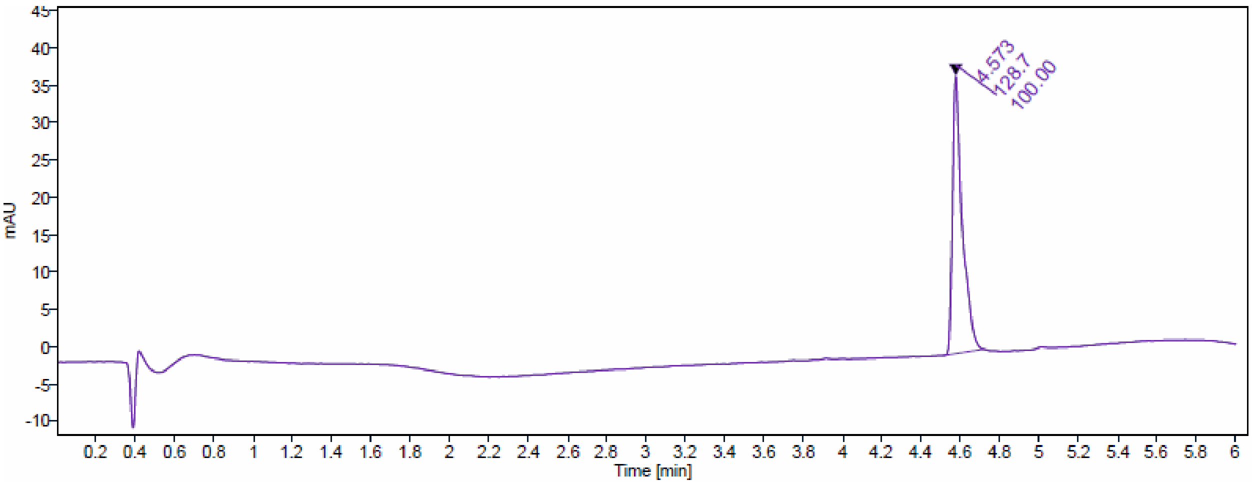

Supplementary Fig. 160. LC of IA163 [Injection Acquisition Method C18_C1_10-100% (Water/Acetonitrile) _6min.amx]

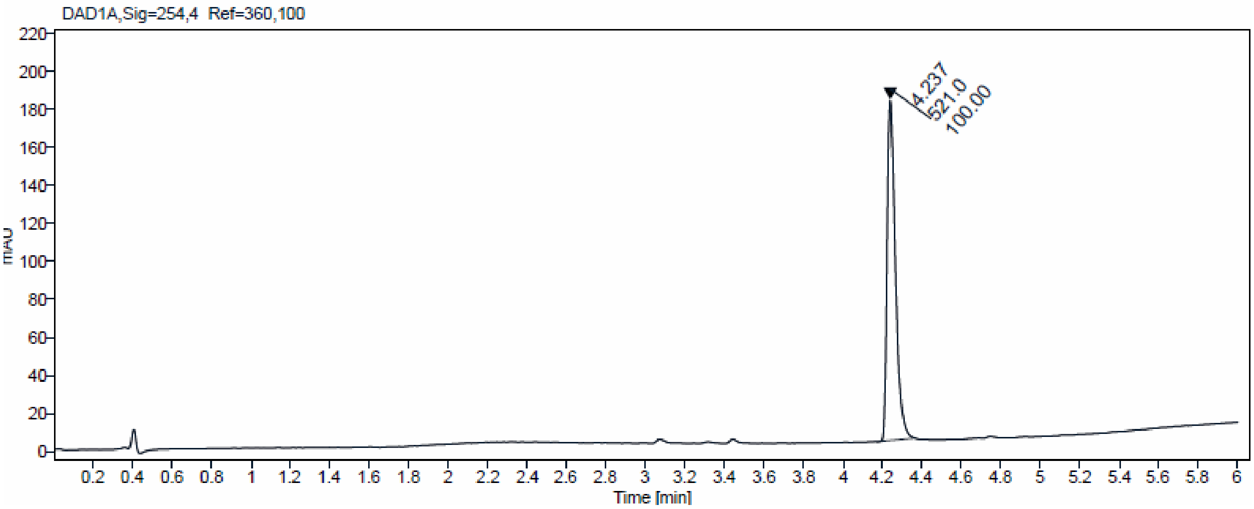

Supplementary Fig. 161. LC of IA216 [Injection Acquisition Method C18_C2_5-95% (Water/Acetonitrile) _20 min.amx]

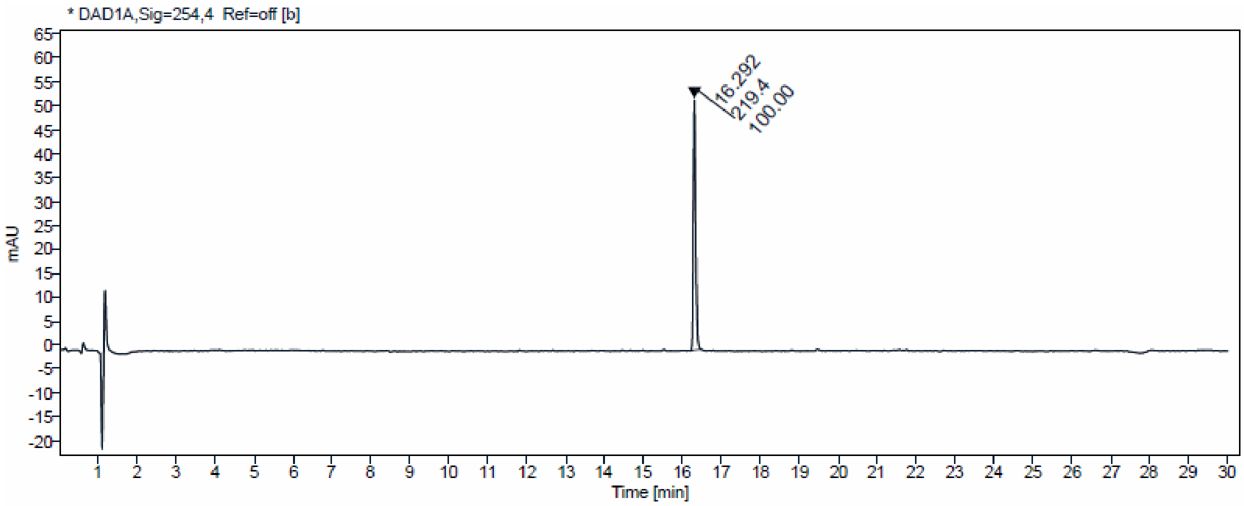

Supplementary Fig. 162. LC of IA217 [Injection Acquisition Method C18_C2_5-95% (Water/Acetonitrile) _20min.amx]

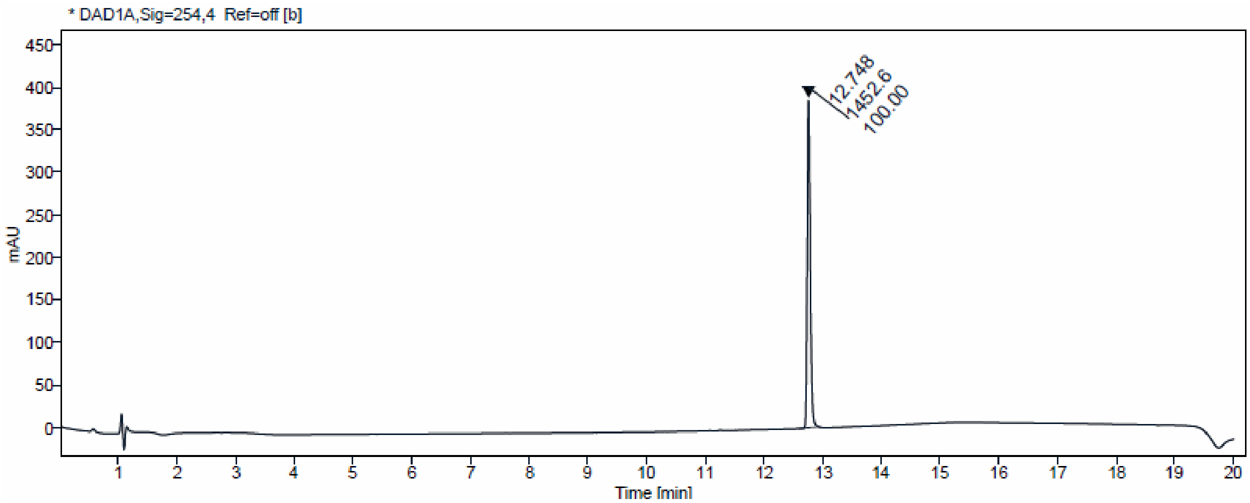

Supplementary Fig. 163. LC of IA41 [Injection Acquisition Method C18_C1_10-100% (Water/Acetonitrile) _6min.amx]

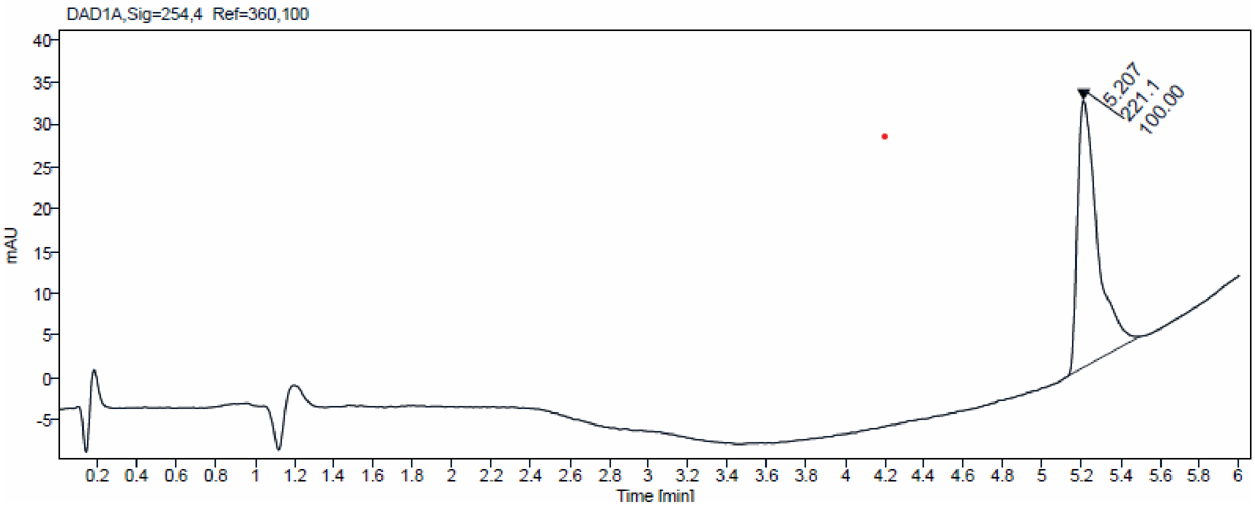

Supplementary Fig. 164. LC of IA42 [Injection Acquisition Method C18_C1_10-100% (Water/Acetonitrile) _6min.amx]

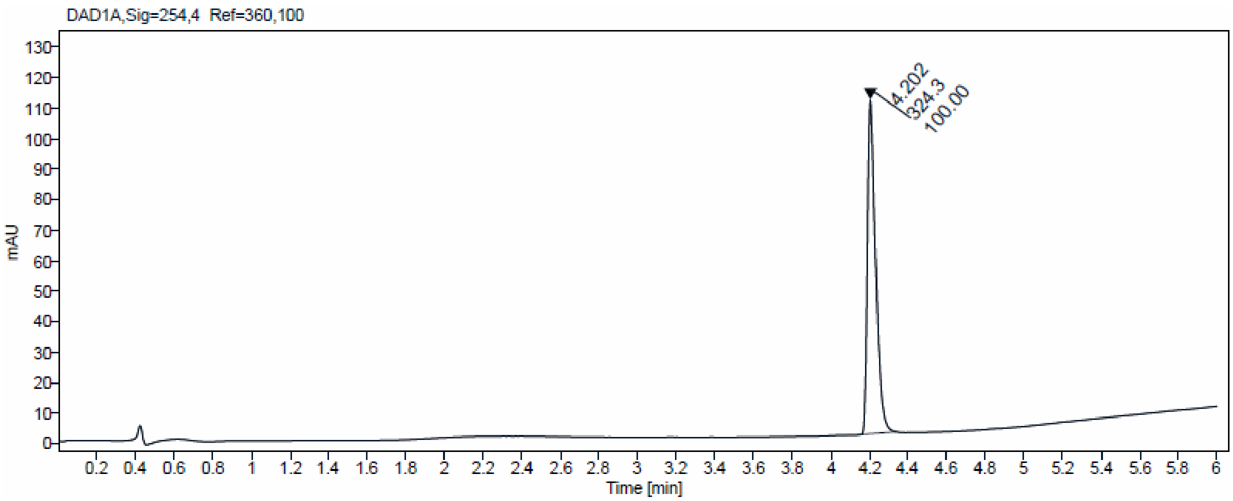

Supplementary Fig. 165. LC of IA44 [Injection Acquisition Method C18_C1_10-100% (Water/Acetonitrile) _6min.amx]

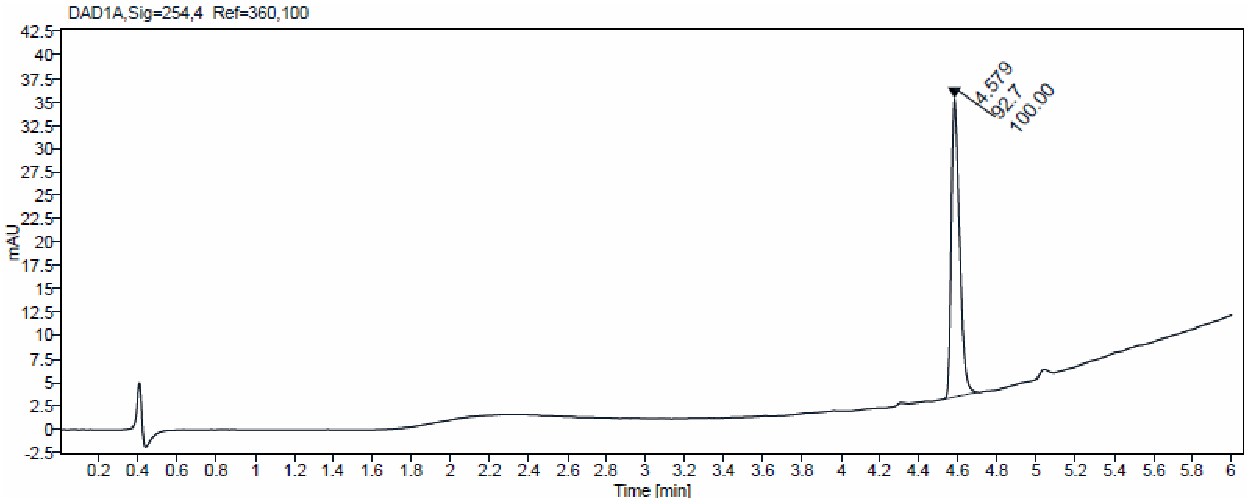

Supplementary Fig. 166. LC of IA47 [Injection Acquisition Method C18_C2_5-95% (Water/Acetonitrile) _20min.amx]

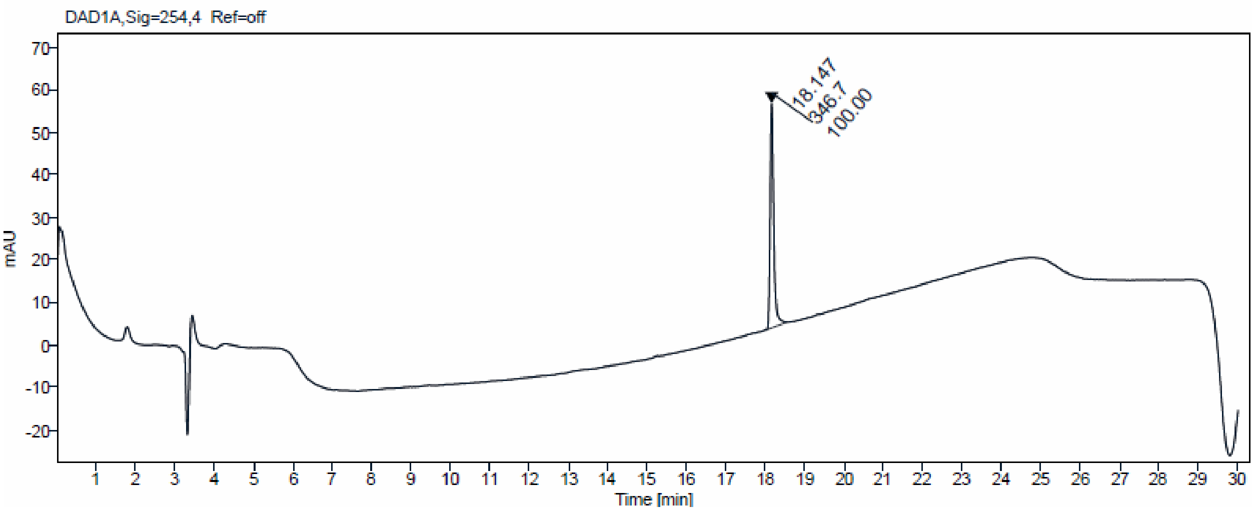

Supplementary Fig. 167. LC of IA048 [Injection Acquisition Method C18_C2_5-95% (Water/Acetonitrile) _20min.amx]

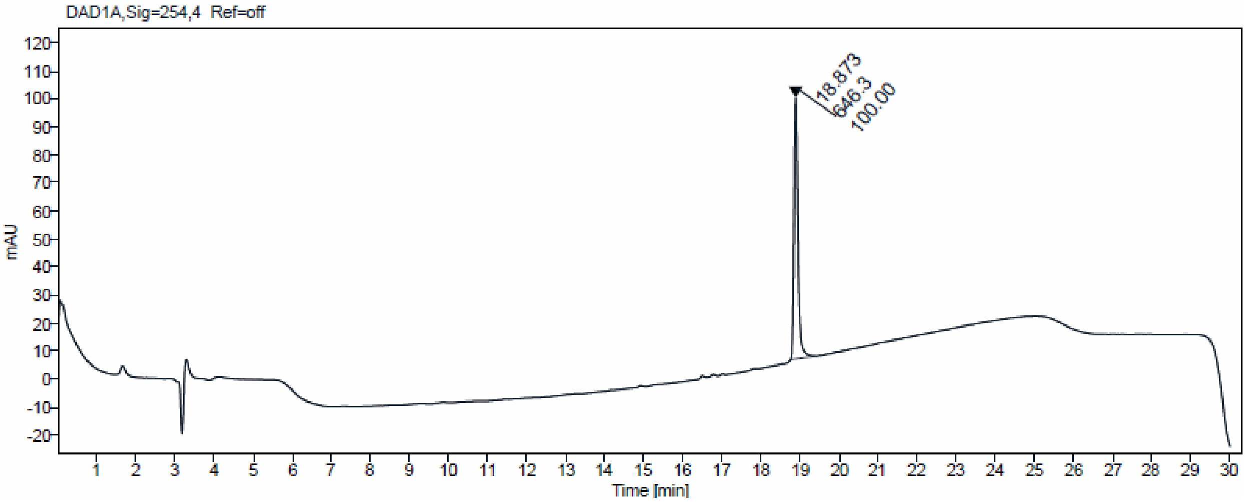

Supplementary Fig. 168. LC of IA60 [Injection Acquisition Method C18_C1_10-100% (Water/Acetonitrile) _6min.amx]

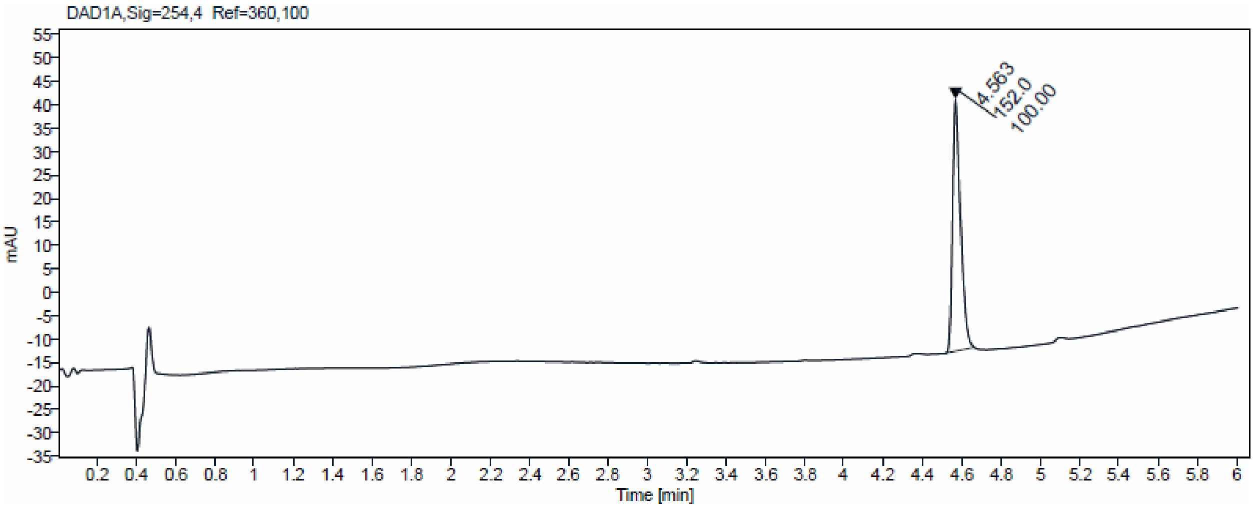

Supplementary Fig. 169. LC of IA63 [Injection Acquisition Method C18_C1_10-100% (Water/Acetonitrile) _6min.amx]

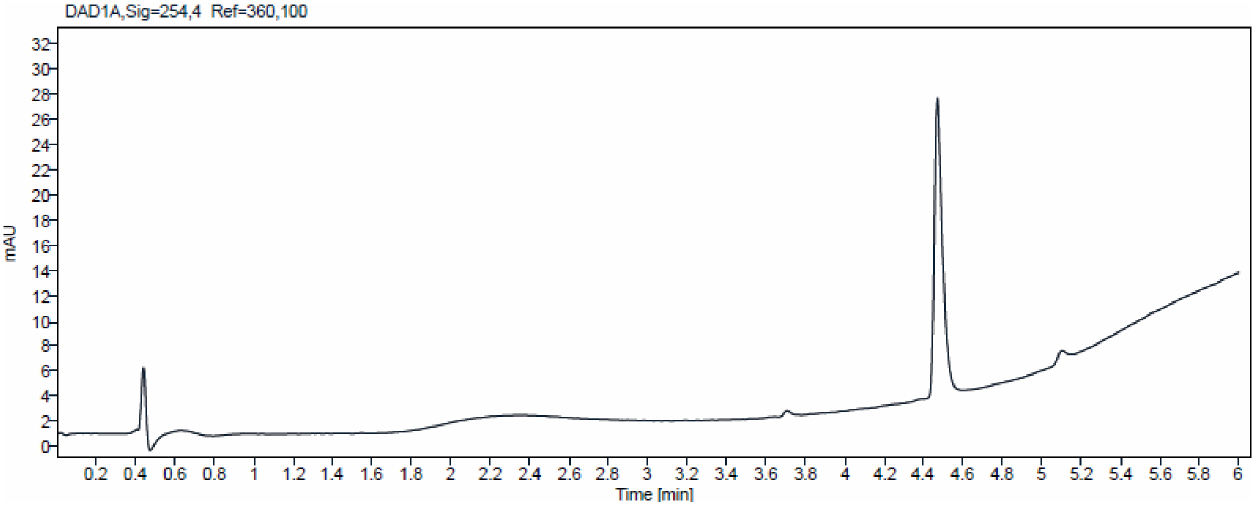

Supplementary Fig. 170. LC of G-1749 [Injection Acquisition Method C18_C2_5-95% (Water/Acetonitrile) _20min.amx]

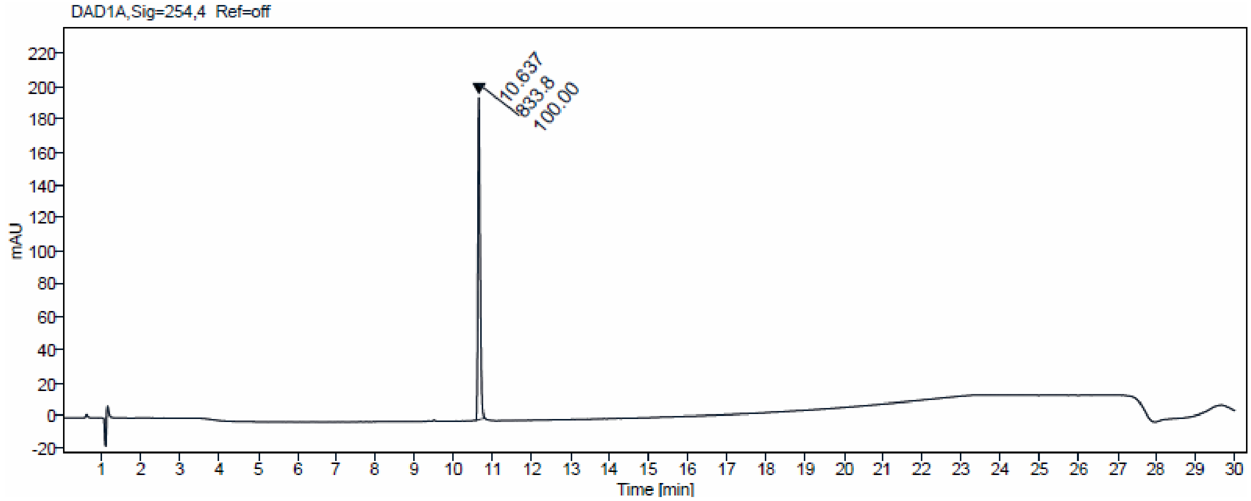

Supplementary Fig. 171. LC of AMG-18 [Injection Acquisition Method C18_C2_5-95% (Water/Acetonitrile) _20min.amx]

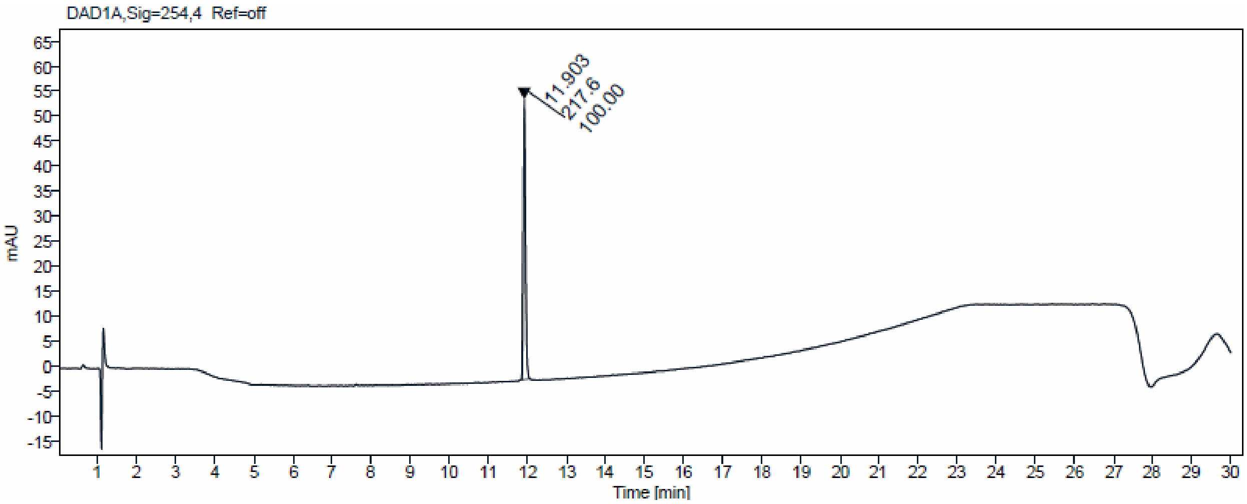

REFERENCES

1. Rosenbaum, C.; Röhrs, S.; Müller, O.; Waldmann, H., Modulation of MRP-1-mediated multidrug resistance by indomethacin analogues. *J Med Chem* **2005,** *48* (4), 1179-87.

2. Wey, S. J.; Augustyniak, M. E.; Cochran, E. D.; Ellis, J. L.; Fang, X.; Garvey, D. S.; Janero, D. R.; Letts, L. G.; Martino, A. M.; Melim, T. L.; Murty, M. G.; Richardson, S. K.; Schroeder, J. D.; Selig, W. M.; Trocha, A. M.; Wexler, R. S.; Young, D. V.; Zemtseva, I. S.; Zifcak, B. M., Structure-based design, synthesis, and biological evaluation of indomethacin derivatives as cyclooxygenase-2 inhibiting nitric oxide donors. *J Med Chem* **2007,** *50* (25), 6367-82.

3. Chambers, S. J.; Coulthard, G.; Unsworth, W. P.; O'Brien, P.; Taylor, R. J. K., From Heteroaromatic Acids and Imines to Azaspirocycles: Stereoselective Synthesis and 3D Shape Analysis. *Chemistry – A European Journal* **2016,** *22* (19), 6496-6500.

4. Ferri, E.; Le Thomas, A.; Wallweber, H. A.; Day, E. S.; Walters, B. T.; Kaufman, S. E.; Braun, M.-G.; Clark, K. R.; Beresini, M. H.; Mortara, K.; Chen, Y.-C. A.; Canter, B.; Phung, W.; Liu, P. S.; Lammens, A.; Ashkenazi, A.; Rudolph, J.; Wang, W., Activation of the IRE1 RNase through remodeling of the kinase front pocket by ATP-competitive ligands. *Nat Commun* **2020,** *11* (1), 6387.

5. Harrington, P. E.; Biswas, K.; Malwitz, D.; Tasker, A. S.; Mohr, C.; Andrews, K. L.; Dellamaggiore, K.; Kendall, R.; Beckmann, H.; Jaeckel, P.; Materna-Reichelt, S.; Allen, J. R.; Lipford, J. R., Unfolded Protein Response in Cancer: IRE1α Inhibition by Selective Kinase Ligands Does Not Impair Tumor Cell Viability. *ACS Med Chem Lett* **2015,** *6* (1), 68-72.
