# Supplementary material for: Harnessing indole scaffolds to identify small-molecule IRE1α inhibitors modulating XBP1 mRNA splicing
Source: Nat Commun. 2025 Sep 26;16:8531. doi: 10.1038/s41467-025-64291-4 (PMC12475274; doi:10.1038/s41467-025-64291-4)
Supplement: Supplementary file 2 — Reporting Summary [file 41467_2025_64291_MOESM2_ESM.pdf]

nature portfolio

Corresponding author(s): Peng Wu

Last updated by author(s): Sep 5, 2025

Reporting Summary

Nature Portfolio wishes to improve the reproducibility of the work that we publish. This form provides structure for consistency and transparency in reporting. For further information on Nature Portfolio policies, see our Editorial Policies and the Editorial Policy Checklist.

Statistics

For all statistical analyses, confirm that the following items are present in the figure legend, table legend, main text, or Methods section.

n/a	Confirmed	
☐	☒	The exact sample size (*n*) for each experimental group/condition, given as a discrete number and unit of measurement
☐	☒	A statement on whether measurements were taken from distinct samples or whether the same sample was measured repeatedly
☐	☒	The statistical test(s) used AND whether they are one- or two-sided<br>*Only common tests should be described solely by name; describe more complex techniques in the Methods section.*
☒	☐	A description of all covariates tested
☒	☐	A description of any assumptions or corrections, such as tests of normality and adjustment for multiple comparisons
☐	☒	A full description of the statistical parameters including central tendency (e.g. means) or other basic estimates (e.g. regression coefficient) AND variation (e.g. standard deviation) or associated estimates of uncertainty (e.g. confidence intervals)
☐	☒	For null hypothesis testing, the test statistic (e.g. *F*, *t*, *r*) with confidence intervals, effect sizes, degrees of freedom and *P* value noted<br>*Give P values as exact values whenever suitable.*
☒	☐	For Bayesian analysis, information on the choice of priors and Markov chain Monte Carlo settings
☒	☐	For hierarchical and complex designs, identification of the appropriate level for tests and full reporting of outcomes
☒	☐	Estimates of effect sizes (e.g. Cohen's *d*, Pearson's *r*), indicating how they were calculated

Our web collection on statistics for biologists contains articles on many of the points above.

Software and code

Policy information about availability of computer code

Data collection	Tecan SparkControl 2.3, Agilent Openlab 2.4.0.628, MicroCal PEAQ-ITC Control Software V1.41, Refeyn AcquireMP V2024 R2, MO.Control software V1.6.1, CXF Maestro 4.1.2433. Synchrotron data was collected from the ID30A-3 beamline at the European Synchrotron Radiation Facility (ESRF)
Data analysis	Graphpad Prism 9, Microsoft excel 16.60, ProMass 3.0 rev12, Phenix 1.20.1-4487, CCP4 9.0.003, Coot 0.9.6, XDS 2024, Pymol 3.0.3, MicroCal PEAQ-ITC analysis software V1.41, Refeyn DiscoverMP V2024 R2, MO. Affinity Analysis V2.3, MestReNova, Agilent OpenLAB CDS.

For manuscripts utilizing custom algorithms or software that are central to the research but not yet described in published literature, software must be made available to editors and reviewers. We strongly encourage code deposition in a community repository (e.g. GitHub). See the Nature Portfolio guidelines for submitting code & software for further information.

Data

Policy information about availability of data

All manuscripts must include a data availability statement. This statement should provide the following information, where applicable:

- Accession codes, unique identifiers, or web links for publicly available datasets
- A description of any restrictions on data availability
- For clinical datasets or third party data, please ensure that the statement adheres to our policy

Data supporting the findings of the study are available from the corresponding author upon request. The protein X-ray crystal data generated in this study have

been deposited in the PDB database under accession code 9GOW. Compound characterizations are provided in the Supplementary Information. All other data are included in the manuscript or in the supplementary Information. Source data for uncropped gels and blots, biochemical experiments and biophysical experiments are provided with this paper. Previously deposited PDB structures 4PL3, 6W3C, 6W39, 6W3E, 6URC were used for modeling, comparison, and alignment. IRE1α protein sequence is available through UniProt under the code O75460. Source Data are provided with this paper

Research involving human participants, their data, or biological material

Policy information about studies with human participants or human data. See also policy information about sex, gender (identity/presentation), and sexual orientation and race, ethnicity and racism.

Reporting on sex and gender	N/A
Reporting on race, ethnicity, or other socially relevant groupings	N/A
Population characteristics	N/A
Recruitment	N/A
Ethics oversight	N/A

Note that full information on the approval of the study protocol must also be provided in the manuscript.

Field-specific reporting

Please select the one below that is the best fit for your research. If you are not sure, read the appropriate sections before making your selection.

☒ Life sciences ☐ Behavioural & social sciences ☐ Ecological, evolutionary & environmental sciences

For a reference copy of the document with all sections, see nature.com/documents/nr-reporting-summary-flat.pdf

Life sciences study design

All studies must disclose on these points even when the disclosure is negative.

Sample size	For quantitative experiments, a sample size of at least 2, typically 3 to 5 independent experiment was chosen in line with what is the standard of the field in the molecular biosciences.<br>For non-quantitative experiments, a sample size of at least 2, typically 3 to 4 independent experiment was chosen in line with what is the standard of the field in the molecular biosciences.
Data exclusions	For the crystal structure processing, part of chain D (K851 to M948) was eliminated due to the poor electron density data.
Replication	All observations were made in at least two independent experiments, all with consistent results. The kinase activity test from the Thermo Fisher SelectScreen service was performed with duplicates.
Randomization	Randomization was not applicable as no experiments involving humans or animals, and no experiments that might be sensitive to the order of measurement / treatment were performed.
Blinding	Blinding was not carried out as no subjective analysis was performed.

Reporting for specific materials, systems and methods

We require information from authors about some types of materials, experimental systems and methods used in many studies. Here, indicate whether each material, system or method listed is relevant to your study. If you are not sure if a list item applies to your research, read the appropriate section before selecting a response.

Materials & experimental systems

n/a	Involved in the study
☐	☒ Antibodies
☐	☒ Eukaryotic cell lines
☒	☐ Palaeontology and archaeology
☒	☐ Animals and other organisms
☒	☐ Clinical data
☒	☐ Dual use research of concern
☒	☐ Plants

Methods

n/a	Involved in the study
☒	☐ ChIP-seq
☒	☐ Flow cytometry
☒	☐ MRI-based neuroimaging

Antibodies

Antibodies used

IRE1α (14C10) Rabbit mAb (Cell signaling technology, #3294s), dilution 1:1000.
XBP-1s (D2C1F) Rabbit mAb (Cell Signaling Technology, #12782T), dilution 1:1000.
GAPDH Rabbit Polyclonal antibody (Proteintech, 10494-1-AP), dilution 1:6000.
HRP-conjugated Goat Anti-Rabbit IgG(H+L) (Proteintech, SA00001-2), dilution 1:6000.

Validation

All antibodies are validated for the application of Western Blotting on human protein as per statements on the manufacturers' websites.

IRE1α (14C10) Rabbit mAb (Cell signaling technology, #3294s). Validated by manufacturer for WB and IP in various cell lines. Details see manufacturer's website, https://www.cellsignal.com/products/primary-antibodies/ire1a-14c10-rabbit-mab/3294
XBP-1s (D2C1F) Rabbit mAb (Cell Signaling Technology, #12782T). Validated by manufacturer for WB in various cell lines. Details see manufacturer's website, https://www.cellsignal.com/products/primary-antibodies/xbp-1s-d2c1f-rabbit-mab/12782
GAPDH Rabbit Polyclonal antibody (Proteintech, 10494-1-AP). Validated by manufacturer for WB, IP, IHC, IF/ICC and FC in various samples. Details see manufacturer's website. https://www.ptglab.com/products/GAPDH-Antibody-10494-1-AP.htm
HRP-conjugated Goat Anti-Rabbit IgG(H+L) (Proteintech, SA00001-2). Validated by manufacturer for IP. Details see manufacturer's website. https://www.ptglab.com/products/HRP-conjugated-Affinipure-Goat-Anti-Rabbit-IgG-H-L-secondary-antibody.htm

Eukaryotic cell lines

Policy information about cell lines and Sex and Gender in Research

Cell line source(s)

A549, HT-29, and MDA-MB-468 were obtained from ATCC (CCL-185, HTB-38, HTB-132), HCT 116 and MDA-MB-231 cell lines were purchased from DSMZ (ACC581, ACC732). The Sf9 cells were obtained form Protein Chemistry Facility of MPI Dortmund.

Authentication

Cell lines were used without further authentication

Mycoplasma contamination

Cells were regularly tested for mycoplasma contamination, with consistently negative results.

Commonly misidentified lines
(See ICLAC register)

No commonly misidentified cell lines were used in this study

Plants

Seed stocks

N/A

Novel plant genotypes

N/A

Authentication

N/A
